# *TaDrAp1* and *TaDrAp2*, Partner Genes of a Transcription Repressor, Coordinate Plant Development and Drought Tolerance in Spelt and Bread Wheat

**DOI:** 10.3390/ijms21218296

**Published:** 2020-11-05

**Authors:** Lyudmila Zotova, Nasgul Shamambaeva, Katso Lethola, Badr Alharthi, Valeriya Vavilova, Svetlana E. Smolenskaya, Nikolay P. Goncharov, Akhylbek Kurishbayev, Satyvaldy Jatayev, Narendra K. Gupta, Sunita Gupta, Carly Schramm, Peter A. Anderson, Colin L. D. Jenkins, Kathleen L. Soole, Yuri Shavrukov

**Affiliations:** 1 Faculty of Agronomy, S. Seifullin Kazakh AgroTechnical University, Nur-Sultan 010000, Kazakhstan; lupezo_83@mail.ru (L.Z.); nazgulya.9797@mail.ru (N.S.); agun.rektor@gmail.com (A.K.); 2 College of Science and Engineering, Biological Sciences, Flinders University, Adelaide, SA 5042, Australia; letholakeka@gmail.com (K.L.); b.harthi@tu.edu.sa (B.A.); carly.schramm@flinders.edu.au (C.S.); peter.anderson@flinders.edu.au (P.A.A.); colin.jenkins@flinders.edu.au (C.L.D.J.); kathleen.soole@flinders.edu.au (K.L.S.); 3 Institute of Cytology and Genetics, Russian Academy of Sciences, Siberian Branch, 630090 Novosibirsk, Russia; valeriya.vavilova@gmail.com (V.V.); svsmol@ngs.ru (S.E.S.); gonch@bionet.nsc.ru (N.P.G.); 4 Department of Plant Physiology, SKN Agriculture University, Jobner 303329, Rajasthan, India; nkgupta69@yahoo.co.in (N.K.G.); sunita.pphy.rari@sknau.ac.in (S.G.)

**Keywords:** down-regulator associated protein (DrAp1), drought, gene expression, microarray 40K SNP assay, negative cofactor 2 (NC2), nuclear factor Y (NF-Y), plant genotyping, single nucleotide polymorphism (SNP), transcription factor (TF), wheat (bread and spelt)

## Abstract

Down-regulator associated protein, DrAp1, acts as a negative cofactor (NC2α) in a transcription repressor complex together with another subunit, down-regulator Dr1 (NC2β). In binding to promotors and regulating the initiation of transcription of various genes, *DrAp1* plays a key role in plant transition to flowering and ultimately in seed production. *TaDrAp1* and *TaDrAp2* genes were identified, and their expression and genetic polymorphism were studied using bioinformatics, qPCR analyses, a 40K Single nucleotide polymorphism (SNP) microarray, and Amplifluor-like SNP genotyping in cultivars of bread wheat (*Triticum aestivum* L.) and breeding lines developed from a cross between spelt (*T. spelta* L.) and bread wheat. *TaDrAp1* was highly expressed under non-stressed conditions, and at flowering, *TaDrAp1* expression was negatively correlated with yield capacity. *TaDrAp2* showed a consistently low level of mRNA production. Drought caused changes in the expression of both *TaDrAp1* and *TaDrAp2* genes in opposite directions, effectively increasing expression in lower yielding cultivars. The microarray 40K SNP assay and Amplifluor-like SNP marker, revealed clear scores and allele discriminations for *TaDrAp1* and *TaDrAp2* and *TaRht-B1* genes. Alleles of two particular homeologs, *TaDrAp1-B4* and *TaDrAp2-B1*, co-segregated with grain yield in nine selected breeding lines. This indicated an important regulatory role for both *TaDrAp1* and *TaDrAp2* genes in plant growth, ontogenesis, and drought tolerance in bread and spelt wheat.

## 1. Introduction

Plant development and responses to the environment are regulated by a very complex hierarchical gene network. Products of regulatory genes act either as activators or repressors to enhance or suppress downstream genes, respectively. Transcription factors (TFs) represent an extremely diverse group of such regulatory gene products, where the majority play an activating role, but then must often be deactivated at a later time. A smaller number of TFs can function as repressors or dual-acting regulators depending on specific conditions [1]. There is an unexpected paradox of TF activators versus repressors based on the simple statement that after activation of any TF gene it must, by definition, be earlier or later repressed, deactivated, and returned back to its initial level. In the situation of much smaller numbers of known repressor TF genes, their actions could be wider and even more general as universal negative deactivators of other genes [2].

The interest in general transcription repressors first occurred in medical and microbiological research, where two negative cofactors, NC1 and NC2, were described [3,4]. These negative cofactors bind TATA boxes in the promoters of transcription factor IID (TFIID) and regulate the start of gene transcription via production of a stable complex between repressors and activators [5,6,7,8]. The first cofactor, NC1, was only identified in humans by chromatographic distinction from others [9], but in the absence of sequence annotation, it has never been found in plants. In contrast, the second negative cofactor NC2 is well established, and highly conserved among all eukaryotic organisms [2,10,11]. In plants, negative cofactor NC2 is more typically designated as a general repressor complex, comprising two subunits or partners, down-regulator, Dr1 (NC2β) and down-regulator associated protein, DrAp1 (NC2α), that mostly act together as co-repressors [2,12,13].

Dr1 (NC2β) has been reported to have the strongest similarity to class B, nuclear factor Y (NF-YB, synonyms HAP3 and CBF-A). The high homology between Dr1 and NF-YB was apparent in the amino acid sequence and the shared histone-fold motif shown in yeast and humans [5,11,12,14]. In *Arabidopsis thaliana* (L.) Heynh., AtNF-YB12 and AtNF-YB13 are designated as AtDr1 (NC2β1, At5g08190) and AtDr2 (NC2β2, At5g23090), indicating that the corresponding two *AtDr* genes encode two down-regulators and are included as members of the TF class, NF-YB [15]. In bread wheat (*Triticum aestivum* L.), a high degree of similarity in amino acid sequence has been reported between the two homeologs, TaDr1A (AF464903) and TaDr1B (TC416575), and TaNF-YB3, which were all initially described as independent genes [16]. However, a recent finding of a third homeolog within the complete set of TaDr1 (BC000036325) genes in bread wheat exhibited only a low similarity score and 18.9% identity with TaNF-YB3 at the amino acid level [17].

DrAp1 (NC2α) shows similarity and extensive homology with NF-YC (synonyms HAP5 and CBF-C), especially in the histone-fold domain in the N-terminus and proline-rich carboxy-terminal end in yeast [18] and in humans [11,12]. A more complicated situation arose in the determination of DrAp1 homeology in plants. In *A. thaliana*, AtDrAp1 was identified as a member of class C in NF-Y and called AtNF-YC11 [19]. However, in rice (*Oriza sativa* L.), it was reported that two down-regulator associated proteins, OsDrAp1 (AF464904) and OsDrAp2 (AB288048), showed 40% and 37% identity, respectively, with the OsNF-YC (OsHAP5) class of proteins, and hence were excluded from the NF-YC family and arranged as an independent cluster [20]. Unexpectedly, the *OsDrAp2* gene (Os05g41450) was identified as ‘HAP2-LIKE 1′ (similar to the NF-YA group) [21], but this was not sufficiently supported by the supplementary data and was therefore likely misinterpreted. In bread wheat, two genes, *TaDrAp1* (TC233433) and *TaDrAp2* (TC241235), were identified and designated as *TaNF-YC6* and *TaNF-YC8*, respectively [16,22], despite their quite isolated phylogenetic clade location among other *TaNF-YC* genes [16].

A clear difference in the number of *DrAp1* genes in yeast, animals, and humans is apparent compared with the genes found in dicot and monocot plant species. All non-plant organisms seem to have only a single copy of the *DrAp1* gene encoding down-regulator associated protein DrAp1. For example, in yeast [18,23], fruit fly *Drosophila* [24], and humans [12], only one *DrAp1* gene was found. In dicot plants, starting with the model species *A. thaliana*, only a single *DrAp1* gene was reported in each species [15,19].

In contrast, all monocot plant species seem to have a duplicated copy of the gene designated as *DrAp1* and *DrAp2*, extending right back to rice [20,21,25] and the model species *Brachypodium distachyon* (L.) P.Beauv. [15,26]. In bread wheat, Stephenson et al. [16] correctly identified the wheat homeolog *TaDrAp1* (TC233433) to the rice *OsDrAp1* (Os11g34200), and in a later study, two genes in bread wheat, *TaDrAp1* (TaNC2α1) and *TaDrAp2* (TaNC2α2), were described [15], with three homeologs of each gene due to the hexaploid nature of the wheat genome [22].

The two down-regulators, Dr1 and DrAp1, do not repress initiation of transcription individually, but together form a heterodimer complex. This Dr1/DrAp1 (NC2β+α) complex, acting as a general co-repressor, enhances repression activity by targeting the TATA-box-binding protein (TBP), finally blocking the initiation of transcription. This was reported in yeast [27], *Drosophila* [24], and humans [6,12,13] but also in plants, including *Arabidopsis* [19] and rice [25]. In rice, it has been found that the Dr1 or DrAp1 proteins interact separately with the TBP-DNA complex, forming heterodimers that strongly interact with the TBP-DNA complex to form larger complexes. However, it was reported that OsDrAp1 had stronger repression activity compared with the relatively weak repression of the partner protein OsDr1 [25].

Under drought stress, *TaDr1* genes have been shown to be upregulated at least two-fold compared with controls, while neither *TaDrAp1* nor *TaDrAp2* was identified in this experiment in the response of bread wheat plants to drought [16]. Similar results were reported in eight bread wheat cultivars grown under drought. The total transcript level of *TaDr1* was increased by 2–2.4-fold in four high-yielding cultivars, while a more modest increased expression of *TaDr1* was found in four other cultivars with lower yield performance [17]. To our knowledge, there is no published information about the down-regulator associated proteins and expression analyses of corresponding genes, *TaDrAp1* or *TaDrAp2*, in wheat under drought or any other stress condition.

The regulatory roles of *DrAp* genes encoding down-regulator associated proteins have been reported to be quite variable across plant species and reveal involvement in many plant development processes. In *A. thaliana*, *AtDrAp1* (*NF-YC11/NC2α*) was shown to be involved in the response of plants to photoperiod, transition to reproductive stage, and time to flowering. It was reported as a mechanism of meristem identity and maintenance of redox-dependent signal transduction [19]. Similarly, in rice, the *OsDrAp2* gene (*OsHAPL1* = Os05g41450) interacted with genes regulating date to heading, (*DTH8*), heading date (*Hd1*), early heading date (*Ehd1*), and flowering locus T1 (*FT1*), strongly affecting the transition time to reproductive development [21].

A recent model for plant reaction to environmental signals also suggested the regulation of the *FT1* gene (synonym, *Vrn3*) via interactions between subunit balances of NF-YA/B/C in TF complexes and CCT domain proteins in downregulated genes affecting the initiation of flowering [28,29]. In wheat, a wider overview indicated that the transition to flowering, as a complex trait, is controlled by *TaVrn1*, *TaVrn2*, and *TaFT1*, and is regulated by a combination of several interacting subunit proteins from both TaNF-YB and TaNF-YC groups [30]. In addition, it has been confirmed recently that the down-regulator gene, *TaDr1*, closely related to the class *NF-YB* (*HAP3*) TF, showed a similar co-expression pattern for *TaFT1* and *TaVrn1* genes [17]. Currently, there is no published information about the involvement of *TaDrAp1* or *TaDrAp2* genes in the regulation of flowering time in wheat, but the above mentioned *AtDrAp1* (*NF-YC11*) in *A. thaliana* and *OsDrAp2* (*OsHAPL1* = Os05g41450) in rice showed very strong impacts on the time to transition to flowering [19,21]. Therefore, we can speculate that *TaDrAp1* (similar to *A. thaliana AtDrAp1*), *TaDrAp2* (similar to rice *OsDrAp2*), or both genes together could be a part of a regulatory network modulating flowering time in wheat plants.

Interestingly, the *OsNF-YB11* gene (*Ghd8* = Os08g07740) in rice was associated not only with flowering time, but also with plant height and grain yield. Rice plants of near-isogenic lines (NIL) carrying the *Ghd8* allele of one parent, NIL^HR5^, showed slightly delayed heading by 7.8 days, a taller stature by 17.3 cm in plant height, and 50% increased grain yield due to significantly more spikelets and seeds per panicle [31]. As mentioned above, the *OsDrAp2* gene (*OsHAPL1* = Os05g41450) plays an important role in rice plant development, time to heading, and flowering. It was also reported that overexpression of *OsDrAp2* in transgenic rice caused taller plants (30–65%), with delayed flowering time and with 2–3.5-fold more seeds per panicle via significantly more primary and secondary branching [21]. Therefore, the pleiotropic effect of NF-Y complex proteins, including Dr1/DrAp1, seems to strongly regulate plant development including flowering time, plant height, and seed productivity, as shown in rice.

Single nucleotide polymorphism (SNP) is one of the most popular methods for molecular genetic analysis of plants [32,33], including wheat [34]. There are two widely known approaches using either a microchip array or individual SNPs. Microarray technology is very well developed and has been successfully applied in the simultaneous analysis of thousands of SNPs, but with only a limited number of genotypes in bread wheat [35,36,37], including those from Kazakhstan [38], and in spelt wheat [39,40]. In contrast, analyses of individual SNPs are based on direct sequencing of targeted genes. There are many methods for SNP analysis in plants [41,42,43,44,45]. Amplifluor or Amplifluor-like (amplification with fluorescence) analysis [17,46,47,48] is convenient for a wide range of SNP genotyping across many plant species, including wheat [41].

Bread wheat (*T. aestivum*) is the largest crop globally in terms of production and food consumption. Spelt wheat (*T. spelta* L.) represents a very closely related species with a similar hexaploid (2*n* = 6*x* = 42) BBAADD genome [49]. Despite some debate, spelt wheat seems to be not an ancestor, but rather an ancient species closely related to bread wheat [50]. Spelt wheat was domesticated and cultivated over thousands of years from the time of the Fertile Crescent, and its hulled seeds are now regarded as an alternative to naked bread wheat [51]. The interest in spelt wheat as an ancient grain is growing rapidly throughout the world [52] for the sustainable nutritional supply of grain protein, fiber, minerals, and phytochemicals [53]; its compositional profile, baking quality, and technological potential to supply raw and functional ingredients [54,55]; and its high antioxidant capacity [56].

Quality and nutrient characteristics are not the only attractive traits promoting the study and inclusion of spelt wheat genotypes in ancient grains and wheat breeding. Many *T. spelta* germplasms are resistant to various diseases [39], and they are easily grown with a low fertilizer input, which is important for organic agriculture and production of ancient grains for a growing market of consumers conscious of health and the environment [55]. Unique alleles were also recently reported in spelt wheat that confer longer root hairs, an important trait for better phosphorous uptake in plants [57].

Spelt and bread wheat are easily crossed for breeding, where spike type, hulled seeds, and taller stature traits are inherited. However, *T. spelta* germplasms were often reported as low yielding and not adapted to modern agricultural practices [51], and spelts have a low ability for heterosis (hybrid vigor). Nonetheless, spelt wheat is still identified as an interesting resource for pre-breeding programs in bread wheat [58]. The improvement of spelt is possible using a hybrid breeding program via crossings with bread wheat, where agronomically important traits can be introgressed, including better tolerance to abiotic stresses and sustainable yield of hulled grains in the selected breeding lines. Interspecies crosses always enrich the gene pool of the genetic and breeding material [59], and they have also enabled the study of inheritance, genetic polymorphisms, and expression of the identified *TaDrAp1* and *TaDrAp2* genes in the current study.

Initially, two mRNA sequences of *TaDrAp1* and *TaDrAp2* genes encoding two down-regulator associated proteins were annotated (TC233433 and TC241235) [16]. Six homeologs of both genes were declared later based on the sequence of cv. Chinese Spring in the list of all NF-Y genes in wheat, but the genes were noted as *TaNF-YC6* and *TaNF-Y8* without any analysis [22]. Given this gap in the literature, the aims of the current study included: (1) identification and bioinformatic analysis of six homeologs of both *TaDrAp1* and *TaDrAp2* for gene structure and similarity with other genes within bread wheat and among various plant species; (2) expression analysis of two genes, *TaDrAp1* and *TaDrAp2*, including each of the six homeologs, during plant ontogenesis and in response to drought stress in bread wheat cultivars; (3) microarray 40K SNP analysis of parents and selected breeding lines from the interspecies hybrid cross No. 18-6 between spelt and bread wheat; and (4) sequencing and genetic polymorphism analysis in gene homeologs *TaDrAp1* and *TaDrAp2* in wheat cultivars and spelt and bread wheat parents using Amplifluor-like SNP genotyping.

## 2. Results

### 2.1. Identification of Homeologs TaDrAp1 and TaDrAp2 Genes in Bread Wheat cv. Chinese Spring Using Public Databases

In bread wheat, two genes, *TaDrAp1* and *TaDrAp2*, were identified and retrieved from the full annotated sequence of cv. Chinese Spring from the Gramene database (http://www.gramene.org). The order of these two genes was confirmed as published earlier for rice *OsDrAp1* (AF464904 = LOC_Os11g34200 [25]) and *OsDrAp2* (AB288048 [20], *OsHAPL1* = LOC_Os05g41450 [21]), and in *Brachypodium distachyon*, *BdDrAp1* and *BdDrAp2* (*Bd-NC2α2* = Bradi4g16840 and *Bd-NC2α1* = Bradi2g21290, respectively [15,26]).

Three homeologous copies were identified for each of the two genes, *TaDrAp1* and *TaDrAp2* (Table 1). To simplify the designation of each homeolog for both genes, genome and chromosome group were added to each gene name. The length of the open reading frame (ORF) differed in the wheat B genome for both genes, approximately twice as long in *TaDrAp1-B4* (10Kb), but about 320 bp shorter in *TaDrAp2-B1* (Table 1).

These differences in gene lengths were directly related to the third intron, which was virtually doubled in length in *TaDrAp1-B4*, but was shorter in *TaDrAp2-B1*. Nevertheless, the length of coding regions for the homeologs of the two genes was quite similar and varied in only one triplet, resulting in a single amino acid difference in the encoded proteins (Table 1).

**Figure 1:**
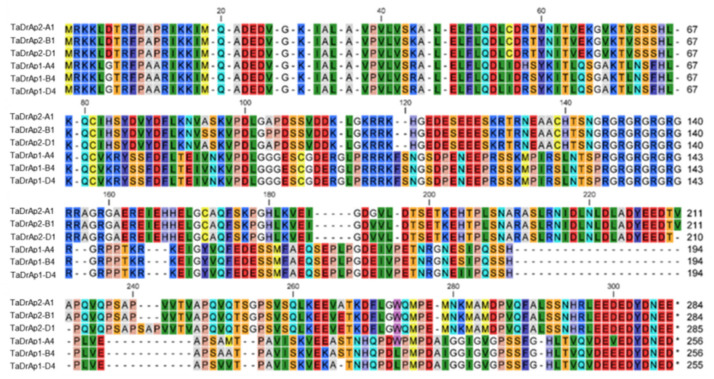
ClustalW analysis of amino acid comparisons between homeologs of two down-regulator associated proteins, TaDrAp1 and TaDrAp2, from the Gramene database with sequence of cv. Chinese Spring, using CLC Main Workbench software.

Full genomic sequences of the identified *TaDrAp1* and *TaDrAp2* genes with the exons/introns, Open reading frames (ORF), Coding sequences (CDS), and encoded polypeptides indicated are presented in Supplementary Material File S1. Protein sequence comparisons revealed 96.0–98.6% identity among homeologs within *TaDrAp1* and *TaDrAp2*, while the molecular similarity between homeologs of each gene accounted for only 36.9–39.8% identity (Figure 1).

### 2.2. Molecular-Phylogenetic Analysis of Homeologs TaDrAp1 and TaDrAp2 in Bread Wheat Compared to Other Plant Species and References

**Figure 2:**
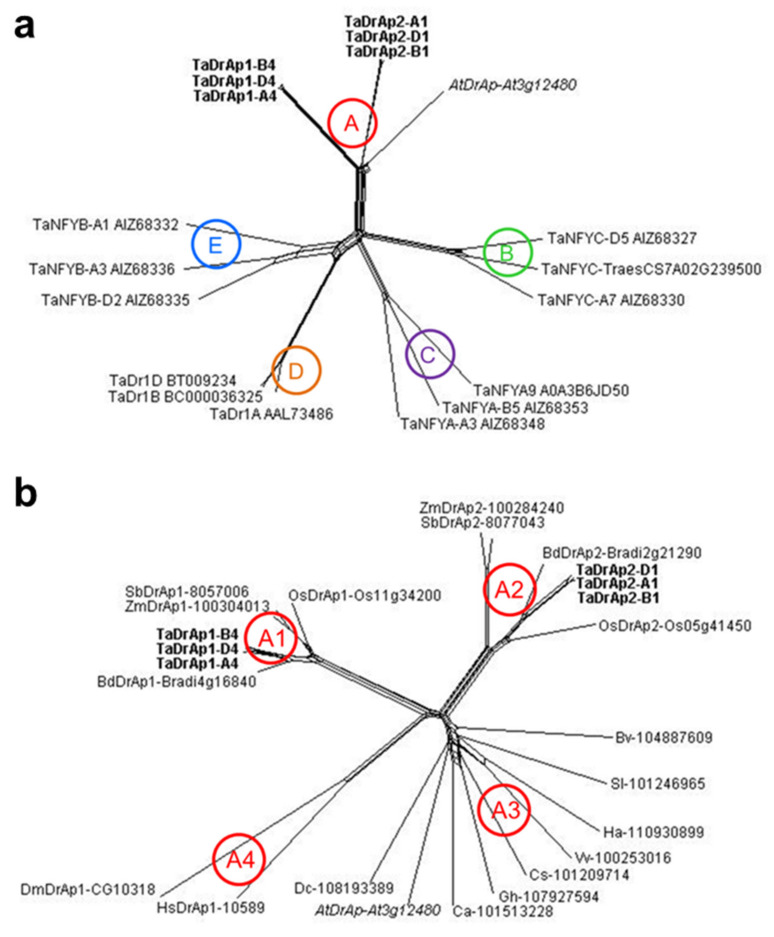
Molecular phylogenetic dendrograms prepared using the SplitsTree4 program for amino acids in two down-regulator associated proteins, DrAp1 and DrAp2, and down-regulator Dr1 and three groups of transcription factors Nuclear factor Y (NF-Y) in wheat (**a**); and extended clade A for DrAp1 and DrAp2 in monocot and dicot plant species and references (**b**). Target proteins are indicated in bold. Sequences were retrieved for bread wheat, Ta (*Triticum aestivum*), from the Gramene and KEGG databases; for rice, Os (*Oryza sativa*), from the rice genome annotation website; for Bd (*Brachypodium distachyon*), from the Brachypodium website; for At (*Arabidopsis thaliana*), indicated in italics as reference plant species, from the TAIR website; and for all other species from the KEGG database, with the following abbreviations in clockwise order: Sb, *Sorghum bicolor*; Zm, *Zea mays*; Bv, *Beta vulgaris*; Sl, *Solanum lycopersicum*; Ha, *Helianthus annuus*; Vv, *Vitis vinifera*; Cs, *Cucumis sativus*; Gh, *Gossypium hirsutum*; Ca, *Cicer arietinum*; Dc, *Daucus carota*; Hs, *Homo sapiens*; Dm, *Drosophila melanogaster*. All analyzed sequences are presented in Supplementary Material File S2.

Several rounds of comparison of TaDrAp1 and TaDrAp2 amino acid sequences were carried out to show similarities or differences with related polypeptides from other species. Within bread wheat (*T. aestivum*), sequences of three representative members were selected from each of three groups of NF-Y transcription factors: NF-YA, NF-YB, and NF-YC, in addition to homeologs of TaDr1 identified earlier [17], and also using the *Arabidopsis* AtDrAp protein as a reference (Figure 2a).

The dendrogram showed five distinct clades with significant molecular genetic differences among the protein accessions. Clade A clearly distinguished the TaDrAp1 and TaDrAp2 polypeptides in wheat and the reference protein AtDrAp from *Arabidopsis*. Homeologs of both polypeptides, TaDrAp1 and TaDrAp2, with corresponding genes located in chromosome groups 4 and 1, respectively, showed very strong conservation of the sequences. Three other clades (C, E, and B) containing isolated NF-YA, NF-YB, and NF-YC proteins, respectively, were clearly distinguished from each other. The last clade D, with three homeologs of TaDr1, was located closer to clade E, indicating some similarity with the group of TaNF-YB transcription factors (Figure 2a).

A comparison of all known DrAp1 and DrAp2 proteins in plant species was overcrowded and, therefore, only a few representative monocot and dicot plant species were selected and their molecular phylogeny is presented in Figure 2b. Clade A from Figure 2a was clearly split into subclades. Monocot plant species, without exception, contained both DrAp1 and DrAp2 proteins located in two subclades, A1 and A2.

In contrast, all dicot plant species had only a single DrAp1 protein, slightly divergent, but still all isolated in the single and distinct subclade A3. For humans and *Drosophila melanogaster*, a single DrAp1 protein each, used for reference, grouped together in the isolated subclade A4 (Figure 2b).

### 2.3. Expression Analysis of TaDrAp1 and TaDrAp2 Genes in Eight Cultivars of Bread Wheat

**Figure 3:**
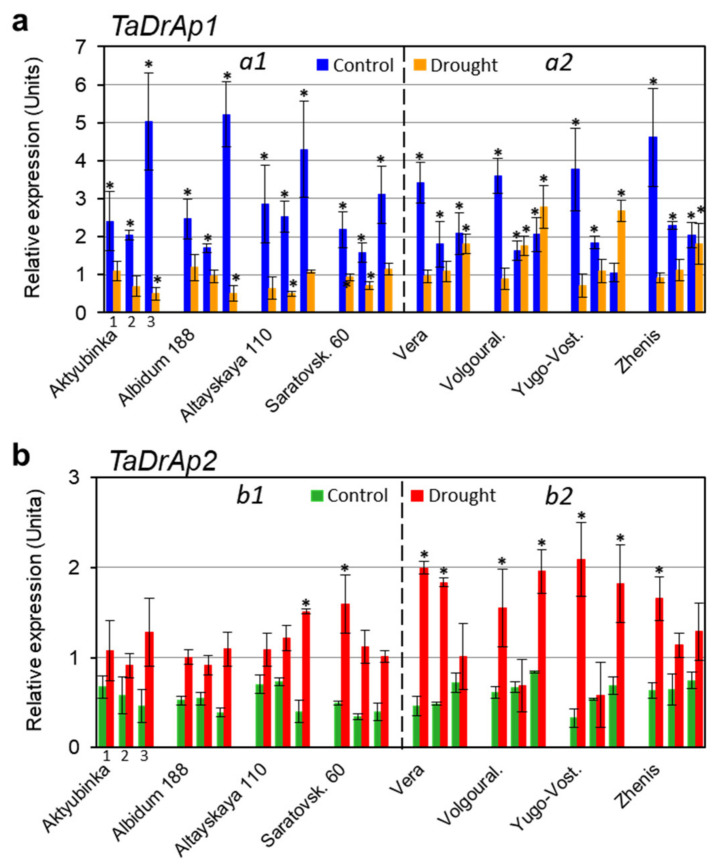
Expression analysis of *TaDrAp1* (**a**) and *TaDrAp2* (**b**) genes as a total of all homeologs for each gene across eight cultivars of bread wheat grown in Kazakhstan in control conditions and under moderate drought in pots with soil. Leaf samples were collected at three consecutive time-points for all genotypes as indicated by numbers for the first genotype: 1, vegetative stage, one month old; 2, tillering stage; 3, initiation of flowering stage. Wheat cultivars were arranged in two groups: high-yielding cultivars on the left part of the figure (Aktyubinka, Albidum 188, Altayskaya 110, and Saratovskaya 60) and low-yielding cultivars on the right (Vera, Volgouralskaya, Yugo-Vostochnaya, and Zhenis). Expression data were normalized using two reference genes, Ta22845 and Ta54825, and presented as the average ± SE of three biological and two technical replicates for each genotype and treatment. Differences from relative level 1 are designated by asterisks above the bars, indicating significant differences (*p* < 0.05) for each genotype and treatment calculated using ANOVA. Expression level in all control samples of *TaDrAp2* (**b**) was significantly below relative level 1, but not indicated by asterisks in the figure.

Experiments analyzing the expression of *TaDrAp1* and *TaDrAp2* genes across eight bread wheat cultivars grown in control conditions revealed a higher expression level for the *TaDrAp1* gene (2–5-fold above level 1 for reference genes), while the *TaDrAp2* gene in all samples showed an expression below level 1 (Figure 3).

Dynamic changes in gene expression during plant ontogenesis, even with just three time-points, showed variations among the different wheat genotypes. However, a clear difference was observed in *TaDrAp1* gene expression between four high-yielding (*a1*) and four low-yielding (*a2*) cultivars: relatively stable mRNA production sharply decreased from a high to moderate level at tillering in the *a2* group of cultivars, and increased with flowering in the *a1* cultivars. The expression level of *TaDrAp2* fluctuated during development in all samples, but remained below level 1 (Figure 3).

The situation changed dramatically when plants were exposed to drought at the same stages of ontogenesis. The expression of *TaDrAp1* dropped to a relative level of about 1 and even below 1 in some genotypes from the high-yielding group (*a1*), but remained at a higher level, by up to 2.7-fold, in the low-yielding genotypes (*a2*). In contrast, a significant increase in mRNA level from the *TaDrAp2* gene was found in all drought-treated genotypes and only in a few cases did it remain below level 1, but it was not significant. Two samples showed a significant increase of up to 1.5-fold in the first group of cultivars (*b1*), while most samples from the second group (*b2*) showed a higher mRNA level, at around 2 relative units (Figure 3).

### 2.4. Microarray 40K SNP Analysis of Parents and Selected Breeding Lines from Interspecies Hybrid Cross No. 18-6 between Spelt and Bread Wheat

**Figure 4:**
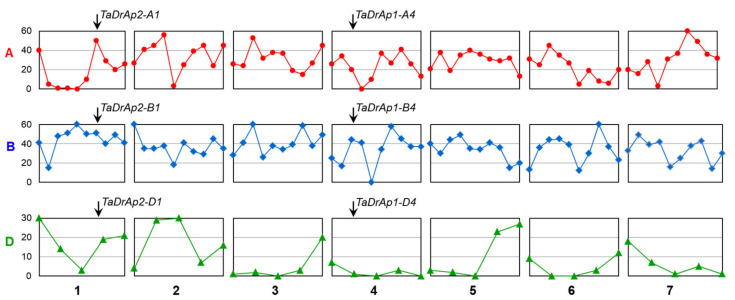
The distribution of 4806 effective and mapped Single nucleotude polymorphisms (SNPs) extracted from the 40K microarray analysis, based on the annotated bread wheat genome cv. Chinese Spring. The SNPs were distributed along each of the seven groups of homeologous chromosomes, as indicated in boxes. Three genomes (A, B, and D) are shown in different colors and the numbers of identified SNPs are shown on the *y*-axis. For A and B genomes, SNPs were bulked into 10 bins of equal genetic distance in each chromosome, while only five bins were used for bulking SNPs for the D genome. The bins are shown as dots, squares, and triangles for A, B, and D, respectively. Positions of the two targeted genes, *TaDrAp1* and *TaDrAp2*, are indicated on chromosome bins by arrows.

The analysis of a 40K SNP microchip array was conducted with DNA from both the parents and nine selected breeding lines. A total of 20,749 SNPs were called across the 40K array, including 12,580 polymorphic SNPs. After filtration for quality, only 4806 SNPs with known locations on the bread wheat genetic map, cv. Chinese Spring, were identified for further analyses, comprising only scores which were identical for two biological replicates of each parental genotype. The distribution of the 4806 mapped SNPs among the three genomes and along the chromosomes in homeologous groups was quite variable (Figure 4). The number of SNPs used for the wheat A genome (1909) was comparable with those for the B genome (2576), while only 321 SNPs were identified and mapped in the D genome (Figure 4).

The genetic polymorphism analysis showed an overview of total numbers and distribution of SNPs identified between spelt and bread wheat genotypes, which were used as parents in the study. Two targeted genes, *TaDrAp1* and *TaDrAp2*, were localized on the genetic map with positions indicated in sets of homeologous chromosomes 4 and 1, respectively (Figure 4).

From the two targeted genes, *TaDrAp1* and *TaDrAp2*, no SNPs were used for the analysis and their chromosome bins were determined using the closest and nearby scaffolds on the genetic map. Importantly, strong consensuses were found in genotyping all clusters of SNP markers in one or two scaffolds surrounding the positions of the targeted genes. Therefore, the identical SNP scores of the surrounding genetic regions indicated an absence of recombination near *TaDrAp1* and *TaDrAp2*, making their projected genotyping easy (Table 2). The only exception was found in the *TaDrAp1-A4* gene, where only monomorphic SNP markers were present in a large surrounding genetic region.

The 4806 SNP markers used were selected for identical scores in the two biological replicates for each parent, but four from nine of the breeding lines represented admixtures of two genotypes, which were not removed and are shown in Table 2. The *TaRht-B1* gene was included in the SNP microarray analysis because parents and breeding lines differed in plant height. Phenotypic evaluation for grain yield and plant height are included in Table 2 for convenience, while the nine breeding lines are arranged in three groups with high, moderate, or low yields of hulled seeds (Table 2).

### 2.5. Genetic Polymorphism and SNP Identification in the Segregating Population No. 18-6 from an Interspecies Cross between Spelt and Bread Wheat

Two parents of the complex interspecies hybrid cross No. 18-6 and eight bread wheat cultivars described in Section 2.3 were sequenced for the targeted genes, *TaDrAp1* and *TaDrAp2* (six homeologs in total for each line). No differences were found in the ORF regions of either of the genes from all the wheat lines, indicating the very conserved structure of these genes among cultivars of bread wheat and the spelt wheat parental form.

**Figure 5:**
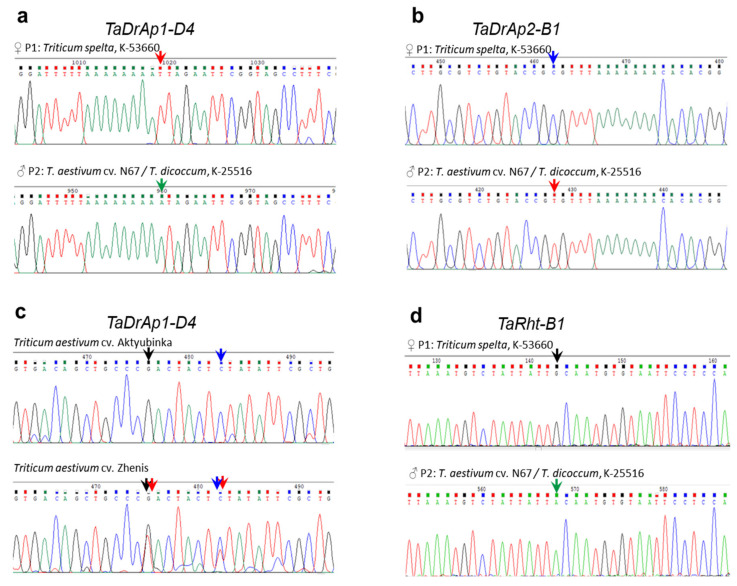
Example of the identified SNPs in parental forms of hybrid cross No. 18-6 in the promotor region of homeologs *TaDrAp1-D4* (**a**) and *TaDrAp2-B1* (**b**). SNP differences among two cultivars of bread wheat in the promotor of *TaDrAp1-D4* (**c**) and in the first intron of *TaRht-B1* gene determining plant height (**d**). SNPs are shown by arrows in corresponding colors.

Variations were seen during sequencing of the promotor regions of the genes. Our primers targeting a ~1 kb fragment of the promotors of *TaDrAp1-A4* were developed based on the sequence of cv. Chinese Spring, and they failed to amplify PCR products in all of the genotypes. In contrast, successful amplification of PCR products from promotors of two other homeologs, *TaDrAp1-B4* and *TaDrAp1-D4*, resulted in two SNPs, one in each homeolog, between parental forms of the segregating population No. 18-6, both originating from the paternal form (bread wheat). For the *TaDrAp2* gene, four SNPs and one small insertion/deletion (InDel) were found in the promotor region of *TaDrAp2-B1*, originating from the maternal genotype (spelt wheat). There was only one SNP in the promotor of *TaDrAp2-D1*, also originating from the maternal genotype (spelt wheat). Examples of the SNPs identified in *TaDrAp1-D4* and *TaDrAp2-B1* of the parental forms of hybrid No. 18-6 are presented in images a and b of Figure 5, respectively.

Eight bread wheat cultivars were compared with respect to genetic polymorphisms in the two targeted genes, *TaDrAp1* and *TaDrAp2*. Rare SNP alleles were present either as an admixture of two alleles, or as heterozygotes, showing doubled peaks in a single nucleotide position in the sequences. Examples of such homozygote and heterozygote alleles in *TaDrAp1-D4* are shown for cultivars Aktyubinka and Zhenis (Figure 5c). Only a few of the wheat cultivars examined showed mixed SNP results.

The sequence analysis for the *TaRht-B1* gene of both parents of the hybrid population No. 18-6 revealed the presence of two clear SNPs in the coding (CDS) and untranslated (3′-UTR) regions of the gene. Both SNPs originated from the paternal parent of the hybrid population, representing novel non-annotated alleles of the gene, and an example of one SNP in the *TaRht-B1* gene in both parental forms of the hybrid is presented in Figure 5d. All cultivars of bread wheat examined had a tall plant stature, with an identical wild-type allele of the *TaRht-B1a* gene.

### 2.6. Amplifluor-Like SNP Genotyping of TaDrAp1 and TaDrAp2 Genes in Segregating Population No. 18-6

Amplifluor-like SNP markers were developed based on SNPs identified during sequencing of the promotor regions of the *TaDrAp1/2* genes, as described in Section 2.5. Details of the sequences with the identified SNPs, results of BLAST analysis, and SNP primers developed are presented in Supplementary Material File S3.

**Figure 6:**
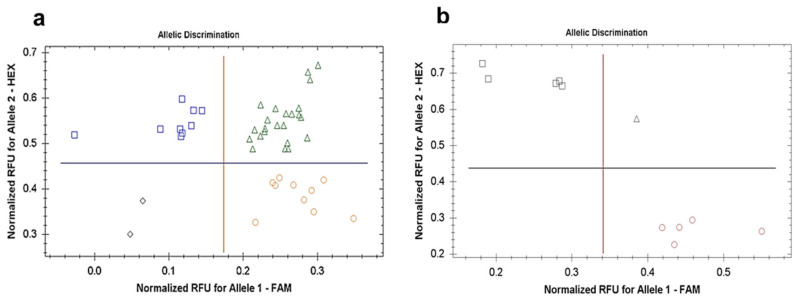
Allele discrimination plots for Amplifluor-like SNP genotyping of *TaDrAp2-B1* in parents and 42 F_3_ progenies of hybrid population No. 18-6 (**a**) and in nine F_5_-selected breeding lines from the same population (**b**). Normalized relative fluorescence units (RFU) for FAM and HEX fluorophores are indicated on the *x*- and *y*-axes. Red circles represent homozygote genotypes with allelle 1 (‘*aa*’), blue squares show homozygote genotypes with allelle 2 (‘*bb*’), heterozygotes for both alleles ‘*ab*’ are indicated by green triangles, and black rhomboids indicate unidentified genotypes. Sterile water, instead of DNA, was used as the no template control (NTC). Fluorescence was determined, scored, and shown automatically on the final time-point of amplification by BioRad software provided with the qPCR instrument. However, data were also checked manually. Genotyping was performed with a single biological replicate (each single plant from F_3_ progeny) (**a**) and with three biological replicates (three plants from each F_5_ breeding line) (**b**), repeated twice over different days (two technical replicates).

A clear discrimination was apparent between the alleles of the identified SNPs among four homeologs of *TaDrAp1/2* genes in the genomes B and D (*TaDrAp1-B4/-D4* and *TaDrAp2-B1/-D1)*, in all the examined wheat cultivars, parents, progenies, and breeding lines of the segregating population No. 18-6. An example of Amplifluor-like SNP genotyping for *TaDrAp2-B1* is shown for the parents and 42 F_3_ progenies, and nine F_5_-selected breeding lines (Figure 6a,b). The genotyping results from the Amplifluor-like SNP analysis were very similar to those presented in Table 2 via the 40K SNP microarray. An exception was related to an unscored *TaDrAp2-A1*, where no SNP in the sequences was found between parents and, therefore, we were unable to fully complete the genotyping using the Amplifluor-like SNP approach.

## 3. Discussion

### 3.1. DrAp1/2 Genes Encode Down-Regulator Associated Proteins in Plants

Transcription regulation is a very important process in plant growth, development, and responses to various stresses. TFs, such as nuclear factor Y, are particularly important in this regulation, where each of the three classes (A, B, and C) play a specific role [60,61,62]. Down-regulator, Dr1, and down-regulator associated protein DrAp1, represent very specific and distinct clades close to NF-YB and NF-YC, respectively. They work together as cofactors of negative regulator 2 (NC2) or partners joining in a single enzyme complex and inhibiting transcription of targeted genes [2,12,13]. However, in plants, the roles of Dr1 and DrAp1 partnerships differ from non-plants, and it was proposed that DrAp1 seems to be a repressor, while Dr1 is a co-repressor based on the study of rice plants [25].

Despite some similarity between amino acid sequences of down-regulator Dr1 and NF-YB, and down-regulator associated proteins DrAp1/DrAp2 and NF-YC, both Dr1 and DrAp1/2 have unique N-terminal sequences that clearly distinguish them from all NF-Y proteins. Additionally, binding of TATA boxes in promotor regions is the very specific function of Dr1/DrAp partner cofactors and this is not a feature of any of the NF-Y proteins. Therefore, Dr1 and DrAp were re-classified as homologs of negative cofactors NC2β and NC2α, respectively, according to the original classification [15]. However, both Dr1 and DrAp represent independent clades close to NF-YB and NF-YC groups, with differing degrees of similarity shown depending on the species.

Both Dr1 and DrAp1 proteins have a specific dual structure. On the one hand, Dr1 and DrAp1 have amino acid similarity to NF-YB and NF-YC classes of TF, respectively, but on the other hand, unique fragments are present in the N-terminal region of Dr1 and DrAp1, which determine their ability to target the TATA box of downregulated genes [11]. This results in uncertainty regarding whether to include them as members of the NF-YB and NF-YC classes. For example, it was stated for *Arabidopsis thaliana* that AtDr1 = AtNF-YB12 and AtDr2 = AtNF-YB13 [15], and AtDrAp1 = AtNF-YC11 [19], as well as for *Brachypodium distachyon* that BdDr1 = BdNF-YB16, BdDrAp1 = BdNF-YC4, and BdDrAp2 = BdNF-YC11 [26]. However, protein sequence data from our bioinformatic analysis showed distinct and isolated positions of both TaDr1 and TaDrAp1/2 homeologs among the TaNF-YB and TaNF-YC groups in wheat (Figure 2a) and similar proteins in rice, while the strong conservatism of Dr1 and DrAp1 proteins was fixed over a long period of evolution, as confirmed in Figure 2b. Therefore, it is reasonable and scientifically sound, to support the re-classification of Dr1 and DrAp1 proteins based on the original description of NC2β and NC2α, respectively [15], as independent clades very close to, but not members of the NF-YB and NF-YC classes, respectively. Mistaken identification of the rice *OsDrAp2* gene (Os05g41450) as ‘HAP2-LIKE 1′ (like to OsNF-YA group) [21] must be corrected to reflect its similarity to the OsNF-YC group.

### 3.2. TaDrAp1 and TaDrAp2 Genes in Wheat

In wheat, the *TaDr1* gene has always been identified as an isolated clade, and as such, has no name as a TaNF-YB member. However, corrections must be made to clarify *TaDr1A* and *TaDr1B* as homeologs of the same single gene with correct ID based on chromosome localization as follows: *TaDr1A* (AAL73486 = AF464903), *TaDr1D* (BT009234 = TC416575) [15,16], and *TaDr1B* (BC000036325) [17], as shown in Figure 2a. Two genes, *TaDrAp1* and *TaDrAp2*, were identified and described as *TaNF-YC6* (TC233433) and *TaNF-YC8* (TC241235), respectively [16], and references to sequences of their six homeologs were indicated [22]. However, the updated version of the genome sequences in the International wheat genome sequencing consortium (IWGSC) assembly does not support annotated sequences of *TaDrAp1* and *TaDrAp2* homeologs and, therefore, we repeated BLAST analyses of the six homeologs (Table 1) and identified the full genome/CDS/protein sequences (Supplementary Material File S1). Strong conservation of both genes, *TaDrAp1* and *TaDrAp2*, and their encoded polypeptide structures (Figure 2) reflected the strong stability of *DrAp* genes during evolution. Exceptions were identified only in homeologs of the B genome in both *TaDrAp1-B4* and *TaDrAp2-B1* genes, where a large insertion was determined in intron 3, doubling the length of the ORF in *TaDrAp1-B4*, though with a shortened intron 2 detected in *TaDrAp2-B1* (Table 1).

The results presented in Figure 2b indicated that divergence of *DrAp* genes in plants was initiated at the origin of monocot and dicot plant species. All monocot species examined were conserved in distinct clades for both genes, *DrAp1* and *DrAp2* (Clades A1 and A2, respectively), while the single *DrAp1* gene in all dicot plants clustered together in one isolated clade A3, which was distinct, at the same time, from human and *Drosophila* clade A4 (Figure 2b). Based on these results, we hypothesize that ancient plant progenitors most likely had doubled *DrAp* genes, but after the divergence of monocot and dicot ancestors at the very early stage of evolution, dicot plant species possibly lost one *DrAp* gene, whereas the progenitor of monocot plant species retained the duplication of the gene.

### 3.3. Expression Analysis of Dr1 and DrAp1 Genes in Monocot Species in Comparison to Bread Wheat during Plant Ontogenesis, Development, and under Drought

During the development of rice plants, the *OsDr1* gene (AF464902 = Os08g29500) is expressed at a low level in leaves, but is more highly expressed in root, stem, and suspension cells [25]. A greater amount of mRNA transcripts from two homeologs of the *TaDr* gene (AF464903 and BT009234) was shown in wheat spikes and developing seeds [16], and a similar expression level of *BdDr1* (*BdNF-YB16* = Bradi3g34930) was recorded in all tissues of *B. distachyon* [26].

Different results have been reported for repressor-partner genes *DrAp1/2*. In rice, the expression profile of *OsDrAp1* (AF464904 = Os11g34200) was very similar to *OsDr1* [25]. In contrast, for *OsDrAp2* (*OsHAPL1 =* Os05g41450), the highest level of mRNA transcript was observed in leaves, while the lowest expression was in root and stem [21]. In *B. distachyon*, expression of *BdDrAp1* (*BdNF-YC4* = Bradi2g21290) and *BdDrAp2* (*BdNF-YC11* = Bradi4g16840) were the lowest in the inflorescence and highest in root and shoot, respectively [26]. In wheat, both genes, *TaDrAp1* (*TaNF-YC6* = TC233433) and *TaDrAp2 (TaNF-YC8* = TC241235), were highly expressed in leaves [16].

Our results with bread wheat indicated a relatively high level of *TaDrAp1* gene expression in leaves of eight cultivars, for example, at the vegetative stage, within a range of 2.2–4.6-fold units relative to the reference genes (Figure 3a). These values were close to the published data for the *TaDrAp1* (TaNF-YC6) gene, with relative mRNA expression level about 8 units in leaves of three-week-old wheat plants cv. Babax [16]. Further, our study on older plants indicated that, at tillering and especially at the start of flowering time, the expression of *TaDrAp1* increased up to 3.1–5.2-fold units in the first group (*a1*) of high-yielding cultivars, but decreased to 1.1–2.1-fold units in the second group (*a2*) comprised of cultivars with lower yield performance. Therefore, there was a common trend for dynamic changes in *TaDrAp1* expression, upregulated or downregulated in wheat from the *a1* and *a2* groups, respectively, during plant growth. However, it would be premature and mere speculation to draw any conclusion in this regard.

In contrast, the level of *TaDrAp2* mRNA production was low at all three time-points in all eight of the wheat accessions examined, and varied in a range of 0.3–0.8-fold, compared with the reference genes (Figure 3a). This observation contradicts data published for *TaDrAp2* (TaNF-YC8), where expression of the gene in leaves of young plants of cv. Babax was about 7 units in relative mRNA level. The reason for such a big difference between our results and those published remains unclear and requires further study.

Under drought, both genes, *TaDrAp1* and *TaDrAp2*, changed their expression but in opposite directions. The level of mRNA of *TaDrAp1* dropped significantly to about 1 relative unit, with some fluctuation in the first group of cultivars (*a1*), while the level mostly ranged higher, between 1 and 2 units, in the second group of cultivars (*a2*). Raised expression was recorded for the *TaDrAp2* gene in almost all wheat genotypes examined, and over the time-points of ontogenesis after drought treatment. However, wheat cultivars from the first (*b1*) and the second groups (*b2*) could be characterized by the same levels of expression: about 1 relative unit, and ranging between units 1 and 2, respectively. Therefore, we can conclude that, under stress, mRNA of both *TaDrAp1* and *TaDrAp2* were regulated to similar levels in most of the accessions despite very high and very low expression of these genes, respectively, in non-stressed control conditions. Additionally, wheat from the second group *b2* had, in general, higher expression of both genes in drought conditions compared with cultivars from the first group *b1* (Figure 3b).

### 3.4. Genetic Polymorphism in TaDrAp1, TaDrAp2, and TaRht-B1 Genes in Spelt and Bread Wheat using 40K SNP Microarray and Amplifluor-Like SNP Genotyping

Microarray is convenient for the estimation of a wide distribution of SNPs over the whole genome, allowing genome-wide association studies (GWAS) in widespread crops such as bread wheat. Scant attention has been paid to the molecular study of spelt wheat, but GWAS with 15K SNP microarrays were applied to analyze molecular diversity among 123 and 293 spelt wheat accessions grown in Europe. These reports offer novel tools for the estimation of genetic diversity and development of marker-assisted selection in *T. spelta* and hybrids between spelt and bread wheat [39,40].

Each breeding line developed from F_2_ progenies has a unique combination of genes due to their specific patterns of recombination. However, relatively large fragments of the chromosome with flanking recombination events may be transferred as unchanged clusters or gene blocks from parents to offspring. Haplotypes may inherit genetic fragments along with several nearby SNPs between two flanking recombination events [36,37]. This appears to have occurred for the genes in the current study, as shown by the 40K SNP assay, where non-recombinant genetic fragments have been transferred without changes, representing either maternal or paternal haplotypes. Therefore, identical allele combinations of SNP markers in the genetic regions surrounding the genes of interest can support an accurate prediction of the genotype of selected breeding lines from the hybrid population No. 18-6 between spelt and bread wheat (Table 2). The presence of a significant number of heterozygotes and admixtures of genotypes in the breeding lines indicated the need for one more round of single seed descent and propagation to produce more homozygous breeding lines. The absence of differences in sequences of ORF regions in the *TaDrAp1* and *TaDrAp2*, genes indicated very strong conservation during the long process of wheat evolution. However, SNPs were found in four homeologs of both genes, *TaDrAp1* and *TaDrAp2*, in the wheat B and D genomes. The genotyping results of parents and breeding lines for the three genes, *TaDrAp1*, *TaDrAp2*, and *TaRht-B1*, with our developed Amplifluor-like SNP markers, confirmed the microarray results for the B and D genomes (Figure 6).

Significant differences were observed from genotyping of *TaDrAp1-B4* and *TaDrAp2-B1* among the three groups of breeding lines from interspecies cross No. 18-6 between spelt and bread wheat (Table 2). The production of hulled seeds in spelt wheat can be improved significantly when alleles *TaDrAp1-B4*, *TaDrAp2-B1*, or both can be introgressed from a drought-tolerant genotype of bread wheat.

### 3.5. Interaction of TaDrAp1 and TaDrAp2 with Genes Controlling Plant Development, Tolerance to Drought, and Yield Components in Spelt and Bread Wheat

Drought is a very complicated trait, where many genes are involved in plant responses. Previously, we reported that the repressor partner gene, *TaDr1* (down-regulator, negative cofactor NC2β) was differentially expressed, and co-expressed with vernalization- and flowering-time-determining genes (*TaVrn1* and *TaFT1*) in bread wheat cultivars showing high and low yield under drought conditions in Kazakhstan [17]. The present results for two partner genes from the same repressor complex, *TaDrAp1* and *TaDrAp2* (down-regulator associated protein, negative cofactor NC2α), were also differentially expressed during transition from vegetative to reproductive stage and flowering in the same bread wheat cultivars. However, under drought, *TaDrAp1* and *TaDrAp2* showed opposite directional changes in expression being strongly downregulated and upregulated, respectively (Figure 3). This agrees with published data for orthologous genes *OsDrAp1* and *OsDrAp2* in rice with single homeologs in the diploid genome, where both rice genes were shown to be strongly associated with plant development and time to flowering [19,21]. To our knowledge, there is no published information about the involvement of *DrAp* genes in drought tolerance in any plant species, but the partner-gene, *TaDr1*, including all three homeologs, were reported as strongly upregulated in wheat plants in response to drought [16,17].

In this study, the hybrid population No. 18-6 from the interspecies cross between spelt and bread wheat was analyzed. Successful genotyping using both the 40K SNP array and Amplifluor-like SNP markers, based on confirmed sequences, showed significant co-segregation of two homeologs from the B genome, *TaDrAp1-B4* and *TaDrAp2-B1*. For the first homeolog, *TaDrAp1-B4*, three selected breeding lines with a high yield of hulled seeds from group 1 were homozygous for ‘*bb*’ genotypes, while heterozygotes ‘*ab*’ genotypes were found in both groups 1 and 3 (Table 2). Results for the second homeolog, *TaDrAp2-B1*, showed even stronger allele discrimination, where breeding lines with high and low yield (groups 1 and 3) were genotyped as ‘*bb*’ and ‘*aa*’, respectively, while an admixture and different alleles were present in breeding lines with moderate grain yield (group 2).

The identified ‘*b*’ alleles in both *TaDrAp1-B4* and *TaDrAp2-B1*, which strongly co-segregated with high grain yield, originated from the paternal genotype No. 18-5 ♂(*T. aestivum* cv. Novosibirskaya 67 (N67) / *T. dicoccum*, K-25516). This finding was surprising initially because, as described in Section 4.2 below, plants of paternal parent No. 18-5 had lower vigor and slower start to growth, due perhaps to the presence of introgressed chromosomes from the tetraploid *T. dicoccum*. However, their growth improved at later stages, as reflected in the yield at harvest. Regardless of these early deficiencies, plants of the paternal parent No. 18-5 were extremely drought tolerant, showing none of the typical symptoms of slower growth and leaf senescence under drought, particularly in the early-middle stages of plant development. We also checked that chromosome segments containing both homeologs, *TaDrAp1-B4* and *TaDrAp2-B1*, did not originate from bread wheat cv. N67 (data not shown).

We conclude that selected breeding lines from hybrid population No. 18-6, particularly from group 1, represent advanced and improved spelt wheat breeding lines with significantly higher grain yield and better drought tolerance. These phenotypic responses strongly co-segregated with particular genotypes of two homeologs, *TaDrAp1-B4* and *TaDrAp2-B1*, and their haplotypes had been introgressed from a wild tetraploid progenitor, *T. dicoccum*. Unique genotypes of both genes, *TaDrAp1* and *TaDrAp2*, as shown in this study, were responsive to drought stress and were associated with a higher yield of hulled seeds in hybrid spelt wheat.

## 4. Materials and Methods

### 4.1. Bioinformatic Analysis

Bioinformatics and systems biology methods were applied in this study to identify the full-length nucleotide sequence, CDS, and structures of the genes, *TaDrAp1* and *TaDrAp2*, and their corresponding polypeptide sequences, using the Gramene database (http://www.gramene.org) [63] with IWGSC data related to the reference sequence of bread wheat, IWGSC RefSeq v1.0. All sequences from other plant species were recovered via both BLASTN and BLASP from the GenomeNet Database Resources, Kyoto University, Japan (https://www.genome.jp/tools/blast) and NCBI (https://www.ncbi.nlm.nih.gov) [64]. Sequences, accession ID, and gene nomenclatures were checked against specialized databases for rice (http://rice.plantbiology.msu.edu) [65], *Brachypodium* (https://brachypodium.org) [66], and *Arabidopsis thaliana* (https://www.arabidopsis.org) [67]. For all other species, KEGG database (https://www.genome.jp/kegg) was used. Multiple sequence alignments of nucleotide sequences were conducted in CLUSTALW using the GenomeNet database (https://www.genome.jp/tools-bin/clustalw) and CLC Main Workbench software (https://www.qiagenbioinformatics.com/products/clc-main-workbench). The molecular dendrogram of DrAp1, DrAp2, Dr1, and NF-Y polypeptides from wheat and various plant species, and reference genomes of *Drosophila* and humans were constructed using the SplitsTree4 program (http://www.splitstree.org) [68].

### 4.2. Plant Material

**Figure 7:**
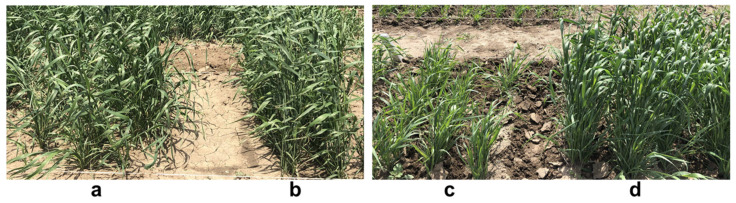
Images of plants from parental and breeding lines from the segregating population No. 18-6 at tillering stage grown in the research field trial in northern Kazakhstan in 2020, as follows. (**a**) breeding line No. 18-6-37; (**b**) ♀ 18-4, spelt wheat; (**c**) ♂ 18-5, bread wheat; (**d**) breeding line No. 18-6-174.

The complex, interspecies hybrid cross No. 18-6, between spelt and bread wheat [♀ *Triticum spelta*, K-53660 × ♂ *T. aestivum* cv. Novosibirskaya 67 (N67) / *T. dicoccum* (Schrank) Schuebl, K-25516], was produced by one of the authors, Nikolay P. Goncharov, at the Institute of Cytology and Genetics, Russian Academy of Sciences, Novosibirsk (Russia). Nine breeding lines (generation F_5_) from the hybrid cross were selected after two rounds of single seed descent and propagation, based on their grain yield: with three breeding lines each selected in the categories of high, moderate, and low yield of hulled seeds. Both the parents and the nine breeding lines were grown in a field trial for phenotypic evaluation. Images of plants from both parents and the breeding lines were randomly selected and are presented in Figure 7. Plants of spelt wheat (the maternal parent) showed very good vigor and early growth, but the yield of hulled seeds was moderate. The growth of plants from the paternal parent of bread wheat with the introgressed tetraploid *T. dicoccum*, was slower, but seed yield was similar in both parents at harvest.

Additionally, eight local spring bread wheat cultivars, representing two groups with contrasting yields, were selected from local varieties tested in field trials, based on their grain yields under the dry conditions in Northern and Central Kazakhstan, as described earlier [17,46]. The first group of four cultivars with high-yield capacity included Aktyubinka, Albidum 188, Altayskaya 110, and Saratovskaya 60, while the second group of four other cultivars with low-yield capacity included Vera, Volgouralskaya, Yugo-Vostochnaya, and Zhenis. These wheat materials were the same as described and published earlier [48]. Seeds of eight cultivars were obtained from S. Seifullin Kazakh AroTechnical University, Nur-Sultan (Kazakhstan).

### 4.3. Drought Experiments in the Field Trial

A small outdoor trial was conducted in the research field of S. Seifullin Kazakh AgroTechnical University, Nur-Sultan, in Northern Kazakhstan in 2020, in the same conditions as described earlier [48]. In brief, Kastanozem soils with 2.5–2.7% of humus provided sufficient conditions for growing crops. The previous season’s crop in this field was a legume (chickpea) and, therefore, no additional mineral fertilizers were applied. Early drought is typical in Northern Kazakhstan [69], but in 2020, the early-spring season was especially dry, with just 3 mm of precipitation in May (31 mm in average). Soil moisture was measured using the direct gravimetric or oven-dry method [70] and, at that moment, ranged from 80% at a 60 cm depth and 30% in the 10 cm top-soil layer. In such an environment, seedlings and young plants survived strong drought stress in the early stages of their development. This was followed by a doubling of expected rainfall in June, with 86 mm compared to the 41 mm average observed over many years in this region, which helped plants to recover. The average temperature in May was higher by 4.2 °C (16.7 °C compared with the average, 12.5 °C), but was then more similar to average in the following months of that year.

Three-row plots were sown, 1 m in length with 5 cm between plants in rows and 20 cm between rows, with four randomized block replicates. According to current agronomic practice in northern Kazakhstan, the standard density of spring bread wheat is 250–300 plants/m^2^, where, in general, the distance between plants is 2 cm in a row. In our experiments, the density ranged between 100 and 120 plants/m^2^, which is the typical density for research fields. Grain yield was measured at harvest for all plants in each plot and re-calculated in ‘g/plot’ with statistical treatment as described below.

### 4.4. DNA Extraction and 40K SNP Microarray

Wheat plants were grown in control (non-stressed) conditions in pots with soil as described above. Individual one-month-old wheat plants were selected from parents and breeding lines from the hybrid population No. 18-6. Two plants (two biological replicates) in each genotype and one leaf was collected separately (no bulking). Leaf samples were frozen in liquid nitrogen and ground in 10-mL tubes with two 9-mm stainless ball bearings using a vortex mixer. DNA was extracted from leaf samples with phenol-chloroform as described in our earlier papers [46,48]. DNA (1 µL) was checked on a 0.8% agarose gel to assess quality, and concentration was measured by a NanoDrop spectrophotometer (ThermoFisher, Carlsbad, CA, USA).

DNA samples were submitted to AgriBio, Agriculture Victoria Research Division, Melbourne (Australia) for the microarray 40K SNP analysis. Genotypes were called for SNPs targeted by the SNP chip assay and imputed to high density using globally diverse in-house exome sequence data. All SNPs targeted by the SNP chip were provided in an MS Excel file, where SNP positions were indicated using the IWGSC cv. Chinese Spring genome assembly.

### 4.5. DNA Extraction and Amplifluor-Like SNP Genotyping

For SNP genotyping with Amplifluor-like SNP markers, five uniform, one-month-old, individual wheat plants were selected for each accession and five leaves were collected and bulked for leaf samples. Leaves were sampled and DNA was extracted from the bulked leaves as described above in Section 4.4.

The Amplifluor-like SNP analysis was carried out using a CFX96 real-time PCR detection system (BioRad, Irvine, CA, USA) using DNA samples as described previously [47,71] with the following adjustment for wheat genotyping. Each reaction with a total volume of 10 µL contained: 3 µL of template DNA adjusted to 20 ng/µL, 1 µL of the mix containing two fluorescently labeled universal probes (0.125 μM each), 1 µL of the allele-specific primer mix (0.075 μM of each of two forward primers and 0.39 μM of the common reverse primer), and 2 µL of 5 × Go-Taq Master mix (Promega, San Luis, CA, USA) with the following final concentration of components: 1.75 mM MgCl_2_, 0.2 mM of dNTP, and 0.05 units of Go-Taq polymerase (Promega, San Luis, CA, USA). Assays were performed in 96-well microplates. The annotated SNP sites were used to design allele-specific primers. The design and sequences of the primers, and sizes of amplicons generated are presented in Supplementary Material File S4.

PCR was conducted using a program adjusted from those published earlier [47,72]: initial denaturation, 95 °C, 2 min; and 20 ‘doubled’ cycles of 95 °C for 10 s, 60 °C for 10 s, 72 °C for 20 s, 95 °C for 10 s, 55 °C for 20 s, and 72 °C for 50 s, with recording of allele-specific fluorescence after each cycle. Genotyping by SNP calling was determined automatically by the instrument software, but each SNP result was also checked manually using amplification curves and final allele discrimination. Experiments were repeated twice over different days, where two technical replicates confirmed the confidence of SNP calls.

### 4.6. RNA Extraction, cDNA Synthesis, and qPCR Analysis

Wheat plants were grown in the ‘Phytotron’-controlled climate chamber at S. Seifullin Kazakh AgroTechnical University, Nur-Sultan (Kazakhstan), as described earlier [48]. Briefly, for mild drought stress with one-month-old plants, watering was withdrawn in one of two soil-filled containers for 12 days until wilted leaves were observed. Control plants in similar containers were watered continuously. Five individual plants were used for each cultivar in drought-affected and well-watered containers. All leaves were collected from each plant in plastic tubes as separate biological replicates, frozen immediately in liquid nitrogen, and kept at −80 °C until RNA extraction. Three samples were used for RNA extraction from each cultivar and treatment, while two additional samples were used as replacements in case of failed extraction or poor RNA quality.

Frozen leaf samples were ground as described above for DNA extraction. TRIzol-like reagent was used for RNA extraction following our earlier protocol [73]. RNA concentration was measured using the NanoDrop spectrophotometer (ThermoFisher, Carlsbad, CA, USA), while the quality and integrity of intact RNA was confirmed by running 1 µg of RNA in a 1.5% denaturing agarose gel. Two micrograms of RNA was used in each sample for cDNA synthesis as described previously [74], including DNase treatment and the use of a ProtoScript-II reverse transcriptase kit (NEB Biolab, Hitchin, UK), following the manufacturer’s instructions. The quality of all cDNA samples was confirmed by PCR with known primers resulting in bands of the expected size on agarose gels.

Samples of cDNA diluted with water (1:10) were used for qPCR analyses using a real-time qPCR system, Model CFX96 (BioRad, Irvine, CA, USA), using qPCR protocols as described earlier [46]. The total volume of 10 μL qPCR reactions included 5 μL of 2×KAPA SYBR FAST (KAPA Biosystems, Wilmington, MA, USA), 4 μL of diluted cDNA, and 1 μL of two gene-specific primers (3 μM of each primer) (Supplementary Material File S5). The volume of 10 μL qPCR reactions was verified over our long-term experience in working with gene expression. Despite the manufacturer’s recommended volume of 20 μL, stability, accuracy, and reproducibility were consistently very high in our experiments using a 10 µL volume. Thermal cycling conditions included a brief initial melt at 95 °C for 3 min, followed by 40 cycles of 95 °C (5 s) and 60 °C (20 s), and finished with a melt curve from 60 °C to 95 °C increasing by 0.5 °C increments every 5 s. The efficiencies of all qPCR primers were calculated based on the slope of the corresponding calibration line, E = 10^slope^, and all were within the suitable range, 1.8–2.0 [75]. Specificities of target and reference genes were confirmed by single distinct peaks on a melting curve and a single band of the expected size in 2% agarose gel electrophoresis. Expression data for the target genes were calculated relative to the average expression of the two reference genes: Ta22845, ATP-dependent 26S proteasome and Ta54825, actin [76]. At least three biological and two technical replicates were used in each qPCR experiment.

The relative standard curve method was used in the study based on the ABI Guide to performing relative quantitation of gene expression using real-time quantitative PCR (http://www3.appliedbiosystems.com/cms/groups/mcb_support/documents/generaldocuments/cms_042380.pdf), where serial dilutions were applied for each target and reference gene individually [77]. Threshold cycle values were determined based on linear calibration of template cDNA dilution factor and cycle quantification value (Cq).

### 4.7. Statistical Treatment

IBM SPSS statistical software was used to calculate and analyze means and standard errors using ANOVA, and to estimate the probabilities for significance using Student’s *t*-test. Differences between genotypes in three groups were analyzed using the non-parametric Mann–Whitney U-test with a low number of samples (nine breeding lines, three lines in each of three groups) via IBM SPSS, Statistics Desktop 25.0.0.0.

## Figures and Tables

**Table 1:** Characteristics of the identified genes, *TaDrAp1* and *TaDrAp2*, encoding two down-regulator associated proteins, from the public database Gramene with sequence of cv. Chinese Spring.

*TaDrAp* Genes	Gene ID (TraesCS)	ORF (bp)	Chromosome Location (Strand)	CDS (bp)	Protein (aa)
***TaDrAp1***					
*TaDrAp1-A4*	4A02G151500	5188	Chr4A: 306,315,444–306,320,631 (−)	768	255
*TaDrAp1-B4*	4B02G161200	10,031	Chr4B: 316,654,368–316,664,398 (−)	765	254
*TaDrAp1-D4*	4D02G158200	4338	Chr4D: 220,464,822–220,469,159 (−)	768	255
***TaDrAp2***					
*TaDrAp2-A1*	1A02G310700	5490	Chr1A: 501,702,108–501,707,597 (+)	852	283
*TaDrAp2-B1*	1B02G322000	5172	Chr1B: 546,528,780–546,533,951 (+)	852	283
*TaDrAp2-D1*	1D02G310200	5492	Chr1D: 406,545,964–406,551,455 (+)	855	284

Note: Open reading frames (ORF); Coding sequences (CDS).

**Table 2:** Phenotypic evaluation for grain yield and plant height, and summary of genotyping scores from the 40K SNP microarray analysis, based on the scaffolds, for the parents and nine selected breeding lines from the segregating population of interspecies cross No. 18-6. Breeding lines were arranged in three groups with high, moderate, and low yield of hulled seeds based on significant differences between groups (*p* < 0.05) at harvest in Northern Kazakhstan in 2020. Significant differences for genotyping of breeding lines among the three groups (*p* < 0.05) are indicated by asterisks (*) using Mann–Whitney non-parametric U-test with *n* = 2 in each genotype. Homozygote genotypes are indicated as ‘*aa*’ in red and ‘*bb*’ in blue, according to maternal and paternal genotypes, respectively, while heterozygotes ‘*ab*’ are indicated in green. Admixed lines are indicated as a mixture of two genotypes. N.P., non-polymorphic SNP markers only.

ID	Grain Yield (g/plot)	Plant Height (cm)	SNP Genotyping						
			*TaDrAp1-A4*	TaDrAp1-B4 *	*TaDrAp1-D4*	*TaDrAp2-A1*	TaDrAp2-B1 *	*TaDrAp2-D1*	*TaRht-B1*
♀ 18-4, spelt	108	71	N.P.	***aa***	***aa***	***aa***	***aa***	***aa***	* **aa** *
♂ 18-5, bread	99	57	N.P.	* **bb** *	* **bb** *	* **bb** *	* **bb** *	* **bb** *	* **bb** *
**Breeding lines, group 1**									
18-6-174	319	94	-	*ab* + *bb*	***aa***	*ab* + *bb*	* **bb** *	***aa*** + *ab*	* **aa** *
18-6-82	281	87	-	* **bb** *	* **bb** *	* **bb** *	* **bb** *	***aa***	* **aa** *
18-6-37	240	67	-	*ab* + *bb*	*ab* + *bb*	*ab* + *bb*	* **bb** *	* **bb** *	*ab* + *bb*
**Breeding lines, group 2**									
18-6-119	174	79	-	***aa***	* **bb** *	***aa***	*ab* + *bb*	* **bb** *	* **aa** *
18-6-93	166	66	-	***aa***	***aa*** + *ab*	***aa***	* **bb** *	***aa***	*ab*
18-6-62	131	68	-	***aa***	* **ab** *	***aa***	***aa***	* **bb** *	* **bb** *
**Breeding lines, group 3**									
18-6-126	115	78	-	* **ab** *	* **bb** *	***aa***	***aa***	* **bb** *	***aa***
18-6-105	94	58	-	***aa***	***aa***	* **bb** *	***aa***	***aa***	* **bb** *
18-6-61	80	57	-	***aa***	* **ab** *	* **ab** *	***aa***	***aa***	* **bb** *
Scaffold, SNP microarray			51014	124156, 109338	133452	10127, 48187	88266, 93878	20922, 92741	30139, 18668

## Supplementary Materials

Supplementary materials can be found at https://www.mdpi.com/1422-0067/21/21/8296/s1.

## Abbreviations

CBF	CCAAT-binding factor
CDS	Coding sequence
Dr1	Down-regulator
DrAp1	Down-regulator associated protein
GWAS	Genome-wide association study
IWGSC	International wheat genome sequencing consortium
NC2	Negative cofactor 2
NF-Y	Nuclear factor Y
NIL	Near-isogenic lines
ORF	Open reading frame
RFU	Relative fluorescence units
SNP	Single nucleotide polymorphism
TBP	TATA-box-binding protein
TF	Transcription factor
TFIID	Transcription factor IID
