# Supplementary material for: TaDrAp1 and TaDrAp2, Partner Genes of a Transcription Repressor, Coordinate Plant Development and Drought Tolerance in Spelt and Bread Wheat
Source: Int J Mol Sci. 2020 Nov 5;21(21):8296. doi: 10.3390/ijms21218296 (PMC7663959; doi:10.3390/ijms21218296)
Supplement: Supplementary file 1 [file ijms-21-08296-s001.pdf]

## Supplementary file 1

### Sequences of genomic DNA, CDS and proteins

Start-codons are indicated in green, Stop-codons are in red, exons are in yellow.

### ***TaDrAp1-A4*** (TraesCS4A02G151500)

**TraesCS4A02G151500 REVERSE STRAND**
pep chromosome:IWGSC:4A:306315187:306320710:-1 gene:TraesCS4A02G151500
transcript:TraesCS4A02G151500.1 gene_biotype:protein_coding
transcript_biotype:protein_coding description:CCAAT-binding transcription factor C
[Source:UniProtKB/TrEMBL;Acc: A0A0A7LUP0 = https://www.uniprot.org/uniprot/A0A0A7LUP0)

***>TaDrAp1-A4***
ATCTGGTAGTTTGGGGGTACGTAGGTATACATAGGAGGAAGGAGTACGTTGATGGATCAATAGGGG
GCCCACGAGGGTAGAGGGCACGCCCAGGGGGGTAGGCGCGCCTCCCTACCTCGTGGCCTCCTGGTT
GATGTCTTGACGTAGGGTCCAAGTCCTCTGGATCATGTTCGTTCCGAAAATCACGTTTCCGAAGGT
TTCATTCCGTTTGGACTCCGTTTGATATTATTTTTGTGCGAAACTCTGAAATAGGCAAAAAACAAC
AATTTTGGGCTGGGCCTCCGGTTAATAGGTTAGTCCCAAAAATAATATAAAAGTGTATAATAAAGC
CCAATAATATCCAAAAAGAATATAATATAACATGAAACAATAAAAAAATATAAATACGTTGGAGAC
GTATTAGTGATTAGGTACCAAGATGATGGCCATAGAGCAGCTAGGAGCAATAGACAAGAAGGCAAG
AAAGAGGAGCAAAAGAAGCAAGGAAAGGAATTGCTAGCAAGCTACATGGACCAAGCGTCCGACACG
TTCACTTATAGCCAACCACAAAGCCAACAGCGGATGAACGTTACAGCGGCCGGACGTCCGACAAGT
TCCAGCAACCGGCCGTCCGGTCTTCACCAGAAATCCTGTACCACATGTCCGGAAGCAAACTGACGG
AAGTCCGATGCGCACTGGACATCCGAACACTTCCGAGCCACCAGACGTCCGGCAAACACCGGAAAT
CAAGTACCACCTGACCAGAGAAGAAAATGGCGGAAGTCCGGCGCCCAACCGGACGTTCGGTCACTC
GCGAACGACTGGGCGTTCGGTACTGCATGTGCACATCCCCACGGTTTTCAGCCCATGTACCCCTCT
TTCACCCCTAGGCTATATACCCTTCTCCTACATCCATCATAGGGCTGGCAAAGACATAATAGAGAA
TTAGTAGAGCTTTGCCCATACTCATTATTCCTCTTGGAGATCAAGACCTCCATGCGAGAACATCCC
CTAGTGGATGCAAGACCCATATAAGGGAAGATCCATCTAGGGATGCAAGACCCCTATATGGGAAGA
TCCTCCATGGATGCTAGATCTCATCTCCTTTGGATTTGAGAAGAACCTTTACCTTTGTGCTTTCCC
CTTTGACTTGTTCTTGTATATTTGTGGATCCCATAGTGATGTGTGATTTGGGTTTGTTTGAGTCTC
TCTCTCTCTCTCGTGTTCTTGAATGCTCATCCTTTCCCCACCTCCAAGTGCGAAAAGATCTCAA
GTCTAGGGTTTTGCCCTACATCACCGTGCCTCCCCATCCCTTCCTCTCCTCTCCTCTTTCCTTTCT
CGTAGGTCACTGACCTCCTACTCGTGTCAGGTCCCTCTCCTCCTTCCTTCCTCGCAGGTCGTCGAG
CAAGGAATCGAGCTGGGCAAACGCGACGGCCGGTCGGATCAATGCCGAATTGACCGGATCCGACGC
AGGGATCACAACAAGGAGGAGAAAGTCGGAGGAAGGAGCGGTGGCGGGCAGGGGCGGCAACCATGG
TGGCGTTGGTGGCAGGAGTGGCAATGGAGGCTCCGCCGACCACGACGAGGAAAGCGGGATCTGCAG
TGAGCTTCGCGGGGTTGGCTGCGGGGGTCAGAGGAGCTGGCCATGGCGACGCTGTGGCCAGCGGGC
GATCAGAGGCGGCGTTCCGGTTGCGCGCGGGAAAAAGGTAGCGATTGGGTTTCGCGATGCAACCGC
TCTTTTTTTGTGGCAGCCGCCCTACTACTCTATATTCGCTGCACTTGAGGACTTTTTGTTGGCTTC
CCCAAATATTTTTTTTCAAAAATATTAGAATTTTTTAGAGGAAAAAAAAAGAATAAAATAGCCTTT
CATGCTCTAAAAACAACTTGTGCCTGAAAATTATAGGGCTTGCAGTGGGAGACGCGGCAAATATCT
TTTTGGCACGAGTCGGTGTGCCCCGTGCCGTGGTGGTGCAAGTTGGTGGAAAGACAACAAAACAAG
AAATCCAATCGTCAAAGAAGAACGAGAAAAGAAAATACACAAAAGATAGACCACGATTGCCGGCAT
CGAGCCCGACGAGGCGTCGGAGGTGCGACAAAGATGAGGAAGAAGCTGGGCACCCGGTTCCCCGCG
GTTCGTTCTCTCCCTCTCCTCCATGGCCCCTCTCTAATCTGGCTTGCCCGTGATCCGATCTGTCCG
TGCTCTCGGTGGATCTCGGTCCTCGCATGGTGGATCTCGCCGTTGACTGGCGCTTTGTTTGTCTTC
TTGTGGTGCGTTTCGTCGCCGAGGCGCCGCCCGGGTGGAGACGCGGAACGGGCTGACGTTCTTCTC
GGGCAAGCGCTCGTGGGCCGGGCGAGGAATCTAATCGTCCTTTTGCTTTGTCTCGCTAGTGAATTG

ATTTTGTCCCCTTGTTGAGTTCATTTGTGGTGGATGGATGGATGGAGTGAGGTGGACTCGGTAGTC
TACCGCGGTTGGTTTTGTGTGAGTGGAAAGATTCTTGATCACTCACTCGGAGCTGACTCTAACTTG
TGATGATCTGGCAAAAAGATCTATCTTGCAAGTGCAAGATGGTGTCGAAGAAGCAAACCCATTGCG
ATGTTCCTTCGTTGTAACTCGGTTGCTCGTTGTACAAGATGAGTAAATCGAGCTTGAATTGCGATT
TAATACTGTACTTTTGCTGCCCAAGTTGCTGGTAGCATGGGCGATCGCTTGCAAGATTCCTCAAGA
TGCAAACACGTGTGGTAAGTGATAGAAAGAAAGAAGGAAAGGCTGAGACAGGCTCAGCAGTGTAGT
AGTATAAAATTTGGCACATGCAACCTATAAATCTCGAGCCTTAATGTTTTCAGGACGTTTGGAACA
TTTTCACTGTTTTATTGTCACCAGCCTGACATCATTCAAGTTTTGAAATTGCACGAGACCTTTCCG
CTTTGCCCTGTATGAGTTCCTTTAGCGTGATAGTAATTTTAATAATACTTCTTGTAACATGCCTTG
TGTTTCCTTGTTGAGAGAACTAGCATGACATCGTGCACCCATTTTTTGGGTGTGTACTGATGGCTG
GAAGTTTTTGTTTATGTCTGTTGCTGAAACCTAGCTTTCCTTTGTTATTTTTGCTTAGCATTATCT
TATGTTGGCTAGTGTGGCTCTTTTGCTCTGTGTTATGTTGTATTCTGTAGGACTATATATTTCAGC
TGTTTGTCCAATCACTAATTTTATGTTGTTGTGTTAGGCACGGATCAAAAAGATAATGCAAGCAGA
TGAGGATGTTGGCAAAATTGCACTGGCTGTGCCTGTTTTAGTTTGTAAGTCCTGCAAGAATCTTCG
TGTGTCTAGTATCTGTTTTAAAATCTCTATGTTTGGCCCATATCTTCTGTTGCTTTTAAAGATATT
GCGGCATCACTGTTTTTTGCTGTGTGCATTGTAACACTAGATAATTCCATGATGAAGCATATAAAG
TGAGTATCTGGCATTCTGAATTTATTGTTTGCATGCAGCGAGAGCCCTTGAATTGTTTCTGCAAGA
TTTGATCGACCACTCATACAAAATTACTCTTCAAAGTGGTGCAAAGACACTGAATTCCTTCCACCT
GTGAGTTCTATTCTATGTCCATGGCTGTTTAATCTTACTATGACTTCGTGCTGTATGTATTGTATC
ACTCCATGTTTTTCTAGACATGGATAGCTTTGAGCCGTCTGTGCTTGCTTGCTTACTTGCTTACGA
AGCCTTTAATCCCAAAAAAGTTGGGGTAGGCTAGATATGAAACCCTTTCACGAGGACTTCCCAGGA
GGTCACCCATCCTAGTACTACTCTCGCCCAAATGGGTTTCATACCCAAAAGACTGGCTAGTTTTTA
CGTTGGCTCACCAAGCCTATCACAACCCTTAGATATGAAACCCTTTCACGACGACTTCCCAGGAGG
TCACCCATCCTAGTACTACTCTCGCTCAAATGGGTTTCATACCTATGGGACTGGCTAGTTTTTATG
TTGGCTCGCCAAGCCTATCACAACCCTCCTCCTTTACCCGGGCTTGGGACCGGCTATGCCGATTGC
CGAGAAGACATAGGCGGAGTAGACATAGGCGGAGTTAGCTTTGAGCCGTATGTGATTTACATATAT
TATAAGGGTCCATTCGGATGTATATGTTTTTCTAGATGAAAGTTAGTAATATGGTGGTTTTCTCCA
ATGTATCTCCATATATTATAAGGGTCCATTCGGATGTCCTCAACATCTACCAAATTCTCAACTCCT
GACTCCCTCTGATTCTAGTTCTGCGTGCGAAGGGTGAACATTTGGCACCATGTACAGACGAATGGT
CTTCATGGGTTGGTAGCCCATTTTGTTAGGATTTGGCTCGTTTAAAAGGGAAAGGAAGGCCAGGGC
TTGGCCCGTTCGGTGGGGAAGGACGTTGTCTCTAGCATGAACAACAGGGAAAGGAAGACCCTCGTC
GCACCGCCCATCCTCGAGGCCGCCCACTGGCGCACCGCTGACGAACAGCGCCAGAAGGGCCGCGTG
ACTGTCATCCTCCGTGGCTGCGAGCCTTCTCTCCGTTGGTGGATCTAGCCGGTGGCGGCTGTAATA
ACGGTGAGTAGGGCGAGGTAGCGCCGTCTGCGATTGTGGATGGTGAATCCGGCGCCGCCACCATTG
TTGTGGAGATGCAATGCCTAGTTGTGGACACCGATTTGGCTGATGGGAGGGAGAAATAAGAGCGAT
CAGGGAGAGAGAGACAAGAGAGACCGGGTGGAGACTCGCTCTGGTTCGGTTTCGCTCACGGGTGAG
GAGAGAAATCGATTATTTTTCCTATTGGTGGCATGGCCTTGTAATTTTGAAGAACTCCGTGAGCCA
CAGTTTTGGGGGGTGAAAGCGGAGCTGGCTTTGTGAAGCTGGGACGTTTGGTCTGGCTCCGTGTTC
GGAGTTGGCGGGGTGTGGAGCCGGAGGCCATCCAAATAGGCCCTAAGTCTATTTAACTTGTGGAAA
CTGCCCAGTTGCTGCTTTGAAGTTTTTCTAGTCTCTTACATATTACGCCACTAAATACCATTAAAT
TAAAGTATCTCACAGGCCTAACATGTGTGGTCTAATGTTGCTTGGACTTTTGGAGCTTAAATAAGC
TCTGTAACCTCTTGAAATGCCATCCCTTTGTATGGACGACTCTTAGCATGTGCAGTTTGATGTTCT
TGGCAAAAGTTTCTCTTTTGTATTTTCATTATTAACCTGACTATTATGATTTAAAACAGGCTATTT
TTTGTTTTCATTCTTTTAAGAGGTTTCATTACTCAAACTAGGGAAGGAAATCAACACCATACATAC
ATTAATTAAAACACGTAGCCAAGTATATTTTTTGCCTCTTGGGTCAGATAGCAAACAACAGCTGTG
CATCCCTCTCAATTTCATCTTGAATAACGACGCCTGAGCAATCCCTATTCCCACACCCTCCATGGA
AGCATCATCCGTAGCTCCCTTCATCATCTGTAATTCTTTCATGGCTGCTGCTGTCAAACTCCGCAC
CTTGTTTGGCTTCAAAAGCTCTCTGGATTCTAATGTGACAGCGAGTCTGGTGACTAGATGAATTAG
CTGAGTTCTGCTGGTCGTCATCTCGTAGTGTATTCCTGTTCTCCCACCATAGCCACCAAAACACAA
TTACTAGCAATTAGTGCTCCCCTGGAAGGCTCCAAAGAGGAACCAGAATATCGGAAGCTGACGAGC

ACAAGAAAATCATTGGGAAATTATTGTCAAGGCTCAAGATGTATCAGCAGGTCAGTTTCGGACAGG
TTCATCTTGGTATCCAATTGGAAACTGGTTTATGTTTTTCAGTGGAAAACTACTCTGGTATGATTT
CCGGTAATGAACTATAGGAGATTTGTGAAGATGGTCTGCCTTCCGAATGGAACTTGAGTGGAAGCT
GAGATGAGCCTGCTCATATGCAGCACATGTGTTTGTCTGACTTATCATGATTTACAACTTTCATCT
GTAATTGGAGCTACTAATTTGATTATAATCTAAGATTAATGATGTAGACTTTTCTTCTTTTGATAC
CTTTGATAATTGTGGTCATCAATGGTGTTTTCTTTTGTCAAAAATTTAGTTACACAAGTCAACCAC
AGCCACTTATGAGATGTTTAAACTGTGTGCCTTTGCCTTTAGTGACTAATGGTTTATATGTTTCTT
ATGAGATGCTCAGAAAGCAATGTGTGAAGAGGTACAGCTCTTTTGACTTCCTAACTGAGATTGTCA
ACAAGGTGCCAGATCTCGGTGGCGGTGAATCTTGTGGAGATGAAAGAGGATTACCCAGAAGAAGGT
AAGTTGACTTGGCATGGTCAAATTCTCATGAATCATTGTGATAAGTAACGATCATGTTATTAATGA
TCATGTTATTAATGATTGGAATTCACTTTCTAGGAAATTTTCAAATGGAAGCGACCCAGAGAATGA
GGAGCCCCGATCTAGCAAAATGGTACAATTCCATCACATATCTTCTTCTTACGAAACCTTTTATCC
CAAACAAGTTGGGGTAGGCTAGATATGAAACCCTTTCACGAGGACTTCCCAGGAGGTCACCCATCC
TAGTACTACTCTCGCCCAAATGGGTTTCATACCCAAAAGACTGGCTAGTTTTTACGTTGGCTCGCC
AAGCCTATCACAACCCTTAGATATGAAACCCTTTCACGAGGACTTCCTAGGAGGTCACCCATCCTA
GTACTATTCTCGCTCAAATGGGTTTCATACCTATGGGACTGGCTAGTTTTTACGTTGGCTCGCCAA
GCCTATCACAACCCTCCTCCTTTACCCGGGCTTGGGACCGGCTATGCCTAGAAGACATAGGTGGAG
TTACAATTCCATCACATATCAGCAGAGTTAAATTTTCTTAGCAGATAAGTTGATAACATGATTGGC
TTTGCAGCCCATAAGAAGCTTGAACACCAGTCCCAGAGGACGAGGCAGAGGTCGAGGAAGAGGGCG
AGGGCGGCCTCCAACCAAGAGAAAGGAAATTGGTTATGTACAGTTTGAGGATGAGAGCAGCATGTT
TGCTGAACAAAGTGAACCCTTGCCAGGAGATGAGATAGTTCCGGAGACCAACCGTGGCAATGAGAG
TATTCCCCAAAGCTCACATCCTCTAGTGGAGGCTCCATCAGCCATGACGCCAGCTGTGATTTCAAA
GGTTGAAGAAGCTAGCACCAACCATCAGCCAGATTGGCCTATGCCAGATGCCATTGGAGGCATTGG
TGTTGGACCATCCAGTTTTGGACATCTGACAGTGCAGGTTGATGAGGTAGAGGACTACGACAATGA
GGATTAGGCATAGTCATCCTCTCATTCATCATATGCTCTAACAGGACAGTTCTCCTGGTGTTGTAC
ATTGTAAATATTGTTTCAAGTAGTTACTGCAGCTATGATGTGTAACCTATTTCTTTTTCCCAATAA
TTGGTTCTGAGTTGCCGCATTGTTGACCTAACTGACTAGGATGTGCTAAATTTAACCCTGTATTTG
ACGGTTGAAGCAGTGATATTCTGTCGTCCTGTTATATTGTACATGTAACCAGCAGTAATTTTGTGT
TACTAGTATATCATGTCGTGACCATCATGCCACGAAATCATTCAGATGTAGCCAAGCAAGCAATGT
GTGTACGCGAGATATCTACCTTGACCTTTACGATTTGGAGGTGGATTTAGGGCAAAACACAAGGAA
CAAGGAGAAAAAACACGAGGAACAAGGAGAAACCAAGAGAAATCTAGATGAAAACATCCAACAGGA
TGTGATTCGCACCAACTACCCTCAAATCCAAGGTCAATTCAGGTACATTTTGCATCCAAGAACAAC
GCAACAAATAAAGGTAACCAAGGGGATATTTTTTCCTCAATCTCTCGAGAGACGTTTCGTCCCCGA
TCCAAAAGGATTCTTCCAAAAGGATTTTTCTTGTTAGGTCTTCATCCTGTATGAAGAAACAGAGCA
AGACAGAGCTTGCCCGGATCATTCGAACCCCCATCTGGATGATCCGGACCAAGCCCGGACGTTCTG
GACCCTCATCCGGACGATCCATCAAATGTGCCTGAAGAGTACAACAAGCCTCAGGACGAAGCCGGA
TCATCCAGATCCACCCGAACATCCGGCCCAACTACAGCGAGAACACAGCTGGACGATCCGGACGGC
TGCTCGGATCATCCGGATCCTCGACGCCCGGATCATCTGGATAGTGCCTGCGTGCATGGCTTGGCC
GAGGCCCATGTACCTCTCTCTACCCATAGACTATATATACTCCTCCCCTGCCCACGTTTTAGGGTT
AGCAAAGGCTTAGCTCAAGATAGAGGTAGAGCTTTGCTCATCCACTTACCTCTCCCATGGAATCAA
GGCCTCCATGTGAGAAGATCCCCCAAGTGGATTCATGACTCCTTCATGGGAAGATCCTTCTAGGAT
TGAAGACCTCTCTCTTTCAAGGATTGGGATGAACTAGCTACCCTTTGTTACCTACTTGTGTTGGAT
TTGAACCCTTGTGTCTTCTCTTTGTGTATGTGGATTTAACACGTGTGATTGGATGTATCAATTGAG
TGTTCTTCTGTTGATTTGCTCCTTTCTCCACCTCCAAGTGTGAAAAGATCATGGACTTGGGTTTCA
CCGTACATCATCTTGGATAAGAGCATAGGTTGATTCACATCTTGGAGTCCATACCCCCATTTGCTA
GCCTAATTTTGTGTGTTTTCCCCAATTTCAAAAATCCCCACAAAAGACCGATACCATTTTTTTTGT
GATTTGCTGGTTTGATAATGTTTTGTTGATTTTGATCCATGGATTCGTTGTGTTGCAAGTGGATCT
AGCATTCCCCGCCTCCTCCACCACAAAATCTACCCAAAATTGCCCCGATTTTTCTTTTTATCCATG
ATTTGGAGTTCTTCCCGACCGCATAGCCGCGCAGACGACCTGCCTGGATCATCCGGACGCACACCC
GGATCATCCGGACATCGCCTGGACCATCCGGACCGCGGCCTGAATCATCCGGCTTCCACTGTCAAA

ACTAAATTTTCGACAGAAAATTTGAGCGACCGCCTCCACTTCCGCATCGCCAACTCCGAGCCCCAC
CATAGCACTTTCGAAAATCCTTTGACACCCATAGCCATACGACCGCTTTCCGCTACCACCATTTTA
CATTTGCCATTGGGATTTTGAGTTGTGGTTTTTTGTTTCCTAACGTGTTTCGGCTACTTAGGAATG
ATTCGGCATCGACATCATCACCGCTCAAATTCGCCATGGACTCGACATCGACAACGACCACTCCCT
CATTTTGACACCACCAACCGTTAGAAAGGACGGTAACCTCGACAACATCACTTGTACATCTCCCTT
GCCATTGCATTATGAATCCCGAGCCAAATTTTGCGTCACTTTCCTATCGAGACTAGCCATTTGAGT
ATTGTTGGGCAACATTACTTGTGCACTCTAGTGATACATGTTCCGCATAGCTATCATAGCGTGCAT
CTATCATCGTATCATCTCTTGTCTTATCAATTGTTCCCGCATATACATAATTGCTATCTTGGTTCG
AGCATTTTGCATAAGTGGGC

**>*TaDrAp1-A4*-CDS-768bp**

ATGAGGAAGAAGCTGGGCACCCGGTTCCCCGCGGCACGGATCAAAAAGATAATGCAAGCAGATGAG
GATGTTGGCAAAATTGCACTGGCTGTGCCTGTTTTAGTTTCGAGAGCCCTTGAATTGTTTCTGCAA
GATTTGATCGACCACTCATACAAAATTACTCTTCAAAGTGGTGCAAAGACACTGAATTCCTTCCAC
CTAAAGCAATGTGTGAAGAGGTACAGCTCTTTTGACTTCCTAACTGAGATTGTCAACAAGGTGCCA
GATCTCGGTGGCGGTGAATCTTGTGGAGATGAAAGAGGATTACCCAGAAGAAGGAAATTTTCAAAT
GGAAGCGACCCAGAGAATGAGGAGCCCCGATCTAGCAAAATGCCCATAAGAAGCTTGAACACCAGT
CCCAGAGGACGAGGCAGAGGTCGAGGAAGAGGGCGAGGGCGGCCTCCAACCAAGAGAAAGGAAATT
GGTTATGTACAGTTTGAGGATGAGAGCAGCATGTTTGCTGAACAAAGTGAACCCTTGCCAGGAGAT
GAGATAGTTCCGGAGACCAACCGTGGCAATGAGAGTATTCCCCAAAGCTCACATCCTCTAGTGGAG
GCTCCATCAGCCATGACGCCAGCTGTGATTTCAAAGGTTGAAGAAGCTAGCACCAACCATCAGCCA
GATTGGCCTATGCCAGATGCCATTGGAGGCATTGGTGTTGGACCATCCAGTTTTGGACATCTGACA
GTGCAGGTTGATGAGGTAGAGGACTACGACAATGAGGATTAG

**>TaDrAp1-A4-Pep-255aa**

MRKKLGTRFPAARIKKIMQADEDVGKIALAVPVLVSRALELFLQDLIDHSYKITLQSGAKTLNSFH
LKQCVKRYSSFDFLTEIVNKVPDLGGGESCGDERGLPRRRKFSNGSDPENEEPRSSKMPIRSLNTS
PRGRGRGRGRGRGRPPTKRKEIGYVQFEDESSMFAEQSEPLPGDEIVPETNRGNESIPQSSHPLVE
APSAMTPAVISKVEEASTNHQPDWPMPDAIGGIGVGPSSFGHLTVQVDEVEDYDNED*

## ***TaDrAp1-B4*** (TraesCS4B02G161200)

**TraesCS4B02G161200 REVERSE STRAND**
pep chromosome:IWGSC:4B:316654107:316664575:-1 gene:TraesCS4B02G161200 transcript:TraesCS4B02G161200.1 gene_biotype:protein_coding transcript_biotype:protein_coding description:CCAAT-binding transcription factor C [Source:UniProtKB/TrEMBL;Acc:A0A0A7LUF6]

***>TaDrAp1-B4***

CAACCCTATCTCTTGTTTCCCCACACATACGGACTCTAAAAACAGCCTATACTCTGGTATTTTGAA
ATTACATGGACCTGACCCAATAATAAGGTGACGCAATACCTATAATAGCCTCTGGACGAAATTTAG
GAAGTGACATCTTGTATATTTCATCCGAGCCTTGATACGCTCATTGTGGTGGCTTCAAAGTCCTGA
AATCGTCACTTGAAACTCAGTCTTGTTTTCCTTGCGCATGCCATCATCTCCATGCTTGATCTTGCT
CAAGTGATCATCCCTCTTGTCCATGCTAGACCCTTTATTTGTAAGCAAAACAAATGTATCCAATTT
AGGCATCATCATATTCTCATGAACATTAGAAACATTACCAAGAAACAAAAGTACCTAATAATTTAA
TTGGCGCGCGCGAGCTCTAGTAATTTGTCCAGTATACGTAGCAGTAGGGGCTGTGGGTGTAACAAT
GATATTGATGTCCTCATCAAGAAGCAAGGAAAGGAGTTGGTAGGAAGCTACAGGCACCGGACGTCT
CACACATTCACTTACAACCAACCACAAAGCCAACAACCAACGAACGCTACAGCGGCCGGACATCCG
ACAAGTTCCAACAACCAGACGTCCGGTCTTCACTAGAAATGTTGTACCACATGTCCGGAAGCAAAA
TGATGGAAGTCTGACGCGCATTGGACATTTGGACCCTCCCGAGCCACCAGACGTCCGGCAAACACC
GGAAATCCCGTACCACCTGACCAGAGAAGAAATGGCGGAAGTCCGACGCCCAACCAGATGTTCAAT
CACTCGCGAACGACCAGACGTCCGGTATTGCGTGTGCGCATCCCGACGGTTTTCAGCCCATGTACC
CCTCTTTCACACCTAGACTATAAATACCCTTCTCCTACCTCCATCCTAGGGCTGGCAAAGACATAA
TAGAGAATTGCTAGAGCTTTGCTCATATTCATTATTCCTCTTGGAGGTCAAGACCTCCATGTGAGA
AGATCCCCCTAGTGGATGCAAGAGCCCTATAAGGGAATATCAATCTAGGGATGCAAGACCCCTATA
TGGGAAGATCCTCCATGGATGCTAGATCTCATCTCCTTTGGATTTGAGAAGAACCTTTACCTTTGT
GCTTCCCCCTTTTGACTTGTTAATATATATTTGTGGATCTCATGTATGCTATCTTAGTGATGTGTG
ATTTGGATTTGTTTGAGTCTCTCTCTCTCTCTCTTGTGTTATTGAGTGTTCATCCTTTCTCCATCC
TCCAAGTGCGAAAAGATCTTGAATCTAGGGTTTTGCCCTACATCACCATGCCTCCCTAGCCCCCCT
CTCCTCTCCTCCTTCCCTTCCCGCAGGTCACTGACCTCCTACTTGTGTCGGGTCCCTCTCTTCCTT
CCTTCCCCGTAGGTCGTTGAGCAAGGAATCGAACTGGGCAAACGTGACGGCCGGCCGGATCGATGC
TGAATTGACCGGATCCGACGTAGGGATCACAACAAGGAGGAGGAAGTCGGAGGAAGGAACGGTGGC
GGGCAGGGGCGGCGGCTACAATGTCGTCGGTGGAAGGAGCGACAATGGAGGCTCCGCCGACCATGG
CGAGGAAAGCGAGATCGGCGATGAGCTTCGCGGGGTCCGCTGCGGGGTCAGAGGAGCTGGCCATGG
CGACGCGGTGGCCGGCGGGAAAGCGGAGGCAACATTCCGGTTGCGCGCGGGAAGAAAAGGGTAGCG
ATTATTTTTCGTGATGCAAGCACTCTTTTGTGGCAGTCGCGTGACTACCCTATATTCGCTACACTC
GAAGCATTTTTTTTAGGTTGCCGGATTTTTTCAAGAAAATTTAGAATTTGATAGCTTTTCGTGCTT
AAAAACAATTCGTGTGTGAAAATAATAGGGCTACTGTGAGAGATGCGGTAAATATCTTTTGGCACG
AGTCCGCCGTGTTGGTGCAAGTTGGTGGAAAGACAACAAAACAAGAAACCCAATCGTCAAGAAGAA
CGAGAAAAGAAAATACACAAAAGGTAGACTACGATTGCCGGCATCGAGCTCGACGAGGCGTCGGAG
TTGCGACAAAGATGAGGAAGAAGCTGGGCACCCGGTTCCCCGCGGTTCGTTCTCTCCCTCTCCTCC
ATGGCCCCTCTCTAATCTGGCTTGCCCGTGATCCGATCTTCCGTGCTCTCGGTGGATCTCGGTCCT
CGCATGGTGGATCTCGCCGTTGACTGGCGCTTTGTTTGTCTTCTTGTGGTGCGTTTCGTCGCCGAG
GCGCCGCCCGGGTGGAGACGCGGAACGGGCTGGTGTTCTTCTCGGGCAAGCGCTCATGGGCCGGGC
GAGGAATCTAATCGTCCTTTTGCTTTGTCTCGCTAGTGAATTGATTTTGTCCCCTTGTGTCGAGTT
CGTTTGTGGTGGATGGATGGATGGAGTGAGGTGGACTCGGTAGTCTACTGCGGTTGGTTTTGTGTG
AGTGGAAATATTCTTGATCACTCACTCGTAGCTGACGCTAACTTGTGATGATCTGGCAAAAATATC
TATCTGGCAAGTGCAAGATGGCGTCGAAGAAGCAAACCTATTGCGATGTTCTTTCGTTGTAACTCG
GTTGCTCGTTGTACAAGATGAGTAAATCGAGCTTGAATTGCGATTTAATACTGTACTGTTGCTGCC
CAAGTTGCTGGTAGCATGGGCGATCGCTTGCAAGATTCCTCAAGATGCAAACACGTGTGGTAAGTG

ATAGAAAGAAAGGCTGAGACAGGCTCAGCAGTGTAGTAGTATAAAATTTGGCACATGCAACCTATA
AATCTTGAGCCTTAATGTTTTCAGCACGTTCGGAACATTTTCACTGTTTTATTGTCACCAGCCTGA
CATCATTCAAGTTTTGAAATTGCACGAGACCTTTCCGCTTTGCCCTGTAGGAGTTCCTTTTGCGTG
ATAGTCATTTTAATAATACTTCTCCTAACATGACTTGTGTTTCCTTGTTGAGAGAACTAGCATGAC
ATCGTGCACACATTTTTTGGGTGTGTACTGATGGCTGGAAGTTTTTGTTTATGTCTGTTACTGAAA
CCTAGCTTTCCTTTGTTTTTTTTGCTTAGCATTATCTTATGTTGGCTAGTGTGGCTCTTTTGCTCT
GTGTTATGTTGTATTCTGTAGGACTATATATTTCAGCTGTTTGTCCAGTCACTAATTTTATGTTGT
TGTGTTAGGCACGGATCAAAAAGATAATGCAAGCAGATGAGGATGTTGGCAAAATTGCACTGGCTG
TGCCTGTTTTAGTTTGTAAGTTCTGCAAGAATCTTCGTGTGTCTTGTATCTGTTTTAAAATCTCTA
TGTTTGGCCCATATCTTCTGTCGCTTTTAAAGATATTGCGGCATCACTGTTTTTTGCTGTGTGCAT
TGTAACACTAGATAATTCCATGATGAAGCATATAAAGTGAGTATCTGGCATTCTGAATTTATTGTT
TGCATGCAGCGAGAGCCCTTGAATTGTTTCTGCAAGATTTGATTGACCGCTCATACAAAATTACTC
TTCAAAGTGGTGCAAAGACACTGAATTCCTTCCACCTGTGAGTTCTATTCTGTGTCCATGGCTGTT
TAATCTTACTATGACTTCGTGCTGTATGTATTGTATCACTCCATGTTTTTCTAGACATATGGATAG
CTTTGAGCCGTCTGTGATTTACACATTGTACTTAGATGAAAGTTAGTAATATGGTGGTTTTCTCCA
ATGTATTTCCATATATTATAAGGGCCCATTCGGATGTCCTCAAAATCTACCAAATTCTCAACTCCG
ACTCCCTCTGATTCCAGTTCCTCATGCGAAGCGTGAACTTTGGCACCATGTACGGCCGAATGGTCT
TCATGGGTTGGTAGCCCGTTTTGTTAGGACTTGGCTCGTTAAAAGGGAAAGGAAGGCCAGGGCTTG
GCCCGTTCAGTGGGGAAGGACGTCGTCTCTAGCACGACCAACAGGGAAAGAAAGATCTCGTCGCAC
CGCCCATCCCTTGAGGCCGCCCACTGGCGCACCGCTGACGAACAGCGCTAGAAGGGCCACGTGACT
GCCGTCCTCTGTGGCTGCGAGCCTTCTCTCCGTTGGTGGATCTAGCCGGTGGCGGCTGTAATAACG
GTGAGTAGGGAGAGGTTGCTCCGTCTGTGATTGTGGATGGTGAATCCGGCGCCGCCACCATTGTTG
TGGGGATGCGAGACCTAGTCGTGGCCGGCGATTTGGCTGATGGGAGGGAGAAATAAGAGCGATGAA
GGAGAGAGAGACAAGAGAGACCGGGTGGAAACTCGCTCTGGTTCGGTTTCGCTCACGGGTGAGGAG
AGAAATCGAATCGTTTTTCCTATTGGTGGCATGGCCTTGTAATTTTGAGGAACTCCGTGAGCCACA
GTTTCGGGAGGTGAAAGCAGAGCTGGCTTCGTGAAGCTGGTGTTAGGAATAAGCAACTTGTCTTTC
CATGAGGCCATAGGCCGATATATATATACATGTACAGGTGTGGAACATATGCAGGAAACCCCTCAT
ACAACGGGATAAATACAAAGGGGTACATGACTTATATTATAACTCTAACACCCCCCTCAAACTCA
TGGTGGATGAACAACACTGAGTTTGGAGAGATAAAAGCCATGTTGTGCTCTAGTCTGAGCCTTTGT
CAGGAAATCCGCCAACTGTAACTCGGAAGGCACATACTGAAGAGCAATAACCTGATCCTGCACAGC
AGCACGCACATAGAAAGCATCAACACCAATATGCTTGGTGAGCTCATGCTTCACAGGATCGCGTGC
AATGCTGATAGCACCTGTACTGTCAGATAGAAGCAGAGTCGGTGTAGTGACAGAAACACCAAAATC
CTGAAGTAACCACTGTAACCAAGTCACCTCTGCCGTCAAAAGAGCCATTGCTCGCAACTCAGCCTC
GGCACTCGAACGGGAAACTGCAAGCTGTTTCTTCGTCTTCCAGGCAATGAGAGAACCACCAAGAAA
AACACAGTAAGCAGAAAGTGAACGGCGATCGGAAGGATCACTAGCCCACGTAGCATCCGAATAGGC
CTGGAGCTGTAAAGAACTGGAGCGAGGAAAGAATAGACGGTGAGAGATCGTGCCTCGAAGATATCG
AAGAACACGAAGGAGATGACTATAGTGAACCGAGGTGGGGGCAGAGACAAACTGACTCAGAATATG
AACCGGATAAGATATGTCCGGACGAGTGACAGCTAGATAGACAAGACTGCCAACAAGATGACGATA
ACGCGTCGGATCAGGGAGAGGATCACCATCAGTAGCACGGAGGTGAACATTGAGCTCCATAGGAGT
CTCAACAACGCGCTCGTCAGTAAGAGCAGCACGAGCAAGAAGATCCTGGATATACTTTTCCTGGGA
TATAAAAAAGCCATCAGAGGTAGAAGAGACTTCAATCCCAAGAAAGTAGCGAAGAGGTCCAAGATC
AGACATAAGAAACTGCTCACTAAGACGGGCCTTTACAAAGGCAATATACTCGGGATCATCTCCCGT
GATGATCATGTCATCAACATAAAGAAGAAGAAGGGTCCGACCACGAGGAGAAAGGTGAATAAACAA
TGCGGGATCATGAGCACTTGCTAAAAAACCAGCAGCAGTGATCACAGAGGCAAAGCGCTCAAACTA
GGCGCGGGGGGCTTGCTTAAGGCCATAGAGAGAGCGACGAAGACGACATACCATGCCATCAGGAAC
AGAATACCCAGGTGGTGGCTGCATGTACACCTCCTCACGCAGCTCACCATTAAGAAAGGCATTCTT
AACATCAAGCTGAGATATAGACCAGTGGCGTGCAGAGGCAACGGCAAGAAGGGTACAAACAATGGT
CATATGAGCCACAGGAGCAAAAGTCTCGTCATAATCACGACCATGCTCCTGCTGAAAACCACGAGC
CACAAGACGAGCTTTGTGACGCTCAAGAGAACCATCGGAGCGAGTCTTAACCTTGTAGACCCACTT
ACAAGTGATCGGATGAACTCCGGGAGGAAGAGAAACAAGATCCCAAGTACCAGTGCGTTCAAGAGC

AGCAATCTCCTCTGCCATCGCAAACTGCCATTCAGGATGAACAACAGCCTGACGGTAAGAAGTCGG
CTCAAGAACAGCAGCACCAGCGGTGGGAAATCCAAAGCGATCAACAGGCGGACGAGGACGAGAACG
CAAGCCATAAGTAGGCTGAGAGGAAGATGACGACCCATCCACAGAGGCATCAACTGGTCGTGGACG
ACGAGTGTAATGCTGAGGAAGAGATGGAATAATAGAAGGAGGAATCGACAAGGTAGAATCAGGGGG
TGGCGACGAAGAAGTCACCGGAGATGAAGGTGTAGAATCCGGTGACAAACTGGGAGAGGAAACCGG
GGAGGAAGTCACCGGAGATGAGGGTGGAGAACCAGGTGACATGCTAGGCGAGGAAACCGGGGAGGA
TGGTGGTTGCAAGTCAACTAGATGTGAAGAGGAAGTGGAACAGAGAGGTACAGTGTCGGCAGGGGT
GATAAGTGAGTCAGGAAAAGTGAGGAAAGAGATATCCTGCACTGAAAAAGTCGAGGAAGATGGGCG
TGGGTAGAAGGGACGAGACTCTTCAAAAGTCACATCTCGAGAGATACGCATCCGACGACCACTAGG
ATCCCAACAACGATAGCCCTTATGCTCATCACTGTAGCCTAAGAAGACACACTCAACAGACTGAGC
GGTCAGTTTGGTGCGTTCGCGAGGGGCAAGAAGAACATAGCAAACACAACCAAACGAGCGAAGCAT
CGAATAATCGGGAGAACGATCAAAAAGTCGCTCGAAAGGAACACCACCCTGTAGAGCAGCGGAAGG
CTGTATATTGATAAGATAGGTGGATGTGGAGACGGCCTCAGCCCAAAAATGAGGCGGGAGAGAGGC
AGCAATCATCAATGCACGAGCCGTCTCAAGAAGATGTCGATGCTTTCGCTCAGCCACACCATTCTG
AGCATGAGCACCAGGACAAGAGAATTGAGAGAGAGTCCCGTGCTCAGCAAGAACACCACGCAACAT
CTTAGAGATATACTCGCCAGCAGAGTCAGCACGAAAAACACGAATGGGTGAAGAGAACTGAGTATG
AACCATGGCAGCAAAACGCTTATAAATTTTTAACACCTCAGAACGAGAAGTCATGAAATAAAGCCA
TGTGTAACGAGAGAAATCATCTATGAAAATAATATAGTATTTATGACCACCTTTCGAAGCGAAAGG
GGCCGGACCCCATACATCAGAATGGACTAAATCGAAAGGACGCTTAGACACGGACTCACTATGTGA
ATATGGTAACTGAATCTGCTTGCCAAGACGACAACCCTGACACTCTAAAGAGACATCTCCTGAGAC
AGACCCCAGAAGGCCTCGACGAACTAAAGAAGACAACCGAGAACCACACAGATGACCAAGTCGATG
ATGCCACTGCTGGAAAGAACCAGTGACGGAGGCAACAGAAGCGGGAGAACCGGCGATGGTGGTGGC
AGCAGAAGGAACATGAAGCCAGTCCAACTCCCAAAGACCCTGAGAATCACGGCGGCGAGGGCCAGC
CCCAACCAGAGTGTGCGTATGACGGTCCTGGACAGAACAAGAGTCAACGTCAAGGATAACGCGACA
ACCAGAATCCGTAAGTTGACCAGCAGAAAACAAATTCATGGTAAGTCGAGGAACATGAGCAACATC
AGGAACAGAATAAGGAGTAATAAGATTGCCTCTACTAGCAACAGAAAGGGGAGTACCATCAGCAGT
GAGGACATGAATAGGAGAATCGAGTGATCTAAGAGAGGACAAAATGGAAGAATGAGAAGACATATG
AAAAGAAGCTCCAGAGTCCAGAACCCATGAGGATGTACCTGACTGTGTAGAGGGTGATCGCTCAGT
GCGGGAAGCATCAGTCACAGAACCAGCAGTACCCGTCGAGGAAGAACCTGAAGCCGCAAGCAGACG
CTTAAGTCTCAGAATATCCTGCTCAGTCAAAGCAATGGCTGAAGCTGTCGAGGTAGATGACGAAGT
CCCTGAAGATGATGATCGAGCCTTGCGCAGGTGTTTCTTCTTCGTGTAGCACTGAGACTCAAGATG
ACCATCATTGTTGCAATAGGCGCAATGTGGACGGGGGCGACCTGAGCCTCCAGAAGGAGTGGGCAA
GAGCGGCGGAGCACTCGAGCGAGAAGGGGTCGGTGGAGCAAGTGGCGTAGAAGCATGAGTAGTAAG
CACAGAGGGAACCTCCAGCAAACCAGCACCACGTAAGCGAGTCTCCTCAGCACGAATCTCAGAAAG
CGCCTCCATGAGAGAAATACGGCCACGAGCAAACAACTGAGCACGCCGGGGCTCAAACTCCTTACG
GAGCCGAGACAAGAACTCATAGACGCGATGAAACTCCAAATTAGCCTGGACAGCCTGGCAACAGGG
GCAGGTACGACACCCAGCACTACGAAGAGAATCAAGCTGGCGCAAGATAGCAGAACTCTGTGCATA
GAAGTCATCAACAGAAGAGTCACCCTGCTGAAGAGCATGCTCCTGACGGACCACAGAGAGGTATAA
GGCATCACCAGAGGGCTGATAGCGCTCACGAAGACGGGTCCACATCTCAAAGACGGTAGAAAGACC
CAGAAATTCAGAGGCAAACTGAGGCAGAACACTAGCAGTGAGAACAGCGGCAGCACGAGCATCATC
ATCAAGCCACTGGGTGTAAACAGACAGAGCACCATGATACGTCTGAAGAGCCTCCTCATAAGCCAA
AACCCTCTCATCATAAGCACAATCAGCAGCATCATCAGCCTTAGCCGCATCCTTGGCGGCCTGATT
AGCATCCGTAGGAAGAACCGATGGAGTTGGCGGAGTAGGGGCCACCGGAGGAACTGGACGTGGCGG
ACAGCAGACCTCGCCAGAAAGAACACCCCAAAGACGGATGCCACGCATGTGAATGCGCATGAAGCC
AGTAAACTCGGTGTAGTTAGTACCATCAAAGATCACCGGACAGCGAGGAACAGCAACATAGCCCGA
TGCAGCAGACATTTCTTTTNNNNNNNNNNNNNNNNNNNNNNNNNNNNNNNNNNNNNNNNNNNNNNN
NNNNNNNNNNNNNNNNNNNNNNNNNNNNNNNNNNNNNNNNNNNNNNNNNNNNNNNNNNNNNNNNNN
NNNNNNNNNNNNNNNNNNNNNNNNNNNNNNNNNNNNNNNNNNNNNNNNNNNNNNNNNNNNNNNNNN
NNNNNNNNNNNNNNNNNNNNNNNNNNNNNNNNNNNNNNNNNNNNNNNNNNNNNNNNNNNNNNNNNN
NNNNNNNNNNNNNNNNNNNNNNNNNNNNNNNNNNNNNNNNNNNNNNNNNNNNNNNNNNNNNNNNNN

NNNNNNNNNNNNNNNNNNNNNNNNNNNNNNNNNNNNNNNNNNNNNNNNNNNNNNNNNNNNNNNNNN
NNNNNNNNNNNNNNNNNNNNNNNNNNNNNNNNNNNNNNNNNNNNNNNNNNNNNNNNNNNNNNNNNN
NNNNNNNNNNNNNNNNNNNNNNNNNNNNNNAGAGATCGATGAAGAGGCAGCTGGCAATCGGCGGCT
GAAGAACCGCAGAGTCAAGGGCCGGCGGCAGCTGCGTTGATTGGAGCCAGGAGCAGGAGATCAGAC
CGGCTCTGATCTGAGTCCACACCGGATCAGGCCGGCTGGGATCAGGCCAGCTGGGAGCGATCCTGC
CTGGGAGCGATCCTGCCTGGGAACGAGGAGCGGGAGAGGAAGAGCGGGCCGGCTGGGATCGCTCCT
GCCTGGAGAACGAGGAGCGGGAGAGGACGATCGGGCCGGCTGGGAGCGATCTGAGCTGAGATCGAG
GAGCCGGACGGAGATCGAGGAGCCCAGACGAGATCGATCCGATCCGATCGGGAGAGAGAGATAAGC
CAGAAGGGATCAATAGCACGAGTTGCAGCGTGCAAAAAATTGACCTAGCTCTAATACCATGTTAGG
AATAAGCAACTTGTCTTTCCATGAGGCCATAGGCCGATATATATATACATGTACAGGTGTGGAACA
TATGCAGGAAACCCCTCATACAACGGGATAAATACAAAGGGGTACATGACTTATATTATAACTCTA
ACAGCTGGGACGTTCGGTCTGGCTCCGTGTTCGGAGTTGGCGGAGTGTGGAGCCGGAGGCCATCCA
AACAAGCCCTAGGTAGCTTGTGGAGACTGTCCAGTTGCTGCTTTTAAGTTTTTCTAGTCGCTTACA
TATTACGCCACTAAATACCATTAAATTATAAAGTATCTCACAAGCCTAACATGTGTGGTCTAATGT
TGCTTGGACTTTTGGAGCTTAAATAAACTCTGTAACCTCTTGAAATGCCATCCCTTTGTATGGACG
ACTCTTAGCATGTGCAGTTTGATGTTCTTGGCAAAAGTTTCTCTTTCGTATTTTCATTATTAACTT
GATGACTATTATGATTTAAAACAGGGTATTTTTTGTTTTCATTCTTTTAAGAGGTTTCATTACTCA
AACTGGGGAAGGAAATCAACACCATACATTAGTTAAAACACGTAGCCAAGTATATTTTTTGCCTCT
TGGGTCAGATAGCAAACAACAGCTGTGCATCCGTCTCAGTTTCATCTTGAATAACGACGCCTGAGC
AATCCCTATCCCCACACCCTCCATGGAAGCATCAGCCGTAATTCTTTCATGGCTGCTGCTGTTAAA
CTCCGCACCTTGTTTGGCTTCAACAGCTCTCCGGACTCTAATGTGACAGTGAGTCTGGTGACTAGA
TGAATTAGCTGAGTTCTGCTGGTCGTCATCTCGTAGTGTATTCCTGTTCTCCCACCATAGCCACCA
AAACACAATTACTAGCAATTAGTGCTTCCCCGGAAGGCTCCAAAGAGGAACCAGAATATGGAAAGC
TGACGAGTACAAGAAAAACATTGGGAAATTATTGTCAAGGCTCAAGATGTATCAGCAGGTCAGTTT
CGGACTGGTTCATCTTGGTAACCAATTGGAAACTGGTTTATGTTTTTCAGTGGAAAACTACTCTGG
TATGATTTCCAGTAATGAACTATAGGAGATTTGCGAAGATGGCCTGCCTTCCGAATGGAACTTGAG
TGGAAGCTGAGATGTGCCTTCTCATATGCAGCACATGTGTTTGTCTGACTTATCATGATTTATAAC
TTTCATCTGTAATTCGAGCTACTAATTTGATTATAATCTAGGATTAATGATGAAGACTTTTCTTCT
TTTGGTACCTTTGGTAATTGTGGTCACCAATGGTGTTTTCTTTTGTCAAAAATTTAGTTACACAAG
TCAACCACAGCCACTTATGCGATGTTTAAACTGTGTGCCTTTGCCTTTAGTGACTAATGGTTTATA
TGTTTCTTATGAGATGCTCAGAAAGCAATGTGTGAAGAGGTACAGCTCTTTTGACTTCCTAACTGA
GATTGTCAACAAGGTGCCAGATCTCGGTGGCGGTGAATCTTGTGGAGATGAAAGAGGATTACCCAG
AAGAAGGTAATTGACTTGGCATAGTCAAATTCTCATGAATCATTGTGATAGCATGTTATTAATGAT
TGAAATTCACTTTCTAGGAAATTTTCAAATGGAAGCGACCCAGAGAATGAGGAGCCCCGATCTAGC
AAAATGGTGCAATTCCATCACATATCAGCAGAGTTAAATTTTCTTAGCAGATAAGTTGATAACATG
ATTGGCTTTGCAGCCCATAAGAAGCTTGAACACTAGTCCCAGAGGACGAGGCAGAGGTCGAGGAAG
AGGGCGAGGGCGGCCTCCAACCAAGAGAAAGGAAATTGGTTATGTACAGTTTGAGGATGAGAGCAG
CATGTTTGCTGAACAAAGTGAACCCTTGCCAGGAGATGAGATAGTTCCAGAGACCAACCGTGGCAA
TGAGATTATTCCCCAAAGCTCACATCCTCTAGTGGAGGCTCCATCGGTCGTGACGCCAGCTGTGAT
TTCAAAGGTTGAAAAAGCCACCAACCATCAGCCAGATTTGCCTATGCCAGATGCCATTGGAGGCAT
TGGTGTTGGACCGTCCAGTTTTGGACATCTGACAGTGCAGGTTGATGAGGAAGAGGACTACGATAA
TGAGGATTAGGCATAGCCATCCTCTCATTCATCATATGCTCTAACAGGACAGTTCTCCTGGTGTTG
TACATTGTAAATATTGTTTCAAGTAGTTACCGCAGCTATGATGTGTAACCTATTTCTTTTTCCCAA
TAATTGGTTTCGAGTTGCCTCGCATTGTTGACTTAACTGACTAGGATGTGCTAAACTTAACCTTGT
ATTTGACGGTTGAAGCAGTGATATTCTGTCGTCCTGTTACATTGTACATGTTCTAACCAGCAGTAA
CTTTTTCTAGTATATCGTGTCGTGACCATCATGCTACGAAATCACGTCATTCAGATGTAGCCAAGC
AAGCAATGTGTGTACGAGAGATATCTACCTTGACCTTCACGTTTTGGAGGTGGATTTAGGGCAAAA
CACAAGGAACAAGGAGAAACAAAGAGAAATCTGGAGGAAAACACTCAACAGGACGTGATTCACACC
AACTACCCTCAAATCCAAGGTCAATACAGGTACATTTTGCATCCAAGAACAACACAAGAAATAAAG
GTAACCAAATGGATAGTTTTTCCTCAAATCTCTAGCAGAGACGGTTCGTCTTTAATCCAAAAGGAT

TCTTCCAAAAGGATTTTTCTCGTCAGGTCTTCATCCGCTATGAGGAAATAAGATGAACAAAACCCC
CTCAAGATCTAGTTTATGCTTTGCTAAGCCTACAAAGTGCAAGAACTACGAAGAGTGGAACCCTAG
ATGGCCTAATCTTTCATAGCTTGGAGGGGGAATCCAAGAGAAACACACAAATCACAAGATGGAACA
CTCAAAGACAAGTCCAAATCACACATCCACTAGATGGGCAACACATGAGATCCATAAGCATACACA
AAAAATCCAAGGGAAATAGCAAGAAGAGTAAGGTCCATCTCAAATCCATAGAGGACATGAGGTCTT
GGAGGTAGGTAGTTCTTCCCAAATCTCTAGGAGAGATGGAGGCGTGAATCCACGAGAGGATCTTCC
AATAAAGGGCCTTGATATCCACTTGGGATCTTGATGCGGAGCATCCCACAGTCATCCCCATGGACC
TACCTTCGTCTTCCTAAGTGACACGTGGACCGATGACATGAGCGCGTGCAAGAGCTATCGAGACCG
AGGTGACATCGCTTCTCGCTCAACTCCTTTTTGAATCACATGAGACATGGCCACTACCTCAAACGG
AGAAACTTTGCATACTCACGTACCAAGGAACTAAGCATGGAGAAGCTAAGGAGCAAGGTCGAACGA
TGGAGGGAGAATAACACGAAGATAGAGAAGAAACGGAGCAAGATAGAGCTTGCCCGGATCATCCGG
ACCCCCATCCGGACGATCCATGTAATGTGCCCGAAGAGTACATCAAGCCTCTGGACGAAGCCGGAT
CATCCGGACCCATCCGGACGACGACCCGGACATTCGGATCAACTACGGCCGCGACGACATAGTCGG
ACGATCCGGACGACCATCTGGATCATCGAGACCCCGACGCCCAGATCATCTGGACAGTGCTTGCGT
GCATGGCTTGGGCTAAGGCCCATGTACCTCTCTCTACCCCTAGACTATATATACTCCTCCCCCGCC
ACATTTTCGGGTTAACAAAGTGATGAGGACATCAATACCATTGTTACACCGCAACCCCTGCTACTA
TACATACTGGACCAATTCTAGAGCTCGTGCACGCCAATTAAATTACCAGGTACTTTCGTTTCTTGG
TAATGATTCTAATGTTCATGAGAATATGATGCTGCCTAAATTGGATACATTTGTTTTGCTTACAAA
TGAAGGGCCTAGCTTGGAGAAGGATGAACATTGGAGAAAGAACAAGCATAGAGATGACGGCATGCG
CAAGGGAAACAAGAACAGAGTTTCAAGTGATGATTTCAGGACTTTGAAGCCACCGTAATGAGTACA
TGAAGTCTTGGACGAAATATACAAGATGCCACTTCACAAATTTCGTCCAGAGGCTATTATAGGTGA
TGTGTCACCTTATTATTGGGCCAGGCCCATGTAATTTCAAAATACATAAGTATAGGCTGTTTTTAG
AGTCCATATGTGATGCGGAGCATTCCACGATCATCCCCATGGACGTGCCTACATCTCCCACGACAC
CATTTGGACCTATGACAAGAGCTCGAGCAAAGGCTATCGAAGATAAGGTGAACTCGCTCCTCTCTG
AACTACCCCTTTCTACTTATGAGACATGGCTTCTACCTCATGCGGAGACCCTATGTGTGATCAGGT
GTTTGGAGGAGATCCACGGATCAGGTA

**>*TaDrAp1-B4*-CDS-765bp**
ATGAGGAAGAAGCTGGGCACCCGGTTCCCCGCGGCACGGATCAAAAAGATAATGCAAGCAGATGAG
GATGTTGGCAAAATTGCACTGGCTGTGCCTGTTTTAGTTTCGAGAGCCCTTGAATTGTTTCTGCAA
GATTTGATTGACCGCTCATACAAAATTACTCTTCAAAGTGGTGCAAAGACACTGAATTCCTTCCAC
CTAAAGCAATGTGTGAAGAGGTACAGCTCTTTTGACTTCCTAACTGAGATTGTCAACAAGGTGCCA
GATCTCGGTGGCGGTGAATCTTGTGGAGATGAAAGAGGATTACCCAGAAGAAGGAAATTTTCAAAT
GGAAGCGACCCAGAGAATGAGGAGCCCCGATCTAGCAAAATGCCCATAAGAAGCTTGAACACTAGT
CCCAGAGGACGAGGCAGAGGTCGAGGAAGAGGGCGAGGGCGGCCTCCAACCAAGAGAAAGGAAATT
GGTTATGTACAGTTTGAGGATGAGAGCAGCATGTTTGCTGAACAAAGTGAACCCTTGCCAGGAGAT
GAGATAGTTCCAGAGACCAACCGTGGCAATGAGATTATTCCCCAAAGCTCACATCCTCTAGTGGAG
GCTCCATCGGTCGTGACGCCAGCTGTGATTTCAAAGGTTGAAAAAGCCACCAACCATCAGCCAGAT
TTGCCTATGCCAGATGCCATTGGAGGCATTGGTGTTGGACCGTCCAGTTTTGGACATCTGACAGTG
CAGGTTGATGAGGAAGAGGACTACGATAATGAGGATTAG

**>TaDrAp1-B4-Pep-254aa**
MRKKLGTRFPAARIKKIMQADEDVGKIALAVPVLVSRALELFLQDLIDRSYKITLQSGAKTLNSFH
LKQCVKRYSSFDFLTEIVNKVPDLGGGESCGDERGLPRRRKFSNGSDPENEEPRSSKMPIRSLNTS
PRGRGRGRGRGRGRPPTKRKEIGYVQFEDESSMFAEQSEPLPGDEIVPETNRGNEIIPQSSHPLVE
APSVVTPAVISKVEKATNHQPDLPMPDAIGGIGVGPSSFGHLTVQVDEEEDYDNED*

## ***TaDrAp1-D4*** (TraesCS4D02G158200)

**TraesCS4D02G158200** **<u>REVERSE STRAND</u>**
pep chromosome:IWGSC:4D:220464598:220469329:-1 gene:TraesCS4D02G158200
transcript:TraesCS4D02G158200.1 gene_biotype:protein_coding
transcript_biotype:protein_coding

**>*TaDrAp1-D4***
GCATGTTTTATTATTGGTGTTGTCCTATGGTGCTCTCCGTGTCGCGCAAGCGTGAGGGATTCCCGC
TGTAGGGTTTGCAATATGTTCATGATTTGCTTATGATGGATGGCATGAGTGACAGAAGCATATACC
CGAGTATGTAGGTTGTTTGCGTATGGGAATAAAGAGGACTTGATACTTTAATGCTATGGTTGGGTT
TTACCTTAATGATCTTTAGTAGTTGCGGATGCTTGCTAGAGCTCCAATCATAAGTGCATATGATCC
AAGTAGAAATTATGTTAGCTTATGCCTCTCCCTCAAATAAAATTGCAATAGTGATTACCGGTCTAG
TTATCGATTGCCTAGGGACAAATAACTTTCTCGTGACAAAAAGCTCTCTACTAAAACTAATTTAGT
TGTGTCTTTATCTAAATAGCCCCTACTTTTTATTTACGTGCTCTTTATTATCTCGCAAACCTATCC
GAAAACACCTACAAAGTACTTCTAGTTTTATACTTGTTCTAGGTAAAGCGAACGTCAAGCGTGCGT
AGAGTTGTATCGGTGGTCGATAGAAGTTGAGGGAATATTTGTTCTACCTTTAGCTCCTCGTTGGGT
TCGACACTCTTACTTATCGAAAGAGTCTACAATTGATCCCCTATACTTGTGGGTTATCAAAGCTAC
ATGCACCGGACGTCCGACACGTTCACTTACAGCCAACCACAAAGCCAATAGCCGACGAACGCTACA
GCGACCGGACGTCCGACAAGTTCCAGCAATCGGACGTCCGGTCTTCACCAGAAATCCTGTACCACA
TGCCCGGAAGCAAAATGACGGAAGTCCGACGCGTACGGGATGTCTGGACCCTCCCGAGCCACTAGA
CGTCCGGCAAACACCGGAAATCCCGTACCACCTGACCGGAGAAGAAATGGCGGAAGTCCGATGCCC
AACCGGATGTTCGATCACTCGTGAACGACCGAACGCCCGGTACTCCCTGTGCGCATCCCGACGGTT
TTCAGCCCATGTACCCCCTTTTCATCCCTAGACTATAAATACCCTTCTGCTAGCTCCATCCTAGGG
CTGGCAAAGACATAATAGAGAATTGATAGAGCTTTGCTCATATTCATTATTCCTCTTGGAGGTCAA
GACATCCATGTGAGAAGATCCCCTAGTGGATGCAAGAGCCCTATAAGGGAAGATCCATCTAGGGAT
GCAAGACCCCTATATGGGAAGATCCTCCATGGATGCTAGATCTCATCTCCTTTGGATTTGAGAAGA
ACCTTTACCCTTGTGCTTCCCCCTTTTGACTTGTTCTTGCATATTTGTGGATCTCATGTATGCTAT
CTTAGTGATGTGTGATTTGGATTTGTCTGAGTCTTTCTCTCTCTTGTGCTCTTGAGTGTTCATCCT
TTCTCCACCTCCAAGTGCGAAAAGATCTCAAATCTAGGGTTTTGCCCTACATCACCGTGCCTCCCT
AGCCCCTCCTCTCCTCTCCCCCTTCCTTCCCCGCAGGTCACTGACCTCCTACTCATGTCGGGTCCC
TCTCCTCCTTCCTTCCCCGTAGGTCGTTGAGCAAGAAATCGAGCTGGGCAAACGTGACGGCTGGCC
GGATCAATGCCGAGTTGTCCGGATCCGACGCAGGGATCACAACAAGGAGGAGGAAGTCGGAGGAAA
GAGAGGTGGCGGGCAGAGGCGGCGGCGACGGTGTCGTCGGTGGAAGGAGCGGCAATGGAAGCTCCG
CCGACCATGGCGAGGAAAGCGAGATCGGCGGTGAGCTTCGCGGGGTCGGTTGCAGGGCCAAAGGAG
CTGGCCATGGCGACGTGATGGCCGGCGGGAGAGCGGAGGCAGCGTTCCGGTTGCGCGCGGGTAGAA
AAAGGTAGCTATTGGGTTTCATGATGCAACCACTCTTTTGTGACAGCTGCCCGACTACTCTATATT
CGCTGCACTGAGGATTTTTAAAAAAAATTAGAATTCGGTAGCCTTTCGTGCTTAAAAACAATTCGT
GTGTGAAAATAATAGGGCTGGGAGATGCGGTAATTATCTTTTGGCACGAGTCCGCGTGCCGTGTAA
GTTGGTGGAAAGACAACAAAACAAGAAATCCAATCGTCAAGAAGAACGAGAAAAGAAAATACACAA
AAGATAGACTACGATTGCCGGCATCGAGCTCGACGAGGCGTCGGAGGTTGCGTCAAAGATGAGGAA
GAAGCTGGGCACCCGGTTCCCCGCGGTTCGTTCTCTCCCTCTCCTCCATGGCCCCTCTCTAATCTG
GCTTGCCCGTGATCCGATCTGTCCGTGCTCTCGGTGGATCTCGGTCCTCGCATGGTGGATCTCGCC
GTTGACTGGCGCTTTGTTTGTCTTCTTGTGGTGCGTTTCGTAGCCGAGGCGCCGCCCGGGTGGAGA
CGCGGAACGGGCTGATGTTCTTCTCGGGCAAGCGCTCGTGGGCCGGGCGAGGAATCTAATCGTACT
TTTGCTTTGTCTCGCTAGTGAATTGATTTTGTCCCCTTGTGTTGAGTTCGTTTGTGGTGGATGGAT
GGATGGAGTGAGGTGGACTCGGTAGTCTACTGCGGTTGGTTTTGTGTGAGTGGAAAGATTCTTGAT
CACTCACTCGTAGCTGACGCTAACTTGTGATGATCTGGCAAGTGCAAGATGGCGTCGAAGAAGCAA
ACCTATTGCGATGTTCCTTCGTTGTAACTCGGTTGCTCGTTGTACAAGATGAGTAAATCGAGCTTG
AATTGCGATTTAATACTGTACTGTTGCTGCCCAAGTTGTTGGTAGCATGGGCGATCGCTTGCAAGA

TTCCTCAAGATGCAAACACGTGTGGTAAGTGATAGAAAGAAAGGCTGAGACAGGCTCAGCAGTGTA
GTAGTATAAAACTTGGCACATGCAACCTATGAATCTTGAGCCTTAATGTTTTCAGGACATTCGGAA
CATTTTCACTGTTTTATTGTCACCAGCCTGACATCATTCAAGTTTTGAAGTTGCACGAGACCTTTC
CGCTTTGCCCTGTAGGAGTTCCTTTAGCGTGATAGTCATTTTAGTAATACTTCTCGTAACATGACT
TGTGTTTCCTTGTTGAGAGAACTAGCGTGACATCGTGCACACATTTTTTGGGTGTGTACTGATGGC
CGGAAGTTTTTGTTTATGTCTGTTACTGAAACCTAGCTTTCCTTTGTTATTTTTGCTTAGCATTAT
CTTATGTTGGCTAGTGTGGCTCTTCTGCTCTGTGTTATGTTGTATTCTGTAGGACTATATATTTCA
GCTGTTTGTCCAGTCACTAATTTTATGTTGTTGTGTTAGGCACGGATCAAAAAGATAATGCAAGCA
GATGAGGATGTTGGCAAAATTGCGCTGGCTGTGCCTGTTTTAGTTTGTAAGTTCTGCAAGAATCTT
CGTGTGTCTTGTATCTGTTTTAAAATCTCTATGTTTGGCCCATATCTTCTGTTGCTTTTAAAGATA
TTGCGGCATCACTGTTTTTTGCTGTGTGCATTGTAACACTAGATAATTCCATGATGAAGCATATAA
AGTGAGTATCTGGCATTCTGAATTTATTGTTTGCATGCAGCGAGAGCCCTTGAATTGTTTCTGCAA
GATTTGATTGACCGCTCATACAAAATTACTCTTCAAAGTGGTGCAAAGACACTGAATTCCTTCCAC
CTGTGAGTTCTATTCTGTGTCCATGGCTGTTTAATCTTACTATGACTTCGTGCTGTATGTATTGTA
TCACTCCATGTTTTTCTAGACATATGGATAGCTTTGAGCCGTCTGTGATTTACACATTGTACTTAG
ATGAAAGTTAGTAATATGGTGGTTTCTCCAATGTATTTCCATATATTATAAGGACCCATTCGGAT
GTCCTCAAAATCTACCAAATTCTCAACTGCCGACTCCCTCTGATTCCAGTTCCGCAGGCAAAGCGT
GAACTTTGGCACCATGTACAGCTGAATGGTCTTCATGGGTTGGTAGCCAATTTTGTTAGGACTTGG
CTCGTTAAAAGGGAAAGGAAGGCCAGGGCTTGGCCCGTTCGGTGGGGAAGGATGTCGTCTCTAGCA
CGACCAACAGGGAAAGAAAGACCTCGTCGCACCGCCCATCCCTCGAGGCCGCCCACTGGCGCACCG
TTGTCGAACAGCGCCAGAAGGGCCGCGTTACTGCCGTCCTCTGCGGCTGCGAGCCTTCTCTCCGTT
GGTGGATCTAGCCGGTGGCGGCTGCAATAACGGTGAGTAGGGCGAGGTAGCCCCGTCTGCGATTGT
GGATGGTGAATCCTGCGCCGCCACCATTGTTGTGGGGATGCGAGGCCTAGTCGTGGCCGGCGATTT
GGCTGATGGGAGGGAGAAATAAAAGTGATGAGGGCGAGAGAGACAAGAGAGACAGGGTGGAGACTC
GCTCTGGTTCGGTTTCGCTCACGGGTGAGGAGAGAAATCGAATCGTTTTTCCTATTGGTGGCATGG
CCTTGTAATTTTGAGGAACTCCGTGAGCCACAATTTCGGGAGGTGAAAGCGGAGCTGGCTTCGTGA
AGCTGGGACGTTCGGTCTGGCTCCGTGTTCGGAGTTGGCGGAGTGTGGAGCCGGAGGCCATCCAAA
CAGGCCCTAGGTCTACTTAGCTTGTGGAAACTGTCCAGTTGCTGCTTTGAAGTTTTTCTAGTCTCT
TACATATTACGCCACTAAATACCATTAAATTAAAGTATCTCACAAGCCTAACATGTGTGGTCTAAT
GTTGCTTGGACTTTTGGAGCTTAAATAAACTCTGTAACCTCTTGAAATGCCATCCCTTTGTATGGA
CGACTCTTAGCATGTGCAGTTTGATGTTCTTGGCAAAAGTTTCTGTTTCGTATTTTCATTATTAAC
TTGACTATTATGATTTAAAACAGGGTATTTTTTGTTTTCATTCTTTTAAGAGGTTTCATTACTCAA
ACTGGGGAAGGAAATCAACACCATACATTAATTAGAACACGTAGCCAAGTATATTTTTTGCCTCTT
GGGTCAGATAGCAAACAACAGCTGTGCATCCGTCTCAGTTTCATCTTGAATAACGACGCCTGAGCA
ATCCCTATTCCCACACCCTCCATGGAAGCATCATCTGTAGTTCCCTTCATCATCCGTAATTCTTTC
ATGGCTGCTGCTGTCAAACTCCGCACCTTGTTTGGCTTCAACAGCTCTCCGGACTCTAATGTGACA
GCGAGTCTGGTGGCTAGATGAATTAACTGAGTTCTGCTGGTCGTCATCTCGTAGTGTATTCCTGTT
CTCCCACCATAGCCACCAAAACACAATTACTAGCAATTAGTGCTCCCCCGGAAGGCTCCAAAGAGG
AACCAGAATATGGAAAGCCGATGAGTACAAGAAAAACATTGGGAAATTATTGCCAAGGCTCAAGAT
GTATCAGCAGGTCAGTTTCGGACTGGTTCATCTTGGTAACCAATTGGAAACTGGTTTATGTTTTTC
AGTGGAAAACTACTCTGGTATGATTTCCAGTAATGAACTATAGGAGATTTGCGAAGATGGTCTGCC
TTCCGAATGGAACTTGAGTGGAAGCTGAGATGTGCCTGCTCATATGCAGCACATGTGTTTGTCTGA
CTTATCATGATTTATAACTTTCATCTGTAGTTCGAGCTACTAATTTGATTATAATCTAAGATTAAT
GATGAAGACTTATCTTCTTTTGATACCTTTGGTAATTGTGGTCATCAATGGTGTTTTCTTTTGTCA
AAAATTTAGTTACACAAGTCAACCACAGCCACTTATGCGATGTTTAAACTGTGTGCCTTTGCCTTT
AGTGACTAATGGTTTATATGTTTCTTATGAGATGCTCAGAAAGCAATGTGTGAAGAGGTACAGCTC
TTTTGACTTCCTAACTGAGATTGTCAACAAGGTGCCGGATCTTGGTGGCGGTGAATCTTGTGGAGA
TGAAAGAGGATTACCCAGAAGAAGGTAAATTGACTTGGCATAGTCAAATTCTCATGAATCATTGCG
ATAAGTAACGAGCATGTTATTAATGATTGGAATTCACTTTCTAGGAAATTTTCAAATGGAAGCGAC
CCAGAGAATGAGGAGCCCCGATCTAGCAAAATGGTACAATTCCATCACATGTCAGCAGAGTTAAAT

TTTCTTAGCAGATAAGTTGATAACATGATTGGCTTTGCAGCCCATAAGAAGCTTGAACACTAGTCC
CAGAGGACGAGGCAGAGGTCGAGGAAGAGGGCGAGGGCGGCCTCCAACCAAGAGAAAGGAAATTGG
TTATGTACAGTTTGAGGATGAGAGCAGCATGTTTGCTGAACAAAGTGAACCCTTGCCAGGAGATGA
GATAGTTCCAGAGACCAACCGTGGCAATGAGAGTATTCCCCAAAGCTCACATCCTCTAGTGGAGGC
TCCATCAGCCGCGACGCCAGCTGTGATTTCAAAGGTTGAAAAAGCTAGCACCAACCATCAGCCAGA
TTTGCCTATGCCAGATGCCATTGGAGGCATTGGTGTTGGACCATCCAGTTTTGGACATCTGACGGT
GCAGGTTGATGAGGAAGAGGACTACGATAATGAGGATTAGGCATAGTCATCCTCTCATTCATCATA
TGCTCTAACAGGACAGTTCTCCTGGTGTTGTACATTGTAAATATTGTTTCAAGTAGTTACCGCAGC
TATGATGTGTAACCTATTTCTTTTTCCCAATAATTGGTTTCGAGTTGCCTCGCACTGTTGACTTAA
CTGACTAGGATGTGCTAAATTTAACCCTGTATTTGACGGTTGAAGCAGTGATATTCTGTCGTCCTG
TTACATTGTACATGTTGTAACCAGCAGTAATTATTTCTAGTATATCATGTCGTGACCATCATGCCA
CGAAATCACGTCATTCAGATGTAGCCCAGCAAGCAATGTGTGTACGAGAGATATCTACCTTGACCT
TTACGTTTTGGAGGTGGATTTAGGGCAAAACACAAGGAACAAGGAGAAACCAAGAGAAATCTAGAG
GAAAACACTCGAGAGGACGTGATTCACACCAACTACCCTCAAATCAAAGGTCAATACAGGTACATT
TTGCATCCAAGAACGACACTAGAAATAAAGGTAACCAAAGGGATAGTTTTTCCTCAAATCTCTAGG
AGAGATGGTTCGTCCTTAATCTGAAAGGATTCTTCCAAAAGGATTTTTCCCGTTAGGTCTTCATCC
TCTATGAGGAAATAAGATGAGCAAAACTCCCTCAAGATCTAGTTTATGCTTTGCTAAGCCTAGAAA
GTGCAAGAACTACGAAGAGTGGAACCCTAGATGGCCTAATCTTTCACATCTTGGAGGGGGAATCCA
AGAGAAACACACAAATCACAAGATGGAACACTCAAAGACAAGTCCAAATCACACATCCACTAGATG
GGCAACACATGAGATCCATAAGCATACAAAAAAGTCCAAGGAAAATAGCAAGAAGAGTAAGGTCCA
TCTCAAATCCATGGAGGACATGAGATCTTGGAGGTAGATAGTTCTTCCCAAAACTCTAGGAGAGAT
GGAGGCTTGAATCCACGAGAGGATATTCCAATAAAGGGCATTGATATCCACTTGGGATCTTGATGC
GGAGCATCCCACAGTCATCCCCATGGACCTACCTTCGTCTCCCCAAGTGCCACGTGGACCGACGAC
ACGAGCGCGTGCAAGAGCTATCGAGACCGAGGTGACATCACTTCTCGCTCAACTCCCTTTCGAATC
ACATGAGACATGGCTACTACCTCAAACGGAGAAACTTTGCATACTCAAGTACCAAGGAACTAGGCA
TGGAGAAGCTAAGGAGCAAGGCCGAACGATGGAGGGAGAATAACGCGAAGATAGAGAAGAAACGGA
GCAAGACAGAGCTTGCCCGGATGATCCGGACCCCCATCCGGACGATCCATGTAATGTGCCCGAAGA
GTACATCAAGCCTCCGGACGAAGCCAGATCATACAGACCCATCCGGACATCCGGACGACGACCCGG
ACATTCAGACCAACTACGGCTGCGACGACACAGCCGGACGATCCGGACGGCCACCCGGATCATCCG
GACCCCGACCCCCCGATCATCTGGACAGTGCCTGCGTGCATGGCTTGGGCTGAGGCCCATGTACCT
CTCTATACCCCTAGACTATATATACTCCTCCCCGCCCATGTTTTAGGGTTAGCAAAGGATTAGCTC
AAGATAGAGATTGAGCTTTGCTCATCCACTTACCTCTCCCATGGAGATCAAGGCCTGCATGTGAGA
AGATCCCCAAGTGGATTCAAGACCCCTTCATGGGAAGATCCTTCTAGGATTGAAAGACCTCTCTCC
TTCAAGAATTGGGATGAACTAGCTACCCTTTGTTACCTACTTGTGTTGGATTTGGATCCTTGTATC
TCCTCTTTGTGTATGTGGATTTAGCACATGTGTGATTGGATGTATCAATTGAGTGTCCTTCTCTTG
ATTTGCTCCTTTCTCCACCTCAAGTGTGAAAAGATCATGAACTAGGGTTTCACCCTACATCATCTT
GGATCAGAGCATAGGTTGATTCACATCTTGGAGTCCATACCCCCATTTGCTAGCCTAATTTTGTGT
GCTTTTCCTCAATTTCGAAAATCCCCACAAAAAGAGCCATACCATTTTTTTTGTGATTTGTTGGTT
TGATGATGTTTTGTTGATTTTGATCCGTGGATTCATTGTGTTGCAAGTGGATCTAGCATTCCCCGA
CTCCTCCACCATGAAATCCACCCAAAATCGCCCCATGTTTTATCCATGATTTGGAGTTCTTCCCGA
CCGCATAGCCCTGCCCGGAT

**>*TaDrAp1-D4*-CDS-768bp**
ATGAGGAAGAAGCTGGGCACCCGGTTCCCCGCGGCACGGATCAAAAAGATAATGCAAGCAGATGAG
GATGTTGGCAAAATTGCGCTGGCTGTGCCTGTTTTAGTTTCGAGAGCCCTTGAATTGTTTCTGCAA
GATTTGATTGACCGCTCATACAAAATTACTCTTCAAAGTGGTGCAAAGACACTGAATTCCTTCCAC
CTAAAGCAATGTGTGAAGAGGTACAGCTCTTTTGACTTCCTAACTGAGATTGTCAACAAGGTGCCG
GATCTTGGTGGCGGTGAATCTTGTGGAGATGAAAGAGGATTACCCAGAAGAAGGAAATTTTCAAAT
GGAAGCGACCCAGAGAATGAGGAGCCCCGATCTAGCAAAATGCCCATAAGAAGCTTGAACACTAGT
CCCAGAGGACGAGGCAGAGGTCGAGGAAGAGGGCGAGGGCGGCCTCCAACCAAGAGAAAGGAAATT

GGTTATGTACAGTTTGAGGATGAGAGCAGCATGTTTGCTGAACAAAGTGAACCCTTGCCAGGAGAT
GAGATAGTTCCAGAGACCAACCGTGGCAATGAGAGTATTCCCCAAAGCTCACATCCTCTAGTGGAG
GCTCCATCAGCCGCGACGCCAGCTGTGATTTCAAAGGTTGAAAAAGCTAGCACCAACCATCAGCCA
GATTTGCCTATGCCAGATGCCATTGGAGGCATTGGTGTTGGACCATCCAGTTTTGGACATCTGACG
GTGCAGGTTGATGAGGAAGAGGACTACGATAATGAGGATTAG

**>TaDrAp1-D4-Pep-255aa**
MRKKLGTRFPAARIKKIMQADEDVGKIALAVPVLVSRALELFLQDLIDRSYKITLQSGAKTLNSFH
LKQCVKRYSSFDFLTEIVNKVPDLGGGESCGDERGLPRRRKFSNGSDPENEEPRSSKMPIRSLNTS
PRGRGRGRGRGRGRPPTKRKEIGYVQFEDESSMFAEQSEPLPGDEIVPETNRGNESIPQSSHPLVE
APSAATPAVISKVEKASTNHQPDLPMPDAIGGIGVGPSSFGHLTVQVDEEEDYDNED*

## ***TaDrAp2-A1*** **(**TraesCS1A02G310700)

**TraesCS1A02G310700** **<u>FORWARD STRAND</u>**
pep chromosome:IWGSC:1A:501701967:501707884:1 gene:TraesCS1A02G310700
transcript:TraesCS1A02G310700.1 gene_biotype:protein_coding
transcript_biotype:protein_coding

***>TaDrAp2-A1***
AGGAGGTTCGCCGGTGTCGCGAGGCCTTCACTCCAGTCTCCGGAGCAGCGGCGGATGCGCGCCTAC
GCGCCAGAATCTCCCAACTGGTCGCGCTGGTTCGCGCTAGAGCACGAGGAGAAGCGCCGCCAGGGC
GTCCACGTCGACCACGACCTTCCTCCTCCGCCACCTGAGGTTCTGCTGCACGAGCAGGACGAAGAG
GCGGCATACCAAGCCGAGCAAGCCCTGCAGCACGCGTTGGAGGCGAGCAGGATCGAGGAGGAGGCC
TAATGGGATGGGATGGAGCGCGCCCTCGCATTGTCGGTGGCAGGAGAGTTCGTCCACGCCCCGCTC
TTCGACTCGCCTCCACCGCCACCGGCGCTACCCATCGTCGAGCCCAAGCCAGAGCCGGAGCCGACG
CGAAAGTGCTCGCCGCCTCCACCCACTTGGCCGGAGGAGTCCTACATGTGGACCGACGAGTACCGT
GAGTGGATGAGCACGCCTCCGGTCCACTACGCCGCGAGGCCGGAGGAGGAGGCGGCCCACCTTGAG
CTCTGGAAGGCGCATTGGCTCGCCTAGGAGGAGGCCGACGGCGAGCGGCAGATGCTTTGGGAGCAG
CAGCTATGCCGCGATGCGGAGGCGTTGCGGCTTGAGGAGGAGAAAGAAGAGTGCGCCCAGCTAGCC
ACAATGTCGGCGCCCCAGCAGACGCCGGAGGAGGCTGCCTTGGCAGTGTACCAAGCGGCGTTTGGG
TGGGCTGGCCCTGCTCCCGTCTTTATCGACCTCACCGGCGGCGATAGCGATGTCAAGGGCAAGGGC
AAGGCGGAGGACGTCTAGGGCAGCGTGCGGGCGCATATCTTTTTATTAATGTTTTTAATTAGGTTT
AAGTGGACTTTGGCCGGCGGTTGACCAACCACTTTAATGTTTAATTATGTTTATTTCTATGACCTG
TTTTTTTCTACACCGGCAAATTTGGGCCCGTCTTTCATTGGGCGCACCTGCCGACCAATATGAAAA
TGCCGACACGCACGTCTGTCTGGCCGATCCAAACAGATGAAAAGCGAATAAAACGCACGTCCGATT
GGGTCGGCGCGTTGGAGGTGCTCTTACACCCTGAAACATGATTCCCTTTACGTTTACGTTAACACT
CCACAAAACAAACATGGCAATGTATTCTTAGCTCACAAAGACAAGTCAAAGCATCTCCCTCCGTTT
CCTTTTTTACTCTACATATACACTTTGATCAAAGTCAAACTAAACAATGTTTCAACAATTTTATAT
AAAAAAATATTAATATCTACAAATATCAAAGTTATATGGTATATGAAAGTTAATTTTATGATGCAT
CTAATAATATTGATTTCATATTGTGAATATTGCTATTGTTACCAATAAAATTAGTCAAACATTATA
AAGTTCGACTTTGACTTTTTTTTGAGCAAATGAACAATTTTTTTAAGACAAACCAACTTTATATGC
AAACTAAAAAGAAAACGGAGGAAGTACCCAGATAAAGCTAGAGCCCAAGAATGGACGATGACTCTC
GCAGCCCAAGGCCACGAACGAGACACAGCCGCCGGATCCCGCCGACCCCTCGAATCGGACGGCGGA
CACAGGGAGCGCCACGAACCAGCCAGAAACATGGGGCCCACCTCATCCGGGGAACGCGACACGGAC
GGCTGACCTGCGGCCGCACGCGCAAACGGTTCGGGAGAGGTCTAACTACGCGGGCCCCCACATCGC
GCTGAGCTTTTACCCACCGCGAAACGTCTCCGTGGACCCGGTCCGTCGCATCCACAGCATGGACCA
TCTACTACGGTCTTAGCGCCGCACGTGAGGCCAAGGCCTATGCGCCGGGTCCAGGAGCTTAGCGTC
CATGCGCCGGAGAGTCCGCGCACCTCTGCCCGCTTCGCGCGGCCATTGGCCGCGCTGTTTATGGGG
GAAAAAATAGTTTGTTTTTATTTTCTCCCGTAAAAAAAGTGAAAAGATAGCGATAACGGAAACCCT
AATCCCCAAATCAGAAATCTATCCGAATCGCTATTTATGTTTGTGGCTTTCTCCCCCCTGCGATCC
GATGTCCCCCGCTTCGCCCGCCGCGCCGATCCGACCTCCCGCCGCCGCGTTCTCTCCGCCGGGGAC
GCTCTCTCCGGCGCCGCCGGCTCCGCGCCATGAGGAAGAAGCTCGACACCCGCTTCCCCGCGGTAT
GACCCGTGCCCCTGCGATTTTTTTTTGCCGGTTCAGGCCTCCCCCCCCACGCTGTGCGCGCCGTCG
ATTTCGCGATTTTTTCTGTGTGCTTCGTCTAGGTTTTGTTTCCGGCGGTTAGGTTTCTGTAAATTG
ACGGCCAATGGACCTGCGATATGCATGTTTGTCTGTTTCTCTCTATTGCTGGGCTTGATAGATGCA
CGTTTGGGGAATTTTTGGGTGCTGTCGCTAAATAATATGTAGATTTCGCAGCACGGTTGGAATTTT
TAGCCTCTCGCGTGTGCTCGTCCTGGGCTTGTTTCATGTACGTTTGTTTTTATGGTGGCTATGGTT
CGCTGTCCCGATGGCAATGAAGCTGCGGTTATAGATGGGCATATGATGTAGGTGGTGGATGCGTGA
AAAATCCTGGATTATGTAGATTCTGCCTACCCTGTTAACGTTTCACTAGTATATGGCTTTCCGCGG
ACGAGTGTGCTATTAATCTACTTTATTATTTAATATTAGGGTCAATTTATTTTATGTTTGACTAAT
TCGTTATGATTAAATCCTTGGGTCATCCTTGTGCTTCAATTGCTAGGCTTGTTCTTTAAGGAACGA

AGTTAACAGAAATTACTTGCATTCACTATTTACTGAAAAAAGATGGACATGATTATTAAATAACTC
TTTTATAAATATGAGCTTTTTTTAGGAATGAGAGTTTAACTTAAGCGGATGTGGCTTAATTGTCTC
AAGAGATGATCAACTCCAGGTCTTCTGCGGTGTTCCCTATTTTGATCGGTAGCTTGCTTCAAACCG
GGCCGATTGTACCTAAACTAATATTCCCACATAATGAACACCAAGTGCTCCTTCCTTTTTTCCCAA
ATTAAAAGAGGAGCGTGATTGTTGGTACAAAGTGCTAACTATAGAATCAAGCATGTGAGTTCTTTT
TATAGTTGTTGATCTGTTGACATTGGTATCATGATTGTTTAGATATAGATTGTGGTGGCAACCTGG
CAGGGAAGCTTCTTAGCCTGATGAGTTAGTACTGTTCAGATTTTGTAGTATATTTCAAATGGTAAA
TACAGTAGGATTCTACTATTTAACATCTGGCCCACAACAGGCAATGAGTGTTGGTTAAAGCTGTGT
ACGATCTATAAAAATATATAGTGTAGAGTGTTTATCTTATTCTAAGCATGGTATATTCTCCGATCT
GTTTTTGGTCCATCTTCGGGGGAAGATGCAAGTGTTTTGATGACATATGTTTGCTGTACAATGGAA
TCCTCATGATACTAAAGTTTTTCTACAGTGGATAGAGATACCTTCTTCATTACCCTTTCTTTCACA
AGGGCTAGCAGAATCTCTTTTCTTCATCTGCTAGGACCTGTGCTTATAACCACATGTGAATACTAT
CGCTCTCATGTCCTTTTCCATCGAAGTTGCATCATTGGCCATTTGCACTGTAGTATGCAACAATCA
TAACTATGATTAATGAGTGTTTTATTATTGACATGTTGTCATAATTTGAAAATGGCAGATTCCTAC
TACTTTTCAGATGGTATCATGGTACTCTTCTGTTAATGTGATATCAGCTTCCTCTTGTACTTTGTT
ATACAGCCTCGGATTAAGAAGATCATGCAAGCAGATGAAGATGTCGGCAAGATTGCTCTAGCTGTA
CCTGTTCTAGTTTGTAAGTTTTCCACAGCCTCTTGTTTGCAGAACTTTCCTTCAACTCGCCTCTTA
TCAGTTCCAGAGTTATTTCACTACATACATCATCTTTTTTTCGCCATCTGTTGTTAACGAGAGAGT
TCGATAGCATGTCTTGAAGAGGCCATAGACATATGCTAAATGTAGTTGGCAAAATAGCTCCATTGA
AGGGCTCCAGAAATGGCAGCGTTGACCACTGCAATAGGAATTTCATCTTTCTTTTTATTTTCTCAT
ATTCAAACCGAAGCTAAAAGTAATTGCAGACATTAGTATCTATTGTTCCTTCTTGGTGTAAACAAA
ACATAGGCAAAATTATTGAACTATTTGGTCCTTTCGGGAAGTTGACAGGGATAGATCCTCTGAATT
TATCTTTACAGAGTTTTGTATTGTTTGGAGCATGACACGTTTGCAGCTGCCGGTCAGTCAGCACTA
CACTTTGATTCAGATGACATGATTTGATATATGGTCATTCTAGATCTCGATTTCAAACTAGAGAAT
CATACTGACCATAAAGCATATAGGGAAGATGTAGAGCAACGGTAGTCATCTTTCTTGTTAAGAGGA
GGAGCTTGCTACTGAGGTCATCAGAGCTAAGGTGCAAATGTTAATCCTTTGGGCCAGGGAAACTGA
GTTCAGGCTCAATGGTCAACCATGTAAGCCTATTCTCCTTATTAATGCATGCATCCACAGTTGCCT
TTACACCTCCCCCACAGGTGCGTCACCACCATATTTCCATGCCAGAGTGACCCCTGACTGGCTCCT
CCACCAGGCATCTCATCTCTACCCTGTTAGCTCAGCGATATGCTGCCAACATGTCTTGATGATTTG
GCTTTCTCTGTTCTCCTGATTTGTAGCTAGTCAATTTATTTTACATGTCCTGGTGGGTCAAACTGG
GTAGAGGAGAAGATGGGCACAAGTTAGGTGGGGAGCCATGGTGCGGGGAAAGGGAAATCTTGGAGA
GGGCTAGACATGGGAACAACTTGTGTATACATTGCACAGTATTAAGGACCACAAAATTGAGTGGAG
TACTTAGGCGTTAGGCGTACTCGATGACTATGGCAGGANNNNNNNNNNNNNNNNNNNNNNNNNNNNNNNN
NNNNNNNNNNNNNNNNNNNNNNNNNNNNNNNNNNNNNNNNNNNNNNNNNNNNNNNNNNNNNNNNNNNNNN
NNNNNNNNNNNNNNNNNNNNNNNNNNNNNNNNNNNNNNNNNNNNNNNNNNNNNNNNNNNNNNNNNNNNNN
NNNNNNNNNNNNNNNNNNNNNNNNNNNNNNNNNNNNNNNNNNNNNNNNNNNNNNNNNNNNNNNNNNNNNN
NNNNNNNNNNNNNNNNNNNNNNNNNNNNNNNNNNNNNNNNNNNNNNNNNNNNNNNNNNNNNNNNNNNNN
NNNNNNNNNNNNNNNNNNNNNNNNNNNNNNNNNNNNNNNNNNNNNNNNNNNNNTTTAGCCGCGGTAAGA
GTTGATGTGTACCTGCTGTTTGCTATATAGGACCATACATTGAGTGAGTAGAGTAATTAAAGATAC
TTGAGAATTGGCACTCTAGCGGATCATACTTCGAATGTAAGCCATTCCATTTTAGACGCATGTACA
AAAATGCCGGTTCATCAGTAGAAAGCAAACCCCTACTTTTATAAGCAGTATAGATAACATCAACAG
CAAGGTATAGCCTAATTGCATGTGCTTATTTGAACTGTATAATTAATTAATGTTAACATGTACAAT
GTTGATATATGATTGACGCAAGCTATATGCGATAGCAAAGCAACTCGGCCTCTTATAGTGCACCTG
CATGCTTATATTTTCTTAGTTTCTTTTTGTAACTCACATCATGCATGTGCTTTCGACTAACATCTT
TTTCTGATATCATGATTTGCAGCCAAAGCGTTGGAGCTATTTTTGCAAGACTTATGTGATAGGACA
TATAATATCACTGTTGAAAAAGGGGTCAAAACAGTGAGTTCATCACATCTGTAAGTTTCCTTGACC
TTATATGTTTTGTCATATCCAAACACCTTTTTGTTTGACTGGATAGATAATTTATATTACCATGTT
GAACTTTTTGATTAGTATGGCAAAACCTTGGAAGCATTACCATTTCTCATTTTGATTTTTCTTGGC
AATACATTGTTCTTGCAATTTTGTACCCATAAATTGTTTCCTCAAGTTCAGTTCTACTGTCATATT
CTGTCGCTTGTGTAGTTTATGTAGTGTCCAAATATACCAATGAGCAAGTTGCTCAGTAACACTACA

AATATCACATGGGAAAATAACATCGTTTCACTGATGACATGCCCTCAGGGTCCACCTTTCATTGTC
ACATTGGCTGTTATTTTTCAATTTTCAGCTTTACTAAACAAATTCAGTTGTATAACAAACATCAGA
AGTGAAAAGCTTTGACGTACTGATATTGCTCAAGTACTCTTTCACAATGACATTTTCAGGAACAAA
TATAGGCTAGCTCCTTTTGAACTGAATTTAATGTCATAGTTGGTTAGCTTATATGTCAATCCTATT
TAGTAACTGATTGGGCCCTGGTGCTACTTTGTGCCAAAGTCAGCAGCGTGTTATATGGATTATGGA
AGCTGTAAAAGTACCTTCCTTGATACATGATTTGAACTGAAAGAGAGAGGTAGATAAGCATGTGTC
TTGTGGTGGTCTATGCTGATTTTGGGGTATTACCGTCTTGGTGGGGGTCCTCAGGGACATGCAGTT
GGGTGGTGGTTGCATCATTTCAATTTGTGGTTGAAATGGGACTTATTTTCTTGATCCTGATATATG
TATTATTTCCTTGAGCAAAACATTTGTTTTTTTTTTCTGATGGTCCCATTATCTCATTCAGTATTA
TGCTATTACAGGAAACAGTGCATTCATAGTTACGATGTGTATGATTTTTTGAAGAACGTTGCCAGC
AAAGTTCCAGATTTGGGTGCACCTGATTCTAGTGTTGATGATAAGCTTGGCAAAAGAAGGTAAGGT
TTCAAATTGTGGTTAATTAGAGTCTGATATGAACTTTAATAATTGGTTAATTTGAGTTTATATGAA
CTTTAATAATTGCTGAAATGTCATCAGGAAACATGGTGAGGATGAAAGCGAGGAGGAATCGAAGCG
GACAAGAAATGTAAGTCTTTCTGACCTGTACCTTTTCTGTATATGTTACACTTACATGTGCATTTT
TGTGAACAGTTTTTAGTATGAGCTCAGGGTTTTCATTTTCTCTGCTATTCAGCCTGTAGCTTGTTT
CGTATGACACAATATATGATCACCCCATTTTATGTCGCCCATTCGACCTCCTTTTGCTGATAAAAG
TTTTGCATATGTAGGAGGCAGCATGCCACACAAGCAACGGCAGAGGGCGTGGGAGGGGACGTGGGA
GAGGTCGCCGTGCTGGGCGTGGAGCTGAGAGGGAGATTGAACACCATGAATTAGGTTGTGCCCAAT
TTAGCAAACCAGGCCACCTGAAGGTAGAGATTGGAGACGGGGTTTTGGACACAAGTGAAACCAAAG
AGCACACCCCTTTGAGCAATGCCAGGGCCTCCTTAAGGAACATTGACTTGAACTTAGATCTTGCTG
ACTACGAGGAGGATACAGTGGCGCCGCAAGTCCAGCCTTCAGCTCCTGTGGTGACCGTGGCGCCGC
AAGTCCAGACTTCAGGACCATCGGTTTCACAGTTGAAAGAAGAAGTGGCGACCAAGGATTTCCTGG
GCTGGCAAATGCCTGAGATGAACAAGATGGCCATGGACCCGGTGCAGTTTGCGCTGTCGTCAAACC
ATAGATTAGAGGAGGACGAAGACTATGACAATGAAGAATGATGTGACTCTAGCGTGGATTTAGTTT
GAGAAGCTGATAGGTAGACATGACATGATGTACATCCTAGCAGGCCCAGCTTGACAATGTAGTAGC
AATTCTCTTGTAGTATGTGTACCCGCAGATGCTGCAGAAGTAATTTAGGATGTGACATATGTTGAT
TGCTCAATAGCCAGTAGTAGCTAGTATCTTGTGTAGCAACTATATAGTGTAGCGACTATATAACTA
GACATGTTCGCCGGTTGTAAATCATGGTTGCTTTTAGATCAAATGCTATGTCATAATGCTTATGTT
AGCTCTTATGAGGTTCCACTCTTCCAGGTGGATTTTTTCTTGTGTTATTTGTGAATTTTTCTCTTC
CAGGTGGGATTTTTATCTTGCATTAGGGATTTAACAATCTTGTTATTTGTGAAGTTTGCTCTTCCA
GGTGGGATTTTTCTCTTGCTTTAGTGATTAACAATCTTGTGTTATTATTATTTTTTGAAACGGACA
ATCTTGTGTTATTTGTGATGCAAGACATCAAGCTTTGGAGATATGTGGGAGGTGAAGTACAAACAA
GCAAAATACTTGTGGGAATCATACACATCAGGCAGGAAGCAGGTTTATTTCATCATATTACACCAG
TATCCAGTGAGGCTGACATGTTGGATAGGCCCCAGCCACAAGCTTCAGCAGTTGAGCTCACATAAC
ATATTTATTTACATCTTTTCAGGCGGCCGGCTTGAAGTCCTGGAGCTGCTTGAAGTTCTCCCAGGC
CTTCTCCCACACCTTGGCCTCGTAGAGCTTCTTGGCGCCGCCCTCCTTGACCTCGATGGTGAAGTA
GTGCATGCACCCGGCCACCACCTGCTGCCTCACCTTCACCAGCCTCTCGAACTCCAGCAGCGCGTT
CTGCGCCCAGCAAAATTCACCACCGATCCAGCAGGTTAATCAGTTACAGTTAATCGTATCGGACGC
CAATTGGCTTGGGATCGGGTCGGGTGGGGATGATCTTACGGCCTTGGTGTTGTGCTCGGCGACGGC
GAAGCGGGCGAGCTCGATGGTTGCGAGGTCGTTCTCCCGCCCCGCCGGCGCGTCCTGGACGCCGCC
CAGCAGCACGCCGCGGCCGCGCAGCCCCCCGCCCTGCGCCGCCTCGGCCATCGCCGTGCGTCCGGA
GGGTGCGCTGAGACGAGAGAGGAGTGTACGACGGATGGCCGCTGCTGAGAGTCTGAGAGAGAACAG
ATCTGTTCCGTGAGCCGAGACGATGTTGATCGAGGGAAGTGGGGGACGGGACTTTATAGCGACGTG
GGGATAGAGGGATTCCGTTTTTGTGCGCGACGTGTGATCTGCGGGGGCTCGAGCGCGACAGCTATA
GGCGACGGACGGGCCGGACGCTCTACGCAGCAGCGCTGGCTGTGCGATGGCGACGGCGATGGCGAT
GGATGCGTTGCAATGCTTAGCTCCGAAGGCAAGTGCTGGCGAAGTAAAGAGCAGCAGACCTGGTGA
AACGGTCGTATATCTATCCCTGAAAAACAATATACACGATTCCCAAAAGAACTGCTTTATTCGGTT
TGCTACTCCTGCATTTTTTAGGTCATTAATCTGAATTTATGTCTCCTCTGCCACCAAAGTAAGTAC
TCCAATTACTTTACTAAGAAAATGTAATCACTTGTGATTTGGTACCACCGAAATAGTCGCGCGCGA
AAGGGCAAAGAGGGCAGTGGGGAAATTCTCTAATGCCTTCATTTCAGTGAGAGAGGTCTGTCCTGT

CAGAGTAAATGATCACGGGTAGCCTCATGAGTCTCCGATGGTGTTTCAATACATCGGGGCTGGCTG
CGCCCCTCAAGAATCAAAGACCAAAACATCGCCTTCTTTGCGGTCGGCTGCCAAAAGACGGCAAAG
CTCAGAAGACGGCCCTGAGGTGGGCCGGCTCCTAGTAGGCGGCCTCCAAAGAGGCATGCTCCTAGT
AGGCGGCCCCAATCACCCTCAAAGTTTGTGCCACATTATAACAACGAGACAGGGTGTGGCTACAGC
GATGCCTGTCACCTTCGAATCCAGGGCGGAGCGTGGCCACAGTGCGGCGTACTGGGCGGACATCCC
TCGCTCGGCGTGACACTGTTGCCACGCAGCCCATGACATCATCATGGCAGAGAAGGCCATGCTCCC
ACTACGAGCTGTCGGTACTGCCTCAGACGGCGGGCCTTACCTGACAGTGAGAAGCCAGAGGGCGGC
GAGCTCGGACCAGTCGGCATGAGGGGAGGCCGACTCCTGGCAGGCGGCCGTCTTCTTCCCTCAGAG
TTTGCCATTAACCAGAAG

**>*TaDrAp2-A1*-CDS-852bp**
ATGAGGAAGAAGCTCGACACCCGCTTCCCCGCGCCTCGGATTAAGAAGATCATGCAAGCAGATGAA
GATGTCGGCAAGATTGCTCTAGCTGTACCTGTTCTAGTTTCCAAAGCGTTGGAGCTATTTTTGCAA
GACTTATGTGATAGGACATATAATATCACTGTTGAAAAAGGGGTCAAAACAGTGAGTTCATCACAT
CTGAAACAGTGCATTCATAGTTACGATGTGTATGATTTTTTGAAGAACGTTGCCAGCAAAGTTCCA
GATTTGGGTGCACCTGATTCTAGTGTTGATGATAAGCTTGGCAAAAGAAGGAAACATGGTGAGGAT
GAAAGCGAGGAGGAATCGAAGCGGACAAGAAATGAGGCAGCATGCCACACAAGCAACGGCAGAGGG
CGTGGGAGGGGACGTGGGAGAGGTCGCCGTGCTGGGCGTGGAGCTGAGAGGGAGATTGAACACCAT
GAATTAGGTTGTGCCCAATTTAGCAAACCAGGCCACCTGAAGGTAGAGATTGGAGACGGGGTTTTG
GACACAAGTGAAACCAAAGAGCACACCCCTTTGAGCAATGCCAGGGCCTCCTTAAGGAACATTGAC
TTGAACTTAGATCTTGCTGACTACGAGGAGGATACAGTGGCGCCGCAAGTCCAGCCTTCAGCTCCT
GTGGTGACCGTGGCGCCGCAAGTCCAGACTTCAGGACCATCGGTTTCACAGTTGAAAGAAGAAGTG
GCGACCAAGGATTTCCTGGGCTGGCAAATGCCTGAGATGAACAAGATGGCCATGGACCCGGTGCAG
TTTGCGCTGTCGTCAAACCATAGATTAGAGGAGGACGAAGACTATGACAATGAAGAATGA

**>TaDrAp2-A1-pep-283aa**
MRKKLDTRFPAPRIKKIMQADEDVGKIALAVPVLVSKALELFLQDLCDRTYNITVEKGVKTVSSSH
LKQCIHSYDVYDFLKNVASKVPDLGAPDSSVDDKLGKRRKHGEDESEEESKRTRNEAACHTSNGRG
RGRGRGRGRRAGRGAERE IEHHELGCAQFSKPGHLKVEIGDGVLDTSETKEHTPLSNARASLRNID
LNLDLADYEEDTVAPQVQPSAPVVTVAPQVQTSGPSVSQLKEEVATKDFLGWQMPEMNKMAMDPVQ
FALSSNHRLEEDEDYDNEE*

## ***TaDrAp2-B1*** (TraesCS1B02G322000)

**TraesCS1B02G322000** **FORWARD STRAND**
pep chromosome:IWGSC:1B:546528685:546534227:1 gene:TraesCS1B02G322000
transcript:TraesCS1B02G322000.1 gene_biotype:protein_coding
transcript_biotype:protein_coding

***>TaDrAp2-B1***
TGAGACGCGGTGAGGCTACAATGATCTCCCACGAGACTGCGGGACTGTAGCCACACCCCTCCCCCG
ATCAAGCATGCGTCATTAACATTGCGGCTACAATAACCAGCCAACCAGACCCGCCAGCGGCGGAGG
CGGCCTGTCGGCTTCGTACCAGACCAGTCGACGGGGCTCACCAGGCTGCGGGCCCCAGCAGTCGGC
AGAGAAGCCGGCGACCGGAGGCACTGCCAACTGGGCCCTACACCCGGTCAGATTACCAATGTACCC
CTGAGGGGTAGGCCTATATAAACCCCACCAGGGCACCCATGCAAAGGGTGCCAGGCTGGTTAGAAC
TAGACTCATACATAGAGGGAGAAGAGAGCAAGCCCTGCCTCCTTCTACCTTCAGCATACAGCTCGA
GGAGCACCATTGTATTCACTAGTGTCTTAGTGATCATGCGGAGACCCCGCAGAGCAGGACTAAGGG
TGTTATCTCCTAGGAGAGCCCCAAACCTGGGTAAAGTGCGCCGACTTTCGTGTCTACGCCTCATCC
CGCTTCCAGGCACCGGCGACGTTCTACTCGCTCCCACCATGATAAGCCATCCTTTGGCATATGTCG
CACCCAACCCCCAACATGTCGCATGGATCTGACGCTGGGTGTGTTGTCACTGCCTTTGGGGTGCCA
CCCGTCTGGCATCTGAAGCATGGCGCGGCGTGCCTGTTGTACGGGGCAACAAGCGCGAGAGGCGGT
GGCGACCCTCATGGCGGTGGATGTCGGCGCAGTGGAGTTGCATTCTCCGATGCCCCGTAAGGCACG
ACAGTCCGGGCGCCACAACTGTGTCGTGGTGGATGTCGCTGGCTCGTCCTGGGATGGATCCATCGT
TGATATGACGTCCGAGTTCCGACGAGGAAGAGTAGGACCTAGGAGACGGCGGCGCCTTGAGTCCCG
TGAGTCGACTCGTGTTCCATGCCCTACTCTACCTTTCAGGCGACCGAACCAACACTCTGAGAAGCG
CAGCCGACCGCCGGACGTAGGCACGACGGAGGCGGACGAGCTCCGCTTAAAGATCGTTTTACGTTG
CATGTAATAATATGAATTTGAGGTTTCTACTTTTAGGTATCCGGTTATAAAATCAAACTTTTAAGA
CGTGATAGGTCATTGTCTGCGGACACGCCCAGACTTGTCTGCGGACATTTGAGGGGGCGGGTTTGC
GAAGTACGACTGTATATCCTCTTACACCCTGAAACATGATCCCTTTACGTTTAGTTAACACTCCTC
AAAACAAAACACGACAAGATATGCCGACTCAGTCTTTCAGATGCGCTTATAGGAATAGAAAATGCG
TGTGTGCGTTTATAGGGATAAGCGTATGCGCGTGTATATGAGCGCTTGCGTCTGTACCGTGTTTAA
AAAAACACACGGCAATCTATTCTGATCTCACAAAGACAAGTCGGAACATGCTATACCCAGATAAAG
CGAGAGCCCGAGAATGGACGATGACTCTCGCAGCCCAAGGCCACGAACGAGGCACAGCGCCACAGC
CACCGGATCCCGCCGACCCCCCGAATCGGACGGCGGACACAGGGAGCGCCACGAACCGGCCAGAAA
CGTGGGGCCCACCTCATCCGGGGAACGCGACGCGGACGGCTGACGTGCGGCCGCACGCGCAAACGA
GGGAGAGGTCTAACTACGCGGGCCCCTACATCGCGCCCAGCTTTTACCCACCGCGAAACGTCTCCA
TAGACCCGGTCCGTCGCATCCCCACATGAACCTTCTACGGTCTTAGCGCCGCACGTGAGGCTAAGA
CCTATGCACCGGGTCCAGGAGCTTAGCCTCCACGCGCCGGAGAGCTCGCGCACCTCTGCCCGCTTC
GCGCGGCCATTGGCCGCGCTGTTTATGGGGGAAAAATATATTTTTTCCCGTAAAAAAAGTGAAAAG
ATAGCGATAACGGAAACCCTAATCCCCAAATCAGAAATCTATCCGAATCGCTATTTATGTTTGTGG
CTTTCTCCCCCCTCCGATCCGATGTCCCCCGCTTCGTCCGCCGCGCCGATCCGACCTCCAGCCGCC
GCATTCTCTCCGCCGGGGACGCTCCCTCCGGCGCCGCCGGCTCCGCGCCATGAGGAAGAAGCTCGA
CACCCGCTTCCCCGCGGTATGACCCATGCCCATGCGATTTTTTGCCGGTTCAGGCCTCCCCTATGC
TGTGCGCGCCGTCGATTTCGCGATTTTTTATGTGCCTCGTCTAGGTTTTGTTTCCAGCGGTTAGGT
TTTCGTAAATTGGCGGCCAATGGACCTGCGATGTGCATGTTTACATGTTTCTCTCCATGGCTGGGC
TTGTTAGATGCACGTTTGGGGATTTTTTTGGGTGCTGTCGGTAAATAGCATGTAGATTTCGCTGCA
CGGTTATGATTTTTAGCCTCTTGCGCGTGCTCATCCTGGGCTTGTTTCATGTACGTTTCTTTTATG
GTGGCTATGGTTCGCTGTCCTGATGGCAATGAAGCTGTGGTTATAGATGGGCATATGATGTAGGCA
GGATGCGTGGAAAATCCTGGATTGTGTAGATTCTGCCTACTCTGGTAATGTTTCACTAGTATATGG
CTTTGCCGCCGACGAGAGTGCTCTATTAATCTACTTTGTTATTTAACATCAGGGTCAATTTATTTT
ATGTTTGATTAATTCGTTATGATTGAATCCTTGGGTCAACCTTGTGCTTCAATTGCTAGGTTTGTT
CTCTAAGGAATGAAGGCAACCGAAATTACTTGCATTCACTATTTACTGAAAAAGATGGACATGAT

TATTAAATAACTCTTTTATAAATATGAGTTTTTTTAGGAATGAGAGTTCAACTTCAGCGGAGATGG
CTTAATTGTCTCGAGAGAAGATCAACTCCAGGTCTTCTGCGGTGTTTCCTATTTTGGTCGGTAGCT
TGCTTCAAACCGGGCCGATTGTACCTAAACTAATATTCCCACATAATGAACACCAAGTGCTCCTTC
CTTTTTTCCTAAATTAAGAGAGGAGTGCGATTGTTGGTACAAAGTGCTAACTATAGAATCAAGCAT
GTGAGTTCTTTTTATAGTTGTTGATCTGTTGACATTGGTCTCATGATTGTTTAGATATAGATTGTG
GTGGCAGCCTGGCAGGGAAGCATCTTAGCCCGACGAGTTGGTACTGGTCAGATTTTGTAGTATATT
TCAAATGGTAAATAGACAGTAGGATTCTACTATTTAACATCTGGCCCACAACAGGCAATGAGTGTT
GGTTAAAGCTGTGTACAATCTATAAAAATATGTAGTGTAGAGTGTTTATTTTATTCTGAACATGGT
ATATTCTCTGATCTGTTTTTGGTCCATCTTTGCGGGAAGATGCAAGTGTTTTGATGACATATGTTT
GCTGTACAATGGAATCCTCATGATACTACAGTTTTTCTACAGTGGATAGAGATACCTTCTTCATTA
CTCTTTCTTTCACAAGGGCTAGCAGAATCTCTTTACCTCATCTGCTAGGACCTGTGCTTATAACGA
CATGTGAATACTATCACTGTCATGTCCTTTTCCATCGAAGTTGCATCGTTGGCCATTTGCACTGAA
GTATGCAACAATCATAATTATGATTAATGAGTGTTTTATTGATGACATGTTGTCATAATTTCAAAA
TGGCAGATTCCCACTACTTTTCAGATGGTATCATGGTACTCTTCTGTTAATGTGATATCAGCTTCC
TCTTGTACTTTGTTATACAGCCTCGGATTAAGAAGATCATGCAAGCAGATGAAGATGTCGGCAAGA
TTGCTCTAGCTGTACCTGTTCTAGTTTGTAAGTTTTCCACAGCCTCTTGTTTGCAGAACTTTCCTT
CAATTCGCCTCTTATCAGTTCCAGAGTTATTTCACTACATACATCATCTTTTTTTCGCCATTTGTT
GTTAACGAGAGAGTTCGGTAGCATATCTTCAAGAGGCCATAGACATATGCTACATACACCGTTTGT
CTATCTAAGCTGCGCCTCTTCAGAAACATAATAATTACTATCTTATTTAAATGTAGTTGGCAAAAT
AGCTCCATTGAAGGGCTCCAGAAATGGCAGTGTTGACCGCTGTAATAGGAATTTCATCTTTGTTTT
TGTTTTCTCATATTCATACCGAAGCTAAAAATAATTGCAGACATTAGTATCTATTGTTCCTTCTTG
GTGTAAACAAAACATAGGCAAAATTGCAGAACTATTTGGTCCTTTCGGGAAGTTGACAGGGATAGA
TCCTCTGAATTTATCTCTACAGAGTCGTGTTTTGTATTGTTTGGAGCATGACACGTTTGCAGCTGC
CGGTCAGTCAGCACTACACTTTGATTCAGATGACATGATTTGATATATGGTCATTCTAGATCTCAA
TTTCAAACTAGAGAATCATACTGACCATAAAGCATATAGGGAAGATGTAGAGCAACGGTAGTCATC
TTTCTTGTTAAGAGGAGGAACTTGCTACCGAGGTCAGCAGAGCCAAGGTGCAAATGTGAATCCTTT
GGGCCAGGGAAACTGAGTTCAGGCTCAATGGTCAACCATGTAAGCCTATTCTCCTATATTAATCCA
TGCATCCACAGTTGCCTTTACACCTCCCCCACAGGTGCGTCACCACCATATTTCCATGCCAGAGTG
ACCCCTGACTGGCTCCTCCACCAGGCATCTCATCTCTACCCTGTTAGCTCAGCATACGCTACCAAC
ATGTCTTGATGATTTGGCTTTCTCTGTTCTCCTGATTTGTAGCTAGTAAATTTATTTTACATGTCC
TGGTGGGTCAAACTGGGTAGAGGAGAAGATGGGCACAAGTTAGGTGGGGAGCCATGGTGCGGGGAA
AGGGAAATCTTGGAGAGGGCTAGACATGGGAAGAACTTGTGTATATATTGCACAGTATTAAGGACC
ACAAAATTGAGTGGAGTACTTAAGCGTACTCGATGACTATGGCGGGACATACATTAAATGAGAGAA
GGGTAGCCGCGGTAAGAGTTGATGTGTACCTGCTGTTTGCAATATAGGACCATACATTGAGTGAGT
ACAGTAATTAAAGATACTTGAGGATCGGCACTCTGGCGGGTCATACTTCGAATGTAAGCCGTTCCA
TTTTAGACGCATGTACAAAAATGCCGGTGCGTCGGTAGACAGCAAACCCCTACTTTTATAAGCAGT
ATAGATAACATCAACAGCTAGGTATAGCTTAATTGCATGTGCTTATGTGAACTATATAATTAATTA
ATGTTGGCATGTACAATGTTGATATATGATTGACACAAGCTATATGCAATAGCAAAGCAACTCGGC
CTCTTATAGTGCACCTACGTGCTTATATTTTCTTAGTTTCTTTTTGTAACTCACATCATGCATGTG
CTTTCGACTAACATCTTTTTCTGATATCATGATTTGCAGCCAAAGCGTTGGAGCTATTTTTGCAAG
ACTTATGCGATAGGACATATAACATCACTGTTGAAAAAGGGGTCAAAACAGTGAGTTCATCACATC
TGTAAGTTTCCTTGACCTTGTATGTTTTGTCTATCGAAACACCTTCTTGATTAGTATGGCAAAACC
TTGGAAGCATTACCATTCGTCATTTTGATTTTTCTTGGCAATACATTGTGCTTGCAATTTTGTACC
TATAAATTGTTTCCTCAAGTTCAGTTCTACTGTCATATTCTGTCCCTTGTGTAGTTTATGCAGTGT
CCAAATATACCAATGAGCAAGTTGCTCAGTAACACTACAAATATCACATGGGGAAATAACATCGTT
TCACTGATGACATACCGTCAGGATCCACCTTTCATTGTCACATTGGCTGTTATTTTTCAATTTGCA
GCTTTACTAAACAAATTCACTTGTATAACAAACATCAGAAGTGAAAAGCTTTGATGTACTGATATT
GCTCTAGTACTCTTTCACAATGACATTTTCAGGAACAACAAATATGGGCTAGCTCCTTTTGAACTG
AATTTAATGTCATAGTTGGTTAGCTTATATGTCAATCCTATTTAGTAACTGATTGGGCCCTGGTGC
TACTTTGTGCCAAAGTCAGCAGCGTGTTATATGGATTATGGAAGCTGTAAAAGTACCTTCCCTGAT

ACACGTTTTAAACTAAAGGAGAGAGGTAGATAAGCATGTGTCTTGTGGTGGTCTATGCTGATTTTG
GGGTATTACCGTCTTGGTGGGGGTCCTCAGGGACATGCAGTTGGGTGGTGGTTGCATCATTTCAAT
TTGTGGTAGAAATGGGACTTATTTTCTTGATCCTGATATGTATTATTTCCTTGAGCAAAACATTTT
TTTTTCTGACGGTCCCATTATCTCATTCAGTATTATGCTATTACAGGAAACAGTGCATTCATAGTT
ACGATGTGTATGATTTTTTGAAGAACGTTTCTAGCAAAGTTCCAGATTTGGGTCCACCTGATTCTA
GTGTTGATGATAAGCTTGGCAAAAGAAGGCAAGGTTCCAAATTGTGGTTAATTAGAGTCTGATATG
AACTTTAATAATTGGTTAATTTGAGTTTATATGAACTTTAATAATTGCTGAAATGTCATCAGGAAA
CATGGTGAAGATGAAAGCGAGGAGGAATCGAAGCGGACAAGAAATGTTAGTCTCTCTGACCTGTAC
CTTTTCTGTATATGTTACACTTACATGTACATTTTTGTGATTTTAGTATGAGCTCAGGATTTTCAT
TTTCTCTGCTATTCAGCCTGTAGCTTGTTTCGTATGTCACAAAATATATGATCACCCCATTTTATG
TCGCCCATTCGACCTCCTTTTGCTGATAAAAGTTTTGCACATGCAGGAGGCAGCATGCCACACAAG
CAACGGCAGAGGGCGTGGGAGGGGACGTGGGAGAGGTCGCCGTGCTGGGCGTGGAGCTGAGAGGGA
GATTGAACACCATGAATTAGGTTGTGCCCAATTTAGCAAACCAGGCCACCTGAAGGTAGAGATTGG
AGACGTTGTTCTGGACACAAGTGAAACCAAAGAGCATACCCCTTTGAGCAATGCCAGGGCCTCCTT
AAGGAACATTGACTTGAACTTAGATCTTGCTGACTACGAGGAGGATACAGTGGCGCCGCAAGTCCA
GCCTTCAGCTCCTGTGGTGACCGTGGCACCGCAAGTCCAGACTTCAGGACCATCGGTTTCACAGTT
GAAAGAAGAAGTGGAGACCAAGGATTTCCTGGGCTGGCAAATGCCTGAGATGAACAAGATGGCCAT
GGACCCGGTCCAGTTTGCGTTGTCGTCAAACCATAGATTAGAGGAGGACGAAGACTATGACAATGA
AGAATGATGTGACTCTAGTTTGAGAAGCTGATAGGTAGACATGACATGATGTACATCCTAGCAGGC
CAAGCTTGACAATGTAGTAGCAATTCTCTTGTAGTATGTGTACTGGCAGATGCTGCAGAAGTAATT
TAGGATGTGACATATGTTGATTGCTCAATAGCCAGTAGTAGCTAGTATTTTGTGTAGCAACTATAT
AGTGTAGCAACTATATAACTAGACAGGTTCTCCGGTTGTAAATCATGGTTGCTTTTAGATCAAATG
CTATGTCATAATGCTTATGTTAGCTCTTACGAGGTTCCACTCTTCCAGGTGGATTTTTTCTCTTGA
TTTAGTTATTAATAATCTTGTGTTATTTGTGAATTTTCTCTTCCAAGTGGGATTTTTCTAATCTTG
TGTTATTTGTGATTTTTCCTCTTCCAGGTGGAATTTTTTACTTGCTTTAGTGATTAACAATCTTGT
GTTATTTCTGAATTTCTCTCTTCCAGGTGGGATTTTTCACTTGCTTTAGTGATTAACAATCTTGTT
ATTAAGACATCAAGCTTTCGAGTTATGTGGGAGGTGAAGTGCAAACAAGCAAAATACTTGTGGGAA
TCAATCATACACATCAGGCAGGAAGCAGGTTTATTTCATCATATTACACCAGTATCCAGTGAGGCT
GGCATGTTTTTTGATAGGCTCCAGCTACAAGCTTCAGCGGTTCAGCTCACATAACATATTTATTTA
CATCTTTTCAGGCGGCCGGCTTGAACTCCTGGAGCTGCTTGAAGTTCTCCCAGGCCTTCTCCCACA
CCTTGGCCTCGTAGAGCTTCTTGGCGCCGCCTTCCTTGACCTCGATGGTGAAGTAGTGCATGCACC
CGGCCACCACCTGCTGCCTCACCTTCACCAGCCTCTCGAACTCCAGCAGCGCGTTCTGCGCACAAC
AAAATTCACCATCAGGTTAATCAGTCAGTTACAGTTACGCGTATCAGACGCCAGATGGCTTGATCG
GGTGGGGATGATCTTACGGCCTTGGTGTTGTGCTCGGCGACGGCGAAGCGTGCGAGCTTGATGGTC
GCGAGGTCGTTCTCCCGCCCCGCCGGCGCGTCCTGGACGCCGCCCAGCAGCACGCCGCGGCCGCGT
AGCCCCCCGCCCTGCGCCGCCTCGGCCATCGCCGCCGTGCGTCCGGAGGGCGCGCTGAGCCGAGAG
AGGAGCGTACGACGGATAACCGCTGCTGAGAGTCTGAGAGAGAACAGATCTATTCCGTGAGTCTAA
CGATGTTGATCGAGGGAAGTTGGGGACGGGACTTTATAGCGACGTGGGGGTAGAGGGATTCCGTTT
TTGTGCGCGACGTGTGATCTGCGGGGGCTCGAGCGCGACAGCTATAGGCGACGGACGGGCCGGACG
CTGTACGCGGCAGCGGTGGGGCTGTGTGTCGACGCGACGGACGCGGTACGCTGCAGCGCTGGCTGT
GCGATGGCGACGGCGACGGCGATGGATGCGTTGCAAACTTGCAATGCTTAGCTCCGAAGGCAAGTG
CTGGCGAAGTAAAGAGCAGCAGACCCGGTGAAACGGTTGTTTATCTATCCCTGGAAAACAATATGT
ATGCGTTTGGTAGGCTGTATTAGGCCCAGCCAGGCTCGGGCGTGCAGAATTCGGCCTGGGCCATTT
GGTTGCCTGGGCTGCATCTAAAATCTGGCCCGCATGAATCTCAGAGCTGGCTGAGAGCTCTGGACG
AACGGCCGAATCGGCAAATTTTTAAGAGTCTGTCTCGCTCGAGCCATGCGTGGGAAGCGCGGGAGA
CGACATGACGTCTCTAACTCTCCTTGCTCCTTGCAATCACCACACTCGACAATTCGCACGCACTGT
CTCCCCTCTCGCCTCACCGCCCATTCGGTTTCTCCGATGGTAGAGTCGCCATCTCGCCGCGCAGCC
CGTGAAGTCCCGTTTTACGGTGAGCCACACCCCTCCCGATGAACATGATCCCTGTTTCGACCGTAG
GAGGTCGAAGAGAAGTCCGTTTCCTCCATTTTGTGGTACGCCACACCCCTCCCGATCAACAGGAGA
AACGGGGTGTTTCCTTCATTTTGCGGTACGCCAGACCCCTCCCAGTGAACATGATCCTGTTTTAAC

TGTAGGCGGTCGAACAGAAGGCCATTTCCACCATCCCGCGGTACGCCAGGCCTCGTTTCGGCCGTT
CCGTCCAAGCCGGTTGGCTCCCGATGAACAGGACGCATTTTGTTACCTCCCGATGAACACGACGTA
GTTTCTCTGTTTCGACCCAGCCGGTTGGTTGCCGATGAA

***>TaDrAp2-B1*-CDS-852bp**

ATGAGGAAGAAGCTCGACACCCGCTTCCCCGCGCCTCGGATTAAGAAGATCATGCAAGCAGATGAA
GATGTCGGCAAGATTGCTCTAGCTGTACCTGTTCTAGTTTCCAAAGCGTTGGAGCTATTTTTGCAA
GACTTATGCGATAGGACATATAACATCACTGTTGAAAAAGGGGTCAAAACAGTGAGTTCATCACAT
CTGAAACAGTGCATTCATAGTTACGATGTGTATGATTTTTTGAAGAACGTTTCTAGCAAAGTTCCA
GATTTGGGTCCACCTGATTCTAGTGTTGATGATAAGCTTGGCAAAAGAAGGAAACATGGTGAAGAT
GAAAGCGAGGAGGAATCGAAGCGGACAAGAAATGAGGCAGCATGCCACACAAGCAACGGCAGAGGG
CGTGGGAGGGGACGTGGGAGAGGTCGCCGTGCTGGGCGTGGAGCTGAGAGGGAGATTGAACACCAT
GAATTAGGTTGTGCCCAATTTAGCAAACCAGGCCACCTGAAGGTAGAGATTGGAGACGTTGTTCTG
GACACAAGTGAAACCAAAGAGCATACCCCTTTGAGCAATGCCAGGGCCTCCTTAAGGAACATTGAC
TTGAACTTAGATCTTGCTGACTACGAGGAGGATACAGTGGCGCCGCAAGTCCAGCCTTCAGCTCCT
GTGGTGACCGTGGCACCGCAAGTCCAGACTTCAGGACCATCGGTTTCACAGTTGAAAGAAGAAGTG
GAGACCAAGGATTTCCTGGGCTGGCAAATGCCTGAGATGAACAAGATGGCCATGGACCCGGTCCAG
TTTGCGTTGTCGTCAAACCATAGATTAGAGGAGGACGAAGACTATGACAATGAAGAATGA

**>TaDrAp2-B1-pep-283aa**

MRKKLDTRFPAPRIKKIMQADEDVGKIALAVPVLVSKALELFLQDLCDRTYNITVEKGVKTVSSSH
LKQCIHSYDVYDFLKNVSSKVPDLGPPDSSVDDKLGKRRKHGEDESEEESKRTRNEAACHTSNGRG
RGRGRGRGRRAGRGAEREIEHHELGCAQFSKPGHLKVEIGDVVLDTSETKEHTPLSNARASLRNID
LNLDLADYEEDTVAPQVQPSAPVVTVAPQVQTSGPSVSQLKEEVETKDFLGWQMPEMNKMAMDPVQ
FALSSNHRLEEDEDYDNEE*

## ***TaDrAp2-D1*** (TraesCS1D02G310200)

**TraesCS1D02G310200 <u>FORWARD STRAND</u>**
pep chromosome:IWGSC:1D:406545874:406551732:1 gene:TraesCS1D02G310200 transcript:TraesCS1D02G310200.1 gene_biotype:protein_coding transcript_biotype:protein_coding

***>TaDrAp2-D1***
TGCTTGGCTTGGGGGGCAAGTCTCCCTAGGATTTTCCCTAGGGAGATCCAATCTGCTTGGCCGCCG
CCCCCTAGGGGAAACCCTAGGGCGCCTCCCCCTCTCCCCTTGCCCATATATATAGTGGAGGGGTAG
GGGGCAGCCGCACCTCTTCCCTGGCGCAGCCCTCTCCTCCTCCAACTCCTCCTCCTCCTCCGTAGT
GCTTAGCAAAGCTCTGCCGGAGAACCACGAGCTCCATTGCCACCATGTCGTCGTGCTGCTGGAGTT
CTCCCTCAACTTCTCCTCTCCCCTTGCTGGATCAAGAAGGAGGAGACGTCCCCGGGCTGTAGGTGT
GTTGAACGCGGAGGCGCCGTCCGTTCGGCGCTAGATCGGATCTTCCGCGATTTGAATCGCCGCGAG
TACGACTCCATCAACCGCGTTCTTGTAACGCTTCCGCTTAGCGATCTTCAAGGGTATGAAGATGCA
CTCCCTCTCTCTCGTTGCTAGCATCTCCTAGATTGATCTTGGTGACACGTAGGAAAATTTTGAATA
ATTACTACGTTCCCCAACAGAGGCCCAATGGGATGGGATGGAGCGCGCCCTGGCCTTGTCGGCGGC
AGGGGACTCCGTCCATGCCCTGCTCTTCGACTCGCCTCCACCGCCGCCCATCGTCGAGCCCAAGCC
AGAGTCGGAGCCGGCGCGGAAGCGCTCGCCGCCTCCACCCACTTGGCCGGAGGAGTCCTACACGTG
GACCGGCGAGTACCGCGAGTGGGTGAGCGCGCCTCCGGTCCACTACGCCGCGACGCCGGAGGAGGA
GGCAGCCCACCTTGAGCGTTGGGAGGCGCACTGGCTCACTCAGGAGGAGGCCGACGGCGAGCAGCA
GATGCTTTGGGAGCAGCAGCTGCGGCTTGAGGAGGAGGAGGAGCGCGCCCGGCTAGCCGCAATGCC
GGCGCCCCAGCAGACGCCGGAGGAGGCTGCCCTGGCAGAGTACCAAGCGGCATTCGGGTGGGCTAG
CCCTGCTCCCGCCTTCATCGACCTCACCGGTGGCGATGGCGTCGTCAAGGGCAAGGGCAAGGCGGA
GGACGTGTAGGGCAGCGTGAGGGCGTAGACTTTTTTTAATTAATGTTTAATTAGGTTTAAGTGGAC
TTTGGTCGGCGGTTGACCAGCCACTTTAATGTTTAATTATGTTTATTTCTACGACCTGTTTTTCTT
CTACGCCGACAAATTTGGGTCCGTCTTTCGTTGGGCGCACCTGCCGACCCATATAAAAATGCGGAC
GTGCACGTCCGCCTGACCGATCTAAACGGACGAAAAGCGGACAAAACGCGCGTCCGTTTGGTCGGC
GCCTTGGAGGTGCTCTTACACCCTGAAACATGATTCCCTTTACATTTACGTTAACACTCCTCAAAA
CAAACACGGCAATCTATTCTGATCTCACAAAGACAAGTCGGAGCATACTCTCTACCCAGATAAAGC
AACAGCCCAAGAATGGACGATGACTCTCGCAGCAAGGCCACGAACGAGGCACGGCGCCACAGCCGC
CGGATCCCGAATCGGACGGCGGACACAGGGAGCGCCACGAACCGGCCAGAAACGTGGGGCCCACCT
CATCCGGGGAACGCGACGCGGACGGCTGACGTGCGGCCGCACGCGCAAACGAGGGAGAGGTCTAAC
TACGCGGGCCCCCACATCGCGCTGAGCTTTTACCCACCGCGAAACGTCTCCGTGGACCCGGTCCGT
CGCATCCGCCACATGGACTCTACGGTCTTAGCGCCGCACGTGAGGCTAAGGCCTATGCGCTGGGTC
CAGGAGCTTAGCGTCCACGCGCCGGAGAGTTCGCGCACCGCTGCCCGCTTCGCGCGGCCATTGGCC
GCGCTGTTTATGGGGAAAAAAATAGTTTGTTTTTATTTTTCCCCGTAAAAAAAGTGAAAAGATAGC
GATAACGGAAACCCTAATCCCCAAATCAGAAATCTATCCGAATCGCTATTTATGTTTGTGGCTTTC
TCCCCCCTCCGATCCGATGTCCCCCGCTTCGTCCGCCGCGCCGATCCGACCTCCAGCCGCCGCGTT
CTCTCCGCCGGGGACGCTCTCTCCGACGCCGCCGGCTCCGCGCCATGAGGAAGAAGCTCGACACCC
GCTTCCCCGCGGTATGACCCATGTCCCTGCGTTTTTTTTTTGCCGGTTCGGGCCTCCCCCTACGCT
GTGCGCGCCGTCGATCTCGCGATTTTTTTGTGTGCTTCGTCTAGGTTTTGTTTCCGGCGGTTAGGA
TTTCGTAAATTGGCGGCCAATGGACCTGCGATATGCATGTTTACCTGTTTCTCTCCATGGCTGGGC
TTGATAGATGCACGTTTGGCGAATTTTTGGGCGCTGTCGGTAAATAGTATGTAGATTTCGCAGCAC
GGTTAGGATTTTTAGCCTCTCGCGTGTGCTCATCCTGGGCTTGTTTCATGTACGTTTGTTTTTATG
GTGGCTATGGTTCGCTGTCCCGATGGCAATGAAGCTGCGGTTATAGATGGGCATATATGATGTAGG
TGGTGGATGCGTGAAAAATCCTGGATTATGTGGATTCTGCCTACCCTGTTAACGTTTCACTAGTAT
ATTGCTTTCCGCGGACGAGAGTGTGCTATTAATCTACTTTATTATTTAATGTTAGGGTCAATTTAT
TTTATGTTTGAATAATTCGTTATGATTAAATCCTTGGGTCAACCTTGTGCTTCAATTGCTAGGCCT
TGTTCTTTAAGGAACGAAGTTAACAGAAATTACTTGCATTCACTATTTACTGAAAATAGATGGACA

TGAATATTAATAACTCTTTTATAAATATGAGCTTTTTTTAGGAATGAGAGTTTAACTTCAGCGGAG
GTGGCTTAATTGTCTCAAGAGATGATCAACACCAGGTCTTCTGCGGTGTTTCCTATTTTGATCGGT
AGCTTGCTTCAAACCGGGCCGATTGTACCTAAACTAATATTCCCACATAATGAACACCAAGTGCTC
CTTCCTTTTTTTCCTAAATTAAAAGAGGAGCACGATTGTTGGTACAAAGTGCTAACTATAGAATCA
AGCATGTGAGTTCTTTTTATAGTTGTTGATCTGTTGACATTGGTATCATAGATATAGATTGTGGTG
GCAACCTGGCAGGGAAGCTTCTTAGCCCGACGAGTTGGTACTGTTCAGATTTTGTAGTATATTTCA
AATGGTAAATAGACAGTAGGATTCTACTATTTGACATCTGGCCCACAACAGGCAATGAGTGTTGGT
TAAAGCTGTGTACAATCTATAAAAATATGTAGTGTAGAGTGTTTATCTTATTATGAGCATGGTATA
TTCTCCAATCTGTTTTTGGTCCATCTTTGGGGGAAGATGCAAGTGTTTTGATGACATACGTTTGCT
GTACAATGGAATCCTCATGATACTAAAGTTTTTCTACAGTGGATAGAGATACCTTCTTCATTACCC
TTTCTTTCACAAGGGCTAGTAGAATCTATTTTCTTCATCTGCTAGGACCTGTGCTCATAACCACAT
GTGAATACTATCGCTGTCATGTCCTTTTCCATCGAAGTTGCATCGTAGGCCATTTGCACTGTAGTA
TGCAACAATCATAATTATGATTAATGAGTGTTTTATTGATGACATGTTGTCATAATTTCAAAATGG
CAGATTCCTACTACTTTTCAGATGGTATCATGGTACTCTTCTGTTAATGTGACATCAGCTTCCTCT
TGTATTTTGTTATACAGCCTCGGATTAAGAAGATCATGCAAGCAGATGAAGATGTCGGCAAGATTG
CTCTAGCAGTACCTGTTCTAGTTTGTAAGTTTTCCACAGCCTCTTGTTTGCAGAACTTTCCTTCAA
CTCGCCTCTTATCAGTTCCGGAGTTATTTCATTACATACATCATCTTTTTTTCGCCATCTGTTGTT
AACGAGAGAGTTCGGTAGCATATCTTCAAGAGGCCATAGACATATGCTACATACACCGTTTGTCTA
TCTAAGCTGCGCCTCTTTAGAAACATAATAAGTACTATCTTATTTAAATGTAGTTGGCAAAATAGC
TCCATTGAAGGGCTCCAGAAATGGCAGTGTTGACCGCTGTAATAGCAATTTCATCTTTCTTTTTAT
TTTCTCATATTCAACCCGAAGCTAAAAATAATTGCAGACATTAGTATCTATTGCTCCTTCTTGGTG
TAAACAAAAAAGCAAAATTGTAGAACTATTTGGTCCTTTCGGGAAGTTGACAGGGATAGATCCTCT
GAATTTATCTTTACAGAGTCGTGTTTTGTATTGTTTGGAGCATGACACGTTTGCAGCTGCCGGTCA
GTCAGCACTACACTTTGATTCAGATGACATGATTTGATATATGGTCATTCTAGATCTTGATTTCAA
ACTAGAGAATCATACTGTCCATAAACATATAGGGAAGATGTAGTGCAACGGTAGTCATTTTTCTTG
TTAAGAGGAGGAACTTGTTACCGAGGTCAGCAGAGCTAAGGTGCAAATGTGAATCCTTTGGGCCAG
GGAAACTGAGTTCAGGCTCAATGGTCAACCATGTAAGCCTATTCTCCTATATTAATCCACGCATCC
ACAGTTGCCTTTACACCTCCCCCACAGGTGCTTCACCACCATATTTCCATGCCAGAGTGACCCCTG
ACTGGCTCCTCCACCAGGCACCTCATCTCTACCCTGTTAGCTCAGCGATATGCTACCAACATGTCT
TGATGATTTGGCTTTCTCTGTTCTCCTGATTTGTAGCTAGTCAATTTATTTTACATGTCCTGGTGG
GTCAAACTTGGTAGAGGAGCACAAGTTAGGTGGGGAGCCATGGTGCAGGGAAAGGGAAATCTTGGA
GAGGGCTAGACATGGGAAGAACTTGTGTATACATTGCACAGTATTANNNNNNNNNNNNNN
NNNNNNNNNNNNNNNNNNNNNNNNNNNNNNNNNNNNNNNNNNNNNNNNNNNNNNNNNNNN
NNNNNNNNNNNNNNNNNNNNNNNNNNNNNNNNNNNNNNNNNNNNNNNNNNNNNNNNNNNN
NNNNNNNNNNNNNNNNNNNNNNNNNNNNNNNNNNNNNNNNNNNNNNNNNNNNNNNNNNNN
NNNNNNNNNNNNNNNNNNNNNNNNNNNNNNNNNNNNNNNNNNNNNNNNNNNNNNNNNNNN
NNNNNNNNNNNNNNNNNNNNNNNNNNNNNNNNNNNNNNNNNNNNNNNNNNNNNNNNNNNN
NNNNNNNNNNNNNNNNNNNNNNNNNNNNNNNNNNNNNNNNNTATAGGACCATACATTGAG
TGAGTACAGTAATTAAAGATACTTTAGGATTGGCACTCTGGCGGGTCATACTTCGAATGTAAGCCA
TTCCATTTTAGAGGCATGTACAAAAATGCCGTGCATCGGTAGAAAGCAAACCCCTACTTTTATAAG
CAGTATAGATAACATCAACAGCAAGGTATAGCTTAATTGCATGTGCTTATGTGAACTATATAATTA
ATTAATGTTGGCATGTACAATGTTGATATATGATTGACACAAGCTATATGCGATAGCAAAGCAACT
CGGCCTCTTATAGTGAACCTACGTGCTTATATTTTCTTAGTTTCTTTTTGTAACTCACATCATGCA
TGTGCTCTCGACTAACATCTTTTTCTGATATCATGATTTGCAGCCAAAGCGTTGGAGCTATTTTTG
CAAGACTTATGTGATAGGACATATAATATCACTGTTGAAAAAGGGGTCAAAACAGTGAGTTCATCA
CATCTGTAAGTTTCCTTGACCTTATGTGTTTTGTCATATCGAAACACCTTTTTGTTTGACTGGAGA
GATAACTTATATTACCATATGAAACTTCTTGATTATTATGGCAAAACCTTGGAAGCACTACCATTT
GTCATTTTGATTTTTCTTGGCAATACATGTTCTTACAATTTTGTACCCATAAATTGTTTCCTCAAG
TTACTGTCATATTCTGTCGCTTGTGTAGTTTATGTAGTGTCCAAATATACCAATGAGCAAGTTGCG
CAGTAACACTACAAATATCACATGGGAAAATAACATCGTTTCACTGATGACATACCCTCAGGATCC

CCACCTTTCATTGTCACATTGGCTGTTATTTTCCAATTTGCAGCTTTACTAAACAAATTCACTTGT
ATAACAAACATCAGAAGTGAAAAGCTTTGACGTACTGATATTGCTCTAGTACTCTTTCACAATGAC
ATTTTCAGGAAGAGTCAACAAATATGGGCTAGCTCCTTTTGAACTGAATTTAATGTCATAGTTGGT
TAGCTTATATGTCAATCCTATAACTGATTGGGCCCTGGTGCTACTTTTTGCCAAAGTCAGCAGCGT
GTTATATATGGATTATGGAAGCTGTAAAAGTACCTTTCCTGATACATGTTTTGAACTAAAAGAGAG
GTAGGTAAGCATGTGTCTTGTGGTGGTCTATGCTGATTTTGGGGTATTACCGTCTTGGTGGGGTCC
TCAGGGACATGCAGTTGGGTGGTGGTTGCATCATTTCAATTTGTGGTAGAAATGGACTTATTTTC
TTGATCCTGATATGTATTATTTCCTTGAGCAAAACATTTGTTTTTTTTCTGACGGTCCCATTATCT
CATTCAGTATTATGCTATTACAGGAAACAGTGCATTCATAGTTACGATGTGTATGATTTTTTGAAG
AACGTAGCCAGCAAAGTTCCAGATTTGGGTGCACCTGATTCTAGTGTTGATGATAAACTTGGCAAA
AGAAGGTAAGGTTTCAAATTGTGGTTAATTAGAGTCTGATATGAACTTTAATAATTGGTTAATTTG
AGTTTATATGAACTTTAATAATTGCTGAAATGTCATCAGGAAACATGGTGAAGATGAAAGCGAGGA
GGAATCGAAGCGGACAAGAAATGTTAGTCTCTCTGACCTGTTCCTTTTCTTTTTATGTTACACTTA
CATGTACATTTTTGTGAACAGTTTTTAGTATGAGCTCAGGATTTTCATTTTCTCTGCTATTCAGCC
TGTAGCTCGTTTCGTATGTCACAAAATATATGTTCACCCCATTTTATGTCGCCCATTCGACCTCCT
TTTGCTGATAAAAGTCTTGCACATGCAGGAGGCAGCATGCCACACAAGCAACGGCAGAGGGCGCGG
GAGGGGACGTGGGAGAGGTCGCCGTGCTGGGCGTGGAGCTGAGAGGGAGATTGAACACCATGAATT
AGGTTGTGCCCAATTTAGCAAACCAGGCCACCTGAAGGTAGAGATTGGAGACGTTGTTCTGGACAC
AAGTGAAACCAAAGAGCATACCCCTTTGAGCAATGCCAGGGCCTCCTTAAGGAACATTGACTTGAA
CTTAGATCTTGCTGACTACGAGGAGGATACGCCGCAAGTCCAGCCTTCGGCTCCTTCAGCTCCTGT
GGTGACCGTGGCACCGCAAGTCCAGACTTCAGGACCGTCGGTTTCACAGTTGAAAGAAGAAGTGGA
GACCAAGGATTTCCTGGGCTGGCAAATGCCTGAGATGAACAAGATGGCCATGGACCCGGTGCAGTT
TGCGCTGTCGTCAAACCATAGATTAGAGGAGGACGAAGACTATGACAATGAAGAATGACGTGACTC
TAGCGTGGATTTAGTTTGAGAAGCTGATAGGTAGACATGACATGATGTACATCCTAGCAGGCCCAG
CTTGACAATGTAGTAGCAATTCTCTTGTAGTATGTGTACCCGCAGATGCTGCAGAAGTGATTTAGG
ATGTGACATATGTTGATTGCTCAATAGCCAGTAGTAGCTAGTATTTTGTGTAGCAACTATATAGTG
TAGCAACTATATAACTAGACAGGTTCGCCGGTTGTAAATCATGGTTGCTTTTAGATCAAATGCTAT
GTCATAATGCTTATGTTAGCTCTTATGAGGTTCCTCCACTCTTCCAGGTGGATTTTCTCTCTTGCT
TTAGTTATTAACAATCTCGTGTTATTTGTGATTTTTCTCTTCCAGGTGGAATTTTTCACTTGCTTT
GGTGATCAACAATCTTGTGTTATTTGTGATTATTTTCTCTTCCTGGTGGGATTTTTCACTTGCTTT
AGTGATCAACAATCTTGTGTTATTTGTGAATATTTCTCTTACAGGTGATATTTTTCTCTTGCTAAG
TGATTAACAATCTTGTGTTATGTGTGAATTTTTCTCTTCCAGGTGGGATTTTTCTCTTGCTTTAGT
GATTAACAATCTTGTGTTATTTGTGATGCAAGACATCAAGCTTTGGAGATATGTGGGAGGTTTAGT
GCAAACAAGCAAAATACTTGTGGGAATCATAGACATGGCAGGAAGCAGGTTTATTTCATCATATTA
CACCAGTATCCAGTGAGGCTGGCGTGTTGGATAGGCCCCATCCACAAGCTTGAGCGGTTCAGCTCA
CATAACATATTTATTTACATCTTTTCAGGCGGCCGGCTTGAAGTCCTGGAGCTGCTTGAAGTTCTC
CCAGGCCTTCTCCCACACCTTGGCCTCGTAGAGCTTCTTGGCGCCGCCTTCCTTGACCTCGATGGT
GAAGTAGTGCATGCACCCGGCCACCACCTGCTGCCTCACCTTCACCAGCCTCTCGAACTCCAGCAG
CGCGTTCTGCGCCCAACAAAATTCACCACCGATCCGTCAGGCAGGTTAATCAAGAAAATTCACCAC
CGATCCGTCAGGCAGGTTAATCAAGAAAATTCACCACCGATCCATCAGGTTAATCAGTCAGTTACA
GTTAATCGTATGAGACGCCAGATGACTTGGGATCGGGCGGGGATGATCTTACGGCCTTGGTGTTGT
GCTCGGCGACGGCGAAGCGCGCGAGCTCGATGGTCTCGAGGTCGTTCTCCCGCCCCGCCGGCGCGT
CCTGGACGCCGCCCAGCAGAGCGCCGCGGCCGCGTAGCCCCCCGCCCTGCGCCGCCTCGGCCATGG
CTGCCGTGCGTCCGGAGGGTGGATTGTACGACGGATGGCCGCTGCAGAGAGTCTGAGAGAGAACAG
ATCTGTTCCGTGAGTCCAGACGATGTTGATCGAGGGAAGTGGGGGACGAGACTTTATAGCGAGGTG
GGGGGTAGAGGGATTCCGTTTTTGTGCGATGTTCCCTGTCAAACGACGTGTGATTTGCGGGGGCGC
GACAGCTATGGGCGACGGACGGACCGGACGCGGTACGCGGCCGCGCTGGCGCTGTGCGTCGACGCG
ACGGACGCGGTACGCAGCAGCGCTGGTGCTGTGCGACGGCGACGGCGACGGATGGCGTTGTTGTAA
TACTTAGCTCCGAAGGCAAGTGCGGGCGAAGCAAAGAGCAGGGTATCCGTCCTGAAAACTCATACA
TGATTCTTTTTTTGTTGTTGTTAATCATGTCATAAGATCACATGGCACATATGTCATAACCAACTC

ATAAGATTCATAACCAACTCATAAGATTCATATGATGTCAAAAAAGAAAAACTCATAAGATTCATA
TATTTCATAATTGTAACATACTCCCGAGTAGGACGTTTTGGTGCCCAGGCTCATCTGCACCCTGTT
AGAAAACAATTCGTAACAAATTCAAAAAAAATAAAAAATAAACCAAAACAAAATTTTTGAGGTAGA
CAATTTGATGCGTGAGGCCTGCTCCAAATTTCAAGTCATTTGGACGTCTGAGCTGCTCTCAGCAAG
AAAGACAAATCAGACCAAAACAGTACATGAACAGTAAATATTTTTACGGACCTCTAATTTGTCTTT
TTTTGCCGAGAGCTCTCCAGATGTTCAAATGATTTGAAAATTTGATCGAACCTCACGCATCAAATG
GTCTACCGCATGGAAAAAAAATTGATATTTTTTGAATTTTCCTAGTATTTATTTTGATTTTTTTCG
TTAGCGGGGGTGCAGATGAGCTCGG

**>*TaDrAp2-D1*-855bp**
ATGAGGAAGAAGCTCGACACCCGCTTCCCCGCGCCTCGGATTAAGAAGATCATGCAAGCAGATGAA
GATGTCGGCAAGATTGCTCTAGCAGTACCTGTTCTAGTTTCCAAAGCGTTGGAGCTATTTTTGCAA
GACTTATGTGATAGGACATATAATATCACTGTTGAAAAAGGGGTCAAAACAGTGAGTTCATCACAT
CTGAAACAGTGCATTCATAGTTACGATGTGTATGATTTTTTGAAGAACGTAGCCAGCAAAGTTCCA
GATTTGGGTGCACCTGATTCTAGTGTTGATGATAAACTTGGCAAAAGAAGGAAACATGGTGAAGAT
GAAAGCGAGGAGGAATCGAAGCGGACAAGAAATGAGGCAGCATGCCACACAAGCAACGGCAGAGGG
CGCGGGAGGGGACGTGGGAGAGGTCGCCGTGCTGGGCGTGGAGCTGAGAGGGAGATTGAACACCAT
GAATTAGGTTGTGCCCAATTTAGCAAACCAGGCCACCTGAAGGTAGAGATTGGAGACGTTGTTCTG
GACACAAGTGAAACCAAAGAGCATACCCCTTTGAGCAATGCCAGGGCCTCCTTAAGGAACATTGAC
TTGAACTTAGATCTTGCTGACTACGAGGAGGATACGCCGCAAGTCCAGCCTTCGGCTCCTTCAGCT
CCTGTGGTGACCGTGGCACCGCAAGTCCAGACTTCAGGACCGTCGGTTTCACAGTTGAAAGAAGAA
GTGGAGACCAAGGATTTCCTGGGCTGGCAAATGCCTGAGATGAACAAGATGGCCATGGACCCGGTG
CAGTTTGCGCTGTCGTCAAACCATAGATTAGAGGAGGACGAAGACTATGACAATGAAGAATGA

**>TaDrAp2-D1-284aa**
MRKKLDTRFPAPRIKKIMQADEDVGKIALAVPVLVSKALELFLQDLCDRTYNITVEKGVKTVSSSH
LKQCIHSYDVYDFLKNVASKVPDLGAPDSSVDDKLGKRRKHGEDESEEESKRTRNEAACHTSNGRG
RGRGRGRGRRAGRGAEREIEHHELGCAQFSKPGHLKVEIGDVVLDTSETKEHTPLSNARASLRNID
LNLDLADYEEDTPQVQPSAPSAPVVTVAPQVQTSGPSVSQLKEEVETKDFLGWQMPEMNKMAMDPV
QFALSSNHRLEEDEDYDNEE*

## Supplementary file 2

### Protein sequences of DrAp1, DrAp2, Dr1 and references used in Figure 3

### Figure 3A

**>TaDrAp1-A4**
MRKKLGTRFPAARIKKIMQADEDVGKIALAVPVLVSRALELFLQDLIDHSYKITLQSGAKTLNSFH
LKQCVKRYSSFDFLTEIVNKVPDLGGGESCGDERGLPRRRKFSNGSDPENEEPRSSKMPIRSLNTS
PRGRGRGRGRGRGRPPTKRKEIGYVQFEDESSMFAEQSEPLPGDEIVPETNRGNESIPQSSHPLVE
APSAMTPAVISKVEEASTNHQPDWPMPDAIGGIGVGPSSFGHLTVQVDEVEDYDNED

**>TaDrAp1-B4**
MRKKLGTRFPAARIKKIMQADEDVGKIALAVPVLVSRALELFLQDLIDRSYKITLQSGAKTLNSFH
LKQCVKRYSSFDFLTEIVNKVPDLGGGESCGDERGLPRRRKFSNGSDPENEEPRSSKMPIRSLNTS
PRGRGRGRGRGRGRPPTKRKEIGYVQFEDESSMFAEQSEPLPGDEIVPETNRGNEIIPQSSHPLVE
APSVVTPAVISKVEKATNHQPDLPMPDAIGGIGVGPSSFGHLTVQVDEEEDYDNED

**>TaDrAp1-D4**
MRKKLGTRFPAARIKKIMQADEDVGKIALAVPVLVSRALELFLQDLIDRSYKITLQSGAKTLNSFH
LKQCVKRYSSFDFLTEIVNKVPDLGGGESCGDERGLPRRRKFSNGSDPENEEPRSSKMPIRSLNTS
PRGRGRGRGRGRGRPPTKRKEIGYVQFEDESSMFAEQSEPLPGDEIVPETNRGNESIPQSSHPLVE
APSAATPAVISKVEKASTNHQPDLPMPDAIGGIGVGPSSFGHLTVQVDEEEDYDNED

**>TaDrAp2-A1**
MRKKLDTRFPAPRIKKIMQADEDVGKIALAVPVLVSKALELFLQDLCDRTYNITVEKGVKTVSSSH
LKQCIHSYDVYDFLKNVASKVPDLGAPDSSVDDKLGKRRKHGEDESEEESKRTRNEAACHTSNGRG
RGRGRGRGRRAGRGAEREIEHHELGCAQFSKPGHLKVEIGDGVLDTSETKEHTPLSNARASLRNID
LNLDLADYEEDTVAPQVQPSAPVVTVAPQVQTSGPSVSQLKEEVATKDFLGWQMPEMNKMAMDPVQ
FALSSNHRLEEDEDYDNEE

**>TaDrAp2-B1**
MRKKLDTRFPAPRIKKIMQADEDVGKIALAVPVLVSKALELFLQDLCDRTYNITVEKGVKTVSSSH
LKQCIHSYDVYDFLKNVSSKVPDLGPPDSSVDDKLGKRRKHGEDESEEESKRTRNEAACHTSNGRG
RGRGRGRGRRAGRGAEREIEHHELGCAQFSKPGHLKVEIGDVVLDTSETKEHTPLSNARASLRNID
LNLDLADYEEDTVAPQVQPSAPVVTVAPQVQTSGPSVSQLKEEVETKDFLGWQMPEMNKMAMDPVQ
FALSSNHRLEEDEDYDNEE

**>TaDrAp2-D1**
MRKKLDTRFPAPRIKKIMQADEDVGKIALAVPVLVSKALELFLQDLCDRTYNITVEKGVKTVSSSH
LKQCIHSYDVYDFLKNVASKVPDLGAPDSSVDDKLGKRRKHGEDESEEESKRTRNEAACHTSNGRG
RGRGRGRGRRAGRGAEREIEHHELGCAQFSKPGHLKVEIGDVVLDTSETKEHTPLSNARASLRNID
LNLDLADYEEDTPQVQPSAPSAPVVTVAPQVQTSGPSVSQLKEEVETKDFLGWQMPEMNKMAMDPV
QFALSSNHRLEEDEDYDNEE

**>TaDr1A_AAL73486**
MDPMDIVGKSKEDVSLPKSTMTKIIKEMLPPDVRVARDTQDLLVECCVEFINLLSSESNDVCSRDD
KKTIAPEHVIRALQDLGFKEYVEEVYAAYEQHKLETLDSPKATKFTGIEMTEEEAVAEQQRMFAEA

RARMNNGAAKPKEPALEPQNQPQQPPQPHLQLHPQAQQPPQPQPQLHYPQSQQPLQPFTQAPPQQP
LHPQLQQYTQAPPQQPLQPPLQLYPQAQPEQPLQPQSSGSTTGTCVISAAAPSATGTLLLQPPPQQ
SPQSQLQLHQQPQPTLVPPPQPQPQPLELQQPQPLTQLQAEHGLDWDS

**>TaDr1B_BC000036325**
MDPMDIVGKSKEDVSLPKSTMTKIIKEMLPPDVRVARDTQDLLVECCVEFINLLSSESNDVCSRDD
KKTIAPEHVIRALQDLGFKEYVEEVYAAYEQHKLETLDSPKATKFTGIEMTEEEAVAEQQRMFAEA
RARMNNEAAKPKEPALEPQNQPQQPPQPQLQLHPQAQQPPQPQSQLHHPQSQQPLHPQLQPYTQAP
PQQPLHPQLQPYTQAPPQQPLQPPLQLYPQAQPEQPLQPQSSGSTTGTCVISTAAPSTTGTTAAAT
AAPAIPAISTAAPSAT
PADASAAAAATSTPAT

**>TaDr1D_BT009234**
MDPMDIVGKSKEDVSLPKSTMTKIIKEMLPPDVRVARDTQDLLVECCVEFINLLSSESNDVCSRDD
KKTIAPEHVIRALQDLGFKEYVEEVYAAYEQHKLETLDSPKATKFTGIEMTEEEAVAEQQRMFAEA
RARMNNGAAKPKEPALEPQNQPQQPPQPHLQLHPQAQQPPQPQPQLHHPQSQQPLHPQLQPYTQAP
PQQPLHPQLQPYTQAPPQQPLQPPLQLYPQAQPEQPLQPQSSGSTTGTCVISTAAPSATGTTAAAT
SAPAIPAISTAAPSATPADASAAAAAAATSTPAT

**>TaNFYA-A3_AIZ68348**
MRRKQNMQENGTIMIQFGQQVPNCESSASDSPQEVSGMSEGSFNEQNDQSGNRDGYTKSSDEGKMM
SALSLGNSEMAYTPPKPDRTHPFAISYPYADPYYGGAVAAYGAHAIMHPQMVGMVPSSRVPLPIEP
AAAEEPIYVNAKQYHAILRRRQLRAKLEAENKLVKSRKPYLHESRHQHAMKRARGTGGRFLNAKEK
SEASGGGNASARSGHAGVPPDGGMFSKHDHTLPSGDFHYRARGGA

**>TaNFYA-B5_AIZ68353**
MTSVADGVSGDHRADEQQKQAAAQGNQEEAPATSIGSQAMVATPSTDYVTPYGHQEACHAMGQIAY
PTVDPFYGSLYAAYGGQPMMHPPMVGMHAAAIPLPTDAIEEPVYVNAKQYNAILRRRQSRAKAESE
RKLIKGRKPYLHESRHQHALKRARGAGGRFLNAKSDDNEEHSDSSSKDKQNGVAPRSSGQSSQSPK
GATSADKSGNHE

**>TaNFYA9_A0A3B6JD50**
MSGMGSRPGESNLVEPRGLPSAGMAMQPWWTGTGFGAVSPAVVAPGSGAGISLSSNPFGGGAPQGK
KKTVDDDPRAESSDDDSPRSGEPKDGSFDEEKQHATSRMPALASDYLQPYSQLELNQPMASAAYPY
PDAYYTGMVGPYGAQAVAHFQLPGLTHPRMPLPLEVSEEPVYVNAKQYHGILRRRQSRAKAELERK
AIKARKPYLHESRHQHAMRRARGTGGRFLNTKKDESGGASNGGRTEASKGEQSSEYLRVPPDLHLR
QA

**>TaNFYB-A1_AIZ68332**
MENDGVPNGPAAPAPTQGTPVVREQDRLMPIANVIRIMRRALPAHAKISDDAKEAIQECVSEFISF
VTGEANERCRMQHRKTVNAEDIVWALNRLGFDDYVVPLSVFLHRMRDPEAGTGGAAAGDSRAVTSA
PPRAAPPVIHAVPLQAQRPMYAPPAPLQVENQMQRPVYAPPAPVQVQMQRGIYGPRAPVHGYAVGM
APVRANVGGQYQVFGGEGVMAQQYYGYGYEEGAYGAGSSNGGAAIGDEESSSNGVPAPGEGMGEPE
PEPAAEESHDKPVQSG

**>TaNFYB-D2_AIZ68335**
MSDEAASPPGGGGGGGGGGSDDGGGGGGFGGVREQDRFLPIANISRIMKKAIPANGKIAKDAKETV
QECVSEFISFITSEASDKCQREKRKTINGDDLLWAMATLGFEEYIEPLKVYLQKYRETEGDSKLAG
KSGDVSVKKDALGPHGGASGTSAQGMGQQVAYNPGMVYMQPQYHNGDISN

**>TaNFYB-A3_AIZ68336**
MPESDNDSGGPSNTGGEGELSSPREQDRFLPIANVSRIMKKALPANAKISKDAKETVQECVSEFIS
FITGEASDKCQREKRKTINGDDLLWAMTTLGFEDYVDPLKHYLHKFREIEGERAAATSTSTSTTPD
MPRNNNNNATGYADAPGGMMMMGQPMYGSPPPPQQQQQHQHQIAMGGRAGFPYLGGGGGSSSSSGF
GRKE

**>TaNFYC-D5_AIZ68327**
MENHQLPYTTQPPATGAAGGAPVPGVPGPPPVPHHHLLQQQQAQLQAFWAYQRQEAERASASDFKN
HQLPLARIKKIMKADEDVRMISAEAPVLFAKACELFILELTIRSWLHAEENKRRTLQRNDVAAAIA
RTDVFDFLVDIVPREEAKEEPGSAALGFAAGGVGAAGGGPAAGLPYYYPPMGQPAAPMMPAWHVPA
WEPAWQQGGADVDQGAGSFGEEGQGYTGGHGGSAGFPPGPPSSD

**>TaNFYC-TraesCS7A02G239500**
MEAKSTTPPPPGPVLGAPVGYPPAAVYPAAPPPGYPHAPALYAPQPPPAAVAAASQQTAAQQQQQL
QVFWAEQYREIEATTDFKNHNLPLARIKKIMKADEDVRMIAAEAPVVFARACEMFILELTHRGWAH
AEENKRRTLQKSDIAAAIARTEVFDFLVDIVPRDEAKDAEAAVAAGMPHPAAGMPAADMGYYYVPQ
Q

**>TaNFYC-A7_AIZ68330**
MEPSSQREPVVGVATAGSQAYPPPAAYPAPAMPAAIPPGSQPAVPFPANPAQLSAQHQLVYQQAQQ
FHQQLQQQQQQLREFWATQMEEIEQATDFKNHTLPLARIKKIMKADEDVRMISAEAPVVFAKACE
VFILELTLRSWMHTEENKRRTLQKNDIAAAITRTDIYDFLVDIIPRDDMKEEGLGLPRVGLPPAAL
GAPADAYPPYYYVPAQQVPGVGMMYGGQQGHPVAYAWQQPQGQQAEEAPEEQQQSPSN

**>AtDrAp_At3g12480**
MRKKLDTRFPAARIKKIMQADEDVGKIALAVPVLVSKSLELFLQDLCDRTYEITLERGAKTVSSLH
LKHCVERYNVFDFLREVVSKVPDYGHSQGQGHGDVTMDDRSISKRRKPISDEVNDSDEEYKKSKTQ
EIGSAKTSGRGGRGRGRGRGRGGRAAKAAEREGLNREMEVEAANSGQPPPEDNVKMHASESSPQED
EKKGIDGTAASNEDTKQHLQSPKEGIDFDLNAESLDLNETKLAPATGTTTTTTAATDSEEYSGWPM
MDISKMDPAQLASLGKRIDEDEEDYDEEG

## Figure 3B

**>OsDrAp1-LOC_Os11g34200**
MRKKLGTRFPAARIKKIMQADEDVGKIALAVPVLVSRALELFLQDLIDRTYEITLQSGAKTLNSFH
LKQCVRRYSSFDFLTEVVNKVPDLGGADSCGDDRALPRRRKALPNGSDPENEESRSSKMAVRSANI
SPRGRGRGRGRGRGRPPTKRKEVGYVQFEDESSMFADQGEALPGEETVPETIHGTESVPPSTHPPA
EAPSAAEIPAPNPKVEEAKNDDHQPDWPMPDAIGNIGVGPSGFGHLTVQVDEDEDYDNED

**>OsDrAp2-LOC_Os05g41450**
MRKKLDTRFPAPRIKKIMQADEDVGKIALAVPVLVSKALELFLQDLCNRTYDITVQRGVKTLSSSH
LKQCIHSYNVYDFLRDVVSKVPDMGTSDAGVDDKLGKRRKTAEDDSEEESKRTRNEAASQTSTGRG
RGRGRGRGRRGGRVSEREIISAYEKFEENHEFPPGQFSKPSQLKVDVSVDGTDAIETKEATPLSNA
RASLRNIDLNIELTDYDDEGSAPLEVQPPAPAAGVVTTSSGPLVSEVNEEAKTKDFLGWQLPELTK
MAMDPVQFALSSNHRLEEDEDYDNEE

**>OsDr1-LOC_Os08g29500**
MDPMDIVGKSKEDVSLPKSTMFKIIKEMLPPDVRVARDAQDLLVECCVEFINLLSSESNEVCSRED
KKTIAPEHVLRALQDLGFREYIEEVQAAYEHHKHDTLDSPKASKFTGVEMTEEQAVAEQQRMFAEA
RARMNNGAAKPKEPEPEAQQQTQQPPQPQLHPQPQQPLQPQLQLHPQPQQQPSQLHPQQLLHPQSQ
QTPQPQPQVHPQPQQPPQLQPQPQLLQQPQLPQQLQPQSQLPPQPQQPPQLQLQSQLHPQPQQPPQ
LQPQPQLHQQPQPQAELQSQSQPQTEHGLDSS

**>OsNFYA3-LOC_Os03g44540**
MLSFKQSHEGFGHVAAAGAGPQQQQQPWWAGSQLLYGEASPEEAALRDGGQFQVVPGGRAALDPAA
PEPEKTAVPAMPKRGGGGGAPEVLKFSVFSGNLEPGDTGEKNREHSATIAMQSPLPEYNGHFELGL
GQSMVSPNYPCIDQCYGLMTTYAMKSMSGGRMLLPLNAPADAPIYVNAKQYEGILRRRRARAKAQR
ENRLVKGRKPYLHESRHRHAMRRARGSGGRFLNTKKEATAAGCGGSSKTPLASLVSPADVAHRPGS
GGRASSLSGSDVSSPGGVMYDHHRHDDADAADHYNSIDHHLRTPFFTPLPIIMDSGGGGGDHASHS
AAAVAAPFRWATAAGDGCCELLKA

**>OsNFYA7-LOC_Os08g09690**
MKPDGETQLRPTAAGHPDPGLGTSSAEYVASLGPATAPVSYPYISTYYGGTYGAYSGQPLVNAALM
AMPPHSVPLVTDAVVEPIYVNARQYHGILRRRQSRAKAESENKANKIRKPYLHESRHLHALKRARG
SGGRFLNSKAVEGKQDTKSVDKKDGAVPSEEKRDKKLANSIIKLENSSPTTQPGADASDVV

**>OsNFYA8-LOC_Os10g25850**
MGFGESTQGNQRKLDGPGKVSTELSLVNLEAKNLHPKPECNQPIEHIPTKGMKCTPLLPLPTEHAD
DEPIYVNAKQYHAIIRRRQRRKIVGSEDKVAAIRKRILVEARQKQAKLRHRGKGGRFISIEHPLEL
SMDDQISKNGGSASPSSSTVSENSSNVNGFTGDL

**>OsNFYB1-LOC_Os02g49410**
MAGNKKRGGRNMDQVKKAAVRSDGVGGSATNAELPMANLVRLIKKVLPGKAKIGGAAKGLTHDCAV
EFVGFVGDEASEKAKAEHRRTVAPEDYLGSFGDLGFDRYVDPMDAYIHGYREFERAGGNRRVAPPP
PAAATPLTPGGPTFTDAELQFLRSVIPSRSDDEYSGSSPAIGGYGYGYGYGKNM

**>OsNFYB5-LOC_Os01g70880**
MTNGQDNLLPIANVGRIMKDGLPPQAKISKRAKETIQECATEFISFVTGEASERCRRERRKTVNGD
DVCHAMRSLGLDHYADAMHRYLQRYREGEELAASLNSSSSAAAAAAAAGSRGGGAIQIDVRAELSI
FRSGNNQGRPNN

**>OsHFYB8-LOC_Os03g29970**
MPDSDNDSGGPSNYAGGELSSPREQDRFLPIANVSRIMKKALPANAKISKDAKETVQECVSEFISF
ITGEASDKCQREKRKTINGDDLLWAMTTLGFEDYVDPLKHYLHKFREIEGERAAASTTGAGTSAAS
TTPPQQQHTANAAGGYAGYAAPGAGPGGMMMMMGQPMYGSPPPPPQQQQQQHHHMAMGGRGGFGHH
PGGGGGGSSSSSGHDEDEDEDEDEDED

**>OsNFYC1-LOC_Os02g07450**
MEPSSQPQPVMGVATGGSQAYPPPAAAYPPQAMVPGAPAVVPPGSQPSAPFPTNPAQLSAQHQLVY
QQAQQFHQQLQQQQQQLREFWANQMEEIEQTTDFKNHSLPLARIKKIMKADEDVRMISAEAPVVF
AKACEVFILELTLRSWMHTEENKRRTLQKNDIAAAITRTDIYDFLVDIVPRDEMKEEGLGLPRVGL
PPNVGGAADTYPYYYVPAQQGPGSGMMYGGQQGHPVTYVWQQPQEQQEEAPEEQHSLPESS

**>OsNFYC2-LOC_Os03g14669**
MDNQQLPYAGQPAAAGAGAPVPGVPGAGGPPAVPHHHLLQQQQAQLQAFWAYQRQEAERASASDFK
NHQLPLARIKKIMKADEDVRMISAEAPVLFAKACELFILELTIRSWLHAEENKRRTLQRNDVAAAI
ARTDVFDFLVDIVPREEAKEEPGSALGFAAGGPAGAVGAAGPAAGLPYYYPPMGQPAPMMPAWHVP
AWDPAWQQGAAPDVDQGAAGSFSEEGQQGFAGHGGAAASFPPAPPSSE

**>OsNFYC7-LOC_Os09g30310**
MDPTKSSTPPPVMGAPVGFPPGAYPPPPPAGAAAAAYAQQLYAPPAAAAAQQAAAAQQQLQMFWAE
QYREIEATTDFKNHNLPLARIKKIMKADEDVRMIAAEAPVVFARACEMFILELTHRGWAHAEENKR
RTLQKSDIAAAIARTEVFDFLVDIVPRDDAKDADAAAAAAAAGIPRPAAGVPATDPLAYYYVPQQ

## Figure 3C

**>TaDrAp1-A4**
MRKKLGTRFPAARIKKIMQADEDVGKIALAVPVLVSRALELFLQDLIDHSYKITLQSGAKTLNSFH
LKQCVKRYSSFDFLTEIVNKVPDLGGGESCGDERGLPRRRKFSNGSDPENEEPRSSKMPIRSLNTS
PRGRGRGRGRGRGRPPTKRKEIGYVQFEDESSMFAEQSEPLPGDEIVPETNRGNESIPQSSHPLVE
APSAMTPAVISKVEEASTNHQPDWPMPDAIGGIGVGPSSFGHLTVQVDEVEDYDNED

**>TaDrAp1-B4**
MRKKLGTRFPAARIKKIMQADEDVGKIALAVPVLVSRALELFLQDLIDRSYKITLQSGAKTLNSFH
LKQCVKRYSSFDFLTEIVNKVPDLGGGESCGDERGLPRRRKFSNGSDPENEEPRSSKMPIRSLNTS
PRGRGRGRGRGRGRPPTKRKEIGYVQFEDESSMFAEQSEPLPGDEIVPETNRGNEIIPQSSHPLVE
APSVVTPAVISKVEKATNHQPDLPMPDAIGGIGVGPSSFGHLTVQVDEEEDYDNED

**>TaDrAp1-D4**
MRKKLGTRFPAARIKKIMQADEDVGKIALAVPVLVSRALELFLQDLIDRSYKITLQSGAKTLNSFH
LKQCVKRYSSFDFLTEIVNKVPDLGGGESCGDERGLPRRRKFSNGSDPENEEPRSSKMPIRSLNTS
PRGRGRGRGRGRGRPPTKRKEIGYVQFEDESSMFAEQSEPLPGDEIVPETNRGNESIPQSSHPLVE
APSAATPAVISKVEKASTNHQPDLPMPDAIGGIGVGPSSFGHLTVQVDEEEDYDNED

**>TaDrAp2-A1**
MRKKLDTRFPAPRIKKIMQADEDVGKIALAVPVLVSKALELFLQDLCDRTYNITVEKGVKTVSSSH
LKQCIHSYDVYDFLKNVASKVPDLGAPDSSVDDKLGKRRKHGEDESEEESKRTRNEAACHTSNGRG
RGRGRGRGRRAGRGAEREIEHHELGCAQFSKPGHLKVEIGDGVLDTSETKEHTPLSNARASLRNID
LNLDLADYEEDTVAPQVQPSAPVVTVAPQVQTSGPSVSQLKEEVATKDFLGWQMPEMNKMAMDPVQ
FALSSNHRLEEDEDYDNEE

**>TaDrAp2-B1**
MRKKLDTRFPAPRIKKIMQADEDVGKIALAVPVLVSKALELFLQDLCDRTYNITVEKGVKTVSSSH
LKQCIHSYDVYDFLKNVSSKVPDLGPPDSSVDDKLGKRRKHGEDESEEESKRTRNEAACHTSNGRG
RGRGRGRGRRAGRGAEREIEHHELGCAQFSKPGHLKVEIGDVVLDTSETKEHTPLSNARASLRNID
LNLDLADYEEDTVAPQVQPSAPVVTVAPQVQTSGPSVSQLKEEVETKDFLGWQMPEMNKMAMDPVQ
FALSSNHRLEEDEDYDNEE

**>TaDrAp2-D1**
MRKKLDTRFPAPRIKKIMQADEDVGKIALAVPVLVSKALELFLQDLCDRTYNITVEKGVKTVSSSH
LKQCIHSYDVYDFLKNVASKVPDLGAPDSSVDDKLGKRRKHGEDESEEESKRTRNEAACHTSNGRG
RGRGRGRGRRAGRGAEREIEHHELGCAQFSKPGHLKVEIGDVVLDTSETKEHTPLSNARASLRNID
LNLDLADYEEDTPQVQPSAPSAPVVTVAPQVQTSGPSVSQLKEEVETKDFLGWQMPEMNKMAMDPV
QFALSSNHRLEEDEDYDNEE

**>OsDrAp1-Os11g34200**
MRKKLGTRFPAARIKKIMQADEDVGKIALAVPVLVSRALELFLQDLIDRTYEITLQSGAKTLNSFH
LKQCVRRYSSFDFLTEVVNKVPDLGGADSCGDDRALPRRRKALPNGSDPENEESRSSKMAVRSANI
SPRGRGRGRGRGRGRPPTKRKEVGYVQFEDESSMFADQGEALPGEETVPETIHGTESVPPSTHPPA
EAPSAAEIPAPNPKVEEAKNDDHQPDWPMPDAIGNIGVGPSGFGHLTVQVDEDEDYDNED

**>OsDrAp2-Os05g41450**
MRKKLDTRFPAPRIKKIMQADEDVGKIALAVPVLVSKALELFLQDLCNRTYDITVQRGVKTLSSSH
LKQCIHSYNVYDFLRDVVSKVPDMGTSDAGVDDKLGKRRKTAEDDSEEESKRTRNEAASQTSTGRG

RGRGRGRGRRGGRVSEREIISAYEKFEENHEFPPGQFSKPSQLKVDVSVDGTDAIETKEATPLSNA
RASLRNIDLNIELTDYDDEGSAPLEVQPPAPAAGVVTTSSGPLVSEVNEEAKTKDFLGWQLPELTK
MAMDPVQFALSSNHRLEEDEDYDNEE

**>BdDrAp1-Bradi4g16840**
MRKKLGTRFPAARIKKIMQADEDVGKIALAVPVLVSRALELFLQDLIDRSYNITVQSGAKTLNSFH
LKQCVKRYNSFDFLTEIVNKVPDLGGADSCGDERGLPRRRKLSNESDPENEEPRSSKMPIRSLNTS
PRGRGRGRGRGRGRPPTKRKEVGYVQFEDESSMFTEQSESLPGDDAIPETKCGSESIPQSANPPAD
APSTGVPAAISKVEEASTNHQPDWPMPDAIGGIGVGPSSFGHLTVQVDEDEDYDNED

**>BdDrAp2-Bradi2g21290**
MRKKLDTRFPAPRIKKIMQADEDVGKIALAVPVLVSKALELFLQDLCDRTYNITVQKGVKTVSSSH
LKQCIHSYDVYDFLKNVVNKVPDLGAPDTSADDKLGKRRKHAEDESEEESKRTRNEVASHASNGRG
RGRGRGRGRRGGRGAERETEYHELAPCESKPVNLKVEIGDVGSDTIETKEPTPLSNARASLRNIDL
NLDLDEYEDTVAQFQPEAPVASVAAPSAGPSVSQSIEDVKTKDFLGWQLPEMSKMGMDPVQFALSS
DHRLEVDEDYDNEE

**>SbDrAp1-8057006**
MRKKLGTRFPAARIKKIMQADEDVGKIALAVPVLVSRALELFLQDLIDRTYEITLQSGAKTLNSFH
LKQCVKRYSSFDFLTEVVNKVPDLGGADSCGDERGLPRRRKSNGSDPENDESRSSKMVIRNANISP
RGRGRGRGRGRGRPPTKRKEVGYVQFEDESSMFAEQGEPLPGEETVQEINNGNEIMPQSTQPPVEA
PPTAPAQATSSKVEEANTNHQSDWPMPDAIGSIGVGPSGFGHLTVQVDEDEDYDNED

**>SbDrAp2-8077043**
MRKKLDTRFPAPRIKKIMQADEDVGKIALAVPVLVSKALELFLQDLCDRTYDITIRKGVKTVGSSH
LKQCIQTYNVYDFLTEVVSKVPDIGPSDVIADDKLGKRRKAEEDGSEEELKRTRNETESYTSNGRG
RGRGRGRGRRGRGAWREVVTTHEQFAENQSSKLASLKVEVADEVSNATEAKEATTPVSNARASIRN
IDLNLDPTDDEDDVTVPPQAQLSAPATSSAAATAGPGPSAAGPGPSVPRSKEGAKLKDFLGAWGLP
DMSNMEMDPVQFALSTNHRVEEDEDYDNED

**>ZmDrAp1-100304013**
MRKKLGTRFPAARIKKIMQADEDVGKIALAVPVLVSRALELFLQDLIDRTYEITLQSGAKTLNSFH
LKQCVKRYSSFDFLTEVVSKVPDLGGADSCGDERVLPRRRKSNGSDPENDESRSSKMAIRNANTSP
RGRGRGRGRGRGRPPTKRKEVGYVQFEDESSMFAEQGETLPGEGTVPEINSGNEITPQSTQPPLTA
PAQATNSKVEEASTDHQSDWPMPDATGNIGVGPSGFGHLTVQVDEDEDYDNED

**>ZmDrAp2-100284240**
MRKKLDTRFPAPRIKKIMQTDEDVGKIAQAVPVLVSKALELFLQDLCDRTYDITIRKGVKTVGSSH
LKQCIQTYNVYDFLREVVSKVPDIGPSDVIADDKLGKRRKAEEDGSEEELKRTRNETESYTSNGRG
RGRGRGRGRRGVRGAWREVVTSPEQFKEENQSSKVASLKVEVADEVSNVTEAKQDTTPMSNARPSL
RNIDLNLDPTDEEDEVTVPPQPQLSAPATSCDAATAAPGPSVPRLREGAKLKDFFGAWELADMNKM
EMDPVQFALSTNHRLEEDEDYDNED

**>Bv-104887609**
MRKKLDTRFPAARIKKIMQADEDVGKIAMAVPVLVSKALELFLQDLCDRTYDITLQRGAKTMSSLH
LKNCIHSFSMFDFLKDVVSKVPDYGNSDANADDRTAPKRRKSAVDEYHDSDEDSKKSRMQEITHAS
SSGRGRGRGRGRGRGRGRPPLERDPPSHHEMEAEPSTSFEPINKPSPIQVPNTDDDPNKPDPMKES
PVVGDGGDAAIRNFDLNAAVAENQDIGPTVTAAENAAVASADPAPESKNEELVSEVDGMAIDPHQI
AQFNSRIEDDEDYDEE

**>Sl-101246965**
MRKKLDTRFPAARIKKIMQADEDVGKIAMAVPVLVSKALELFLQDLCDRTYDITLRRGAKTVNSLH
LKHCVQSYNVFDFLREVVSKVPDYGHSDAAGEMPKRRKVAIEEHHDSEDEYKKSRTEMSPVGSSGR
GRGRGRGRGRGRVSRADKELSRPDMQLESCTSAQQSVQQNPNPGTQTENCSEPKESPTQDSTVCDK
ENSVVTTPNLKVNVDDNTDKPAAPEIAPCNPSPRPVNEKAEEGPQWSLEMDRMVIDPAHMSQLNTS
VAEEEEDYDEEE

**>Ha-110930899**
MRKKLDTRFPAARIKKIMQADEDVGKIAMAVPLLVSKALELFLQDLCDRTYEVTLQRGAKTLNSTH
LKQCVQSFNVFDFLRDIVGKVPDLGGSDAAGEDRSAPKRRKVADGDHNDGDEIEDSKRDKRPEILH
ANGSGRGRGRGRGRGRGRPSRADKEAYAAAAAAYDKHETNSSISDKPKPNSTLDSLEDADSKENQN
LAGVKAEDTVKVRNFDLNLDLDENGNSPVIPAVTPPKQAVEMEMKPHEYPGWSMTDVEKMVVDPLK
LASFNSRVDEDDEDYDEEG

**>Vv-100253016**
MRKKLDTRFPASRIKKIMQADEDVGKIALAVPLLVSKALELFLQDLCDRTYQITLERGAKTMSSLH
LKQCVQRFNVFDFLREIVSKVPDLGCSDAGGEDRSASKRRKVVDDEGNDSDEESKRSRMHETGHTV
GSGSGSGSGRGRGRGRGRGRGRGSRTIERDTIAESEKPEDDTDMHTNNDNQSQNPETRDNGAEPEE
SKENISVVKNESKENISVGKNADSCVRNFDLNVDLDENGDSTSILPAAPAPAPVTPSPKLTEMKHE
EYPGWSLSDMEKMEIDPIQLANLNRRIDEDEEDYDEE

**>Cs-101209714**
MRKKLDTRFPAARIKKIMQADEDVGKIALAVPVLVSKALELFLQDLCDRTYEITLQRGAKTMNSLH
LKHCVQSYSVFDFLRDIVGRVPDYGHGHSDGAVDDRKRRKPLGDEGNDFDELKKSKMHDMSHVSTG
RGRGRGRGRGRGRPARSAERDNYQPNPDVDPCTSIENRNKNPNPDVHMEHHVEPSDTPKEVSEANQ
AIQNFDLNADVSTNEDSKTVPAAAVSDAPIEPAAPTTTTESETKAADEFPAWPLSDVDKMAIDPLQ
YTHLSSRVDEEEEDYDEEE

**>Gh-107927594**
MRKKLDTRFPAARIKKIMQADEDVGKIALAVPVLVSKALELFLQDLCDRTYEITLQRGAKTMNSLH
LKHCVQSYNVFDFLREIVSRVPDYGHGHGHSDAVAAAAADDDRSISRRRKAAGDEVHDSDEESKRS
RIHDVGHVGTIGRGRGRGRGRGRGRGGRNVERDAHREIEPEPCATLQQNSKSHPTPGMVVDDGSES
KEPIKENTIAEDANQAVRNFDLNAEVDENVEAKPTVGAATAAALEPATAAAAAAQPSSAEPITETK
HEEYPGWSLSDMDKMAIDPLQLAQLGKRLDEDEEDYDEEG

**>Ca-101513228**
MKKKLDTRFPAARIKKIMQADEDVGKIALAVPVLVSKALELFLQDLCDRTYEITLQRGAKTMNSLH
LKHCVQSYNVFDFLKDVVSKVPDYGHGHGHTDAGAADDQAISKRRKAIGDDCNDSDEEAKRGKMVE
LSHASPTGRGRGRGRGRGRGRGRGRGSRATLREGHLPETESEPFPPVQQVNQHTTDTNVEGKHDDVTH
SESKEELPKENIVAPVENSDSLRNIDLNANMNETDDKKASAVANPAPAATNPAPTTANTSLSEPPH
IDDSNHEEMPGWSLSEVDKMAIDSMQLAQLGTRMEEDDEDYDEEG

**>AtDrAp-At3g12480**
MRKKLDTRFPAARIKKIMQADEDVGKIALAVPVLVSKSLELFLQDLCDRTYEITLERGAKTVSSLH
LKHCVERYNVFDFLREVVSKVPDYGHSQGQGHGDVTMDDRSISKRRKPISDEVNDSDEEYKKSKTQ
EIGSAKTSGRGGRGRGRGRGRGGRAAKAAEREGLNREMEVEAANSGQPPPEDNVKMHASESSPQED
EKKGIDGTAASNEDTKQHLQSPKEGIDFDLNAESLDLNETKLAPATGTTTTTTAATDSEEYSGWPM
MDISKMDPAQLASLGKRIDEDEEDYDEEG

**>Dc-108193389**
MRKKLDTRFPAARIKKIMQADEDVGKIAMAVPVLVSKALELFLQDLCDRTYDITLQRGAKTVSSLH
LKHCVQRYNVFDFLKEVVSKVPDYTHTEAGGDDVNMTKRRKITSEEANDSDEESKRNRVHEPSQVS
SSGSKGRGRGRGRGRGRGRGVRSADKEAAQTDFDYDSCNNNRILVPVAATEVADDASELKEVPKDS
INISNNSNQASRNFDLNADLDDNAIDKTPTEAPSLPSATPTAPRSEDEYPGWPLSEMDRIPIDPVD
LAKLNSRMDDDDEDYDEEG

**>HsDrAp1-10589**
MPSKKKKYNARFPPARIKKIMQTDEEIGKVAAAVPVIISRALELFLESLLKKACQVTQSRNAKTMT
TSHLKQCIELEQQFDFLKDLVASVPDMQGDGEDNHMDGDKGARRGRKPGSGGRKNGGMGTKSKDKK
LSGTDSEQEDESEDTDTDGEEETSQPPPQASHPSAHFQSPPTPFLPFASTLPLPPAPPGPSAPDEE
DEEDYDS

**>DmDrAp1-CG10318**
MPSKKKKYNARFPAGRIKKIMQSDEEIGKVAQAVPVIISRTLELFVESLLTKTLRITNARNAKTLS
PSHMRQCIVSEKRFDFLKELVRNIPDISVAEEAAYNEDDVLRSSPEEQYPDSDTPYDLSLPSTSMR
SQANGTAAYMRSMSLNNGAGSGGGAAATKRQFQSQHSTQETPTTSTTLPAKLARSGSMPAYTPRGR
PPNHLKKQSVQLDTAIPAPICNYELNKPIVKIDYSHVQMPTANLCTPDASDSGSGSGAGSGAFNFD
IAAPVINIDLTNIVAGGAPGSGVPPASIGLPAATPGDNNNVNQMAGKSSAPIIPKATAASATPTET
VFELDEDYDNI

# Supplementary file 3

## Promotor regions and primer design

### *TaDrAp1*

***>TaDrAp1-A4***

ATCTGGTAGTTTGGGGGTACGTAGGTATACATAGGAGGAAGGAGTACGTTGATGGATCAATAGGGG
GCCCACGAGGGTAGAGGGCACGCCCAGGGGGGTAGGCGCGCCTCCCTACCTCGTGGCCTCCTGGTT
GATGTCTTGACGTAGGGTCCAAGTCCTCTGGATCATGTTCGTTCCGAAAATCACGTTTCCGAAGGT
TTCATTCCGTTTGGACTCCGTTTGATATTATTTTTGTGCGAAACTCTGAAATAGGCAAAAAACAAC
AATTTTGGGCTGGGCCTCCGGTTAATAGGTTAGTCCCAAAAATAATATAAAAGTGTATAATAAAGC
CCAATAATATCCAAAAAGAATATAATATAACATGAAACAATAAAAAAATATAAATACGTTGGAGAC
GTATTAGTGATTAGGTACCAAGATGATGGCCATAGAGCAGCTAGGAGCAATAGACAAGAAGGCAAG
AAAGAGGAGCAAAAGAAGCAAGGAAAGGAATTGCTAGCAAGCTACATGGACCAAGCGTCCGACACG
TTCACTTATAGCCAACCACAAAGCCAACAGCGGATGAACGTTACAGCGGCCGGACGTCCGACAAGT
TCCAGCAACCGGCCGTCCGGTCTTCACCAGAAATCCTGTACCACATGTCCGGAAGCAAACTGACGG
AAGTCCGATGCGCACTGGACATCCGAACACTTCCGAGCCACCAGACGTCCGGCAAACACCGGAAAT
CAAGTACCACCTGACCAGAGAAGAAAATGGCGGAAGTCCGGCGCCCAACCGGACGTTCGGTCACTC
GCGAACGACTGGGCGTTCGGTACTGCATGTGCACATCCCCACGGTTTTCAGCCCATGTACCCCTCT
TTCACCCCTAGGCTATATACCCTTCTCCTACATCCATCATAGGGCTGGCAAAGACATAATAGAGAA
TTAGTAGAGCTTTGCCCATACTCATTATTCCTCTTGGAGATCAAGACCTCCATGCGAGAACATCCC
CTAGTGGATGCAAGACCCATATAAGGGAAGATCCATCTAGGGATGCAAGACCCCTATATGGGAAGA
TCCTCCATGGATGCTAGATCTCATCTCCTTTGGATTTGAGAAGAACCTTTACCTTTGTGCTTTCCC
CTTTGACTTGTTCTTGTATATTTGTGGATCCCATAGTGATGTGTGATTTGGGTTTGTTTGAGTCTC
TCTCTCTCTCTCGTGTTCTTGAATGCTCATCCTTTCCCCACCTCCAAGTGCGAAAAGATCTCAA
GTCTAGGGTTTTGCCCTACATCACCGTGCCTCCCCATCCCTTCCTCTCCTCTCCTCTTTCCTTTCT
CGTAGGTCACTGACCTCCTACTCGTGTCAGGTCCCTCTCCTCCTTCCTTCCTCGCAGGTCGTCGAG
CAAGGAATCGAGCTGGGCAAACGCGACGGCCGGTCGGATCAATGCCGAATTGACCGGATCCGACGC
AGGGATCACAACAAGGAGGAGAAAGTCGGAGGAAGGAGCGGTGGCGGGCAGGGGCGGCAACCATGG
TGGCGTTGGTGGCAGGAGTGGCAATGGAGGCTCCGCCGACCACGACGAGGAAAGCGGGATCTGCAG
TGAGCTTCGCGGGGTTGGCTGCGGGGGTCAGAGGAGCTGGCCATGGCGACGCTGTGGCCAGCGGGC
GATCAGAGGCGGCGTTCCGGTTGCGCGCGGGAAAAAGGTAGCGATTGGGTTTCGCGATGCAACCGC
TCTTTTTTTGTGGCAGCCGCCCTACTACTCTATATTCGCTGCACTTGAGGACTTTTTGTTGGCTTC
CCCAAATATTTTTTTTCAAAAATATTAGAATTTTTTAGAGGAAAAAAAAAGAATAAAATAGCCTTT
CATGCTCTAAAAACAACTTGTGCCTGAAAATTATAGGGCTTGCAGTGGGAGACGCGGCAAATATCT
TTTTGGCACGAGTCGGTGTGCCCCGTGCCGTGGTGGTGCAAGTTGGTGGAAAGACAACAAAACAAG
AAATCCAATCGTCAAAGAAGAACGAGAAAAGAAAATACACAAAAGATAGACCACGATTGCCGGCAT
CGAGCCCGACGAGGCGTCGGAGGTGCGACAAAGATGAGGAAGAAGCTGGGCACCCGGTTCCCCGCG

***>TaDrAp1-B4***

CAACCCTATCTCTTGTTTCCCCACACATACGGACTCTAAAAACAGCCTATACTCTGGTATTTTGAA
ATTACATGGACCTGACCCAATAATAAGGTGACGCAATACCTATAATAGCCTCTGGACGAAATTTAG
GAAGTGACATCTTGTATATTTCATCCGAGCCTTGATACGCTCATTGTGGTGGCTTCAAAGTCCTGA
AATCGTCACTTGAAACTCAGTCTTGTTTTCCTTGCGCATGCCATCATCTCCATGCTTGATCTTGCT
CAAGTGATCATCCCTCTTGTCCATGCTAGACCCTTTATTTGTAAGCAAAACAAATGTATCCAATTT
AGGCATCATCATATTCTCATGAACATTAGAAACATTACCAAGAAACAAAAGTACCTAATAATTTAA
TTGGCGCGCGCGAGCTCTAGTAATTTGTCCAGTATACGTAGCAGTAGGGGCTGTGGGTGTAACAAT
GATATTGATGTCCTCATCAAGAAGCAAGGAAAGGAGTTGGTAGGAAGCTACAGGCACCGGACGTCT

CACACATTCACTTACAACCAACCACAAAGCCAACAACCAACGAACGCTACAGCGGCCGGACATCCG
ACAAGTTCCAACAACCAGACGTCCGGTCTTCACTAGAAATGTTGTACCACATGTCCGGAAGCAAAA
TGATGGAAGTCTGACGCGCATTGGACATTTGGACCCTCCCGAGCCACCAGACGTCCGGCAAACACC
GGAAATCCCGTACCACCTGACCAGAGAAGAAATGGCGGAAGTCCGACGCCCAACCAGATGTTCAAT
CACTCGCGAACGACCAGACGTCCGGTATTGCGTGTGCGCATCCCGACGGTTTTCAGCCCATGTACC
CCTCTTTCACACCTAGACTATAAATACCCTTCTCCTACCTCCATCCTAGGGCTGGCAAAGACATAA
TAGAGAATTGCTAGAGCTTTGCTCATATTCATTATTCCTCTTGGAGGTCAAGACCTCCATGTGAGA
AGATCCCCCTAGTGGATGCAAGAGCCCTATAAGGGAATATCAATCTAGGGATGCAAGACCCCTATA
TGGGAAGATCCTCCATGGATGCTAGATCTCATCTCCTTTGGATTTGAGAAGAACCTTTACCTTTGT
GCTTCCCCCTTTTGACTTGTTAATATATATTTGTGGATCTCATGTATGCTATCTTAGTGATGTGTG
ATTTGGATTTGTTTGAGTCTCTCTCTCTCTCTCTTGTGTTATTGAGTGTTCATCCTTTCTCCATCC
TCCAAGTGCGAAAAGATCTTGAATCTAGGGTTTTGCCCTACATCACCATGCCTCCCTAGCCCCCCT
CTCCTCTCCTCCTTCCCTTCCCGCAGGTCACTGACCTCCTACTTGTGTCGGGTCCCTCTCTTCCTT
CCTTCCCCGTAGGTCGTTGAGCAAGGAATCGAACTGGGCAAACGTGACGGCCGGCCGGATCGATGC
TGAATTGACCGGATCCGACGTAGGGATCACAACAAGGAGGAGGAAGTCGGAGGAAGGAACGGTGGC
GGGCAGGGGCGGCGGCTACAATGTCGTCGGTGGAAGGAGCGACAATGGAGGCTCCGCCGACCATGG
CGAGGAAAGCGAGATCGGCGATGAGCTTCGCGGGGTCCGCTGCGGGGTCAGAGGAGCTGGCCATGG
CGACGCGGTGGCCGGCGGGAAAGCGGAGGCAACATTCCGGTTGCGCGCGGGAAGAAAAGGGTAGCG
ATTATTTTTCGTGATGCAAGCACTCTTTTGTGGCAGTCGCGTGACTACCCTATATTCGCTACACTC
GAAGCATTTTTTTTAGGTTGCCGGATTTTTTCAAGAAAATTTAGAATTTGATAGCTTTTCGTGCTT
AAAAACAATTCGTGTGTGAAAATAATAGGGCTACTGTGAGAGATGCGGTAAATATCTTTTGGCACG
AGTCCGCCGTGTTGGTGCAAGTTGGTGGAAAGACAACAAAACAAGAAACCCAATCGTCAAGAAGAA
CGAGAAAAGAAAATACACAAAAGGTAGACTACGATTGCCGGCATCGAGCTCGACGAGGCGTCGGAG
TTGCGACAAAGATGAGGAAGAAGCTGGGCACCCGGTTCCCCGCG

### *>TaDrAp1-D4*

GCATGTTTTATTATTGGTGTTGTCCTATGGTGCTCTCCGTGTCGCGCAAGCGTGAGGGATTCCCGC
TGTAGGGTTTGCAATATGTTCATGATTTGCTTATGATGGATGGCATGAGTGACAGAAGCATATACC
CGAGTATGTAGGTTGTTTGCGTATGGGAATAAAGAGGACTTGATACTTTAATGCTATGGTTGGGTT
TTACCTTAATGATCTTTAGTAGTTGCGGATGCTTGCTAGAGCTCCAATCATAAGTGCATATGATCC
AAGTAGAAATTATGTTAGCTTATGCCTCTCCCTCAAATAAAATTGCAATAGTGATTACCGGTCTAG
TTATCGATTGCCTAGGGACAAATAACTTTCTCGTGACAAAAAGCTCTCTACTAAAACTAATTTAGT
TGTGTCTTTATCTAAATAGCCCCTACTTTTTATTTACGTGCTCTTTATTATCTCGCAAACCTATCC
GAAAACACCTACAAAGTACTTCTAGTTTTATACTTGTTCTAGGTAAAGCGAACGTCAAGCGTGCGT
AGAGTTGTATCGGTGGTCGATAGAAGTTGAGGGAATATTTGTTCTACCTTTAGCTCCTCGTTGGGT
TCGACACTCTTACTTATCGAAAGAGTCTACAATTGATCCCCTATACTTGTGGGTTATCAAAGCTAC
ATGCACCGGACGTCCGACACGTTCACTTACAGCCAACCACAAAGCCAATAGCCGACGAACGCTACA
GCGACCGGACGTCCGACAAGTTCCAGCAATCGGACGTCCGGTCTTCACCAGAAATCCTGTACCACA
TGCCCGGAAGCAAAATGACGGAAGTCCGACGCGTACGGGATGTCTGGACCCTCCCGAGCCACTAGA
CGTCCGGCAAACACCGGAAATCCCGTACCACCTGACCGGAGAAGAAATGGCGGAAGTCCGATGCCC
AACCGGATGTTCGATCACTCGTGAACGACCGAACGCCCGGTACTCCCTGTGCGCATCCCGACGGTT
TTCAGCCCATGTACCCCCTTTTCATCCCTAGACTATAAATACCCTTCTGCTAGCTCCATCCTAGGG
CTGGCAAAGACATAATAGAGAATTGATAGAGCTTTGCTCATATTCATTATTCCTCTTGGAGGTCAA
GACATCCATGTGAGAAGATCCCCTAGTGGATGCAAGAGCCCTATAAGGGAAGATCCATCTAGGGAT
GCAAGACCCCTATATGGGAAGATCCTCCATGGATGCTAGATCTCATCTCCTTTGGATTTGAGAAGA
ACCTTTACCCTTGTGCTTCCCCCTTTTGACTTGTTCTTGCATATTTGTGGATCTCATGTATGCTAT
CTTAGTGATGTGTGATTTGGATTTGTCTGAGTCTTTCTCTCTCTTGTGCTCTTGAGTGTTCATCCT
TTCTCCACCTCCAAGTGCGAAAAGATCTCAAATCTAGGGTTTTGCCCTACATCACCGTGCCTCCCT
AGCCCCTCCTCTCCTCTCCCCCTTCCTTCCCCGCAGGTCACTGACCTCCTACTCATGTCGGGTCCC
TCTCCTCCTTCCTTCCCCGTAGGTCGTTGAGCAAGAAATCGAGCTGGGCAAACGTGACGGCTGGCC

GGATCAATGCCGAGTTGTCCGGATCCGACGCAGGGATCACAACAAGGAGGAGGAAGTCGGAGGAAA
GAGAGGTGGCGGGCAGAGGCGGCGGCGACGGTGTCGTCGGTGGAAGGAGCGGCAATGGAAGCTCCG
CCGACCATGGCGAGGAAAGCGAGATCGGCGGTGAGCTTCGCGGGGTCGGTTGCAGGGCCAAAGGAG
CTGGCCATGGCGACGTGATGGCCGGCGGGAGAGCGGAGGCAGCGTTCCGGTTGCGCGCGGGTAGAA
AAAGGTAGCTATTGGGTTTCATGATGCAACCACTCTTTTGTGACAGCTGCCCGACTACTCTATATT
CGCTGCACTGAGGATTTTTAAAAAAAATTAGAATTCGGTAGCCTTTCGTGCTTAAAAACAATTCGT
GTGTGAAAATAATAGGGCTGGGAGATGCGGTAATTATCTTTTGGCACGAGTCCGCGTGCCGTGTAA
GTTGGTGGAAAGACAACAAAACAAGAAATCCAATCGTCAAGAAGAACGAGAAAAGAAAATACACAA
AAGATAGACTACGATTGCCGGCATCGAGCTCGACGAGGCGTCGGAGGTTGCGTCAAAGATGAGGAA
GAAGCTGGGCACCCGGTTCCCCGCG

# 1KB Promoter regions:

```
TaDrAp1-B4      TCACTAGAAATGTTGTACCACATGTCCGGAAGCAAAATGATGGAAGTCTGACGCGCATTG
TaDrAp1-D4      TCACCAGAAATCCTGTACCACATGCCCGGAAGCAAAATGACGGAAGTCCGACGCGTACGG
TaDrAp1-A4      TCACCAGAAATCCTGTACCACATGTCCGGAAGCAAACTGACGGAAGTCCGATGCGCACTG
                **** ******  *********** *********** *** ******* ** *** *  *

TaDrAp1-B4      GACATTTGGACCCTCCCGAGCCACCAGACGTCCGGCAAACACCGGAAATCCCGTACCACC
TaDrAp1-D4      GATGTCTGGACCCTCCCGAGCCACTAGACGTCCGGCAAACACCGGAAATCCCGTACCACC
TaDrAp1-A4      GACATCCGAACACTTCCGAGCCACCAGACGTCCGGCAAACACCGGAAATCAAGTACCACC
                **  *  * ** ** ********* *************************  ********

TaDrAp1-B4      TGACCAGAGAAGAAA-TGGCGGAAGTCCGACGCCCAACCAGATGTTCAATCACTCGCGAA
TaDrAp1-D4      TGACCGGAGAAGAAA-TGGCGGAAGTCCGATGCCCAACCGGATGTTCGATCACTCGTGAA
TaDrAp1-A4      TGACCAGAGAAGAAAATGGCGGAAGTCCGGCGCCCAACCGGACGTTCGGTCACTCGCGAA
                ***** ********* *************  ******** ** ****  ******* ***

TaDrAp1-B4      CGACCAGACGTCCGGTATTGCGTGTGCGCATCCCGACGGTTTTCAGCCCATGTACCCCTC
TaDrAp1-D4      CGACCGAACGCCCGGTACTCCCTGTGCGCATCCCGACGGTTTTCAGCCCATGTACCCCCT
TaDrAp1-A4      CGACTGGGCGTTCGGTACTGCATGTGCACATCCCCACGGTTTTCAGCCCATGTACCCCTC
                ****    **  ***** * * ***** ****** ***********************

TaDrAp1-B4      TTTCACACCTAGACTATAAATACCCTTCTCCTACCTCCATCCTAGGGCTGGCAAAGACAT
TaDrAp1-D4      TTTCATCCCTAGACTATAAATACCCTTCTGCTAGCTCCATCCTAGGGCTGGCAAAGACAT
TaDrAp1-A4      TTTCACCCCTAGGCTAT--ATACCCTTCTCCTACATCCATCATAGGGCTGGCAAAGACAT
                *****  ***** ****  ********** ***  ****** ******************

TaDrAp1-B4      AATAGAGAATTGCTAGAGCTTTGCTCATATTCATTATTCCTCTTGGAGGTCAAGACCTCC
TaDrAp1-D4      AATAGAGAATTGATAGAGCTTTGCTCATATTCATTATTCCTCTTGGAGGTCAAGACATCC
TaDrAp1-A4      AATAGAGAATTAGTAGAGCTTTGCCCATACTCATTATTCCTCTTGGAGATCAAGACCTCC
                ***********  *********** **** ****************** ******* ***

TaDrAp1-B4      ATGTGAGAAGATCCCCCTAGTGGATGCAAGAGCCCTATAAGGGAATATCAATCTAGGGAT
TaDrAp1-D4      ATGTGAGAAGATCCCC-TAGTGGATGCAAGAGCCCTATAAGGGAAGATCCATCTAGGGAT
TaDrAp1-A4      ATGCGAGAACATCCCC-TAGTGGATGCAAGACCCATATAAGGGAAGATCCATCTAGGGAT
                *** ***** ****** ************** ** ********** *** **********

TaDrAp1-B4      GCAAGACCCCTATATGGGAAGATCCTCCATGGATGCTAGATCTCATCTCCTTTGGATTTG
TaDrAp1-D4      GCAAGACCCCTATATGGGAAGATCCTCCATGGATGCTAGATCTCATCTCCTTTGGATTTG
TaDrAp1-A4      GCAAGACCCCTATATGGGAAGATCCTCCATGGATGCTAGATCTCATCTCCTTTGGATTTG
                ************************************************************

TaDrAp1-B4      AGAAGAACCTTTACCTTTGTGCTTCCCCCTTTTGACTTGTTAATATATATTTGTGGATCT
TaDrAp1-D4      AGAAGAACCTTTACCCTTGTGCTTCCCCCTTTTGACTTGTTCTTGCATATTTGTGGATCT
TaDrAp1-A4      AGAAGAACCTTTACCTTTGTGCTTTCCCCTTT-GACTTGTTCTTGTATATTTGTGGATCC
                *************** ******** ******* ********  *  *************

TaDrAp1-B4      CATGTATGCTATCTTAGTGATGTGTGATTTGGATTTGTTTGAGTCTCTCTCTCTCTCTCT
TaDrAp1-D4      CATGTATGCTATCTTAGTGATGTGTGATTTGGATTTGTCTGAGTCTTTCTCTCTCT----
TaDrAp1-A4      CAT------------AGTGATGTGTGATTTGGGTTTGTTTGAGTCTCTCTCTCTCTCTCT
                ***            ***************** ***** ******* *********

TaDrAp1-B4      TGTGTTATTGAGTGTTCATCCTTTCTCCATCCTCCAAGTGCGAAAAGATCTTGAATCTAG
TaDrAp1-D4      TGTGCTCTTGAGTGTTCATCCTTTCTCCA-CCTCCAAGTGCGAAAAGATCTCAAATCTAG
TaDrAp1-A4      CGTGTTCTTGAATGCTCATCCTTTCCCCA-CCTCCAAGTGCGAAAAGATCTCAAGTCTAG
                 *** * **** ** ********** *** *********************  * *****

TaDrAp1-B4      GGTTTTGCCCTACATCACCATGCCTCCCTAGCCCC-CCTCTCCTCTCCTCCTTCCCTTCC
TaDrAp1-D4      GGTTTTGCCCTACATCACCGTGCCTCCCTAGCCCCTCCTCTCCTCTCCCCCTTCCTTCCC
TaDrAp1-A4      GGTTTTGCCCTACATCACCGTGCCTCCCCATCCCTTCCTCTCCTCTCCTCTTTCCTTTCT
                ******************* ******** * ***  ************ * **** * *

TaDrAp1-B4      CGCAGGTCACTGACCTCCTACTTGTGTCGGGTCCCTCTCTTCCTTCCTTCCCCGTAGGTC
TaDrAp1-D4      CGCAGGTCACTGACCTCCTACTCATGTCGGGTCCCTCTCCTCCTTCCTTCCCCGTAGGTC
TaDrAp1-A4      CGTAGGTCACTGACCTCCTACTCGTGTCAGGTCCCTCTCCTCCTTCCTTCCTCGCAGGTC
                ** *******************  **** ********** *********** ** *****
```

```
TaDrAp1-B4      GTTGAGCAAGGAATCGAACTGGGCAAACGTGACGGCCGGCCGGATCGATGCTGAATTGAC
TaDrAp1-D4      GTTGAGCAAGAAATCGAGCTGGGCAAACGTGACGGCTGGCCGGATCAATGCCGAGTTGTC
TaDrAp1-A4      GTCGAGCAAGGAATCGAGCTGGGCAAACGCGACGGCCGGTCGGATCAATGCCGAATTGAC
                ** ******* ****** *********** ****** ** ****** **** ** *** *

TaDrAp1-B4      CGGATCCGACGTAGGGATCACAACAAGGAGGAGGAAGTCGGAGGAAGGAACGGTGGCGGG
TaDrAp1-D4      CGGATCCGACGCAGGGATCACAACAAGGAGGAGGAAGTCGGAGGAAAGAGAGGTGGCGGG
TaDrAp1-A4      CGGATCCGACGCAGGGATCACAACAAGGAGGAGAAAGTCGGAGGAAGGAGCGGTGGCGGG
                *********** ********************* ************ **  *********

TaDrAp1-B4      CAGGGGCGGCGGCTACAATGTCGTCGGTGGAAGGAGCGACAATGGAGGCTCCGCCGACCA
TaDrAp1-D4      CAGAGGCGGCGGCGACGGTGTCGTCGGTGGAAGGAGCGGCAATGGAAGCTCCGCCGACCA
TaDrAp1-A4      CAGGGGCGGCAACCATGGTGGCGTTGGTGGCAGGAGTGGCAATGGAGGCTCCGCCGACCA
                *** ******  * *   ** *** ***** ***** * ******* *************

TaDrAp1-B4      TGGCGAGGAAAGCGAGATCGGCGATGAGCTTCGCGGGGTCCGCTGCGGGG-TCAGAGGAG
TaDrAp1-D4      TGGCGAGGAAAGCGAGATCGGCGGTGAGCTTCGCGGGGTCGGTTGCAGGG-CCAAAGGAG
TaDrAp1-A4      CGACGAGGAAAGCGGGATCTGCAGTGAGCTTCGCGGGGTTGGCTGCGGGGGTCAGAGGAG
                 * *********** **** **  ***************  * *** ***  ** *****

TaDrAp1-B4      CTGGCCATGGCGACGCGGTGGCCGGCGGGAAAGCGGAGGCAACATTCCGGTTGCGCGCGG
TaDrAp1-D4      CTGGCCATGGCGACGTGATGGCCGGCGGGAGAGCGGAGGCAGCGTTCCGGTTGCGCGCGG
TaDrAp1-A4      CTGGCCATGGCGACGCTGTGGCCAGCGGGCGATCAGAGGCGGCGTTCCGGTTGCGCGCGG
                ***************   ***** *****  * * *****  * ****************

TaDrAp1-B4      GAAGAAAAGGGTAGCGATTATTTTTCGTGATGCAAGCACTCTTTT---GTGGCAGTCGCG
TaDrAp1-D4      GTAGAAAAAGGTAGCTATTGGGTTTCATGATGCAACCACTCTTTT---GTGACAGCTGCC
TaDrAp1-A4      G---AAAAAGGTAGCGATTGGGTTTCGCGATGCAACCGCTCTTTTTTTGTGGCAGCCGCC
                *   **** ****** ***   ****  ******* * *******   *** ***  ** 

TaDrAp1-B4      TGACTACCCTATATTCGCTACACTCGAAGCATTTTTTTTAGGTTGCCGGA----TTTTTT
TaDrAp1-D4      CGACTACTCTATATTCGCTGCACT--GAGGATTTTT------------------------
TaDrAp1-A4      CTACTACTCTATATTCGCTGCACTTGAGGACTTTTTGTTGGCTTCCCCAAATATTTTTTT
                  ***** *********** ****    *  *****

TaDrAp1-B4      CAAGAAAATTTAGAATTT-----------------------GATAGCTTTTCGTGCT-TA
TaDrAp1-D4      -AAAAAAAATTAGAATTC-----------------------GGTAGCCTTTCGTGCT-TA
TaDrAp1-A4      TCAAAAATATTAGAATTTTTTAGAGGAAAAAAAAAGAATAAAATAGCCTTTCATGCTCTA
                  * ***  ********                         **** **** **** **

TaDrAp1-B4      AAAACAATTCGTGTGTGAAAATAATAGGGCTA-CTGTGAGAGATGCGGTAAATATCTTTT
TaDrAp1-D4      AAAACAATTCGTGTGTGAAAATAATAGGGCT------GGGAGATGCGGTAATTATCTTTT
TaDrAp1-A4      AAAACAACTTGTGCCTGAAAATTATAGGGCTTGCAGTGGGAGACGCGGCAAATATCTTTT
                ******* * ***  ******* ********      * **** **** ** ********

TaDrAp1-B4      -GGCACGAGTC-----------CGCCGTGTTGGTGCAAGTTGGTGGAAAGACAACAAAAC
TaDrAp1-D4      -GGCACGAGTC-----------CGC-GTGCCG-TGTAAGTTGGTGGAAAGACAACAAAAC
TaDrAp1-A4      TGGCACGAGTCGGTGTGCCCCGTGCCGTGGTGGTGCAAGTTGGTGGAAAGACAACAAAAC
                 **********            ** ***  * ** ************************

TaDrAp1-B4      AAGAAACCCAATCGTCAA-GAAGAACGAGAAAAGAAAATACACAAAAGGTAGACTACGAT
TaDrAp1-D4      AAGAAATCCAATCGTCAA-GAAGAACGAGAAAAGAAAATACACAAAAGATAGACTACGAT
TaDrAp1-A4      AAGAAATCCAATCGTCAAAGAAGAACGAGAAAAGAAAATACACAAAAGATAGACCACGAT
                ****** *********** ***************************** ***** *****

TaDrAp1-B4      TGCCGGCATCGAGCTCGACGAGGCGTCGGAG-TTGCGACAAAGATGAGGAAGAAGCTGGG
TaDrAp1-D4      TGCCGGCATCGAGCTCGACGAGGCGTCGGAGGTTGCGTCAAAGATGAGGAAGAAGCTGGG
TaDrAp1-A4      TGCCGGCATCGAGCCCGACGAGGCGTCGGAG-GTGCGACAAAGATGAGGAAGAAGCTGGG
                ************** ****************  **** **********************

TaDrAp1-B4      CACCCGGTTCCCCGCG
TaDrAp1-D4      CACCCGGTTCCCCGCG
TaDrAp1-A4      CACCCGGTTCCCCGCG
                ****************
```

## Primers used for sequencing *TaDrAp1*:

**TaDrAp1-A4-seq-F: GACTGGGCGTTCGGTACTGCA** 21 bp, 62%GC, Tm=58.3C
**TaDrAp1-A4-seq-R: CACGGCACGGGGCACACC** 18 bp, 78%GC, Tm=59.4C
TaDrAp1-A4-seq-R (RevCom): GGTGTGCCCCGTGCCGTG
Amplicon size: 1,148 bp

**TaDrAp1-B4-seq-F: CAATCACTCGCGAACGACCAGA** 22 bp, 55%GC, Tm=56.7C
**TaDrAp1-B4-seq-R: TGTCTTTCCACCAACTTGCACCAA** 24 bp, 46%GC, Tm=55.7C
TaDrAp1-B4-seq-R (RevCom): TTGGTGCAAGTTGGTGGAAAGACA
Amplicon size: 1,162 bp

**TaDrAp1-D4-seq-F: GCCCATGTACCCCCTTTTCATC** 22 bp, 55%GC, Tm=56.7C
**TaDrAp1-D4-seq-R: TTGTCTTTCCACCAACTTACACGG** 24 bp, 46%GC, Tm=55.7C
TaDrAp1-D4-seq-R (RevCom): CCGTGTAAGTTGGTGGAAAGACAA
Amplicon size: 1,068 bp

## *TaDrAp2* - Promotor regions – 2Kb

*>TaDrAp2-A1*

AGGAGGTTCGCCGGTGTCGCGAGGCCTTCACTCCAGTCTCCGGAGCAGCGGCGGATGCGCGCCTAC
GCGCCAGAATCTCCCAACTGGTCGCGCTGGTTCGCGCTAGAGCACGAGGAGAAGCGCCGCCAGGGC
GTCCACGTCGACCACGACCTTCCTCCTCCGCCACCTGAGGTTCTGCTGCACGAGCAGGACGAAGAG
GCGGCATACCAAGCCGAGCAAGCCCTGCAGCACGCGTTGGAGGCGAGCAGGATCGAGGAGGAGGCC
TAATGGGATGGGATGGAGCGCGCCCTCGCATTGTCGGTGGCAGGAGAGTTCGTCCACGCCCCGCTC
TTCGACTCGCCTCCACCGCCACCGGCGCTACCCATCGTCGAGCCCAAGCCAGAGCCGGAGCCGACG
CGAAAGTGCTCGCCGCCTCCACCCACTTGGCCGGAGGAGTCCTACATGTGGACCGACGAGTACCGT
GAGTGGATGAGCACGCCTCCGGTCCACTACGCCGCGAGGCCGGAGGAGGAGGCGGCCCACCTTGAG
CTCTGGAAGGCGCATTGGCTCGCCTAGGAGGAGGCCGACGGCGAGCGGCAGATGCTTTGGGAGCAG
CAGCTATGCCGCGATGCGGAGGCGTTGCGGCTTGAGGAGGAGAAAGAAGAGTGCGCCCAGCTAGCC
ACAATGTCGGCGCCCCAGCAGACGCCGGAGGAGGCTGCCTTGGCAGTGTACCAAGCGGCGTTTGGG
TGGGCTGGCCCTGCTCCCGTCTTTATCGACCTCACCGGCGGCGATAGCGATGTCAAGGGCAAGGGC
AAGGCGGAGGACGTCTAGGGCAGCGTGCGGGCGCATATCTTTTTATTAATGTTTTTAATTAGGTTT
AAGTGGACTTTGGCCGGCGGTTGACCAACCACTTTAATGTTTAATTATGTTTATTTCTATGACCTG
TTTTTTTCTACACCGGCAAATTTGGGCCCGTCTTTCATTGGGCGCACCTGCCGACCAATATGAAAA
TGCCGACACGCACGTCTGTCTGGCCGATCCAAACAGATGAAAAGCGAATAAAACGCACGTCCGATT
GGGTCGGCGCGTTGGAGGTGCTCTTACACCCTGAAACATGATTCCCTTTACGTTTACGTTAACACT
CCACAAAACAAACATGGCAATGTATTCTTAGCTCACAAAGACAAGTCAAAGCATCTCCCTCCGTTT
CCTTTTTTACTCTACATATACACTTTGATCAAAGTCAAACTAAACAATGTTTCAACAATTTTATAT
AAAAAAATATTAATATCTACAAATATCAAAGTTATATGGTATATGAAAGTTAATTTTATGATGCAT
CTAATAATATTGATTTCATATTGTGAATATTGCTATTGTTACCAATAAAATTAGTCAAACATTATA
AAGTTCGACTTTGACTTTTTTTTGAGCAAATGAACAATTTTTTTAAGACAAACCAACTTTATATGC
AAACTAAAAAGAAAACGGAGGAAGTACCCAGATAAAGCTAGAGCCCAAGAATGGACGATGACTCTC
GCAGCCCAAGGCCACGAACGAGACACAGCCGCCGGATCCCGCCGACCCCTCGAATCGGACGGCGGA
CACAGGGAGCGCCACGAACCAGCCAGAAACATGGGGCCCACCTCATCCGGGGAACGCGACACGGAC
GGCTGACCTGCGGCCGCACGCGCAAACGGTTCGGGAGAGGTCTAACTACGCGGGCCCCCACATCGC
GCTGAGCTTTTACCCACCGCGAAACGTCTCCGTGGACCCGGTCCGTCGCATCCACAGCATGGACCA
TCTACTACGGTCTTAGCGCCGCACGTGAGGCCAAGGCCTATGCGCCGGGTCCAGGAGCTTAGCGTC
CATGCGCCGGAGAGTCCGCGCACCTCTGCCCGCTTCGCGCGGCCATTGGCCGCGCTGTTTATGGGG
GAAAAAATAGTTTGTTTTTATTTTCTCCCGTAAAAAAAGTGAAAAGATAGCGATAACGGAAACCCT
AATCCCCAAATCAGAAATCTATCCGAATCGCTATTTATGTTTGTGGCTTTCTCCCCCCTGCGATCC
GATGTCCCCCGCTTCGCCCGCCGCGCCGATCCGACCTCCCGCCGCCGCGTTCTCTCCGCCGGGGAC
GCTCTCTCCGGCGCCGCCGGCTCCGCGCCATGAGGAAGAAGCTCGACACCCGCTTCCCCGCG

*>TaDrAp2-B1*

TGAGACGCGGTGAGGCTACAATGATCTCCCACGAGACTGCGGGACTGTAGCCACACCCCTCCCCCG
ATCAAGCATGCGTCATTAACATTGCGGCTACAATAACCAGCCAACCAGACCCGCCAGCGGCGGAGG
CGGCCTGTCGGCTTCGTACCAGACCAGTCGACGGGGCTCACCAGGCTGCGGGCCCCAGCAGTCGGC
AGAGAAGCCGGCGACCGGAGGCACTGCCAACTGGGCCCTACACCCGGTCAGATTACCAATGTACCC
CTGAGGGGTAGGCCTATATAAACCCCACCAGGGCACCCATGCAAAGGGTGCCAGGCTGGTTAGAAC
TAGACTCATACATAGAGGGAGAAGAGAGCAAGCCCTGCCTCCTTCTACCTTCAGCATACAGCTCGA
GGAGCACCATTGTATTCACTAGTGTCTTAGTGATCATGCGGAGACCCCGCAGAGCAGGACTAAGGG
TGTTATCTCCTAGGAGAGCCCCAAACCTGGGTAAAGTGCGCCGACTTTCGTGTCTACGCCTCATCC
CGCTTCCAGGCACCGGCGACGTTCTACTCGCTCCCACCATGATAAGCCATCCTTTGGCATATGTCG
CACCCAACCCCCAACATGTCGCATGGATCTGACGCTGGGTGTGTTGTCACTGCCTTTGGGGTGCCA
CCCGTCTGGCATCTGAAGCATGGCGCGGCGTGCCTGTTGTACGGGGCAACAAGCGCGAGAGGCGGT
GGCGACCCTCATGGCGGTGGATGTCGGCGCAGTGGAGTTGCATTCTCCGATGCCCCGTAAGGCACG

ACAGTCCGGGCGCCACAACTGTGTCGTGGTGGATGTCGCTGGCTCGTCCTGGGATGGATCCATCGT
TGATATGACGTCCGAGTTCCGACGAGGAAGAGTAGGACCTAGGAGACGGCGGCGCCTTGAGTCCCG
TGAGTCGACTCGTGTTCCATGCCCTACTCTACCTTTCAGGCGACCGAACCAACACTCTGAGAAGCG
CAGCCGACCGCCGGACGTAGGCACGACGGAGGCGGACGAGCTCCGCTTAAAGATCGTTTTACGTTG
CATGTAATAATATGAATTTGAGGTTTCTACTTTTAGGTATCCGGTTATAAAATCAAACTTTTAAGA
CGTGATAGGTCATTGTCTGCGGACACGCCCAGACTTGTCTGCGGACATTTGAGGGGGCGGGTTTGC
GAAGTACGACTGTATATCCTCTTACACCCTGAAACATGATCCCTTTACGTTTAGTTAACACTCCTC
AAAACAAAACACGACAAGATATGCCGACTCAGTCTTTCAGATGCGCTTATAGGAATAGAAAATGCG
TGTGTGCGTTTATAGGGATAAGCGTATGCGCGTGTATATGAGCGCTTGCGTCTGTACCGTGTTTAA
AAAAACACACGGCAATCTATTCTGATCTCACAAAGACAAGTCGGAACATGCTATACCCAGATAAAG
CGAGAGCCCGAGAATGGACGATGACTCTCGCAGCCCAAGGCCACGAACGAGGCACAGCGCCACAGC
CACCGGATCCCGCCGACCCCCCGAATCGGACGGCGGACACAGGGAGCGCCACGAACCGGCCAGAAA
CGTGGGGCCCACCTCATCCGGGGAACGCGACGCGGACGGCTGACGTGCGGCCGCACGCGCAAACGA
GGGAGAGGTCTAACTACGCGGGCCCCTACATCGCGCCCAGCTTTTACCCACCGCGAAACGTCTCCA
TAGACCCGGTCCGTCGCATCCCCACATGAACCTTCTACGGTCTTAGCGCCGCACGTGAGGCTAAGA
CCTATGCACCGGGTCCAGGAGCTTAGCCTCCACGCGCCGGAGAGCTCGCGCACCTCTGCCCGCTTC
GCGCGGCCATTGGCCGCGCTGTTTATGGGGGAAAAATATATTTTTTCCCGTAAAAAAAGTGAAAAG
ATAGCGATAACGGAAACCCTAATCCCCAAATCAGAAATCTATCCGAATCGCTATTTATGTTTGTGG
CTTTCTCCCCCCTCCGATCCGATGTCCCCCGCTTCGTCCGCCGCGCCGATCCGACCTCCAGCCGCC
GCATTCTCTCCGCCGGGGACGCTCCCTCCGGCGCCGCCGGCTCCGCGCCATGAGGAAGAAGCTCGA
CACCCGCTTCCCCGCG

>*TaDrAp2-D1*
TGCTTGGCTTGGGGGGCAAGTCTCCCTAGGATTTTCCCTAGGGAGATCCAATCTGCTTGGCCGCCG
CCCCCTAGGGGAAACCCTAGGGCGCCTCCCCCTCTCCCCTTGCCCATATATATAGTGGAGGGGTAG
GGGGCAGCCGCACCTCTTCCCTGGCGCAGCCCTCTCCTCCTCCAACTCCTCCTCCTCCTCCGTAGT
GCTTAGCAAAGCTCTGCCGGAGAACCACGAGCTCCATTGCCACCATGTCGTCGTGCTGCTGGAGTT
CTCCCTCAACTTCTCCTCTCCCCTTGCTGGATCAAGAAGGAGGAGACGTCCCCGGGCTGTAGGTGT
GTTGAACGCGGAGGCGCCGTCCGTTCGGCGCTAGATCGGATCTTCCGCGATTTGAATCGCCGCGAG
TACGACTCCATCAACCGCGTTCTTGTAACGCTTCCGCTTAGCGATCTTCAAGGGTATGAAGATGCA
CTCCCTCTCTCTCGTTGCTAGCATCTCCTAGATTGATCTTGGTGACACGTAGGAAAATTTTGAATA
ATTACTACGTTCCCCAACAGAGGCCCAATGGGATGGGATGGAGCGCGCCCTGGCCTTGTCGGCGGC
AGGGGACTCCGTCCATGCCCTGCTCTTCGACTCGCCTCCACCGCCGCCCATCGTCGAGCCCAAGCC
AGAGTCGGAGCCGGCGCGGAAGCGCTCGCCGCCTCCACCCACTTGGCCGGAGGAGTCCTACACGTG
GACCGGCGAGTACCGCGAGTGGGTGAGCGCGCCTCCGGTCCACTACGCCGCGACGCCGGAGGAGGA
GGCAGCCCACCTTGAGCGTTGGGAGGCGCACTGGCTCACTCAGGAGGAGGCCGACGGCGAGCAGCA
GATGCTTTGGGAGCAGCAGCTGCGGCTTGAGGAGGAGGAGGAGCGCGCCCGGCTAGCCGCAATGCC
GGCGCCCCAGCAGACGCCGGAGGAGGCTGCCCTGGCAGAGTACCAAGCGGCATTCGGGTGGGCTAG
CCCTGCTCCCGCCTTCATCGACCTCACCGGTGGCGATGGCGTCGTCAAGGGCAAGGGCAAGGCGGA
GGACGTGTAGGGCAGCGTGAGGGCGTAGACTTTTTTAATTAATGTTTAATTAGGTTTAAGTGGAC
TTTGGTCGGCGGTTGACCAGCCACTTTAATGTTTAATTATGTTTATTTCTACGACCTGTTTTTCTT
CTACGCCGACAAATTTGGGTCCGTCTTTCGTTGGGCGCACCTGCCGACCCATATAAAAATGCGGAC
GTGCACGTCCGCCTGACCGATCTAAACGGACGAAAAGCGGACAAAACGCGCGTCCGTTTGGTCGGC
GCCTTGGAGGTGCTCTTACACCCTGAAACATGATTCCCTTTACATTTACGTTAACACTCCTCAAAA
CAAACACGGCAATCTATTCTGATCTCACAAAGACAAGTCGGAGCATACTCTCTACCCAGATAAAGC
AACAGCCCAAGAATGGACGATGACTCTCGCAGCAAGGCCACGAACGAGGCACGGCGCCACAGCCGC
CGGATCCCGAATCGGACGGCGGACACAGGGAGCGCCACGAACCGGCCAGAAACGTGGGGCCCACCT
CATCCGGGGAACGCGACGCGGACGGCTGACGTGCGGCCGCACGCGCAAACGAGGGAGAGGTCTAAC
TACGCGGGCCCCCACATCGCGCTGAGCTTTTACCCACCGCGAAACGTCTCCGTGGACCCGGTCCGT
CGCATCCGCCACATGGACTCTACGGTCTTAGCGCCGCACGTGAGGCTAAGGCCTATGCGCTGGGTC

CAGGAGCTTAGCGTCCACGCGCCGGAGAGTTCGCGCACCGCTGCCCGCTTCGCGCGGCCATTGGCC
GCGCTGTTTATGGGAAAAAAAATAGTTTGTTTTTATTTTTCCCCGTAAAAAAAGTGAAAAGATAGC
GATAACGGAAACCCTAATCCCCAAATCAGAAATCTATCCGAATCGCTATTTATGTTTGTGGCTTTC
TCCCCCCTCCGATCCGATGTCCCCCGCTTCGTCCGCCGCGCCGATCCGACCTCCAGCCGCCGCGTT
CTCTCCGCCGGGGACGCTCTCTCCGACGCCGCCGGCTCCGCGCCATGAGGAAGAAGCTCGACACCC
GCTTCCCCGCG

## *TaDrAp2* – Promotor region

### CLUSTAL 2.1 multiple sequence alignment

```
TaDrAp2-A1      GACCTTCCTCCTCCGCCACCTGAGGTTCTGCT-GCACGAGCAGGACGAAGAGGCGGCATA
TaDrAp2-D1      GTTCTTGTAACGCTTCCGCTTAGCGATCTTCA-AGGGTATGAAGATGCACTCCCTCTCTC
TaDrAp2-B1      GCAAAGGGTGCCAGGCTGGTTAGAACTAGACTCATACATAGAGGGAGAAGAGAGCAAGCC
                *         *    *    *     *   *          * *  * *

TaDrAp2-A1      CCAAGCCGAGCAAGCCCTGCAGCACGCGTTGGAGGCGAGCAGGA-----TCGAGGAG---
TaDrAp2-D1      TCGTTGCTAGCATCTCCTAGATTG-ATCTTGGTGACACGTAGGAAAATTTTGAATAATTA
TaDrAp2-B1      CTGCCTCCTTCTACCTTCAGCATACAGCTCGAGGA---GCACCATTGTATTCACTAGTGT
                      *   *                 * *  *    * *  *     *  *  *

TaDrAp2-A1      ---------------GAGGCCTAATGGGATGGGATGGAGCGCGCCCTCGCATTGTCGGTG
TaDrAp2-D1      CTACGTTCCCCAACAGAGGCCCAATGGGATGGGATGGAGCGCGCCCTGGCCTTGTCGGCG
TaDrAp2-B1      CTTAGTGATCATGCGGAGACCCCGCAGAGCAGGACTAAGGGTGTTATCTCCTAG--GAGA
                               *** **     *    ***   ** * *   *  * * *  *

TaDrAp2-A1      GCAGGAGAGTTCGTCCA--CGCCCCGCTCTTCGACTCGCCTCCACCGCCACCGGCGCTAC
TaDrAp2-D1      GCAGGGGACTCCGTCCA--TGCCCTGCTCTTCGACTCGCCTCCACCGCCGCC--------
TaDrAp2-B1      GCCCCAAACCTGGGTAAAGTGCGCCGACTTTCG---TGTCTACGCCTCATCC--------
                **     *    *   *   ** * *   ****    * ** * ** *  **

TaDrAp2-A1      CCATCGTCGAGCCCAAGCCAGAGCCGGA--GCCGACGCGAAAGTGCTCGCCGCCTCCACC
TaDrAp2-D1      -CATCGTCGAGCCCAAGCCAGAGTCGGA--GCCGGCGCGGAAGCGCTCGCCGCCTCCACC
TaDrAp2-B1      -CGCTTCCAGGCACCGGCGACGTTCTACTCGCTCCCACCATGATAAGCCATCCTTTGGCA
                 *     *  ** *  ** *    *     **   * *         *    * *   *

TaDrAp2-A1      CACTTGGCCGGAGGAGTCCTACATGTGGACCGACGAGTACCGTGAGTGGATGAGCAC-GC
TaDrAp2-D1      CACTTGGCCGGAGGAGTCCTACACGTGGACCGGCGAGTACCGCGAGTGGGTGAGCGC-GC
TaDrAp2-B1      TATGTCGCACCCAACCCCCAACATGTCGCATGGATCTGACGCTGGGTGTGTTGTCACTGC
                 *  * **          ** *** ** *   *      **   * ***  *   * * **

TaDrAp2-A1      CTCCGGTCCACTACGCCGCGAGGCCGGAGGAGGAGGCGGCCCACCTTGAGCTCTGGAAGG
TaDrAp2-D1      CTCCGGTCCACTACGCCGCGACGCCGGAGGAGGAGGCAGCCCACCTTGAGCGTTGGGAGG
TaDrAp2-B1      CTTTGGGGTGCCAC-CCGTCTGGCATCTGAAGCA--TGGCGCGGCGTGCCTGTTGTACGG
                **  **    * ** ***    **    * ** *    ** *  * **     **   **

TaDrAp2-A1      CGCATTGGCTCGCCTAGGAGGAGGCCGACGGCG-AGCGGCAGATGCTTTGGGAGCAGCAG
TaDrAp2-D1      CGCACTGGCTCACTCAGGAGGAGGCCGACGGCG-AGCAGCAGATGCTTTGGGAGCAGCAG
TaDrAp2-B1      GGCAACAAGCGCGAGAGGCGGTGGCGACCCTCATGGCGGTGGATGTCGGCGCAGTGG-AG
                 ***           *** ** ***   *  *   ** *  ****     * **  * **

TaDrAp2-A1      CTATGCCGCGATGCGGAGGCGTTGCGGCTTGAGGAGGAGAAAGAAGAGTGCGCCCAGCTA
TaDrAp2-D1      CT---------------------GCGGCTTGAGGAGGAG---GAGGAGCGCGCCCGGCTA
TaDrAp2-B1      TT---------------------GCATTCTCCGATGCCC--CGTAAGGCACGACAGTCCG
                 *                      **    *  *  *      *    *  ** *   *

TaDrAp2-A1      GCCACAATGTCGGCGCCCCAGCAGACGCCGGAGGAGGCTGCCTTGGCAGTGTACCAAGCG
TaDrAp2-D1      GCCGCAATGCCGGCGCCCCAGCAGACGCCGGAGGAGGCTGCCCTGGCAGAGTACCAAGCG
TaDrAp2-B1      GGCGCCACAACTGTGTCGTGGTGGATGTCGCTGG--CTCGTCCTGGGATGGATCCAT---
                * * * *   * * * *   *  ** * **  **     * * *** *  *  ***

TaDrAp2-A1      GCGTTTGGGTGGGCTGGCCCTGCTCCCGTCTTTATCGACCTCACCGGCGGCGATAGCGAT
TaDrAp2-D1      GCATTCGGGTGGGCTAGCCCTGCTCCCGCCTTCATCGACCTCACCGGTGGCGATGGCGTC
TaDrAp2-B1      -CGTTGATATGAC---GTCCGAGTTCCGACGAGGAAGAGTAGGACCTAGGAGACGGCGGC
                 * **    **     * **   * *** *      **      *   ** **  ***
```

```
TaDrAp2-A1      GTCAAGGGCAAGGGCAAGGCGGAGGACGTCTAGGGCAGCGTGCGGGCGCATATCTTTTT-
TaDrAp2-D1      GTCAAGGGCAAGGGCAAGGCGGAGGACGTGTAGGGCAGCGTGAGGGCGTAGACTTTTTTT
TaDrAp2-B1      GCCTTGAGTCCCG-TGAGTCGACTCGTGTTCCATGCCCTACTCTACCTTTCAGGCGACCG
                * *  * *    *   ** **      **     **          *    *

TaDrAp2-A1      -ATTAATGTTTTTAATTAGGTTTAAGTGGACTTTGGCCGGCGGT-TGACCAACCACTTTA
TaDrAp2-D1      AATTAATGTTT--AATTAGGTTTAAGTGGACTTTGGTCGGCGGT-TGACCAGCCACTTTA
TaDrAp2-B1      AACCAACACTCTGAG--AAGCGCAGCCGACCGCCGGACGTAGGCACGACGGAGGCGGACG
                 *  **   *   *   * *   *   *  *   ** **  **   ***

TaDrAp2-A1      ATGTTTAATTATGTTTATTTCTATGACCTGTTTTT-TTCTACACCGGCAAATTTGGGCCC
TaDrAp2-D1      ATGTTTAATTATGTTTATTTCTACGACCTGTTTTTCTTCTACGCCGACAAATTTGGGTCC
TaDrAp2-B1      AGCTCCGCTTAAAGATCGTTTTACG------TTGCATGTAATAATATGAATTTGAGGTTT
                *  *    ***    *  ** ** *      **   *   *       ** **  **

TaDrAp2-A1      GTCTTTCATTGGGCGCACCTGCCGACCAATATGAAAATGCCGACACGCACGTCTGTCTGG
TaDrAp2-D1      GTCTTTCGTTGGGCGCACCTGCCGACCCATATAAAAATGCGGACGTGCACGTCCGCCTGA
TaDrAp2-B1      CTACTTTTAGGTATCCGGTTATAAAATCAAACTTTTAAGACGTGATAGGTCATTGTCTG-
                 *  **    *    *   *    *   * *     * *  *            * ***

TaDrAp2-A1      CCGATCCAAACAGA-TGAAAAGCGAATAAAACGCACGTCCGATTGGGTCGGCGCG-TTGG
TaDrAp2-D1      CCGATCTAAACGGA-CGAAAAGCGGACAAAACGCGCGTCCGTTTGG-TCGGCGCC-TTGG
TaDrAp2-B1      CGGACACGCCCAGACTTGTCTGCGGACATTTGAGGGGGCGGGTTTGCGAAGTACGACTGT
                * **      * **       *** * *        * * * ** *    *  *   **

TaDrAp2-A1      AGGTGCTCTTACACCCTGAAACATGATTCCCTTTACGTTTACGTTAACACTCCACAAAAC
TaDrAp2-D1      AGGTGCTCTTACACCCTGAAACATGATTCCCTTTACATTTACGTTAACACTCCTCAAAAC
TaDrAp2-B1      ATATCCTCTTACACCCTGAAACATGAT-CCCTTTACGTTTA-GTTAACACTCCTCAAAAC
                *  * ********************** ******** **** *********** ******

TaDrAp2-A1      AAA-CATGGCAATGTATTCTTAGCTCACAAAGACAAGTCAAAGCATCTCCCTCCGTTTCC
TaDrAp2-D1      AAA-CACGGCAATCTATTCTGATCTCACAAAGACAAGTCGGAGCAT--------------
TaDrAp2-B1      AAAACACGACAAGATATGCCGA-CTCAGTCTTTCAGATGCGCTTATA------------G
                *** ** * ***  *** *  * ****      **  *      **

TaDrAp2-A1      TTTTTTACTCTACATATACACTTTGATCAAAGTCAAACTAAACAATGTTTCAACAATTTT
TaDrAp2-D1      ------ACTCTC------------------------------------------------
TaDrAp2-B1      GAATAGAAAATGCGTGTGTGCGTTTATAGGGATAAGCGTATGCG----------------
                      *   *

TaDrAp2-A1      ATATAAAAAAATATTAATATCTACAAATATCAAAGTTATATGGTATATGAAAGTTAATTT
TaDrAp2-D1      ------------------------------------------------------------
TaDrAp2-B1      ------------------------------------------------------------

TaDrAp2-A1      TATGATGCATCTAATAATATTGATTTCATATTGTGAATATTGCTATTGTTACCAATAAAA
TaDrAp2-D1      ------------------------------------------------------------
TaDrAp2-B1      ------------------------------------------------------------

TaDrAp2-A1      TTAGTCAAACATTATAAAGTTCGACTTTGACTTTTTTTTGAGCAAATGAACAATTTTTTT
TaDrAp2-D1      ------------------------------------------------------------
TaDrAp2-B1      -----CGTGTATATGAGCGCTTGCGTCTGTACCGTGTTTAAAAAAACACACGGC------

TaDrAp2-A1      AAGACAAACCAACTTTATATGCAAACTAAAAAGAAAACGGAGGAAGTACCCAGATAAAGC
TaDrAp2-D1      ----------------------------------------------TACCCAGATAAAGC
TaDrAp2-B1      ------AATCTATTCTGATCTCACAAAGACAAGTCGGAACATGCTATACCCAGATAAAGC
                                                              **************
```

```
TaDrAp2-A1      TAGAGCCCAAGAATGGACGATGACTCTCGCAGCCCAAGGCCACGAACGAGACACAGCC--
TaDrAp2-D1      AACAGCCCAAGAATGGACGATGACTCTCGCAGC--AAGGCCACGAACGAGGCACGGC---
TaDrAp2-B1      GAGAGCCCGAGAATGGACGATGACTCTCGCAGCCCAAGGCCACGAACGAGGCACAGCGCC
                 * ***** ************************  *************** *** **

TaDrAp2-A1      ------GCCGGATCCCGCCGACCCCTCGAATCGGACGGCGGACACAGGGAGCGCCACGAA
TaDrAp2-D1      ------GCCACAGCCGCCGGATCC--CGAATCGGACGGCGGACACAGGGAGCGCCACGAA
TaDrAp2-B1      ACAGCCACCGGATCCCGCCGACCCCCCGAATCGGACGGCGGACACAGGGAGCGCCACGAA
                       **  * **  * ** **  **********************************

TaDrAp2-A1      CCAGCCAGAAACATGGGGCCCACCTCATCCGGGGAACGCGACACGGACGGCTGACCTGCG
TaDrAp2-D1      CCGGCCAGAAACGTGGGGCCCACCTCATCCGGGGAACGCGACGCGGACGGCTGACGTGCG
TaDrAp2-B1      CCGGCCAGAAACGTGGGGCCCACCTCATCCGGGGAACGCGACGCGGACGGCTGACGTGCG
                ** ********* ***************************** ************ ****

TaDrAp2-A1      GCCGCACGCGCAAACGGTTCGGGAGAGGTCTAACTACGCGGGCCCCCACATCGCGCTGAG
TaDrAp2-D1      GCCGCACGCGCAAACGA---GGGAGAGGTCTAACTACGCGGGCCCCCACATCGCGCTGAG
TaDrAp2-B1      GCCGCACGCGCAAACGA---GGGAGAGGTCTAACTACGCGGGCCCCTACATCGCGCCCAG
                ****************    ************************** *********  **

TaDrAp2-A1      CTTTTACCCACCGCGAAACGTCTCCGTGGACCCGGTCCGTCGCATCCACAGCATGGACCA
TaDrAp2-D1      CTTTTACCCACCGCGAAACGTCTCCGTGGACCCGGTCCGTCGCATCCGCCACATGGACT-
TaDrAp2-B1      CTTTTACCCACCGCGAAACGTCTCCATAGACCCGGTCCGTCGCATCC-CCACATGAACCT
                ************************* * ******************* *  **** **

TaDrAp2-A1      TCTACTACGGTCTTAGCGCCGCACGTGAGGCCAAGGCCTATGCGCCGGGTCCAGGAGCTT
TaDrAp2-D1      ----CTACGGTCTTAGCGCCGCACGTGAGGCTAAGGCCTATGCGCTGGGTCCAGGAGCTT
TaDrAp2-B1      T---CTACGGTCTTAGCGCCGCACGTGAGGCTAAGACCTATGCACCGGGTCCAGGAGCTT
                    *************************** *** ******* * **************

TaDrAp2-A1      AGCGTCCATGCGCCGGAGAGTCCGCGCACCTCTGCCCGCTTCGCGCGGCCATTGGCCGCG
TaDrAp2-D1      AGCGTCCACGCGCCGGAGAGTTCGCGCACCGCTGCCCGCTTCGCGCGGCCATTGGCCGCG
TaDrAp2-B1      AGCCTCCACGCGCCGGAGAGCTCGCGCACCTCTGCCCGCTTCGCGCGGCCATTGGCCGCG
                *** **** ***********  ******** *****************************

TaDrAp2-A1      CTGTTTATGGGGGAAAAAATAGTTTGTTTTTATTTTCTCCCGTAAAAAAAGTGAAAAGAT
TaDrAp2-D1      CTGTTTATGGGGAAAAAAATAGTTTGTTTTTATTTTTCCCCGTAAAAAAAGTGAAAAGAT
TaDrAp2-B1      CTGTTTATGGGGGAAAAA----------TATATTTTTTCCCGTAAAAAAAGTGAAAAGAT
                ************ *****          * ******  **********************

TaDrAp2-A1      AGCGATAACGGAAACCCTAATCCCCAAATCAGAAATCTATCCGAATCGCTATTTATGTTT
TaDrAp2-D1      AGCGATAACGGAAACCCTAATCCCCAAATCAGAAATCTATCCGAATCGCTATTTATGTTT
TaDrAp2-B1      AGCGATAACGGAAACCCTAATCCCCAAATCAGAAATCTATCCGAATCGCTATTTATGTTT
                ************************************************************

TaDrAp2-A1      GTGGCTTTCTCCCCCCTGCGATCCGATGTCCCCCGCTTCGCCCGCCGCGCCGATCCGACC
TaDrAp2-D1      GTGGCTTTCTCCCCCCTCCGATCCGATGTCCCCCGCTTCGTCCGCCGCGCCGATCCGACC
TaDrAp2-B1      GTGGCTTTCTCCCCCCTCCGATCCGATGTCCCCCGCTTCGTCCGCCGCGCCGATCCGACC
                ***************** ********************** *******************

TaDrAp2-A1      TCCCGCCGCCGCGTTCTCTCCGCCGGGGACGCTCTCTCCGGCGCCGCCGGCTCCGCGCCA
TaDrAp2-D1      TCCAGCCGCCGCGTTCTCTCCGCCGGGGACGCTCTCTCCGACGCCGCCGGCTCCGCGCCA
TaDrAp2-B1      TCCAGCCGCCGCATTCTCTCCGCCGGGGACGCTCCCTCCGGCGCCGCCGGCTCCGCGCCA
                *** ******** ********************* ***** *******************

TaDrAp2-A1      TGAGGAAGAAGCTCGACACCCGCTTCCCCGCG
TaDrAp2-D1      TGAGGAAGAAGCTCGACACCCGCTTCCCCGCG
TaDrAp2-B1      TGAGGAAGAAGCTCGACACCCGCTTCCCCGCG
                ********************************
```

## Primers used for sequencing *TaDrAp2*:

**TaDrAp2-A1-seq-F: AATGCCGACACGCACGTCTGTC** 22 bp, 59%GC, Tm=58.6C
**TaDrAp2-A1-seq-R: AGCGGGCAGAGGTGCGCGG** 19 bp, 79%GC, Tm=61.9C
TaDrAp2-A1-seq-R (RevCom): CCGCGCACCTCTGCCCGCT
Amplicon size: 894 bp

**TaDrAp2-A1-seq-F2: TCCGCCACCTGAGGTTCTGC** 20 bp, 65%GC, Tm=57.9C
**TaDrAp2-A1-seq-R2: GGCAAAAAAAAATCGCAGGGGCAC** 24 bp, 50%GC, Tm=57.4C
TaDrAp2-A1-seq-R2 (RevCom): GTGCCCCTGCGATTTTTTTTTGCC
Amplicon size: 1,983+66=2,049 bp

**TaDrAp2-B1-seq-F: GTGTTCCATGCCCTACTCTACCT** 23 bp, 52%GC, Tm=57.1C
**TaDrAp2-B1-seq-R: CGGGCAGAGGTGCGCGAG** 18 bp, 78%GC, Tm=59.4C
TaDrAp2-B1-seq-R (RevCom): CTCGCGCACCTCTGCCCG
Amplicon size: 908 bp

**TaDrAp2-B1-seq-F2: GCTACAATAACCAGCCAACCAGA** 23 bp, 48%GC, Tm=55.3C
**TaDrAp2-B1-seq-R2: CCTGAACCGGCAAAAAATCGCAT** 23 bp, 48%GC, Tm=55.3C
TaDrAp2-B1-seq-R2 (RevCom): ATGCGATTTTTTGCCGGTTCAGG
Amplicon size: 2,003+71=2,074 bp

**TaDrAp2-D1-seq-F: TTGAGGAGGAGGAGGAGCGC** 20 bp, 65%GC, Tm=57.9C
**TaDrAp2-D1-seq-R: ACGCTAAGCTCCTGGACCCA** 20 bp, 60%GC, Tm=55.9C
TaDrAp2-D1-seq-R (RevCom): TGGGTCCAGGAGCTTAGCGT
Amplicon size: 912 bp

**TaDrAp2-D1-seq-F2: CTTCCCTGGCGCAGCCCT** 18 bp, 72%GC, Tm=57.2C
**TaDrAp2-D1-seq-R2: CCGGCAAAAAAAAAACGCAGGGA** 23 bp, 49%GC, Tm=55.3C
TaDrAp2-D1-seq-R2 (RevCom): TCCCTGCGTTTTTTTTTTGCCGG
Amplicon size: 1,943+68=2,011 bp

## *TaDrAp2* - First intron

***>TaDrAp2-A1***

GCTCTCTCCGGCGCCGCCGGCTCCGCGCCATGAGGAAGAAGCTCGACACCCGCTTCCCCGCGGTAT
GACCCGTGCCCCTGCGATTTTTTTTTGCCGGTTCAGGCCTCCCCCCCACGCTGTGCGCGCCGTCG
ATTTCGCGATTTTTTCTGTGTGCTTCGTCTAGGTTTTGTTTCCGGCGGTTAGGTTTCTGTAAATTG
ACGGCCAATGGACCTGCGATATGCATGTTTGTCTGTTTCTCTCTATTGCTGGGCTTGATAGATGCA
CGTTTGGGGAATTTTTGGGTGCTGTCGCTAAATAATATGTAGATTTCGCAGCACGGTTGGAATTTT
TAGCCTCTCGCGTGTGCTCGTCCTGGGCTTGTTTCATGTACGTTTGTTTTTATGGTGGCTATGGTT
CGCTGTCCCGATGGCAATGAAGCTGCGGTTATAGATGGGCATATGATGTAGGTGGTGGATGCGTGA
AAAATCCTGGATTATGTAGATTCTGCCTACCCTGTTAACGTTTCACTAGTATATGGCTTTCCGCGG
ACGAGTGTGCTATTAATCTACTTTATTATTTAATATTAGGGTCAATTTATTTTATGTTTGACTAAT
TCGTTATGATTAAATCCTTGGGTCATCCTTGTGCTTCAATTGCTAGGCTTGTTCTTTAAGGAACGA
AGTTAACAGAAATTACTTGCATTCACTATTTACTGAAAAAAGATGGACATGATTATTAAATAACTC
TTTTATAAATATGAGCTTTTTTTAGGAATGAGAGTTTAACTTAAGCGGATGTGGCTTAATTGTCTC
AAGAGATGATCAACTCCAGGTCTTCTGCGGTGTTCCCTATTTTGATCGGTAGCTTGCTTCAAACCG
GGCCGATTGTACCTAAACTAATATTCCCACATAATGAACACCAAGTGCTCCTTCCTTTTTTCCCAA
ATTAAAAGAGGAGCGTGATTGTTGGTACAAAGTGCTAACTATAGAATCAAGCATGTGAGTTCTTTT
TATAGTTGTTGATCTGTTGACATTGGTATCATGATTGTTTAGATATAGATTGTGGTGGCAACCTGG
CAGGGAAGCTTCTTAGCCTGATGAGTTAGTACTGTTCAGATTTTGTAGTATATTTCAAATGGTAAA
TACAGTAGGATTCTACTATTTAACATCTGGCCCACAACAGGCAATGAGTGTTGGTTAAAGCTGTGT
ACGATCTATAAAAATATATAGTGTAGAGTGTTTATCTTATTCTAAGCATGGTATATTCTCCGATCT
GTTTTTGGTCCATCTTCGGGGGAAGATGCAAGTGTTTTGATGACATATGTTTGCTGTACAATGGAA
TCCTCATGATACTAAAGTTTTTCTACAGTGGATAGAGATACCTTCTTCATTACCCTTTCTTTCACA
AGGGCTAGCAGAATCTCTTTTCTTCATCTGCTAGGACCTGTGCTTATAACCACATGTGAATACTAT
CGCTCTCATGTCCTTTTCCATCGAAGTTGCATCATTGGCCATTTGCACTGTAGTATGCAACAATCA
TAACTATGATTAATGAGTGTTTTATTATTGACATGTTGTCATAATTTGAAAATGGCAGATTCCTAC
TACTTTTCAGATGGTATCATGGTACTCTTCTGTTAATGTGATATCAGCTTCCTCTTGTACTTTGTT
ATACAGCCTCGGATTAAGAAGATCATGCAAGCAGATGAAGATGTCGGCAAGATTGCTCTAGCTGTA
CCTGTTCTAGTTT

***>TaDrAp2-B1***

GCATTCTCTCCGCCGGGGACGCTCCCTCCGGCGCCGCCGGCTCCGCGCCATGAGGAAGAAGCTCGA
CACCCGCTTCCCCGCGGTATGACCCATGCCCATGCGATTTTTTGCCGGTTCAGGCCTCCCCTATGC
TGTGCGCGCCGTCGATTTCGCGATTTTTTATGTGCCTCGTCTAGGTTTTGTTTCCAGCGGTTAGGT
TTTCGTAAATTGGCGGCCAATGGACCTGCGATGTGCATGTTTACATGTTTCTCTCCATGGCTGGGC
TTGTTAGATGCACGTTTGGGGATTTTTTTGGGTGCTGTCGGTAAATAGCATGTAGATTTCGCTGCA
CGGTTATGATTTTTAGCCTCTTGCGCGTGCTCATCCTGGGCTTGTTTCATGTACGTTTCTTTTATG
GTGGCTATGGTTCGCTGTCCTGATGGCAATGAAGCTGTGGTTATAGATGGGCATATGATGTAGGCA
GGATGCGTGGAAAATCCTGGATTGTGTAGATTCTGCCTACTCTGGTAATGTTTCACTAGTATATGG
CTTTGCCGCCGACGAGAGTGCTCTATTAATCTACTTTGTTATTTAACATCAGGGTCAATTTATTTT
ATGTTTGATTAATTCGTTATGATTGAATCCTTGGGTCAACCTTGTGCTTCAATTGCTAGGTTTGTT
CTCTAAGGAATGAAGGCAACCGAAATTACTTGCATTCACTATTTACTGAAAAAAGATGGACATGAT
TATTAAATAACTCTTTTATAAATATGAGTTTTTTTAGGAATGAGAGTTCAACTTCAGCGGAGATGG
CTTAATTGTCTCGAGAGAAGATCAACTCCAGGTCTTCTGCGGTGTTTCCTATTTTGGTCGGTAGCT
TGCTTCAAACCGGGCCGATTGTACCTAAACTAATATTCCCACATAATGAACACCAAGTGCTCCTTC
CTTTTTTCCTAAATTAAGAGAGGAGTGCGATTGTTGGTACAAAGTGCTAACTATAGAATCAAGCAT
GTGAGTTCTTTTTATAGTTGTTGATCTGTTGACATTGGTCTCATGATTGTTTAGATATAGATTGTG
GTGGCAGCCTGGCAGGGAAGCATCTTAGCCCGACGAGTTGGTACTGGTCAGATTTTGTAGTATATT
TCAAATGGTAAATAGACAGTAGGATTCTACTATTTAACATCTGGCCCACAACAGGCAATGAGTGTT

GGTTAAAGCTGTGTACAATCTATAAAAATATGTAGTGTAGAGTGTTTATTTTATTCTGAACATGGT
ATATTCTCTGATCTGTTTTTGGTCCATCTTTGCGGGAAGATGCAAGTGTTTTGATGACATATGTTT
GCTGTACAATGGAATCCTCATGATACTACAGTTTTTCTACAGTGGATAGAGATACCTTCTTCATTA
CTCTTTCTTTCACAAGGGCTAGCAGAATCTCTTTACCTCATCTGCTAGGACCTGTGCTTATAACGA
CATGTGAATACTATCACTGTCATGTCCTTTTCCATCGAAGTTGCATCGTTGGCCATTTGCACTGAA
GTATGCAACAATCATAATTATGATTAATGAGTGTTTTATTGATGACATGTTGTCATAATTTCAAAA
TGGCAGATTCCCACTACTTTTCAGATGGTATCATGGTACTCTTCTGTTAATGTGATATCAGCTTCC
TCTTGTACTTTGTTATACAGCCTCGGATTAAGAAGATCATGCAAGCAGATGAAGATGTCGGCAAGA
TTGCTCTAGCTGTACCTGTTCTAGTTT

***>TaDrAp2-D1***
CTCTCCGCCGGGGACGCTCTCTCCGACGCCGCCGGCTCCGCGCCATGAGGAAGAAGCTCGACACCC
GCTTCCCCGCGGTATGACCCATGTCCCTGCGTTTTTTTTTTGCCGGTTCGGGCCTCCCCCTACGCT
GTGCGCGCCGTCGATCTCGCGATTTTTTTGTGTGCTTCGTCTAGGTTTTGTTTCCGGCGGTTAGGA
TTTCGTAAATTGGCGGCCAATGGACCTGCGATATGCATGTTTACCTGTTTCTCTCCATGGCTGGGC
TTGATAGATGCACGTTTGGCGAATTTTTGGGCGCTGTCGGTAAATAGTATGTAGATTTCGCAGCAC
GGTTAGGATTTTTAGCCTCTCGCGTGTGCTCATCCTGGGCTTGTTTCATGTACGTTTGTTTTTATG
GTGGCTATGGTTCGCTGTCCCGATGGCAATGAAGCTGCGGTTATAGATGGGCATATATGATGTAGG
TGGTGGATGCGTGAAAAATCCTGGATTATGTGGATTCTGCCTACCCTGTTAACGTTTCACTAGTAT
ATTGCTTTCCGCGGACGAGAGTGTGCTATTAATCTACTTTATTATTTAATGTTAGGGTCAATTTAT
TTTATGTTTGAATAATTCGTTATGATTAAATCCTTGGGTCAACCTTGTGCTTCAATTGCTAGGCCT
TGTTCTTTAAGGAACGAAGTTAACAGAAATTACTTGCATTCACTATTTACTGAAAATAGATGGACA
TGAATATTAATAACTCTTTTATAAATATGAGCTTTTTTTAGGAATGAGAGTTTAACTTCAGCGGAG
GTGGCTTAATTGTCTCAAGAGATGATCAACACCAGGTCTTCTGCGGTGTTTCCTATTTTGATCGGT
AGCTTGCTTCAAACCGGGCCGATTGTACCTAAACTAATATTCCCACATAATGAACACCAAGTGCTC
CTTCCTTTTTTTCCTAAATTAAAAGAGGAGCACGATTGTTGGTACAAAGTGCTAACTATAGAATCA
AGCATGTGAGTTCTTTTTATAGTTGTTGATCTGTTGACATTGGTATCATAGATATAGATTGTGGTG
GCAACCTGGCAGGGAAGCTTCTTAGCCCGACGAGTTGGTACTGTTCAGATTTTGTAGTATATTTCA
AATGGTAAATAGACAGTAGGATTCTACTATTTGACATCTGGCCCACAACAGGCAATGAGTGTTGGT
TAAAGCTGTGTACAATCTATAAAAATATGTAGTGTAGAGTGTTTATCTTATTATGAGCATGGTATA
TTCTCCAATCTGTTTTTGGTCCATCTTTGGGGGAAGATGCAAGTGTTTTGATGACATACGTTTGCT
GTACAATGGAATCCTCATGATACTAAAGTTTTTCTACAGTGGATAGAGATACCTTCTTCATTACCC
TTTCTTTCACAAGGGCTAGTAGAATCTATTTTCTTCATCTGCTAGGACCTGTGCTCATAACCACAT
GTGAATACTATCGCTGTCATGTCCTTTTCCATCGAAGTTGCATCGTAGGCCATTTGCACTGTAGTA
TGCAACAATCATAATTATGATTAATGAGTGTTTTATTGATGACATGTTGTCATAATTTCAAAATGG
CAGATTCCTACTACTTTTCAGATGGTATCATGGTACTCTTCTGTTAATGTGACATCAGCTTCCTCT
TGTATTTTGTTATACAGCCTCGGATTAAGAAGATCATGCAAGCAGATGAAGATGTCGGCAAGATTG
CTCTAGCAGTACCTGTTCTAGTTT

## *TaDrAp2* - Fist intron

### CLUSTAL 2.1 multiple sequence alignment

```
TaDrAp2-A1      --------------------GCTCTCTCCGGCGCCGCCGGCTCCGCGCCATGAGGAAGAA
TaDrAp2-D1      -----CTCTCCGCCGGGGACGCTCTCTCCGACGCCGCCGGCTCCGCGCCATGAGGAAGAA
TaDrAp2-B1      GCATTCTCTCCGCCGGGGACGCTCCCTCCGGCGCCGCCGGCTCCGCGCCATGAGGAAGAA
                                    **** ***** *****************************

TaDrAp2-A1      GCTCGACACCCGCTTCCCCGCGGTATGACCCGTGCCCCTGCGATTTTTTTTTGCCGGTTC
TaDrAp2-D1      GCTCGACACCCGCTTCCCCGCGGTATGACCCATGTCCCTGCGTTTTTTTTTTGCCGGTTC
TaDrAp2-B1      GCTCGACACCCGCTTCCCCGCGGTATGACCCATGCCCATGCGATTTT---TTGCCGGTTC
                ******************************* ** ** **** ****   **********

TaDrAp2-A1      AGGCCTCCCCCCCCACGCTGTGCGCGCCGTCGATTTCGCGATTTTTTCTGTGTGCTTCGT
TaDrAp2-D1      GGGCCTCCCCCT--ACGCTGTGCGCGCCGTCGATCTCGCGATTTTTT-TGTGTGCTTCGT
TaDrAp2-B1      AGGCCTCCCCT---ATGCTGTGCGCGCCGTCGATTTCGCGATTTTTT--ATGTGCCTCGT
                 *********    * ****************** ************   ***** ****

TaDrAp2-A1      CTAGGTTTTGTTTCCGGCGGTTAGGTTTCTGTAAATTGACGGCCAATGGACCTGCGATAT
TaDrAp2-D1      CTAGGTTTTGTTTCCGGCGGTTAGGATTTCGTAAATTGGCGGCCAATGGACCTGCGATAT
TaDrAp2-B1      CTAGGTTTTGTTTCCAGCGGTTAGGTTTTCGTAAATTGGCGGCCAATGGACCTGCGATGT
                *************** ********* **  ******** ******************* *

TaDrAp2-A1      GCATGTTTGTCTGTTTCTCTCTATTGCTGGGCTTGATAGATGCACGTTTGGGGAATTTTT
TaDrAp2-D1      GCATGTTTACCTGTTTCTCTCCATGGCTGGGCTTGATAGATGCACGTTTGGCGAATTTTT
TaDrAp2-B1      GCATGTTTACATGTTTCTCTCCATGGCTGGGCTTGTTAGATGCACGTTTGGGGATTTTTT
                ********   ********** ** ********** *************** ** *****

TaDrAp2-A1      -GGGTGCTGTCGCTAAATAATATGTAGATTTCGCAGCACGGTTGGAATTTTTAGCCTCTC
TaDrAp2-D1      -GGGCGCTGTCGGTAAATAGTATGTAGATTTCGCAGCACGGTTAGGATTTTTAGCCTCTC
TaDrAp2-B1      TGGGTGCTGTCGGTAAATAGCATGTAGATTTCGCTGCACGGTTATGATTTTTAGCCTCTT
                 *** ******* ******  ************* ********   *************

TaDrAp2-A1      GCGTGTGCTCGTCCTGGGCTTGTTTCATGTACGTTTGTTTTTATGGTGGCTATGGTTCGC
TaDrAp2-D1      GCGTGTGCTCATCCTGGGCTTGTTTCATGTACGTTTGTTTTTATGGTGGCTATGGTTCGC
TaDrAp2-B1      GCGCGTGCTCATCCTGGGCTTGTTTCATGTACGTTT-CTTTTATGGTGGCTATGGTTCGC
                *** ****** *************************  **********************

TaDrAp2-A1      TGTCCCGATGGCAATGAAGCTGCGGTTATAGATGGGCATAT--GATGTAGGTGGTGGATG
TaDrAp2-D1      TGTCCCGATGGCAATGAAGCTGCGGTTATAGATGGGCATATATGATGTAGGTGGTGGATG
TaDrAp2-B1      TGTCCTGATGGCAATGAAGCTGTGGTTATAGATGGGCATAT--GATGTAGGCAG--GATG
                ***** **************** ******************  ********  *  ****

TaDrAp2-A1      CGTGAAAAATCCTGGATTATGTAGATTCTGCCTACCCTGTTAACGTTTCACTAGTATATG
TaDrAp2-D1      CGTGAAAAATCCTGGATTATGTGGATTCTGCCTACCCTGTTAACGTTTCACTAGTATATT
TaDrAp2-B1      CGTGGAAAATCCTGGATTGTGTAGATTCTGCCTACTCTGGTAATGTTTCACTAGTATATG
                **** ************* *** ************ *** *** ***************

TaDrAp2-A1      GCTTT-CCGCGGACGAG--TGTGCTATTAATCTACTTTATTATTTAATATTAGGGTCAAT
TaDrAp2-D1      GCTTT-CCGCGGACGAGAGTGTGCTATTAATCTACTTTATTATTTAATGTTAGGGTCAAT
TaDrAp2-B1      GCTTTGCCGCCGACGAGAGTGCTCTATTAATCTACTTTGTTATTTAACATCAGGGTCAAT
                ***** **** ******  **  *************** ********  * *********

TaDrAp2-A1      TTATTTTATGTTTGACTAATTCGTTATGATTAAATCCTTGGGTCATCCTTGTGCTTCAAT
TaDrAp2-D1      TTATTTTATGTTTGAATAATTCGTTATGATTAAATCCTTGGGTCAACCTTGTGCTTCAAT
TaDrAp2-B1      TTATTTTATGTTTGATTAATTCGTTATGATTGAATCCTTGGGTCAACCTTGTGCTTCAAT
                *************** *************** ************* **************
```

```
TaDrAp2-A1      TGCTAGGC-TTGTTCTTTAAGGAACGAAGTTAACAGAAATTACTTGCATTCACTATTTAC
TaDrAp2-D1      TGCTAGGCCTTGTTCTTTAAGGAACGAAGTTAACAGAAATTACTTGCATTCACTATTTAC
TaDrAp2-B1      TGCTAGGT-TTGTTCTCTAAGGAATGAAGGCAACCGAAATTACTTGCATTCACTATTTAC
                *******  ******* ******* ****  *** *************************

TaDrAp2-A1      TGAAAAAAGATGGACATGATTATTAAATAACTCTTTTATAAATATGAGCTTTTTTTAGGA
TaDrAp2-D1      TGAAAATAGATGGACATGAATATTAA-TAACTCTTTTATAAATATGAGCTTTTTTTAGGA
TaDrAp2-B1      TGAAAAAAGATGGACATGATTATTAAATAACTCTTTTATAAATATGAG-TTTTTTTAGGA
                ****** ************ ****** ********************* ***********

TaDrAp2-A1      ATGAGAGTTTAACTTAAGCGGATGTGGCTTAATTGTCTCAAGAGATGATCAACTCCAGGT
TaDrAp2-D1      ATGAGAGTTTAACTTCAGCGGAGGTGGCTTAATTGTCTCAAGAGATGATCAACACCAGGT
TaDrAp2-B1      ATGAGAGTTCAACTTCAGCGGAGATGGCTTAATTGTCTCGAGAGAAGATCAACTCCAGGT
                ********* ***** ******  *************** ***** ******* ******

TaDrAp2-A1      CTTCTGCGGTGTTCCCTATTTTGATCGGTAGCTTGCTTCAAACCGGGCCGATTGTACCTA
TaDrAp2-D1      CTTCTGCGGTGTTTCCTATTTTGATCGGTAGCTTGCTTCAAACCGGGCCGATTGTACCTA
TaDrAp2-B1      CTTCTGCGGTGTTTCCTATTTTGGTCGGTAGCTTGCTTCAAACCGGGCCGATTGTACCTA
                ************* ********* ************************************

TaDrAp2-A1      AACTAATATTCCCACATAATGAACACCAAGTGCTCCTTCCTTTTTTCCC-AAATTAAAAG
TaDrAp2-D1      AACTAATATTCCCACATAATGAACACCAAGTGCTCCTTCCTTTTTTTCCTAAATTAAAAG
TaDrAp2-B1      AACTAATATTCCCACATAATGAACACCAAGTGCTCCTTCCTTTTTTCCT-AAATTAAGAG
                ********************************************** *  ******* **

TaDrAp2-A1      AGGAGCGTGATTGTTGGTACAAAGTGCTAACTATAGAATCAAGCATGTGAGTTCTTTTTA
TaDrAp2-D1      AGGAGCACGATTGTTGGTACAAAGTGCTAACTATAGAATCAAGCATGTGAGTTCTTTTTA
TaDrAp2-B1      AGGAGTGCGATTGTTGGTACAAAGTGCTAACTATAGAATCAAGCATGTGAGTTCTTTTTA
                *****   ****************************************************

TaDrAp2-A1      TAGTTGTTGATCTGTTGACATTGGTATCATGATTGTTTAGATATAGATTGTGGTGGCAAC
TaDrAp2-D1      TAGTTGTTGATCTGTTGACATTGGTATCAT--------AGATATAGATTGTGGTGGCAAC
TaDrAp2-B1      TAGTTGTTGATCTGTTGACATTGGTCTCATGATTGTTTAGATATAGATTGTGGTGGCAGC
                ************************* ****        ******************** *

TaDrAp2-A1      CTGGCAGGGAAGCTTCTTAGCCTGATGAGTTAGTACTGTTCAGATTTTGTAGTATATTTC
TaDrAp2-D1      CTGGCAGGGAAGCTTCTTAGCCCGACGAGTTGGTACTGTTCAGATTTTGTAGTATATTTC
TaDrAp2-B1      CTGGCAGGGAAGCATCTTAGCCCGACGAGTTGGTACTGGTCAGATTTTGTAGTATATTTC
                ************* ******** ** ***** ****** *********************

TaDrAp2-A1      AAATGGTAAATA--CAGTAGGATTCTACTATTTAACATCTGGCCCACAACAGGCAATGAG
TaDrAp2-D1      AAATGGTAAATAGACAGTAGGATTCTACTATTTGACATCTGGCCCACAACAGGCAATGAG
TaDrAp2-B1      AAATGGTAAATAGACAGTAGGATTCTACTATTTAACATCTGGCCCACAACAGGCAATGAG
                ************  ******************* **************************

TaDrAp2-A1      TGTTGGTTAAAGCTGTGTACGATCTATAAAAATATATAGTGTAGAGTGTTTATCTTATTC
TaDrAp2-D1      TGTTGGTTAAAGCTGTGTACAATCTATAAAAATATGTAGTGTAGAGTGTTTATCTTATTA
TaDrAp2-B1      TGTTGGTTAAAGCTGTGTACAATCTATAAAAATATGTAGTGTAGAGTGTTTATTTTATTC
                ******************** ************** ***************** *****

TaDrAp2-A1      TAAGCATGGTATATTCTCCGATCTGTTTTTGGTCCATCTTCGGGGGAAGATGCAAGTGTT
TaDrAp2-D1      TGAGCATGGTATATTCTCCAATCTGTTTTTGGTCCATCTTTGGGGGAAGATGCAAGTGTT
TaDrAp2-B1      TGAACATGGTATATTCTCTGATCTGTTTTTGGTCCATCTTTGCGGGAAGATGCAAGTGTT
                * * **************  ******************** * *****************

TaDrAp2-A1      TTGATGACATATGTTTGCTGTACAATGGAATCCTCATGATACTAAAGTTTTTCTACAGTG
TaDrAp2-D1      TTGATGACATACGTTTGCTGTACAATGGAATCCTCATGATACTAAAGTTTTTCTACAGTG
TaDrAp2-B1      TTGATGACATATGTTTGCTGTACAATGGAATCCTCATGATACTACAGTTTTTCTACAGTG
                *********** ******************************** ***************
```

```
TaDrAp2-A1      GATAGAGATACCTTCTTCATTACCCTTTCTTTCACAAGGGCTAGCAGAATCTCTTTTCTT
TaDrAp2-D1      GATAGAGATACCTTCTTCATTACCCTTTCTTTCACAAGGGCTAGTAGAATCTATTTTCTT
TaDrAp2-B1      GATAGAGATACCTTCTTCATTACTCTTTCTTTCACAAGGGCTAGCAGAATCTCTTTACCT
                *********************** ******************** ******* *** * *

TaDrAp2-A1      CATCTGCTAGGACCTGTGCTTATAACCACATGTGAATACTATCGCTCTCATGTCCTTTTC
TaDrAp2-D1      CATCTGCTAGGACCTGTGCTCATAACCACATGTGAATACTATCGCTGTCATGTCCTTTTC
TaDrAp2-B1      CATCTGCTAGGACCTGTGCTTATAACGACATGTGAATACTATCACTGTCATGTCCTTTTC
                ******************** ***** **************** ** *************

TaDrAp2-A1      CATCGAAGTTGCATCATTGGCCATTTGCACTGTAGTATGCAACAATCATAACTATGATTA
TaDrAp2-D1      CATCGAAGTTGCATCGTAGGCCATTTGCACTGTAGTATGCAACAATCATAATTATGATTA
TaDrAp2-B1      CATCGAAGTTGCATCGTTGGCCATTTGCACTGAAGTATGCAACAATCATAATTATGATTA
                *************** * ************** ****************** ********

TaDrAp2-A1      ATGAGTGTTTTATTATTGACATGTTGTCATAATTTGAAAATGGCAGATTCCTACTACTTT
TaDrAp2-D1      ATGAGTGTTTTATTGATGACATGTTGTCATAATTTCAAAATGGCAGATTCCTACTACTTT
TaDrAp2-B1      ATGAGTGTTTTATTGATGACATGTTGTCATAATTTCAAAATGGCAGATTCCCACTACTTT
                **************  ******************* *************** ********

TaDrAp2-A1      TCAGATGGTATCATGGTACTCTTCTGTTAATGTGATATCAGCTTCCTCTTGTACTTTGTT
TaDrAp2-D1      TCAGATGGTATCATGGTACTCTTCTGTTAATGTGACATCAGCTTCCTCTTGTATTTTGTT
TaDrAp2-B1      TCAGATGGTATCATGGTACTCTTCTGTTAATGTGATATCAGCTTCCTCTTGTACTTTGTT
                *********************************** ***************** ******

TaDrAp2-A1      ATACAGCCTCGGATTAAGAAGATCATGCAAGCAGATGAAGATGTCGGCAAGATTGCTCTA
TaDrAp2-D1      ATACAGCCTCGGATTAAGAAGATCATGCAAGCAGATGAAGATGTCGGCAAGATTGCTCTA
TaDrAp2-B1      ATACAGCCTCGGATTAAGAAGATCATGCAAGCAGATGAAGATGTCGGCAAGATTGCTCTA
                ************************************************************

TaDrAp2-A1      GCTGTACCTGTTCTAGTTT
TaDrAp2-D1      GCAGTACCTGTTCTAGTTT
TaDrAp2-B1      GCTGTACCTGTTCTAGTTT
                ** ****************
```

# Supplementary file 4

## Sequences, BLAST and primer design, Universal probes and sizes of amplicons generated

### TaDrAp1-B4

**>CS**

CAATCACTCGCGAACGACCAGACGTCCGGTATTGCGTGTGCGCATCCCGACGGTTTTCAGCCCATG
TACCCCTCTTTCACACCTAGACTATAAATACCCTTCTCCTACCTCCATCCTAGGGCTGGCAAAGAC
ATAATAGAGAATTGCTAGAGCTTTGCTCATATTCATTATTCCTCTTGGAGGTCAAGACCTCCATGT
GAGAAGATCCCCCTAGTGGATGCAAGAGCCCTATAAGGGAATATCAATCTAGGGATGCAAGACCCC
TATATGGGAAGATCCTCCATGGATGCTAGATCTCATCTCCTTTGGATTTGAGAAGAACCTTTACCT
TTGTGCTTCCCCCTTTTGACTTGTTAATATATATTTGTGGATCTCATGTATGCTATCTTAGTGATG
TGTGATTTGGATTTGTTTGAGTCTCTCTCTCTCTCTCTTGTGTTATTGAGTGTTCATCCTTTCTCC
ATCCTCCAAGTGCGAAAAGATCTTGAATCTAGGGTTTTGCCCTACATCACCATGCCTCCCTAGCCC
CCCTCTCCTCTCCTCCTTCCCTTCCCGCAGGTCACTGACCTCCTACTTGTGTCGGGTCCCTCTCTT
CCTTCCTTCCCCGTAGGTCGTTGAGCAAGGAATCGAACTGGGCAAACGTGACGGCCGGCCGGATCG
ATGCTGAATTGACCGGATCCGACGTAGGGATCACAACAAGGAGGAGGAAGTCGGAGGAAGGAACGG
TGGCGGGCAGGGGCGGCGGCTACAATGTCGTCGGTGGAAGGAGCGACAATGGAGGCTCCGCCGACC
ATGGCGAGGAAAGCGAGATCGGCGATGAGCTTCGCGGGGTCCGCTGCGGGGTCAGAGGAGCTGGCC
ATGGCGACGCGGTGGCCGGCGGGAAAGCGGAGGCAACATTCCGGTTGCGCGCGGGAAGAAAAGGGT
AGCGATTATTTTTCGTGATGCAAGCACTCTTTTGTGGCAGTCGCGTGACTACCCTATATTCGCTAC
ACTCGAAGCATTTTTTTTAGGTTGCCGGATTTTTTCAAGAAAATTTAGAATTTGATAGCTTTTCGT
GCTTAAAAACAATTCGTGTGTGAAAATAATAGGGCTACTGTGAGAGATGCGGTAAATATCTTTTGG
CACGAGTCCGCCGTGTTGGTGCAAGTTGGTGGAAAGACA

>18-4-Female

GACCAGACGTTCCGGTTATTGGCGTGTGGCGCATCCCCGACGGTTTTTCAGCCCCATGTACCCCTT
CTTTTCACAACCTAGAACTATAAAATACCCTTTTTCCTACCCTCCCATCCTAGGGCTGGCAAAGAC
ATAATAGAGAAATTGCTAGAGCTTTGCTCATATTCATTATTCCTCTTGGAGGTCAAGACCTCCCAT
GTGAGAAGATCCCCCCTAGTGGATGCAAGAGCCCTATAAGGGAATATCAATCTAGGGATGCAAGAC
CCCTATATGGGAAGATCCTCCATGGATGCTAGATCTCATCTCCTTTGGATTTGAGAAGAACCTTTA
CCTTTGTGCTTCCCCCTTTTGACTTGTTAATATATATTTGTGGATCTCATGTATGCTATCTTAGTG
ATGTGTGATTTGGATTTGTTTGAGTCTCTCTCTCTCTCTCTTGTGTTATTGAGTGTTCATCCTTTC
TCCACCTCCAAGTGCGAAAAGATCTTGAATCTAGGGTTTTGCCCTACATCACCATGCCTCCCTAGC
CCCCCTCTCCTCTCCTCCTTCCCTTCCCGCAGGTCACTGACCTCCTACTTGTGTCGGGTCCCTCTC
TTCCTTCCTTCCCCGTAGGTCGTTGAGCAAGGAATCGAACTGGGCAAACGTGACGGCCGGCCGGAT
CGATGCTGAATTGACCGGATCCGACGTAGGGATCACAACAAGGAGGAGGAAGTCGGAGGAAGGAAC
GGTGGCGGGCAGGGGCGGCGGCTACAATGTCGTCGGTGGAAGGAGCGACAATGGAGGCTCCGCCGA
CCATGGCGAGGAAAGCGAGATCGGCGATGAGCTTCGCGGGGTCCGCTGCGGGGTCAGAGGAGCTGG
CCATGGCGACGCGGTGGCCGGCGGCGAATAGGCGGACCGCAACATTTCCGGTT

>18-5-Male

GTCAAAGACCTCCCATGTGAGAAAGATCCCCCCTTAGTGGATGCAAAGAGCCCCTATAAAGGGAAT
ATCAAATCTAGGGGATGCAAGACCCCTTATATGGGAAGATCCTCCATGGATGCTAGATATCATCTC
CTTTGGATGTGAGAAGAACCTTTACCTTTGTGCTTCCCCCCTTTTGACTTGTTAATATATATCTGT
GGATCTCACGTTTGCTATTTTAGTGATGTGTGATTTGGATTTGTATGAGTCTCTCTCTCTCTCTCT
TGTGTTACTGAGTGTTCATCCTTTCTCCACCTCCAAGTGAGAAAAGATCTTGAATCTAGGGTTTTG
CCATACATCACCATGCCTCCCTAGCCCCCCTCTCCTCTCCTCCTTCCCTTCCCGCAGGTCACTGAC

CTCCTACTTGTGTCGGGTCCCTCTCTTCCTTCCTTCCCCGTAGGTCGTTGAGCAAGGAATCGAACT
GGGCAAACGTGACGGCCGGCCGGATCGATGCTGAATTGACCGGATCCGACGTAGGGATCACAACAA
GGAGGAGGAAGTCGGAGGAAGGAACGGTGGCGGGCAGGGGCGGCGGCTACAATGTCGTCGCTGGAA
GGAGCGACAATGGAGGCTCCGCCGACCATGGCGAGGAAAGCGAGATCGGCGATGAGCTTCGCGGGG
TCCGCTGCGGGGTCAGAGGAGCTGGCCATGGCGACGCGGTGGCC

**CLUSTAL 2.1 multiple sequence alignment**

## TaDrAp1-B4

```
18-4-Female        GATCCTCCATGGATGCTAGATCTCATCTCCTTTGGATTTGAGAAGAACCTTTACCTTTGT
18-5-Male          GATCCTCCATGGATGCTAGATCTCATCTCCTTTGGATTTGAGAAGAACCTTTACCTTTGT
CS                 GATCCTCCATGGATGCTAGATCTCATCTCCTTTGGATTTGAGAAGAACCTTTACCTTTGT
                   ************************************************************

18-4-Female        GCTTCCCCCTTTTGACTTGTTAATATATATTTGTGGATCTCATGTATGCTATCTTAGTG
18-5-Male          GCTTCCCCCTTTTGACTTGTTAATATATATTTGTGGATCTCATGTATGCTATCTTAGTG
CS                 GCTTCCCCCTTTTGACTTGTTAATATATATTTGTGGATCTCATGTATGCTATCTTAGTG
                   ***********************************************************

18-4-Female        ATGTGTGATTTGGATTTGTTTGAGTCTCTCTCTCTCTCTCTTGTGTTATTGAGTGTTCAT
18-5-Male          ATGTGTGATTTGGATTTGTTTGAGTCTCTCTCTCTCTCTCTTGTGTTATTGAGTGTTCAT
CS                 ATGTGTGATTTGGATTTGTTTGAGTCTCTCTCTCTCTCTCTTGTGTTATTGAGTGTTCAT
                   ************************************************************

18-4-Female        CCTTTCTCCATCCTCCAAGTGCGAAAAGATCTTGAATCTAGGGTTTTGCCCTACATCACC
18-5-Male          CCTTTCTCCATCCTCCAAGTGCGAAAAGATCTTGAATCTAGGGTTTTGCCATACATCACC
CS                 CCTTTCTCCATCCTCCAAGTGCGAAAAGATCTTGAATCTAGGGTTTTGCCCTACATCACC
                   ************************************************** *********

18-4-Female        ATGCCTCCCTAGCCCCCCTCTCCTCTCCTCCTTCCCTTCCCGCAGGTCACTGACCTCCTA
18-5-Male          ATGCCTCCCTAGCCCCCCTCTCCTCTCCTCCTTCCCTTCCCGCAGGTCACTGACCTCCTA
CS                 ATGCCTCCCTAGCCCCCCTCTCCTCTCCTCCTTCCCTTCCCGCAGGTCACTGACCTCCTA
                   ************************************************************

18-4-Female        CTTGTGTCGGGTCCCTCTCTTCCTTCCTTCCCCGTAGGTCGTTGAGCAAGGAATCGAACT
18-5-Male          CTTGTGTCGGGTCCCTCTCTTCCTTCCTTCCCCGTAGGTCGTTGAGCAAGGAATCGAACT
CS                 CTTGTGTCGGGTCCCTCTCTTCCTTCCTTCCCCGTAGGTCGTTGAGCAAGGAATCGAACT
                   ************************************************************

18-4-Female        GGGCAAACGTGACGGCCGGCCGGATCGATGCTGAATTGACCGGATCCGACGTAGGGATCA
18-5-Male          GGGCAAACGTGACGGCCGGCCGGATCGATGCTGAATTGACCGGATCCGACGTAGGGATCA
CS                 GGGCAAACGTGACGGCCGGCCGGATCGATGCTGAATTGACCGGATCCGACGTAGGGATCA
                   ************************************************************

18-4-Female        CAACAAGGAGGAGGAAGTCGGAGGAAGGAACGGTGGCGGGCAGGGGCGGCGGCTACAATG
18-5-Male          CAACAAGGAGGAGGAAGTCGGAGGAAGGAACGGTGGCGGGCAGGGGCGGCGGCTACAATG
CS                 CAACAAGGAGGAGGAAGTCGGAGGAAGGAACGGTGGCGGGCAGGGGCGGCGGCTACAATG
                   ************************************************************

18-4-Female        TCGTCGGTGGAAGGAGCGACAATGGAGGCTCCGCCGACCATGGCGAGGAAAGCGAGATCG
18-5-Male          TCGTCGGTGGAAGGAGCGACAATGGAGGCTCCGCCGACCATGGCGAGGAAAGCGAGATCG
CS                 TCGTCGGTGGAAGGAGCGACAATGGAGGCTCCGCCGACCATGGCGAGGAAAGCGAGATCG
                   ************************************************************

18-4-Female        GCGATGAGCTTCGCGGGGTCCGCTGCGGGGTCAGAGGAGCTGGCCATGGCGACGCGGTGG
18-5-Male          GCGATGAGCTTCGCGGGGTCCGCTGCGGGGTCAGAGGAGCTGGCCATGGCGACGCGGTGG
CS                 GCGATGAGCTTCGCGGGGTCCGCTGCGGGGTCAGAGGAGCTGGCCATGGCGACGCGGTGG
                   ************************************************************
```

## TaDrAp1-B4

**Primers:**

**DrAp1-B4-SNP-F1: CTAGGGAGGCATGGTGATGTAG** 22 bp, 55%GC, 56.7C
DrAp1-B4-SNP-F1 (RevCom): CTACATCACCATGCCTCCCTAG

**DrAp1-B4-SNP-F2: CTAGGGAGGCATGGTGATGTAT** 22 bp, 50%GC, 54.8C
DrAp1-B4-SNP-F2 (RevCom): ATACATCACCATGCCTCCCTAG

**DrAp1-B4-SNP-R: GTGGATCTCATGTATGCTATCTTAG** 25 bp, 40%GC, 54.4C

Amplicon size: 159 bp

**Ordered primers:**
**DrAp1-B4-SNP-F1:** GAAGGTGACCAAGTTCATGCT**CTAGGGAGGCATGGTGATGTAG**
**DrAp1-B4-SNP-F2:** GAAGGTCGGAGTCAACGGATT**CTAGGGAGGCATGGTGATGTAT**
**DrAp1-B4-SNP-R: GTGGATCTCATGTATGCTATCTTAG**

## TaDrAp1-D4

**>CS**

GCCCATGTACCCCCTTTTCATCCCTAGACTATAAATACCCTTCTGCTAGCTCCATCCTAGGGCTGG
CAAAGACATAATAGAGAATTGATAGAGCTTTGCTCATATTCATTATTCCTCTTGGAGGTCAAGACA
TCCATGTGAGAAGATCCCCTAGTGGATGCAAGAGCCCTATAAGGGAAGATCCATCTAGGGATGCAA
GACCCCTATATGGGAAGATCCTCCATGGATGCTAGATCTCATCTCCTTTGGATTTGAGAAGAACCT
TTACCCTTGTGCTTCCCCCTTTTGACTTGTTCTTGCATATTTGTGGATCTCATGTATGCTATCTTA
GTGATGTGTGATTTGGATTTGTCTGAGTCTTTCTCTCTCTTGTGCTCTTGAGTGTTCATCCTTTCT
CCACCTCCAAGTGCGAAAAGATCTCAAATCTAGGGTTTTGCCCTACATCACCGTGCCTCCCTAGCC
CCTCCTCTCCTCTCCCCCTTCCTTCCCCGCAGGTCACTGACCTCCTACTCATGTCGGGTCCCTCTC
CTCCTTCCTTCCCCGTAGGTCGTTGAGCAAGAAATCGAGCTGGGCAAACGTGACGGCTGGCCGGAT
CAATGCCGAGTTGTCCGGATCCGACGCAGGGATCACAACAAGGAGGAGGAAGTCGGAGGAAAGAGA
GGTGGCGGGCAGAGGCGGCGGCGACGGTGTCGTCGGTGGAAGGAGCGGCAATGGAAGCTCCGCCGA
CCATGGCGAGGAAAGCGAGATCGGCGGTGAGCTTCGCGGGGTCGGTTGCAGGGCCAAAGGAGCTGG
CCATGGCGACGTGATGGCCGGCGGGAGAGCGGAGGCAGCGTTCCGGTTGCGCGCGGGTAGAAAAAG
GTAGCTATTGGGTTTCATGATGCAACCACTCTTTTGTGACAGCTGCCCGACTACTCTATATTCGCT
GCACTGAGGATTTTTAAAAAAAAA**T**TAGAATTCGGTAGCCTTTCGTGCTTAAAAACAATTCGTGTGT
GAAAATAATAGGGCTGGGAGATGCGGTAATTATCTTTTGGCACGAGTCCGCGTGCCGTGTAAGTTG
GTGGAAAGACAA

**>18-4-Female**

TTCATCCCTAGACTATAAATACCCTTCTGCTAGCTCCATCCTAGGGCTGGCAAAGACATAATAGAG
AATTGATAGAGCTTTGCTCATATTCATTATTCCTCTTGGAGGTCAAGACATCCATGTGAGAAGATC
CCCTAGTGGATGCAAGAGCCCTATAAGGGAAGATCCATCTAGGGATGCAAGACCCCTATATGGGAA
GATCCTCCATGGATGCTAGATCTCATCTCCTTTGGATTTGAGAAGAACCTTTACCCTTGTGCTTCC
CCCTTTTGACTTGTTCTTGCATATTTGTGGATCTCATGTATGCTATCTTAGTGATGTGTGATTTGG
ATTTGTCTGAGTCTTTCTCTCTCTTGTGCTCTTGAGTGTTCATCCTTTCTCCACCTCCAAGTGCGA
AAAGATCTCAAATCTAGGGTTTTGCCCTACATCACCGTGCCTCCCTAGCCCCTCCTCTCCTCTCCC
CCTTCCTTCCCCGCAGGTCACTGACCTCCTACTCATGTCGGGTCCCTCTCCTCCTTCCTTCCCCGT
AGGTCGTTGAGCAAGAAATCGAGCTGGGCAAACGTGACGGCTGGCCGGATCAATGCCGAGTTGTCC
GGATCCGACGCAGGGATCACAACAAGGAGGAGGAAGTCGGAGGAAAGAGAGGTGGCGGGCAGAGGC
GGCGGCGACGGTGTCGTCGGTGGAAGGAGCGGCAATGGAAGCTCCGCCGACCATGGCGAGGAAAGC
GAGATCGGCGGTGAGCTTCGCGGGGTCGGTTGCAGGGCCAAAGGAGCTGGCCATGGCGACGTGATG
GCCGGCGGGAGAGCGGAGGCAGCGTTCCGGTTGCGCGCGGGTAGAAAAAGGTAGCTATTGGGTTTC
ATGATGCAACCACTCTTTTGTGACAGCTGCCCGACTACTCTATATTCGCTGCACTGAGGATTTTTA
AAAAAAA**T**TAGAATTCGGTAGCCTTTCGTGCTTAAAAACAATTCGTGTGTGAAAATAATAGGGCTG
GGAGATGCGGT

**>18-5-Male**

CCCCATGTAACCCCCTTTTCATCCCTAGACTATAAATACCCTTCTGCTAGCTCCATCCTAGGGCTG
GCAAAGACATAATAGAGAATTGATAGAGCTTTTGTTCATATTCATTATTCCTCTTGGAGGTCAAGA
CATCCATGTGAGAAGATCCCCTAGTGGATGCAAGAGCCCTATAAGGGAAGATCCATCTAGGGATGC
AAGACCCCTATATGGGAAGATCCTCCATGGATGCTAGATCTCATCTCCTTTGGATTTGAGAAGAAC
CTTTACCCTTGTGCTTCCCCCTTTTGACTTGTTCTTGCATATTTGTGGATCTCATGTATGCTATCT
TAGTGATGTGTGATTTGGATTTGTCTGAGTCTTTCTCTCTCTTGTGCTCTTGAGTGTTCATCCTTT
CTCCACCTCCAAGTGCGAAAAGATCTCAAATCTAGGGTTTTGCCCTACATCACCGTGCCTCCCTAG
CCCCTCCTCTCCTCTCCCCCTTCCTTCCCCGCAGGTCACTGACCTCCTACTCATGTCGGGTCCCTC
TCCTCCTTCCTTCCCCGTAGGTCGTTGAGCAAGAAATCGAGCTGGGCAAACGTGACGGCTGGCCGG
ATCAATGCCGAGTTGTCCGGATCCGACGCAGGGATCACAACAAGGAGGAGGAAGTCGGAGGAAAGA

```
GAGGTGGCGGGCAGAGGCGGCGGCGACGGTGTCGTCGGTGGAAGGAGCGGCAATGGAAGCTCCGCC
GACCATGGCGAGGAAAGCGAGATCGGCGGTGAGCTTCGCGGGGTCGGTTGCAGGGCCAAAGGAGCT
GGCCATGGCGACGTGATGGCCGGCGGGAGAGCGGAGGCAGCGTTCCGGTTGCGCGCGGGTAGAAAA
AGGTAGCTATTGGGTTTCATGATGCAACCACTCTTTTGTGACAGCTGCCCGACTACTCTATATTCG
CTGCACTGAGGATTTTTAAAAAAAAAATAGAATTCGGTAGCCTTTCGTGCTTAAAAACAATTCGTGT
GTGAAAATATATAGGGCTGGGAGATTGCGGCT
```

**CLUSTAL 2.1 multiple sequence alignment**

## TaDrAp1-D4

```
CS                CTCAAATCTAGGGTTTTGCCCTACATCACCGTGCCTCCCTAGCCCCTCCTCTCCTCTCCC
18-4-Female       CTCAAATCTAGGGTTTTGCCCTACATCACCGTGCCTCCCTAGCCCCTCCTCTCCTCTCCC
18-5-Male         CTCAAATCTAGGGTTTTGCCCTACATCACCGTGCCTCCCTAGCCCCTCCTCTCCTCTCCC

CS                CCTTCCTTCCCCGCAGGTCACTGACCTCCTACTCATGTCGGGTCCCTCTCCTCCTTCCTT
18-4-Female       CCTTCCTTCCCCGCAGGTCACTGACCTCCTACTCATGTCGGGTCCCTCTCCTCCTTCCTT
18-5-Male         CCTTCCTTCCCCGCAGGTCACTGACCTCCTACTCATGTCGGGTCCCTCTCCTCCTTCCTT

CS                CCCCGTAGGTCGTTGAGCAAGAAATCGAGCTGGGCAAACGTGACGGCTGGCCGGATCAAT
18-4-Female       CCCCGTAGGTCGTTGAGCAAGAAATCGAGCTGGGCAAACGTGACGGCTGGCCGGATCAAT
18-5-Male         CCCCGTAGGTCGTTGAGCAAGAAATCGAGCTGGGCAAACGTGACGGCTGGCCGGATCAAT

CS                GCCGAGTTGTCCGGATCCGACGCAGGGATCACAACAAGGAGGAGGAAGTCGGAGGAAAGA
18-4-Female       GCCGAGTTGTCCGGATCCGACGCAGGGATCACAACAAGGAGGAGGAAGTCGGAGGAAAGA
18-5-Male         GCCGAGTTGTCCGGATCCGACGCAGGGATCACAACAAGGAGGAGGAAGTCGGAGGAAAGA

CS                GAGGTGGCGGGCAGAGGCGGCGGCGACGGTGTCGTCGGTGGAAGGAGCGGCAATGGAAGC
18-4-Female       GAGGTGGCGGGCAGAGGCGGCGGCGACGGTGTCGTCGGTGGAAGGAGCGGCAATGGAAGC
18-5-Male         GAGGTGGCGGGCAGAGGCGGCGGCGACGGTGTCGTCGGTGGAAGGAGCGGCAATGGAAGC

CS                TCCGCCGACCATGGCGAGGAAAGCGAGATCGGCGGTGAGCTTCGCGGGGTCGGTTGCAGG
18-4-Female       TCCGCCGACCATGGCGAGGAAAGCGAGATCGGCGGTGAGCTTCGCGGGGTCGGTTGCAGG
18-5-Male         TCCGCCGACCATGGCGAGGAAAGCGAGATCGGCGGTGAGCTTCGCGGGGTCGGTTGCAGG

CS                GCCAAAGGAGCTGGCCATGGCGACGTGATGGCCGGCGGGAGAGCGGAGGCAGCGTTCCGG
18-4-Female       GCCAAAGGAGCTGGCCATGGCGACGTGATGGCCGGCGGGAGAGCGGAGGCAGCGTTCCGG
18-5-Male         GCCAAAGGAGCTGGCCATGGCGACGTGATGGCCGGCGGGAGAGCGGAGGCAGCGTTCCGG

CS                TTGCGCGCGGGTAGAAAAAGGTAGCTATTGGGTTTCATGATGCAACCACTCTTTTGTGAC
18-4-Female       TTGCGCGCGGGTAGAAAAAGGTAGCTATTGGGTTTCATGATGCAACCACTCTTTTGTGAC
18-5-Male         TTGCGCGCGGGTAGAAAAAGGTAGCTATTGGGTTTCATGATGCAACCACTCTTTTGTGAC

CS                AGCTGCCCGACTACTCTATATTCGCTGCACTGAGGATTTTTAAAAAAAATTAGAATTCG
18-4-Female       AGCTGCCCGACTACTCTATATTCGCTGCACTGAGGATTTTTAAAAAAAATTAGAATTCG
18-5-Male         AGCTGCCCGACTACTCTATATTCGCTGCACTGAGGATTTTTAAAAAAAAATAGAATTCG

CS                GTAGCCTTTCGTGCTTAAAAACAATTCGTGTGTGAAAATAATAGGGCTGGGAGAT
18-4-Female       GTAGCCTTTCGTGCTTAAAAACAATTCGTGTGTGAAAATAATAGGGCTGGGAGAT
18-5-Male         GTAGCCTTTCGTGCTTAAAAACAATTCGTGTGTGAAAATAATAGGGCTGGGAGAT

CS                GCGGTAATTATCTTTTGGCACGAGTCCGCGTGCCGTGTAAGTTGGTGGAAAGACAA
18-4-Female       GCGGT---------------------------------------------------
18-5-Male         GCGGCT--------------------------------------------------
```

## TaDrAp1-D4

**Primers:**

**DrAp1-D4-SNP-F1: CACGAAAGGCTACCGAATTCTAA** 23 bp, 43%GC, 53.5C
DrAp1D4-SNP-F1 (RevCom): TTAGAATTCGGTAGCCTTTCGTG

**DrAp1-D4-SNP-F2: CACGAAAGGCTACCGAATTCTAT** 23 bp, 43%GC, 53.5C
DrAp1D4-SNP-F2 (RevCom): ATAGAATTCGGTAGCCTTTCGTG

**DrAp1-D4-SNP-R: GGTTGCAGGGCCAAAGGA** 18 bp, 61%GC, 52.6C

Amplicon size: 202 bp

**Ordered primers:**
**DrAp1-D4-SNP-F1:** GAAGGTGACCAAGTTCATGCT**CACGAAAGGCTACCGAATTCTAA**
**DrAp1-D4-SNP-F2:** GAAGGTCGGAGTCAACGGATT**CACGAAAGGCTACCGAATTCTAT**
**DrAp1-D4-SNP-R: GGTTGCAGGGCCAAAGGA**

## TaDrAp2-B1

**>CS**

GTGTTCCATGCCCTACTCTACCTTTCAGGCGACCGAACCAACACTCTGAGAAGCGCAGCCGACCGC
CGGACGTAGGCACGACGGAGGCGGACGAGCTCCGCTTAAAGATCGTTTTACGTTGCATGTAATAAT
ATGAATTTGAGGTTTCTACTTTTAGGTATCCGGTTATAAAATCAAACTTTTAAGACGTGATAGGTC
ATTGTCTGCGGACACGCCCAGACTTGTCTGCGGACATTTGAGGGGGCGGGTTTGCGAAGTACGACT
GTATATCCTCTTACACCCTGAAACATGATCCCTTTACGTTTAGTTAACACTCCTCAAAACAAAACA
CGACAAGATATGCCGACTCAGTCTTTCAGATGCGCTTATAGGAATAGAAAATGCGTGTGTGCGTTT
ATAGGGATAAGCGTATGCGCGTGTATATGAGCGCTTGCGTCTGTACCGTGTTTAAAAAAACACACG
GCAATCTATTCTGATCTCACAAAGACAAGTCGGAACATGCTATACCCAGATAAAGCGAGAGCCCGA
GAATGGACGATGACTCTCGCAGCCCAAGGCCACGAACGAGGCACAGCGCCACAGCCACCGGATCCC
GCCGACCCCCCGAATCGGACGGCGGACACAGGGAGCGCCACGAACCGGCCAGAAACGTGGGGCCCA
CCTCATCCGGGGAACGCGACGCGGACGGCTGACGTGCGGCCGCACGCGCAAACGAGGGAGAGGTCT
AACTACGCGGGCCCCTACATCGCGCCCAGCTTTTACCCACCGCGAAACGTCTCCATAGACCCGGTC
CGTCGCATCCCCACATGAACCTTCTACGGTCTTAGCGCCGCACGTGAGGCTAAGACCTATGCACCG
GGTCCAGGAGCTTAGCCTCCACGCGCCGGAGAGCTCGCGCACCTCTGCCCGCTTCGCGCGGCCATT
GGCCGCGCTGTTTATGGGGGAAAAATATATTTTTTCCCGTAAAAAAAGTGAAAAGATAGCGATAAC
GGAAACCCTAATCCCCAAATCAGAAATCTATCCGAATCGCTATTTATGTTTGTGGCTTTCTCCCCC
CTCCGATCCGATGTCCCCCGCTTCGTCCGCCGCGCCGATCCGACCTCCAGCCGCCGCATTCTCTCC
GCCGGGGACGCTCCCTCCGGCGCCGCCGGCTCCGCGCCATGAGGAAGAAGCTCGACACCCGCTTCC
CCGCGGTATGACCCATGCCCATGCGATTTTTTGCCGGTTCAGG

**>TaDrAp2-B1-18-4-Female**

TTTTTCCTCNNCNCNTCCNAACCTTTAAGGGGCCCGAACCAACCCCTTTGGAAAGGGGGACCGACC
CCCCGNACCTAGGCCCGACGGAGGCGGGCGAGCTTCGCTTAAAGATCCGTTTTACATTGCACGTAA
TAATATGAAATTGAAGGTTTGTACTCTTAGGTATCCGGGTTATAAATCAAACTTTTAAGATGTGAT
AGGTCATTGTNTGCGGACACGCCCAGAATTTTCTGTGGACATTTGAGGGGGCCGGGTTTGCGAAGT
ACGACTGTATATCCTCTTACACCCTGAAACATGATTCCTTTACGCTTAGTTAACACTCCTCAAAAC
AAAACACGACAAGATATGCCGACTCAGTCTTTCAGATGCGCTTATAGGAATAGAAAATGCGTGTGT
GCGTTTATAGGGATAAGCGTATGCGCGTATATATGAGCGCTTGCGTCTGTACCGCGTTTAAAACAA
CACACGGCAATCTATTCTGATCTCACAAAGACAAGTCGGAACATGCTCTACCCAGATAAAGCGAGA
GCCCGAGAATGGACGATGACTCTCGCAGCCCAAGGCCACGAACGAGGCACAGCGCCACAGCCACCG
GATCCCGCCGACCCCCCGAATCGGACGGCGGACACAGGGAGCGCCACGAACCGGCCAGAAACGTGG
GGCCCACCTCATCCGGGGAACGCGACGCGGACGGCTGACGTGCGGCCGCACGCGCAAACGAGGGAG
AGGTCTAACTACGCGGGCCCCTACATCGCGCCCAGCTTTTACCCACCGCGAAACGTCTCCATAGAC
CCGGTCCGTCGCATCCCCACATGAACCTTCTACGGTCTTAGCGCCGCACGTGAGGCTAAGACCTAT
GCACCGGGTCCAGGAGCTTAGCCTCCACGCGCCGGAGAGCTCGCGCACCTCTGCCCGCTTCGCGCG
GCCATTGGCCGCGCTGTTTATGGGGGAAAAAATAGTTTGTTTTATTTTTTCCCGTAAAAAAAGTGA
AAAGATAGCGATAACGGAAACCCTAATCCCCAAATCAGAAATCTATCCGAATCGCTATTTATGTTT
GTGGCTTTCTCCCCCCTCCGATCCGATGTCCCCCGCTTCGTCCGCCGCGCCGATCCGACCTCCAGC
CGCCGCGTTCTCTCCGCCGGGGACGCTCCCTCCGGCGCCGCCGGCTCCGCGCCATGAGGAAGAAGC
TCGACACCCGCTTCCCCGCGGTTGACCCCCCCCC

**>TaDrAp2-B1-18-5-Male**

TTTTACTTTCAGGCGACCGAACCAACATTTGAGAAGCGCAGCCGACCGCCGGACGTAGGCACGACG
GAGGCGGACGAGNTCCGCTTAAAGATCGTTTTACGTTGCATGTAATAATATGAATTTGAGGTTTCT
ACTTTTAGGTATCCGGTTATAAAATCAAACTTTTAAGA**C**GTGATAGGTCATTGTCTGCGGACACGC
CCAGACTTGTCTGCGGACATTTGAGGGGGCGGGTTTGCGAAGTACGACTGTATATCCTCTTACACC
CTGAAACATGATCCCTTTACGTTTAGTTAACACTCCTCAAAACAAAACACGACAAGATATGCCGAC

```
TCAGTCTTTCAGATGCGCTTATAGGAATAGAAAATGCGTGTGTGCGTTTATAGGGATAAGCGTATG
CGCGTGTATATGAGCGCTTGCGTCTGTACCGTGTTTAAAAAAACACACGGCAATCTATTCTGATCT
CACAAAGACAAGTCGGAACATGCTATACCCAGATAAAGCGAGAGCCCGAGAATGGACGATGACTCT
CGCAGCCCAAGGCCACGAACGAGGCACAGCGCCACAGCCACCGGATCCCGCCGACCCCCCGAATCG
GACGGCGGACACAGGGAGCGCCACGAACCGGCCAGAAACGTGGGGCCCACCTCATCCGGGGAACGC
GACGCGGACGGCTGACGTGCGGCCGCACGCGCAAACGAGGGAGAGGTCTAACTACGCGGGCCCCTA
CATCGCGCCCAGCTTTTACCCACCGCGAAACGTCTCCATAGACCCGGTCCGTCGCATCCCCACATG
AACCTTCTACGGTCTTAGCGCCGCACGTGAGGCTAAGACCTATGCACCGGGTCCAGGAGCTTAGCC
TCCACGCGCCGGAGAGCTCGCGCACCTCTGCCCGCTTCGCGCGGCCATTGGCCGCGCTGTTTATGG
GGGAAAAATATATTTTTTCCCGTAAAAAAAGTGAAAAGATAGCGATAACGGAAACCCTAATCCCCA
AATCAGAAATCTATCCGAATCGCTATTTATGTTTGTGGCTTTCTCCCCCCTCCGATCCGATGTCCC
CCGCTTCGTCCGCCGCGCCGATCCGACCTCCAGCCGCCGCATTCTCTCCGCCGGGGACGCTCCCTC
CGGCGCCGCCGGCTCCGCGCCATGAGGAAGAAGCTCGACACCCGCTTCCCCGCGGTTACCCCCNNN
N
```

**CLUSTAL 2.1 multiple sequence alignment**

## TaDrAp2-B1

```
18-5-Male       TTGAGGGGGCGGGTTTGCGAAGTACGACTGTATATCCTCTTACACCCTGAAACATGATC
CS              TTGAGGGGGCGGGTTTGCGAAGTACGACTGTATATCCTCTTACACCCTGAAACATGATC
18-4-Female     TTGAGGGGGCGGGTTTGCGAAGTACGACTGTATATCCTCTTACACCCTGAAACATGATC
                ***********************************************************

18-5-Male       CCTTTACGTTTAGTTAACACTCCTCAAAACAAAACACGACAAGATATGCCGACTCAGTCT
CS              CCTTTACGTTTAGTTAACACTCCTCAAAACAAAACACGACAAGATATGCCGACTCAGTCT
18-4-Female     CCTTTACGTTTAGTTAACACTCCTCAAAACAAAACACGACAAGATATGCCGACTCAGTCT
                ************************************************************

18-5-Male       TTCAGATGCGCTTATAGGAATAGAAAATGCGTGTGTGCGTTTATAGGGATAAGCGTATGC
CS              TTCAGATGCGCTTATAGGAATAGAAAATGCGTGTGTGCGTTTATAGGGATAAGCGTATGC
18-4-Female     TTCAGATGCGCTTATAGGAATAGAAAATGCGTGTGTGCGTTTATAGGGATAAGCGTATGC
                ************************************************************

18-5-Male       GCGTGTATATGAGCGCTTGCGTCTGTACCGTGTTTAAAAAAACACACGGCAATCTATTCT
CS              GCGTGTATATGAGCGCTTGCGTCTGTACCGTGTTTAAAAAAACACACGGCAATCTATTCT
18-4-Female     GCGTATATATGAGCGCTTGCGTCTGTACCGCGTTTAAAAAAACACACGGCAATCTATTCT
                **** ************************ ******************************

18-5-Male       GATCTCACAAAGACAAGTCGGAACATGCTATACCCAGATAAAGCGAGAGCCCGAGAATGG
CS              GATCTCACAAAGACAAGTCGGAACATGCTATACCCAGATAAAGCGAGAGCCCGAGAATGG
18-4-Female     GATCTCACAAAGACAAGTCGGAACATGCTCTACCCAGATAAAGCGAGAGCCCGAGAATGG
                ***************************** ******************************

18-5-Male       ACGATGACTCTCGCAGCCCAAGGCCACGAACGAGGCACAGCGCCACAGCCACCGGATCCC
CS              ACGATGACTCTCGCAGCCCAAGGCCACGAACGAGGCACAGCGCCACAGCCACCGGATCCC
18-4-Female     ACGATGACTCTCGCAGCCCAAGGCCACGAACGAGGCACAGCGCCACAGCCACCGGATCCC
                ************************************************************

18-5-Male       GCCGACCCCCCGAATCGGACGGCGGACACAGGGAGCGCCACGAACCGGCCAGAAACGTGG
CS              GCCGACCCCCCGAATCGGACGGCGGACACAGGGAGCGCCACGAACCGGCCAGAAACGTGG
18-4-Female     GCCGACCCCCCGAATCGGACGGCGGACACAGGGAGCGCCACGAACCGGCCAGAAACGTGG
                ************************************************************

18-5-Male       GGCCCACCTCATCCGGGGAACGCGACGCGGACGGCTGACGTGCGGCCGCACGCGCAAACG
CS              GGCCCACCTCATCCGGGGAACGCGACGCGGACGGCTGACGTGCGGCCGCACGCGCAAACG
18-4-Female     GGCCCACCTCATCCGGGGAACGCGACGCGGACGGCTGACGTGCGGCCGCACGCGCAAACG
                ************************************************************
```

```
18-5-Male      AGGGAGAGGTCTAACTACGCGGGCCCCTACATCGCGCCCAGCTTTTACCCACCGCGAAAC
CS             AGGGAGAGGTCTAACTACGCGGGCCCCTACATCGCGCCCAGCTTTTACCCACCGCGAAAC
18-4-Female    AGGGAGAGGTCTAACTACGCGGGCCCCTACATCGCGCCCAGCTTTTACCCACCGCGAAAC
               ************************************************************

18-5-Male      GTCTCCATAGACCCGGTCCGTCGCATCCCCACATGAACCTTCTACGGTCTTAGCGCCGCA
CS             GTCTCCATAGACCCGGTCCGTCGCATCCCCACATGAACCTTCTACGGTCTTAGCGCCGCA
18-4-Female    GTCTCCATAGACCCGGTCCGTCGCATCCCCACATGAACCTTCTACGGTCTTAGCGCCGCA
               ************************************************************

18-5-Male      CGTGAGGCTAAGACCTATGCACCGGGTCCAGGAGCTTAGCCTCCACGCGCCGGAGAGCTC
CS             CGTGAGGCTAAGACCTATGCACCGGGTCCAGGAGCTTAGCCTCCACGCGCCGGAGAGCTC
18-4-Female    CGTGAGGCTAAGACCTATGCACCGGGTCCAGGAGCTTAGCCTCCACGCGCCGGAGAGCTC
               ************************************************************

18-5-Male      GCGCACCTCTGCCCGCTTCGCGCGGCCATTGGCCGCGCTGTTTATGGGGGAAAAA-----
CS             GCGCACCTCTGCCCGCTTCGCGCGGCCATTGGCCGCGCTGTTTATGGGGGAAAAA-----
18-4-Female    GCGCACCTCTGCCCGCTTCGCGCGGCCATTGGCCGCGCTGTTTATGGGGGAAAAAATAGT
               *******************************************************

18-5-Male      ----TATATTTTTTCCCGTAAAAAAAGTGAAAAGATAGCGATAACGGAAACCCTAATCCC
CS             ----TATATTTTTTCCCGTAAAAAAAGTGAAAAGATAGCGATAACGGAAACCCTAATCCC
18-4-Female    TTGTTTTATTTTTTCCCGTAAAAAAAGTGAAAAGATAGCGATAACGGAAACCCTAATCCC
                   * ******************************************************

18-5-Male      CAAATCAGAAATCTATCCGAATCGCTATTTATGTTTGTGGCTTTCTCCCCCCTCCGATCC
CS             CAAATCAGAAATCTATCCGAATCGCTATTTATGTTTGTGGCTTTCTCCCCCCTCCGATCC
18-4-Female    CAAATCAGAAATCTATCCGAATCGCTATTTATGTTTGTGGCTTTCTCCCCCCTCCGATCC
               ************************************************************

18-5-Male      GATGTCCCCCGCTTCGTCCGCCGCGCCGATCCGACCTCCAGCCGCCGCATTCTCTCCGCC
CS             GATGTCCCCCGCTTCGTCCGCCGCGCCGATCCGACCTCCAGCCGCCGCATTCTCTCCGCC
18-4-Female    GATGTCCCCCGCTTCGTCCGCCGCGCCGATCCGACCTCCAGCCGCCGCGTTCTCTCCGCC
               ************************************************ ***********

18-5-Male      GGGGACGCTCCCTCCGGCGCCGCCGGCTCCGCGCCATGAGGAAGAAGCTCGACACCCGCT
CS             GGGGACGCTCCCTCCGGCGCCGCCGGCTCCGCGCCATGAGGAAGAAGCTCGACACCCGCT
18-4-Female    GGGGACGCTCCCTCCGGCGCCGCCGGCTCCGCGCCATGAGGAAGAAGCTCGACACCCGCT
               ************************************************************
```

## TaDrAp2-B1

**Primers:**

**DrAp2-B1-SNP-F1: ACAGACGCAAGCGCTCATATAT** 22 bp, 45%GC, 53C
DrAp2-B1-SNP-F1 (Rev-Com): ATATATGAGCGCTTGCGTCTGT

**DrAp2-B1-SNP-F2: ACAGACGCAAGCGCTCATATAC** 22 bp, 50%GC, 54.8C
DrAp2-B1-SNP-F2 (Rev-Com): GTATATGAGCGCTTGCGTCTGT

**DrAp2-B1-SNP-R: CACGACAAGATATGCCGACTCA** 22 bp, 50%GC, 54.8C
Amplicon size: 112 bp

**Ordered primers:**
**DrAp2-B1-SNP-F1:** GAAGGTGACCAAGTTCATGCT**ACAGACGCAAGCGCTCATATAT**
**DrAp2-B1-SNP-F2:** GAAGGTCGGAGTCAACGGATT**ACAGACGCAAGCGCTCATATAC**
**DrAp2-B1-SNP-R: CACGACAAGATATGCCGACTCA**

# Supplementary file 5

## Primer design and sequences for RT-qPCR

### TaDrAp1

**TaDrAp1-Total-Fq: GACGCCAGCTGTGATTTCAAAG** 22bp; 50% GC; 54.8C
**TaDrAp1-Total-Rq: GTACAACACCAGGAGAACTGTC** 22bp; 50% GC; 54.8C
TaDrAp1-Total-Rq (RevCom): GACAGTTCTCCTGGTGTTGTAC
Amplicon size = 219 bp

**TaDrAp1-A4-Fq: AGTGGAGGCTCCATCAGCCA** 20bp; 60% GC; 55.9C
**TaDrAp1-A4-Rq: ATGACTATGCCTAATCCTCATTG** 23bp; 39% GC; 51.7C
TaDrAp1-A4-Rq (RevCom): CAATGAGGATTAGGCATAGTCAT
Amplicon size = 191 bp

**TaDrAp1-B4-Fq: CTAGTGGAGGCTCCATCGGT** 20bp; 60% GC; 55.9C
**TaDrAp1-B4-Rq: GCATATGATGAATGAGAGGATGG** 23bp; 43% GC; 53.5C
TaDrAp1-B4-Rq (RevCom): CCATCCTCTCATTCATCATATGC
Amplicon size = 209 bp

**TaDrAp1-D4-Fq: TGAGGATGTTGGCAAAATTGCG** 22bp; 45% GC; 53C
**TaDrAp1-D4-Rq: ATTCACCGCCACCAAGATCC** 20bp; 55% GC; 53.8C
TaDrAp1-D4-Rq (RevCom): GGATCTTGGTGGCGGTGAAT
Amplicon size = 221 bp

### CLUSTAL 2.1 multiple sequence alignment

```
B4              ATGAGGAAGAAGCTGGGCACCCGGTTCCCCGCGGCACGGATCAAAAAGATAATGCAAGCA
D4              ATGAGGAAGAAGCTGGGCACCCGGTTCCCCGCGGCACGGATCAAAAAGATAATGCAAGCA
A4              ATGAGGAAGAAGCTGGGCACCCGGTTCCCCGCGGCACGGATCAAAAAGATAATGCAAGCA
                ************************************************************

B4              GATGAGGATGTTGGCAAAATTGCACTGGCTGTGCCTGTTTTAGTTTCGAGAGCCCTTGAA
D4              GATGAGGATGTTGGCAAAATTGCGCTGGCTGTGCCTGTTTTAGTTTCGAGAGCCCTTGAA
A4              GATGAGGATGTTGGCAAAATTGCACTGGCTGTGCCTGTTTTAGTTTCGAGAGCCCTTGAA
                *********************** ************************************

B4              TTGTTTCTGCAAGATTTGATTGACCGCTCATACAAAATTACTCTTCAAAGTGGTGCAAAG
D4              TTGTTTCTGCAAGATTTGATTGACCGCTCATACAAAATTACTCTTCAAAGTGGTGCAAAG
A4              TTGTTTCTGCAAGATTTGATCGACCACTCATACAAAATTACTCTTCAAAGTGGTGCAAAG
                ******************** **** **********************************

B4              ACACTGAATTCCTTCCACCTAAAGCAATGTGTGAAGAGGTACAGCTCTTTTGACTTCCTA
D4              ACACTGAATTCCTTCCACCTAAAGCAATGTGTGAAGAGGTACAGCTCTTTTGACTTCCTA
A4              ACACTGAATTCCTTCCACCTAAAGCAATGTGTGAAGAGGTACAGCTCTTTTGACTTCCTA
                ************************************************************

B4              ACTGAGATTGTCAACAAGGTGCCAGATCTCGGTGGCGGTGAATCTTGTGGAGATGAAAGA
D4              ACTGAGATTGTCAACAAGGTGCCGGATCTTGGTGGCGGTGAATCTTGTGGAGATGAAAGA
A4              ACTGAGATTGTCAACAAGGTGCCAGATCTCGGTGGCGGTGAATCTTGTGGAGATGAAAGA
                *********************** ***** ******************************

B4              GGATTACCCAGAAGAAGGAAATTTTCAAATGGAAGCGACCCAGAGAATGAGGAGCCCCGA
D4              GGATTACCCAGAAGAAGGAAATTTTCAAATGGAAGCGACCCAGAGAATGAGGAGCCCCGA
A4              GGATTACCCAGAAGAAGGAAATTTTCAAATGGAAGCGACCCAGAGAATGAGGAGCCCCGA
                ************************************************************
```

```
B4          TCTAGCAAAATGCCCATAAGAAGCTTGAACACTAGTCCCAGAGGACGAGGCAGAGGTCGA
D4          TCTAGCAAAATGCCCATAAGAAGCTTGAACACTAGTCCCAGAGGACGAGGCAGAGGTCGA
A4          TCTAGCAAAATGCCCATAAGAAGCTTGAACACCAGTCCCAGAGGACGAGGCAGAGGTCGA
            ******************************** ***************************

B4          GGAAGAGGGCGAGGGCGGCCTCCAACCAAGAGAAAGGAAATTGGTTATGTACAGTTTGAG
D4          GGAAGAGGGCGAGGGCGGCCTCCAACCAAGAGAAAGGAAATTGGTTATGTACAGTTTGAG
A4          GGAAGAGGGCGAGGGCGGCCTCCAACCAAGAGAAAGGAAATTGGTTATGTACAGTTTGAG
            ************************************************************

B4          GATGAGAGCAGCATGTTTGCTGAACAAAGTGAACCCTTGCCAGGAGATGAGATAGTTCCA
D4          GATGAGAGCAGCATGTTTGCTGAACAAAGTGAACCCTTGCCAGGAGATGAGATAGTTCCA
A4          GATGAGAGCAGCATGTTTGCTGAACAAAGTGAACCCTTGCCAGGAGATGAGATAGTTCCG
            *********************************************************** 

B4          GAGACCAACCGTGGCAATGAGATTATTCCCCAAAGCTCACATCCTCTAGTGGAGGCTCCA
D4          GAGACCAACCGTGGCAATGAGAGTATTCCCCAAAGCTCACATCCTCTAGTGGAGGCTCCA
A4          GAGACCAACCGTGGCAATGAGAGTATTCCCCAAAGCTCACATCCTCTAGTGGAGGCTCCA
            ********************** *************************************

B4          TCGGTCGTGACGCCAGCTGTGATTTCAAAGGTTGAAAAAGC---CACCAACCATCAGCCA
D4          TCAGCCGCGACGCCAGCTGTGATTTCAAAGGTTGAAAAAGCTAGCACCAACCATCAGCCA
A4          TCAGCCATGACGCCAGCTGTGATTTCAAAGGTTGAAGAAGCTAGCACCAACCATCAGCCA
            ** * *  **************************** ****   ****************

B4          GATTTGCCTATGCCAGATGCCATTGGAGGCATTGGTGTTGGACCGTCCAGTTTTGGACAT
D4          GATTTGCCTATGCCAGATGCCATTGGAGGCATTGGTGTTGGACCATCCAGTTTTGGACAT
A4          GATTGGCCTATGCCAGATGCCATTGGAGGCATTGGTGTTGGACCATCCAGTTTTGGACAT
            **** *************************************** ***************

B4          CTGACAGTGCAGGTTGATGAGGAAGAGGACTACGATAATGAGGATTAGGCATAGCCATCC
D4          CTGACGGTGCAGGTTGATGAGGAAGAGGACTACGATAATGAGGATTAGGCATAGTCATCC
A4          CTGACAGTGCAGGTTGATGAGGTAGAGGACTACGACAATGAGGATTAGGCATAGTCATCC
            ***** **************** ************ ****************** *****

B4          TCTCATTCATCATATGCTCTAACAGGACAGTTCTCCTGGTGTTGTACATTGTAAATATTG
D4          TCTCATTCATCATATGCTCTAACAGGACAGTTCTCCTGGTGTTGTACATTGTAAATATTG
A4          TCTCATTCATCATATGCTCTAACAGGACAGTTCTCCTGGTGTTGTACATTGTAAATATTG
            ************************************************************

B4          TTTCAAGTAGTTACCGCAGCTATGATGTGTAACCTATTTCTTTTTCCCAATAATTGGTTT
D4          TTTCAAGTAGTTACCGCAGCTATGATGTGTAACCTATTTCTTTTTCCCAATAATTGGTTT
A4          TTTCAAGTAGTTACTGCAGCTATGATGTGTAACCTATTTCTTTTTCCCAATAATTGGTTC
            ************** ******************************************** 

B4          CGAGTTGCCTCGCATTGTTGACTTAACTGACTAGGATGTGCTAAACTTAACCTTGTATTT
D4          CGAGTTGCCTCGCACTGTTGACTTAACTGACTAGGATGTGCTAAATTTAACCCTGTATTT
A4          TGAGTTGCC--GCATTGTTGACCTAACTGACTAGGATGTGCTAAATTTAACCCTGTATTT
             ********  *** ******* ********************** ****** *******

B4          GACGGTTGAAGCAGTGATATTCTGTCGTCCTGTTACATTGTACATGTTCTAACCAGCAGT
D4          GACGGTTGAAGCAGTGATATTCTGTCGTCCTGTTACATTGTACATGTTGTAACCAGCAGT
A4          GACGGTTGAAGCAGTGATATTCTGTCGTCCTGTTATATTGTACATGT---AACCAGCAGT
            *********************************** ***********   **********

B4          AACTTTTT----CTAGTATATCGTGTCGTGACCATCATGCTACGAAATCACGTCATTCAG
D4          AATTATTT----CTAGTATATCATGTCGTGACCATCATGCCACGAAATCACGTCATTCAG
A4          AATTTTGTGTTACTAGTATATCATGTCGTGACCATCATGCCACGAAATCA-----TTCAG
            ** * * *    ********** ***************** *********     *****
```

## TaDrAp2

**TaDrAp2-Total-Fq: TCACAGTTGAAAGAAGAAGTGG** 22bp; 41% GC; 51.1C
**TaDrAp2-Total-Rq: CGTCCTCCTCTAATCTATGGT** 21bp; 48% GC; 52.4C
TaDrAp2-Total-Rq (RevCom): ACCATAGATTAGAGGAGGACG
Amplicon size = 124 bp

**TaDrAp2-A1-Fq: GCAAAAGAAGGAAACATGGTGAG** 23bp; 43% GC; 53.5C
**TaDrAp2-A1-Rq: TGGCATTGCTCAAAGGGGTG** 20bp; 55% GC; 55.8C
TaDrAp2-A1-Rq (RevCom): CACCCCTTTGAGCAATGCCA
Amplicon size = 267 bp

**TaDrAp2-B1-Fq: GAAGAATGATGTGACTCTAGTTTG** 24bp; 38% GC; 52.3C
**TaDrAp2-B1-Rq: AGCAACCATGATTTACAACCGGA** 23bp; 43% GC; 53.5C
TaDrAp2-B1-Rq (RevCom): TCCGGTTGTAAATCATGGTTGCT
Amplicon size = 253 bp

**TaDrAp2-D1-Fq: AGTCCAGCCTTCGGCTCCT** 19bp; 63% GC; 55.4C
**TaDrAp2-D1-Rq: CTAAATCCACGCTAGAGTCACG** 22bp; 50% GC; 54.8C
TaDrAp2-D1-Rq (RevCom): CGTGACTCTAGCGTGGATTTAG
Amplicon size = 242 bp

**CLUSTAL 2.1 multiple sequence alignment**

```
A1              ATGAGGAAGAAGCTCGACACCCGCTTCCCCGCGCCTCGGATTAAGAAGATCATGCAAGCA
D1              ATGAGGAAGAAGCTCGACACCCGCTTCCCCGCGCCTCGGATTAAGAAGATCATGCAAGCA
B1              ATGAGGAAGAAGCTCGACACCCGCTTCCCCGCGCCTCGGATTAAGAAGATCATGCAAGCA
                ************************************************************

A1              GATGAAGATGTCGGCAAGATTGCTCTAGCTGTACCTGTTCTAGTTTCCAAAGCGTTGGAG
D1              GATGAAGATGTCGGCAAGATTGCTCTAGCAGTACCTGTTCTAGTTTCCAAAGCGTTGGAG
B1              GATGAAGATGTCGGCAAGATTGCTCTAGCTGTACCTGTTCTAGTTTCCAAAGCGTTGGAG
                ***************************** ******************************

A1              CTATTTTTGCAAGACTTATGTGATAGGACATATAATATCACTGTTGAAAAAGGGGTCAAA
D1              CTATTTTTGCAAGACTTATGTGATAGGACATATAATATCACTGTTGAAAAAGGGGTCAAA
B1              CTATTTTTGCAAGACTTATGCGATAGGACATATAACATCACTGTTGAAAAAGGGGTCAAA
                ******************** ************** ************************

A1              ACAGTGAGTTCATCACATCTGAAACAGTGCATTCATAGTTACGATGTGTATGATTTTTTG
D1              ACAGTGAGTTCATCACATCTGAAACAGTGCATTCATAGTTACGATGTGTATGATTTTTTG
B1              ACAGTGAGTTCATCACATCTGAAACAGTGCATTCATAGTTACGATGTGTATGATTTTTTG
                ************************************************************

A1              AAGAACGTTGCCAGCAAAGTTCCAGATTTGGGTGCACCTGATTCTAGTGTTGATGATAAG
D1              AAGAACGTAGCCAGCAAAGTTCCAGATTTGGGTGCACCTGATTCTAGTGTTGATGATAAA
B1              AAGAACGTTTCTAGCAAAGTTCCAGATTTGGGTCCACCTGATTCTAGTGTTGATGATAAG
                ********  * ********************* ************************* 

A1              CTTGGCAAAAGAAGGAAACATGGTGAGGATGAAAGCGAGGAGGAATCGAAGCGGACAAGA
D1              CTTGGCAAAAGAAGGAAACATGGTGAAGATGAAAGCGAGGAGGAATCGAAGCGGACAAGA
B1              CTTGGCAAAAGAAGGAAACATGGTGAAGATGAAAGCGAGGAGGAATCGAAGCGGACAAGA
                ************************** *********************************

A1              AATGAGGCAGCATGCCACACAAGCAACGGCAGAGGGCGTGGGAGGGGACGTGGGAGAGGT
D1              AATGAGGCAGCATGCCACACAAGCAACGGCAGAGGGCGCGGGAGGGGACGTGGGAGAGGT
B1              AATGAGGCAGCATGCCACACAAGCAACGGCAGAGGGCGTGGGAGGGGACGTGGGAGAGGT
                ************************************** *********************
```

```
A1          CGCCGTGCTGGGCGTGGAGCTGAGAGGGAGATTGAACACCATGAATTAGGTTGTGCCCAA
D1          CGCCGTGCTGGGCGTGGAGCTGAGAGGGAGATTGAACACCATGAATTAGGTTGTGCCCAA
B1          CGCCGTGCTGGGCGTGGAGCTGAGAGGGAGATTGAACACCATGAATTAGGTTGTGCCCAA
            ************************************************************

A1          TTTAGCAAACCAGGCCACCTGAAGGTAGAGATTGGAGACGGGGTTTTGGACACAAGTGAA
D1          TTTAGCAAACCAGGCCACCTGAAGGTAGAGATTGGAGACGTTGTTCTGGACACAAGTGAA
B1          TTTAGCAAACCAGGCCACCTGAAGGTAGAGATTGGAGACGTTGTTCTGGACACAAGTGAA
            ****************************************  *** **************

A1          ACCAAAGAGCACACCCCTTTGAGCAATGCCAGGGCCTCCTTAAGGAACATTGACTTGAAC
D1          ACCAAAGAGCATACCCCTTTGAGCAATGCCAGGGCCTCCTTAAGGAACATTGACTTGAAC
B1          ACCAAAGAGCATACCCCTTTGAGCAATGCCAGGGCCTCCTTAAGGAACATTGACTTGAAC
            *********** ************************************************

A1          TTAGATCTTGCTGACTACGAGGAGGATACAGTGGCGCCGCAAGTCCAGCCTTC-------
D1          TTAGATCTTGCTGACTACGAGGAGGATAC------GCCGCAAGTCCAGCCTTCG GCTCCT
B1          TTAGATCTTGCTGACTACGAGGAGGATACAGTGGCGCCGCAAGTCCAGCCTTC-------
            *****************************      ******************

A1          --AGCTCCTGTGGTGACCGTGGCGCCGCAAGTCCAGACTTCAGGACCATCGGTTTCACAG
D1          TCAGCTCCTGTGGTGACCGTGGCACCGCAAGTCCAGACTTCAGGACCGTCGGTTTCACAG
B1          --AGCTCCTGTGGTGACCGTGGCACCGCAAGTCCAGACTTCAGGACCATCGGTTTCACAG
              ********************* *********************** ************

A1          TTGAAAGAAGAAGTGGCGACCAAGGATTTCCTGGGCTGGCAAATGCCTGAGATGAACAAG
D1          TTGAAAGAAGAAGTGGAGACCAAGGATTTCCTGGGCTGGCAAATGCCTGAGATGAACAAG
B1          TTGAAAGAAGAAGTGGAGACCAAGGATTTCCTGGGCTGGCAAATGCCTGAGATGAACAAG
            **************** *******************************************

A1          ATGGCCATGGACCCGGTGCAGTTTGCGCTGTCGTCAAACCATAGATTAGAGGAGGACGAA
D1          ATGGCCATGGACCCGGTGCAGTTTGCGCTGTCGTCAAACCATAGATTAGAGGAGGACGAA
B1          ATGGCCATGGACCCGGTCCAGTTTGCGTTGTCGTCAAACCATAGATTAGAGGAGGACGAA
            ***************** ********* ********************************

A1          GACTATGACAATGAAGAATGATGTGACTCTAGCGTGGATTTAGTTTGAGAAGCTGATAGG
D1          GACTATGACAATGAAGAATGACGTGACTCTAGCGTGGATTTAGTTTGAGAAGCTGATAGG
B1          GACTATGACAATGAAGAATGATGTGACTC-----------TAGTTTGAGAAGCTGATAGG
            ********************* *******           ********************

A1          TAGACATGACATGATGTACATCCTAGCAGGCCCAGCTTGACAATGTAGTAGCAATTCTCT
D1          TAGACATGACATGATGTACATCCTAGCAGGCCCAGCTTGACAATGTAGTAGCAATTCTCT
B1          TAGACATGACATGATGTACATCCTAGCAGGCCAAGCTTGACAATGTAGTAGCAATTCTCT
            ******************************** ***************************

A1          TGTAGTATGTGTACCCGCAGATGCTGCAGAAGTAATTTAGGATGTGACATATGTTGATTG
D1          TGTAGTATGTGTACCCGCAGATGCTGCAGAAGTGATTTAGGATGTGACATATGTTGATTG
B1          TGTAGTATGTGTACTGGCAGATGCTGCAGAAGTAATTTAGGATGTGACATATGTTGATTG
            **************  ***************** **************************

A1          CTCAATAGCCAGTAGTAGCTAGTATCTTGTGTAGCAACTATATAGTGTAGCGACTATATA
D1          CTCAATAGCCAGTAGTAGCTAGTATTTTGTGTAGCAACTATATAGTGTAGCAACTATATA
B1          CTCAATAGCCAGTAGTAGCTAGTATTTTGTGTAGCAACTATATAGTGTAGCAACTATATA
            ************************* ************************* ********

A1          ACTAGACATGTTCGCCGGTTGTAAATCATGGTTGCTTTTAGATCAAATGCTATGTCATAA
D1          ACTAGACAGGTTCGCCGGTTGTAAATCATGGTTGCTTTTAGATCAAATGCTATGTCATAA
B1          ACTAGACAGGTTCTCCGGTTGTAAATCATGGTTGCTTTTAGATCAAATGCTATGTCATAA
            ******** **** **********************************************
```
